# Home-based transcranial direct current stimulation treatment for major depressive disorder: a fully remote phase 2 randomized sham-controlled trial

**DOI:** 10.1038/s41591-024-03305-y

**Published:** 2024-10-21

**Authors:** Rachel D. Woodham, Sudhakar Selvaraj, Nahed Lajmi, Harriet Hobday, Gabrielle Sheehan, Ali-Reza Ghazi-Noori, Peter J. Lagerberg, Maheen Rizvi, Sarah S. Kwon, Paulette Orhii, David Maislin, Lucia Hernandez, Rodrigo Machado-Vieira, Jair C. Soares, Allan H. Young, Cynthia H. Y. Fu

**Affiliations:** 1 School of Psychology, University of East London, London, UK; 2 Center of Excellence on Mood Disorders, Faillace Department of Psychiatry and Behavioral Sciences, McGovern Medical School, University of Texas Health Science Center at Houston, Houston, TX USA; 3 Intra-Cellular Therapies Inc, New York, NY USA; 4 Paul L. Foster School of Medicine, Texas Tech University Health Sciences Center at El Paso, El Paso, TX USA; 5 Biomedical Statistical Consulting, Philadelphia, PA USA; 6 Centre for Affective Disorders, Institute of Psychiatry, Psychology and Neuroscience, King’s College London, London, UK; 7 National Institute for Health Research, Biomedical Research Centre at South London and Maudsley NHS Foundation Trust, King’s College London, London, UK; 8 South London and Maudsley NHS Foundation Trust, Bethlem Royal Hospital, Beckenham, UK

**Keywords:** Medical research, Depression

## Abstract

Transcranial direct current stimulation (tDCS) has been proposed as a new treatment in major depressive disorder (MDD). This is a fully remote, multisite, double-blind, placebo-controlled, randomized superiority trial of 10-week home-based tDCS in MDD. Participants were 18 years or older, with MDD in current depressive episode of at least moderate severity as measured using the Hamilton Depression Rating Scale (mean = 19.07 ± 2.73). A total of 174 participants (120 women, 54 men) were randomized to active (*n* = 87, mean age = 37.09 ± 11.14 years) or sham (*n* = 87, mean age = 38.32 ± 10.92 years) treatment. tDCS consisted of five sessions per week for 3 weeks then three sessions per week for 7 weeks in a 10-week trial, followed by a 10-week open-label phase. Each session lasted 30 min; the anode was placed over the left dorsolateral prefrontal cortex and the cathode over the right dorsolateral prefrontal cortex (active tDCS 2 mA and sham tDCS 0 mA, with brief ramp up and down to mimic active stimulation). As the primary outcome, depressive symptoms showed significant improvement when measured using the Hamilton Depression Rating Scale: active 9.41 ± 6.25 point improvement (10-week mean = 9.58 ± 6.02) and sham 7.14 ± 6.10 point improvement (10-week mean = 11.66 ± 5.96) (95% confidence interval = 0.51–4.01, *P* = 0.012). There were no differences in discontinuation rates. In summary, a 10-week home-based tDCS treatment with remote supervision in MDD showed high efficacy, acceptability and safety. ClinicalTrials.gov registration: NCT05202119

## Abstract (web summary)

A randomized, sham-controlled, superiority trial of a 10-week course of home-based transcranial direct current stimulation found greater improvements in depressive symptoms with active compared to sham stimulation in major depressive disorder.

## Main

Major depressive disorder (MDD) is common and it is a leading cause of disability worldwide; it is the most notable precursor in suicide^1^. MDD is characterized by a prolonged low mood or inability to experience usual feelings of pleasure, which is accompanied by disturbances in sleep, appetite, psychomotor functioning and energy levels, and in cognitive functioning. First-line treatments are antidepressant medications and psychological therapies. However, more than a third of individuals with MDD do not achieve full clinical remission despite full treatment trials^2,3^.

Transcranial direct current stimulation (tDCS) is a form of noninvasive brain stimulation that applies a weak (0.5–2 mA) direct current via scalp electrodes^4^. Anodal stimulation shifts membrane potentials toward depolarization and increasing cortical excitability, whereas cathodal stimulation tends to shift membrane potentials toward hyperpolarization, decreasing potential cell firing and inhibiting cortical excitability^5^. tDCS modulates the resting state potential, thereby modulating cortical tissue excitability, rather than directly triggering an action potential that is in contrast to repetitive transcranial magnetic stimulation^6^. Neurophysiological effects typically persist beyond the immediate stimulation period^7^. Anodal tDCS can enhance cortical excitability, which is dependent on the *N*-methyl-d-aspartate receptor and calcium channel activity, demonstrating a sustained increase in synaptic transmission that is long-term potentiation-like, whereas cathodal tDCS decreases excitability and facilitates long-term depression-like changes^8^. Neural recordings demonstrate measurables effects on cortical electric fields^9^. Neurophysiological measures reveal network-level modulatory effects, in which anodal tDCS applied to left dorsolateral prefrontal cortex (DLPFC) is associated with significant changes in connectivity in default mode, self-referential and frontoparietal networks compared with sham tDCS^10^; it can extend into the deeper limbic brain regions, including the amygdala^11^, which are key regions in MDD neurocircuitry and reflect potential mechanisms of effect^4^.

tDCS is applied through a flexible cap or band that is worn over the forehead. The anode electrode is typically placed over the left DLPFC and the cathode is placed over the right DLPFC, in the suborbital or frontotemporal region^6^. In an individual-patient data meta-analysis, active tDCS relative to sham tDCS was associated with a significantly greater rate of clinical response (30.9% versus 18.9%; number needed to treat (NNT) = 9) and remission (19.9% versus 11.7%; NNT = 13) from 572 participants with MDD in nine studies^12^. tDCS is safe and well tolerated with no significant differences in attrition rate and adverse events between active and sham stimulation, offering a potential new first-line treatment for MDD^4^. However, a course of tDCS treatment involves daily sessions for several weeks; most studies have been conducted in a research clinic and have required daily visits^6,12^.

As it is portable and safe, tDCS can be provided for home use^4^. We developed a protocol that provides home-based tDCS with real-time remote supervision using videoconferencing^13^. In MDD, we found significant improvements in depressive symptoms, high acceptability and feasibility^13^, as also observed in additional open-label trials^14,15^. However, in our protocol, all participants had both the active tDCS device and real-time visits using videoconferencing, which were associated with meaningful experiences of support and containment^16^. Three randomized controlled trials (RCTs) of home-based tDCS in MDD have taken place^17–19^; however, none were fully remote because all included in-person study appointments, two trials were probably underpowered because of small sample sizes (*n* = 11 (ref. ^18^) and *n* = 58 (ref. ^19^)), and all were limited to a 6-week duration; they found no significant effects of active relative to sham tDCS^17–19^. However, the recent meta-analysis by Nikolin et al.^20^ reported that the active tDCS effects continue to increase for up to 10 weeks compared to sham stimulation.

In the present study, we sought to investigate the clinical efficacy and safety of a 10-week course of home-based tDCS for MDD in a large, double-blind, randomized superiority trial conducted in both the UK and USA. All participants had MDD as determined by a structured diagnostic interview; all were in a current depressive episode of at least moderate severity. Participants in our study might be taking stable antidepressant medication for at least 6 weeks, might be in psychotherapy for at least 6 weeks or might be treatment-free, reflecting the range of forms of MDD from first-episode and recurrent MDD to treatment-resistant depression. All study visits were remote and we were able to monitor participants’ tDCS use in real time. The primary objective was to investigate clinical efficacy at the 10-week end point of treatment between active and sham tDCS treatment arms.

## Results

### Participant data

Recruitment was from 12 May 2022 to 10 March 2023 (ClinicalTrials.gov registration: NCT05202119). From 2,234 individuals who had an initial telephone screen, 368 individuals provided written informed consent and had an assessment using Microsoft Teams videoconferencing. In total, 174 participants with MDD (120 women, 69%) with a mean age of 37.63 years (s.d. = 11.00) were enrolled. One hundred and forty-five (83.3%) had white ethnicity. All had an MDD diagnosis based on the criteria of the *Diagnostic and Statistical Manual of Mental Disorders*, Fifth Edition^21^, were assessed using a structured clinical interview^22^ and were in a current depressive episode of at least moderate severity as measured by a minimum score of 16 in 17-item Hamilton Depression Rating Scale (HDRS)^23^. The mean HDRS was 19.07 (s.d. = 2.73); the median number of depressive episodes was three (interquartile range (IQR) = 1–5) (Table 1). The sex of participants was based on self-report. There were no exclusions of participants based on either sex or gender.

**Table 1:** Demographic and clinical characteristics of participants at baseline

Characteristic	Active	Sham
Number of participants	87	87
Age	37.09 ± 11.14	38.32 ± 10.92
Sex		
Women	54 (62)	66 (76)
Ethnicity		
Asian	9 (10)	2 (2)
Black or African American	3 (3)	1 (1)
Native Hawaiian or Other	0 (0)	0 (0)
White	72 (83)	73 (84)
Other	3 (3)	11 (13)
Missing	0 (0)	0 (0)
Educational level		
Lower than high school or secondary school	1 (1)	0 (0)
Some college education	18 (21)	19 (22)
Diploma	9 (10)	7 (8)
Bachelor’s or Professional Degree	37 (43)	37 (43)
Master’s or Doctoral Degree	22 (25)	23 (26)
Preferred not to answer/missing	0 (0)	1 (1)
Age of onset of MDD, years	22.08 ± 9.68	22.40 ± 8.78
Previous number of episodes	4.11 (0–30)	4.80 (0–30)
Previous number of suicide attempts	0.10 (0–2)	0.16 (0–2)
First episode of MDD	18 (21)	10 (11)
Clinical ratings		
HDRS	19.18 ± 2.83	18.92 ± 2.63
HDRS severity:		
Moderate (HDRS score: 16–18)	45 (52)	45 (52)
Severe (HDRS score: 19–22)	29 (33)	33 (38)
Very severe (HDRS score: 23 or greater)	13 (15)	9 (10)
MADRS	24.72 ± 4.68	23.87 ± 5.49
MADRS-s	26.77 ± 6.90	25.67 ± 6.34
HAM-A	15.45 ± 4.61	14.25 ± 4.57
YMRS	2.10 ± 1.72	1.92 ± 1.58
EQ-5D-3L	0.75 ± 0.13	0.75 ± 0.14
RAVLT	57.92 ± 11.15	58.51 ± 13.40
SDMT	52.26 ± 10.13	50.40 ± 10.14
Taking antidepressant medication	56 (64)	53 (61)
Selective serotonin reuptake inhibitor	40 (46)	35 (40)
Nonselective monoamine reuptake inhibitor	1 (1)	3 (3)
Other antidepressant medications	18 (21)	17 (20)
Taking combination of antidepressant medications	5 (6)	3 (4)
In psychotherapy during the trial	12 (14)	14 (16)
In psychotherapy and taking antidepressant medication	6 (7)	12 (14)
No antidepressant medication or psychotherapy during the trial	25 (29)	32 (37)

Categorical variables are presented as the number of participants with percentage in parentheses. Mean values are presented as ‘±’ the s.d. The previous number of episodes and suicide attempts are presented as the mean with the range (median (IQR): previous number of episodes; active = 3 (1–5), sham = 3 (1.5–5); previous number of suicide attempts; active = 0 (0), sham = 0 (0)). Diploma, a certificate that signifies a certain level of education and practical experience. SDMT active, *n* = 85, SDMT sham, *n* = 85. Age at onset, active *n* = 86, sham *n* = 86. HDRS scores range from 0 to 52, MADRS scores range from 0 to 60 and MADRS-s scores range from 0 to 54, with higher scores indicating increased depressive symptom severity. RAVLT scores range from 0 to 75. SDMT scores range from 0 to 110. A two-sided significance test (Fisher’s exact test for categorical variables or *t*-test for continuous variables) found a significant difference between groups for ethnicity (*P* = 0.012). *P* > 0.05 for all other characteristics.

Inclusion criteria included being treatment-free or taking stable antidepressant medication or undergoing psychotherapy for at least 6 weeks before enrollment. Having persistent depressive symptoms of at least moderate severity and meeting the MDD criteria while taking antidepressant medication for at least 6 weeks have been clinical criteria for treatment-resistant depression in previous trials^24,25^. The composition of the participant cohort was as follows: treatment-free = 57 (32.8%); taking antidepressant medication = 109 (62.6%); undergoing psychotherapy = 26 (14.9%); taking medication and undergoing psychotherapy = 18 (10.3%) participants.

**Fig. 1:**
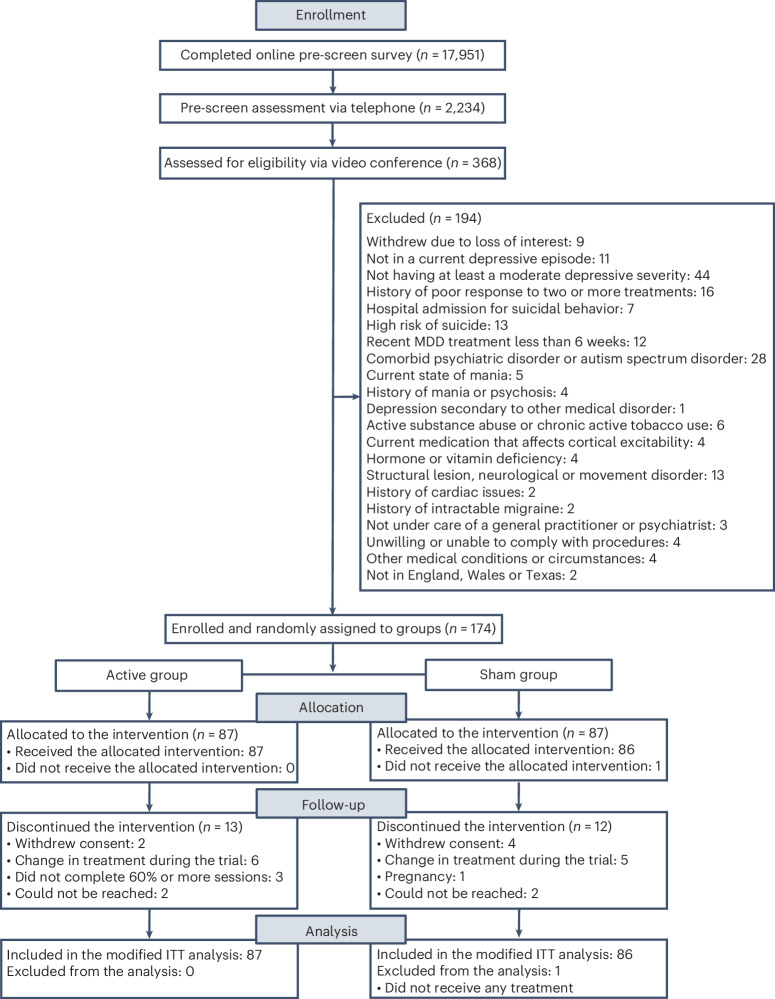
CONSORT diagram. Enrollment, group allocation, follow-up and analysis.

Participants were randomly allocated to active tDCS treatment (87 with MDD, mean age = 37.09 years (s.d. = 11.14)) or sham tDCS (87 with MDD, mean age = 38.32 years (s.d. = 10.92)) (Fig. 1, Table 1 and Supplementary Tables 2–5). One participant did not continue and had not started any treatment; therefore, the modified intention-to-treat (ITT) sample was 173 participants. There were no significant differences in discontinuation rates between groups (total = 25 participants, 14.3%: 13 (14.9%) in the active group and 12 (13.7%) in the sham group (*P* = 0.99)) (Supplementary Table 6). Based on a priori blinded interim analysis, recruitment ended early (Supplementary Notes—Interim Analysis).

### Primary outcome

**Fig. 2:**
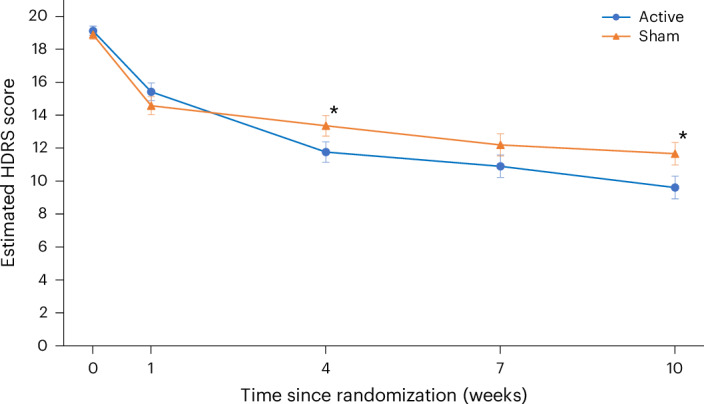
Change in depressive severity ratings over time. Estimated mean 17-item HDRS rating scores from baseline to week 10 in the modified ITT analysis sample (*n* = 173) for the active and sham tDCS treatment arms. The error bars represent ± 1 s.e. The HDRS scores range from 0 to 52, with higher values indicating more severe depressive symptoms. A significant improvement was observed in the change in HDRS ratings from baseline to week 10 in the active tDCS treatment arm, that is, an HDRS decrease of 9.41 ± 6.25 (s.d.) (mean HDRS at week 10 = 9.58 ± 0.70 (s.e.)), compared to the sham tDCS treatment arm (HDRS decrease = 7.14 ± 6.10 (s.d.)) (mean HDRS at week 10 = 11.66 ± 0.69 (s.e.)) (95% CI = 0.5–4.0, *P* = 0.012). The difference in change scores was also significant at week 4 (95% CI = 0.2–3.4, *P* = 0.03), with a greater score decrease in the active treatment arm. A fully conditional specification (FCS) approach was used to produce 20 multiply imputed complete datasets. The FCS approach accommodates nonmonotonicity in the pattern of missing data and requires regression models to be specified for each variable, with missing values needing imputation. All models included age, sex, undergoing psychotherapy at baseline, use of any antidepressants at baseline and treatment group. The resulting complete datasets were combined using Rubin’s rules. *P < 0.05.

A significant improvement was observed with regard to a change in depressive symptomatology as measured by the HDRS score from baseline to week 10 (the end of treatment) in the active tDCS treatment arm: HDRS decrease of 9.41 points (s.d. = 6.25) (estimated week 10 mean = 9.58 (s.d. = 6.02)), compared to the sham tDCS treatment arm: HDRS decrease of 7.14 points (s.d. = 6.10) (estimated week 10 mean = 11.66 (s.d. = 5.96)) (95% confidence interval (CI) = 0.51–4.01, *P* = 0.012) (Fig. 2).

### Secondary outcomes

Based on the HDRS ratings, the active tDCS treatment arm was associated with a significantly greater clinical response of 58.3% compared to the sham treatment arm (37.8%; *P* = 0.017) (post hoc odds ratio (OR) = 2.31 (lower bound = 1.17, upper bound = 4.55); the active treatment arm was associated with a significantly greater remission rate of 44.9% relative to the sham treatment arm (21.8%; *P* = 0.004) (post hoc OR = 2.93, lower bound = 1.41, upper bound = 6.09).

Based on the Montgomery–Åsberg Depression Rating Scale (MADRS)^26^ ratings, the active tDCS treatment arm showed a significant improvement from baseline to week 10, with a mean improvement of 11.31 (s.d. = 8.81) (estimated mean at week 10 = 12.46 (s.d. = 9.40)) compared to sham treatment (mean improvement = 7.74; s.d. = 8.47; *P* = 0.006) (estimated mean at week 10 = 15.30 (s.d. = 9.28)). Regarding clinical response, the active treatment arm was associated with a significantly greater response rate of 64.2% compared to sham treatment (32.3%; *P* < 0.001) (post hoc OR = 3.76, lower bound = 1.83, upper bound = 7.74). Regarding clinical remission, the active treatment arm was associated with a significantly greater remission rate of 57.5% relative to sham treatment (29.4%; *P* = 0.002) (post hoc OR = 3.26, lower bound = 1.53, upper bound = 6.94).

**Extended Data Fig. 1:**
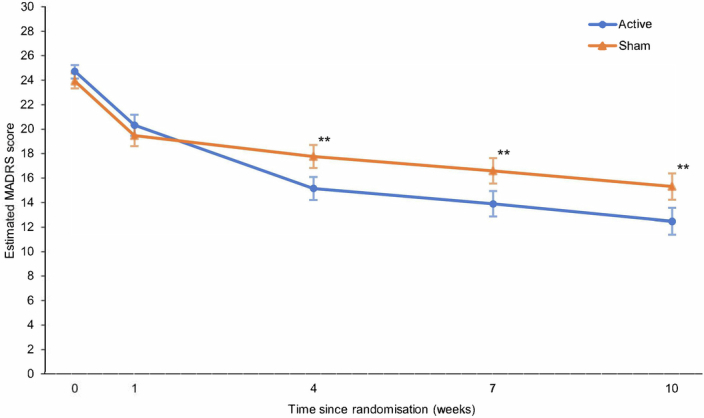
Change in Montgomery-Åsberg Depression Rating Scale (MADRS) ratings over time. Estimated mean MADRS rating scores from baseline to week 10 in the modified intention-to-treat analysis sample (n = 173) in active tDCS and sham tDCS treatment arms. Error bars represent ± 1 standard error (SE). MADRS scores range from 0 to 60 with higher values indicating more severe depressive symptoms. A significant improvement was observed in the change in MADRS ratings from baseline to week 10 in the active tDCS treatment arm, MADRS change 11.31 ± 8.81(standard deviation (SD)) (mean week 10 MADRS 12.46 ± 1.09 (SE)) as compared to sham tDCS treatment arm, MADRS change 7.74 ± 8.47 (SD) (mean week 10 MADRS 15.30 ± 1.07 (SE)) (95% CI 1.1 to 6.1, *p* = 0.006). The difference in change scores was also significant at week 4 (95% CI 1.2 to 5.5, *p* = 0.003) and week 7 (95% CI 1.1 to 5.8, *p* = 0.005) with a greater score decrease in the active treatment arm. Fully Conditional Specification (FCS) approach was used to produce 20 multiply imputed completed data sets. The FCS approach accommodates nonmonotonicity in the pattern of missing data and requires regression models to be specified for each variable with missing values needing imputation. All models included age, sex, in psychotherapy at baseline, use of any antidepressants at baseline and treatment group. The resulting completed datasets were combined using Rubin’s Rules. ** = *p* < 0.01.

**Extended Data Fig. 2:**
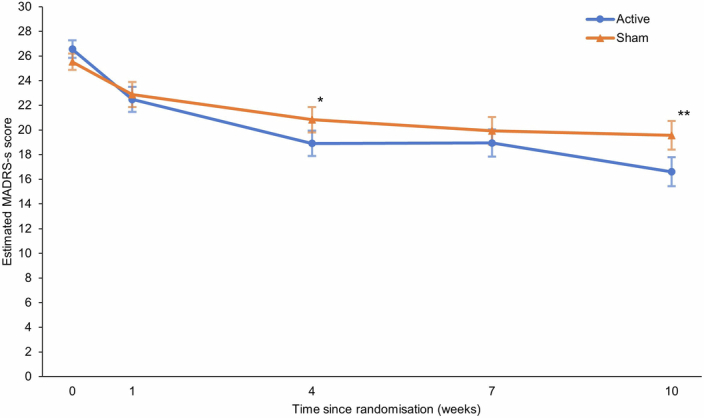
Change in Montgomery-Åsberg Depression Rating Scale-Self report (MADRS-s) ratings over time. Estimated mean MADRS-s rating scores from baseline to week 10 in the modified intention-to-treat analysis sample (n = 173) for the active tDCS and sham tDCS treatment arms. Error bars represent ± 1 standard error (SE). MADRS-s scores range from 0 to 60 with higher values indicating more severe depression. A significant improvement was observed in the change in MADRS-s ratings from baseline to week 10 in the active tDCS treatment arm, MADRS-s change 9.90 ± 8.94 (standard deviation (SD)) (mean week 10 MADRS-s 16.60 ± 1.18 (SE)) as compared to sham tDCS treatment arm, MADRS-s change 6.23 ± 9.13 (SD) (mean week 10 MADRS-s 19.55 ± 1.16 (SE)) (95% CI 0.9 to 6.4, *p* = 0.009). The difference in change scores was also significant at week 4 (95% CI 0.3 to 4.9, *p* = 0.030) with a greater score decrease in the active treatment arm. Fully Conditional Specification (FCS) approach was used to produce 20 multiply imputed completed data sets. The FCS approach accommodates nonmonotonicity in the pattern of missing data and requires regression models to be specified for each variable with missing values needing imputation. All models included age, sex, in psychotherapy at baseline, use of any antidepressants at baseline and treatment group. The resulting completed datasets were combined using Rubin’s Rules. * = *p* < 0.05, ** = *p* < 0.01.

Based on the MADRS self-report scale (MADRS-s)^27^, the active tDCS treatment arm was associated with a significant improvement from baseline to week 10, with a mean improvement of 9.90 (s.d. = 8.94) (estimated mean at week 10 = 16.60 (s.d. = 9.33)) compared to sham treatment (mean improvement = 6.23 (s.d. = 9.13), *P* = 0.009) (estimated mean at week 10 = 19.55 (s.d. = 9.62)). Regarding clinical response, the active treatment arm was associated with a significantly greater response rate of 51.8% compared to sham (25.1%; *P* = 0.002) (post hoc OR = 3.22, lower bound = 1.15, upper bound = 6.94). Regarding clinical remission, the active treatment arm was associated with a significantly greater remission rate of 53.8% compared to sham (23.4%; *P* = 0.002) (post hoc OR = 3.83, lower bound = 1.61, upper bound = 9.13) (Table 2 and Extended Data Figs. 1 and 2). There were no significant differences in quality of life between treatment arms as measured by EQ-5D-3L^28,29^ (*P* = 0.326).

**Table 2:** Primary and secondary outcomes: changes in depressive severity as measured using the HDRS, MADRS and MADRS-s, and quality of life as measured using the EQ-5D-3L after a 10-week course of active or sham tDCS

Measure	Active (*n* = 87)	Sham (*n* = 86)	Difference or OR (95% CI)	Cohen’s *d* or NNT	*P*
Primary outcome					
Decrease in HDRS score	9.41 ± 6.25	7.14 ± 6.10	2.26 (0.51 to 4.01)	0.37	0.012
Secondary outcomes					
HDRS					
Clinical response	44 (58.3%)	29 (37.8%)	2.31 (1.17 to 4.55)	5	0.017
Clinical remission	34 (44.9%)	17 (21.8%)	2.93 (1.41 to 6.09)	4	0.004
MADRS					
Decrease in score	11.31 ± 8.81	7.74 ± 8.47	3.57 (1.06 to 6.07)	0.41	0.006
Clinical response	47 (64.2%)	26 (32.3%)	3.76 (1.83 to 7.74)	4	0.0002
Clinical remission	42 (57.5%)	25 (29.4%)	3.26 (1.53 to 6.94)	4	0.002
MADRS-s					
Decrease in score	9.90 ± 8.94	6.23 ± 9.13	3.66 (0.93 to 6.40)	0.41	0.009
Clinical response	32 (51.8%)	15 (25.1%)	3.22 (1.50 to 6.94)	4	0.002
Clinical remission	32 (53.8%)	18 (23.4%)	3.83 (1.61 to 9.13)	3	0.002
EQ-5D-3L					
Change in score	0.07 ± 0.15	0.07 ± 0.17	0.02 (−0.02 to 0.06)	–	0.326

EQ-5D-3L is a quality-of-life measure^58,59^ (https://euroqol.org). Mean values are presented ‘±’ the s.d. A change in rating for the HDRS, MADRS and MADRS-s represents a decrease in total ratings from baseline to week 10. Between-group differences are shown for the changes in scores from baseline to week 10; ORs are shown for clinical response and remission outcomes. The percentages for clinical response and remission outcomes are estimated based on ORs. HDRS scores range from 0 to 52; MADRS scores range from 0 to 60; MADRS-s scores range from 0 to 54, with higher scores indicating increased depressive symptom severity. Clinical response was defined as a decrease in score (indicating reduced depressive severity) of 50% or more from baseline to week 10. Clinical remission was defined as follows: HDRS score of 7 or less; MADRS score of 10 or less; MADRS-s score of 12 or less. An FCS approach was used to produce 20 multiply imputed complete datasets. All models included age, sex, if undergoing psychotherapy at baseline, if using any antidepressant at baseline and treatment group. The resulting complete datasets were combined using Rubin’s rules. The estimated standard effect size (Cohen’s *d*) is the group difference in the estimated means divided by the pooled within-group s.d.

### Exploratory outcomes

Regarding anxiety symptoms, there were no significant differences between an active mean Hamilton Anxiety Rating Scale (HAM-A)^28^ score improvement of 6.62 (s.d. = 6.09) (mean = 8.24 (s.d. = 5.65)), compared to a sham improvement of 4.88 (s.d. = 5.88) (mean = 9.29 (s.d. = 4.90)) (*P* = 0.08). Regarding hypomanic symptoms, the Young Mania Rating Scale (YMRS)^29^ mean score was 1.27 (s.d. = 1.40) in the active treatment arm at week 10 and 1.84 (s.d. = 1.69) in the sham treatment arm; this was statistically significant (*P* = 0.03) (Supplementary Tables 12 and 13).

In the neuropsychological assessments, there were no significant differences in Rey Auditory Verbal Learning Test (RAVLT)^30^ total learning or Symbol Digit Modalities Test (SDMT)^31^ between treatment arms (Supplementary Table 14).

Per-protocol and sensitivity analyses in participants with persistent depressive symptoms who had been taking antidepressant medication at study enrollment showed significant improvements in depressive symptoms, clinical response and remission (Supplementary Tables 15 and 18).

### Analysis of study blinding and unblinding

Before unblinding at week 10 (end of trial), participants were asked to guess whether they thought they were receiving the active or sham tDCS device and their level of certainty, rating from ‘1’ for ‘very uncertain’ to ‘5’ for to ‘very certain’. A guess of active tDCS was made by 77.6% in the active treatment arm and 59.3% in the sham treatment arm; the difference was significant (*P* = 0.01). The certainty of having received active tDCS was rated highly by 57.6% (38 out of 66 guesses) in the active arm and 41.7% (20 out of 48 guesses) in the sham arm, as measured by a rating of 4 or 5, while certainty was rated low by 16.7% (11 out of 66 guesses) in the active treatment arm and 18.8% (9 out of 48 guesses) in the sham treatment arm, as measured by a rating of 1 or 2 (Supplementary Tables 36 and 39).

### Adverse events and safety

At week 10, reports of skin redness ((active = 54 (63.5%); sham = 15 (18.5%), *P* < 0.001), skin irritation ((active = 6 (6.9%); sham = 0 (0%), *P* = 0.03) and trouble concentrating ((active = 12 (14.1%); sham = 3 (3.7%), *P* = 0.03) were greater in the active treatment arm relative to the sham treatment arm. There were no differences in headache, neck pain, scalp pain, itching, burning sensation, sleepiness or acute mood changes between treatment arms. Two participants in the active group described developing ‘burns’ at the left anode site. When reviewed, they might have been caused by using sponges that had dried out. Neither developed into residual skin lesions or scarring. Participants had not contacted the 24-h contact number; both had informed the research team at their following study visit, which was 1–2 weeks afterward. There were no visible lesions at the study visits. One participant had taken a break from the sessions for 4 days and the burn had fully healed. The second participant was experiencing dry skin at the electrode site and was advised that they could take a break from the sessions until the skin had healed; however, they did not take a break until after the next study visit, 3 weeks later, when they were advised to take a break from the sessions to allow the dry skin and tenderness to heal. The participant then missed the next three stimulations; the skin was no longer tender but it was still dry at the week 10 end-of-study visit. There were no serious adverse events related to the device; no participants developed mania or hypomania (Tables 3 and 4 and Supplementary Tables 24–29).

**Table 3:** Unanticipated adverse events at 10 weeks

Event category	Active tDCS (*n* = 87)	Sham tDCS (*n* = 86)	Difference (95% CI)	*P*
Ear and labyrinth disorders	2 (2.3)	2 (2.3)	0 (−6.2 to 6.0)	0.99
Eye disorders	3 (3.4)	1 (1.2)	2.3 (−3.3 to 8.9)	0.62
Gastrointestinal disorders	2 (2.3)	1 (1.2)	1.1 (−4.5 to 7.0)	0.99
General disorders and administration site conditions	3 (3.4)	2 (2.3)	1.1 (−5.2 to 8.0)	0.99
Infections and infestations	1 (1.1)	1 (1.2)	0 (−5.5 to 5.3)	0.99
Injury, poisoning and procedural complications	2 (2.3)	0 (0)	2.3 (−2.2 to 8.1)	0.49
Nervous system disorders	7 (8.0)	8 (9.3)	−1.3 (−10.4 to 8.0)	0.79
Psychiatric disorders	4 (4.6)	4 (4.7)	−0.1 (−7.5 to 7.3)	0.99
Skin and subcutaneous tissue disorders	17 (19.5)	7 (8.1)	11.4 (1.0 to 22.3)	0.05
Vascular disorders	1 (1.1)	0 (0)	1.1 (−3.3 to 6.4)	0.99
Number of participants with adverse events at week 10				
≥1 Mild adverse event	21 (24.1)	14 (16.3)	7.9 (−4.5 to 20.3)	0.25
≥1 Moderate adverse event	13 (14.9)	18 (9.3)	5.6 (−4.5 to 16.1)	0.35
≥1 Severe adverse event	3 (3.4)	1 (1.2)	2.3 (−3.3 to 8.9)	0.62
Serious adverse events during the trial				
Hospitalization for hypertension	1 (1.1)	0 (0)	1.1 (–3.3 to 6.4)	0.99
Death	0	0	–	–
New-onset mania or hypomania	0	0	–	–

The adverse event categories are displayed as the number of participants with the percentage in parentheses. The difference between groups is displayed as a percentage. *P* values, determined using a two-sided Fisher’s exact test, represent the between-group difference. An adverse event was deemed present if the participant rated that it was at least possibly associated with the intervention. Participants rated the severity of the adverse events as mild, moderate or severe; the adverse events were assessed by the investigator. Analyses were completed for participants who completed at least one tDCS session. The serious adverse event was not related to the intervention.

**Table 4:** Anticipated adverse events at 10 weeks as measured using the tDCS Adverse Events Questionnaire^39^

	Active (*n* = 87)				Sham (*n* = 86)				
Adverse event category	Total	Mild	Moderate	Severe	Total	Mild	Moderate	Severe	*P*
Headache	36 (42.4)	24 (28.2)	11 (12.9)	1 (1.2)	29 (35.8)	18 (22.2)	9 (11.1)	2 (2.5)	0.43
Neck pain	2 (2.4)	0 (0)	2 (2.4)	0 (0)	4 (4.9)	1 (1.2)	3 (3.7)	0 (0)	0.44
Scalp pain	18 (21.2)	14 (16.5)	3 (3.5)	1 (1.2)	10 (12.3)	7 (8.6)	3 (3.7)	0 (0)	0.15
Itching	43 (50.6)	37 (43.5)	3 (3.5)	3 (3.5)	35 (43.2)	28 (34.6)	7 (8.6)	0 (0)	0.08
Burning sensation	37 (43.5)	32 (37.6)	4 (4.7)	1 (1.2)	31 (38.3)	25 (30.9)	6 (7.4)	0 (0)	0.43
Skin redness	54 (63.5)	42 (49.4)	11 (12.9)	1 (1.2)	15 (18.5)	13 (16.0)	2 (2.5%)	0 (0)	<0.001*
Sleepiness	10 (11.8)	5 (5.9)	4 (4.7)	1 (1.2)	12 (14.8)	9 (11.1)	2 (2.5)	1 (1.1)	0.65
Trouble concentrating	12 (14.1)	8 (9.4)	3 (3.5)	1 (1.2)	3 (3.7)	2 (2.5)	1 (1.2)	0 (0)	0.03
Acute mood change	7 (8.2)	3 (3.5)	3 (3.5)	1 (1.2)	6 (7.4)	5 (6.2)	1 (1.2)	0 (0)	1.00

Data are *n* (%). An adverse event was present if the participant rated that it was at least remotely possible that it was associated with the intervention. Participants rated the severity of the adverse events as mild, moderate or severe. *P* values, determined with a two-sided Fisher’s exact test, represent the group differences of the total number of events per event category. *Exact *P* value for skin redness (0.000000003).

## Discussion

In this international, multisite, sham-controlled, RCT of home-based tDCS treatment for MDD, a 10-week course of active stimulation was associated with significantly greater improvements in depressive symptoms, clinical response and remission rates compared to sham stimulation. Improvements were evident in both clinician-rated depressive symptom ratings (HDRS and MADRS) and in self-reported ratings (MADRS-s). The clinical significance of the outcomes is highlighted by high rates of treatment response and remission that were 2–3 times greater in the active treatment arm compared to the sham treatment arm. Clinical efficacy was demonstrated in a wide range of forms of MDD, from first-episode MDD to individuals having a history of recurrent episodes and participants with treatment-resistant depression.

Meta-analyses of clinic-based tDCS sessions reported that active tDCS is associated with greater improvements in depressive symptoms, clinical response and clinical remission rates compared to sham tDCS, particularly in first-episode and recurrent MDD^6,32–34^. However, in a recent large trial, Burkhardt et al.^35^ did not observe any significant effects of adjunct tDCS treatment to antidepressant medication in a 6-week trial. In the present trial, we had a comparable inclusion criteria for treatment-resistant depression but a longer 10-week treatment duration. Burkhardt et al.^35^ included participants with MDD with persistent depressive symptoms of at least moderate severity while taking a selective serotonin reuptake inhibitor for a minimum of 4 weeks. Similarly, our inclusion criteria were participants with MDD with persistent depressive symptoms of at least moderate severity while taking antidepressant medication for a minimum of 6 weeks at the point of screening. Our inclusion criteria met the UK National Institute for Health and Care Excellence definition of treatment-resistant depression^24,25^; 63% of our sample fulfilled these criteria. However, treatment-resistant depression is negatively correlated with clinical efficacy to tDCS treatment^6,32–34^. This is a clinical definition that can be further delineated by the number and types of failed treatment trials. Our exclusion criteria included having a history of poor treatment response to two or more antidepressant medications, which reflects increased severity of treatment-resistant depression. About 12–17% of participants in the study by Burkhardt et al.^35^ had such a history of treatment failure, which could have affected their observed lack of clinical effects. The level of depressive symptom severity and mean ages were comparable in Burkhardt et al.^35^ and in the present trial, while age at onset was younger in the present trial by about 10 years and we did not have an upper age limit.

Furthermore, the clinical effects of tDCS continue to increase for up to 10 weeks^20^. In the present trial, we found strong clinical efficacy and safety with our 10-week home-based protocol. This is in contrast with recent home-based tDCS trials; all had 6-week treatment durations and two trials had small sample sizes (*n* = 11 (ref. ^18^) and *n* = 58 (ref. ^19^))^17–19^. A single-blind RCT of tDCS augmentation to antidepressant medication, consisting of hybrid clinic-based and home-based tDCS sessions, reported significant improvements in depressive symptoms in the active group as measured by self-reported symptoms rating but not in clinician-based ratings^19^. In a large RCT (*n* = 210), no significant effects were observed between three treatment arms: active tDCS, active tDCS combined with a digital psychological intervention (double-active); and sham tDCS combined with internet browsing (double-sham). In the present trial, clinical treatment effects were evident at 10 weeks. Longer treatment durations may be necessary to observe clinical efficacy^36^; in their meta-analysis, Nikolin et al.^20^ reported that effect sizes continued to increase with longer treatment durations.

We found a high level of safety in the present trial. Safety was monitored using real-time assessments by videoconferencing and a dedicated contact number with 24-h access to researchers. A recent trial ended early because of adverse events involving skin lesions, which were the result of an accumulation of electrical burns in five participants in the active tDCS group from a total enrollment of 11 participants with MDD^18^. Electrical burns can be an unanticipated side effect; they are usually caused by the application of tap water to moisten sponges^37^, insufficient moistening with conductive saline solution^38^ or preexisting skin lesions. In the present trial, we had two incidents of reported electrical burns; both participants reported these during the study visit. Both were probably caused by insufficient sponge moistening; neither instance of electrical burn developed into residual skin lesions or scarring, and participants were keen to continue the tDCS sessions after a brief break. There were no serious adverse events related to the device and no incidents of serious suicide risk. However, active stimulation was associated with higher rates of skin redness, irritation and dry skin relative to sham treatment^39,40^.

During the tDCS sessions, participants were asked to sit or lie down and to avoid engaging in activities that might compromise safety or device functionality. Their activities had not been recorded by the research team. State-dependent effects of tDCS stimulation are possible; an interaction between external stimulation, location and internal state of the region or network has been observed^41,42^. The type of task activity during stimulation can influence cognitive enhancement in healthy participants^43^ and treatment response in clinical samples^44^. Concurrent administration of active tDCS and cognitive control training (CCT) has been associated with sustained improvements in depressive symptoms compared to active tDCS plus sham CCT or sham tDCS plus CCT^44^. However, a 6-week trial of cognitive behavioral therapy (CBT) with three treatment arms—CBT alone, CBT plus active tDCS and CBT plus sham tDCS—in a sample of 126 participants with MDD, reported no significant effects between groups^45^.

Blinding is key in RCTs to mitigate potential biases that can impact on the outcome. Procedures involve the establishment and maintenance of blinding, measures to prevent unblinding and assessment of successful blinding^46,47^. To establish blinding in the present trial, all participants and researchers were blinded to treatment arm allocation; the placebo-sham control intervention was identical in appearance to the active intervention. Furthermore, when using the sham device, there was brief stimulation at the start and at the end of each session to mimic active tDCS sensations to aid in blinding and to balance potential nocebo effects across groups^48^. To maintain blinding, the treatment protocol and study visits were identical in both treatment arms. All participants maintained their ongoing treatments throughout the trial, and all participants used the active tDCS device in the subsequent open-label phase of the trial to incorporate real-life clinical care while balancing expectations between groups and to limit attrition^47^. The tDCS treatment arms were described as ‘active’ or ‘inactive’ stimulation by researchers during the trial to maintain comparable phrasing and reduce potential negative connotations associated with the words ‘placebo’ or ‘sham’. Outcome assessors were blinded to group allocation as a second independent researcher was present for the clinical ratings^47^. Ethicality was assessed a priori and worsening of symptoms was included as a withdrawal criterion. An automatic email report was sent to all research team members when unblinding occurred as notification and to prevent potential concealment of any accidental unblinding. Timing of the blinded assessment questionnaire at the end of the blinded treatment phase, rather than at time points throughout the trial, reduced the influence of potential interjections.

In the blinding assessment, participants were asked to guess if they had been receiving the ‘active’ or ‘sham’ treatment and the certainty of their guess, ranging from ‘very uncertain’ to ‘very certain’ on a five-point scale. Participants who felt ‘very uncertain’ of their guess are comparable to participants guessing ‘don’t know’. More participants in the active treatment arm guessed that they were receiving active tDCS (77.6%) compared to participants in the sham treatment arm (59.3%). However, a moderate proportion were ‘very uncertain’ about their guess in the active (16.7%) and sham (18.8%) treatment arms; endorsement of being ‘very certain’ in the active (57.6%) and sham (41.7%) treatment arms was limited, with no significant differences between treatment arms. It is possible that participants who believed that they were in the active treatment arm were more likely to show a placebo response. However, in their meta-analysis of antidepressant medication RCTs, Lin et al.^49^ found no association between blinding effects and treatment effect sizes. The 2010 Consolidated Standards of Reporting Trials (CONSORT) guidance recommends specifying how blinding is established but no longer recommends reporting on how the success of blinding is assessed because healthcare providers and participants are likely to know if the primary outcome has been achieved by participants, making interpretation more difficult because responses might reflect accurate assumptions about the efficacy of the intervention rather than a failure of blinding^50^. Moreover, significant clinical efficacy was maintained for active relative to sham treatment in participants who had made a guess of ‘active’ treatment; the placebo response rate in the sham treatment arm in the present trial (26.9%) was lower than placebo response rates observed in a sham group (36%)^19^ and double-sham group (38%)^17^ that had included in-person study visits at the clinical research center^17,19^ and weekly online visits for 6 weeks^19^.

Limitations of the present trial include the lack of a ‘don’t know’ option in the blinding assessment. Well-executed blinding to treatment allocation should lead participants to be uncertain of which treatment they are receiving. By including a ‘don’t know’ option, it should be possible to calculate a proposed index of blinding^51^. Differences in head sizes, individual anatomical features and the positioning of devices among users may lead to unique configurations of electric field density within the brain^52,53^. Interindividual variations in tDCS can be partially explained due to differences in electric fields^54^. The tDCS device used in the present study has undergone electric field modeling, indicating that the device targets areas within the prefrontal cortex linked to MDD pathophysiology^52^. While participants were taught how to use the device and positioning had been observed in real time, variations in positioning could potentially affect electric field intensity and in turn treatment outcomes^52^. All clinical rating scale assessments were performed using videoconferencing, although no significant differences were found between face-to-face and videoconferencing HDRS ratings conducted within the same day^55^; we sought to have a second team member to perform clinical ratings to maintain blinding and ensure validity. Video consultation for clinical assessment and mental health treatment has become more common in recent years and is as effective as face-to-face visits for improving clinical outcomes and providing patients with more flexibility^56,57^. Regarding quality of life, there was no significant difference between groups in a self-report measure. The scores on the quality-of-life measure were relatively high at baseline and both treatment arms reported some improvement in quality of life that was not statistically significant. MDD is more common in women and the present study consisted of a larger proportion of female participants as expected. All participants self-reported their sex. An effect of sex or gender on clinical efficacy was not expected, although this warrants further investigation. Ethnic diversity in the present sample was limited and a history of hospital admissions was an exclusion criterion that may limit the generalizability of the findings.

In summary, a 10-week course of home-based active tDCS was associated with greater improvements in depressive symptoms, clinical response and remission in participants with MDD with at least a moderate severity of depressive symptoms compared to sham tDCS. Efficacy was observed in participants who were taking antidepressant medication indicative of treatment-resistant depression or undergoing psychotherapy, as well as participants who were treatment-free. All participants had real-time remote supervision visits. High acceptability and safety were observed in the present trial. Home-based tDCS could be a potential first-line treatment for MDD as it demonstrates efficacy, acceptability and safety; however, ongoing safety monitoring is required.

## Methods

### Ethics and study design

The study was a multisite, double-blind, placebo-controlled, randomized, superiority controlled trial of 10-week home-based tDCS treatment for MDD followed by a 10-week open-label treatment. Participants were recruited from throughout England and Wales (UK) and Texas (USA). Recruitment sites were at the University of East London in London, UK and at the University of Texas Health Science Center in Houston, Texas, USA, respectively.

All participants provided written informed consent. Ethical approval was provided by the South Central-Hampshire B Research Ethics Committee (ref. 22/SC/0023) and the WIRB-Copernicus Group International Review Board (ref. 1324775). ClinicalTrials.gov registration: NCT05202119. Research execution included local research assistants who are included as coauthors. The study protocol is available in the Supplementary Information.

### Participants

Participants were adults with MDD aged 18 years or older, in a current depressive episode as determined by the DSM-5 (ref. ^21^) criteria and assessed in a structed clinical interview (Mini-International Neuropsychiatric Interview (MINI) v.7.0.2 (ref. ^22^)). Inclusion criteria included: having at least moderate severity of depressive symptoms, as measured by score of 16 or greater on the 17-item HDRS^23^; being treatment-free or taking stable antidepressant medication or undergoing psychotherapy for at least 6 weeks before enrollment and being agreeable to maintaining the same treatment throughout the trial; being under care of general practitioner or psychiatrist. Exclusion criteria included: having treatment-resistant depression, defined as inadequate clinical response to two or more trials of antidepressant medication at an adequate dose and duration; high suicide risk based on the Columbia Suicide Severity Rating Scale (C-SSRS) Triage and Risk Identification Screener^60^; having a comorbid psychiatric disorder; taking medications that affect cortical excitability (for example, benzodiazepines, epilepsy medication); and contraindications to tDCS. Sex was determined by participant self-report; there was no exclusion of males or females and no upper limit on how many participants of each sex or gender could enroll^61^. The full inclusion and exclusion criteria are presented in the Supplementary Notes—Inclusion and exclusion criteria.

### Procedures

Participants were recruited through the Flow Neuroscience website, email lists and social media posts. Individuals completed an online pre-screening form, hosted by a contract research organization, followed by a telephone call with a contract research organization member. Individuals then provided written informed consent and had an assessment with a research team member using Microsoft Teams videoconferencing. All participants were registered with a primary care physician as an inclusion criterion (Supplementary Notes—Inclusion and exclusion criteria; Supplementary Table 1). Research team members completed training in clinical trial ethics and procedures, namely good clinical practice, MINI interview schedule, C-SSRS and clinical rating scales. The site principal investigators were consultant psychiatrists and reviewed the eligibility of each participant and clinical assessments. Participants were compensated £30 or US$60 for each study visit during the blinded phase of the trial. Participants enrolled in the UK were able to keep the tDCS device after trial completion.

### Randomization

Participants were randomly assigned to either sham or active tDCS treatment at a 1:1 ratio, which was performed independently in UK and USA. Block randomization, which is a form of stratified random sampling, was used with permuted block sizes of four and six. This was conducted by the sponsor, Flow Neuroscience, and stored in a dedicated database, which was not accessible to research team members.

### Intervention

Active and sham tDCS was administered using the Flow FL-100 device. The device was a headset placed over the forehead with two prepositioned conductive rubber electrodes, each 23 cm^2^. Electrodes were fixed with approximate placement of the anode over F3 (left DLPFC) and the cathode over F4 (right DLPFC) based on international 10–20 electroencephalography system^52^.

Active stimulation consisted of 2 mA direct current stimulation for 30 min with gradual ramp up over 120 s at the start and ramp down over 15 s at end of the session. Sham stimulation with the same device and app was used to resemble the active intervention and to receive the treatment schedule. An initial ramp up from 0 to 1 mA over 30 s then ramp down to 0 mA over 15 s was repeated at the end of the session to cause a tingling sensation that mimics active stimulation.

The 10-week RCT consisted of five tDCS sessions per week for 3 weeks followed by three tDCS sessions per week for 7 weeks. The tDCS parameters were based on meta-analyses, which demonstrated that treatment effects are most evident for a 30-min stimulus duration for at least 20 sessions (2-mA current) in MDD^32–34^.

At week 10, participants and researchers were informed of treatment arm allocation. The 10-week open-label phase consisted of active tDCS sessions for all participants. Participants who received active tDCS treatment were offered three sessions per week for 10 weeks; participants in the sham treatment arm were offered the active tDCS stimulation schedule, that is, five sessions per week for three weeks then three sessions per week for 7 weeks.

tDCS stimulation was provided using a study-specific installation of the app that connected to the headset via Bluetooth. Researchers had access to remote monitoring, with real-time data use to monitor compliance. Researchers received training to use the headset and were present by videoconferencing for the initial session to support participants who were at home, with app-guided training to demonstrate electrode placement, consisting of video and augmented reality via the device camera. All remaining tDCS sessions were completed by the participants at home, without the presence of a researcher. Participants were asked to have video and microphone on during the initial session. Participants were advised to sit or lie down during use, not to use the headset outdoors, close to water, while driving, during any activity that could lead to a risk of injury, while intoxicated or incapacitated, or in environments with strong magnetic fields.

### Blinding

Participants and research team members were blinded to group allocation. We sought to have the same research team member present for the same participant at each study visit. A second research team member joined the clinical reviews for independent rating and would not be present while adverse events or stimulation was discussed to prevent any potential bias. Ratings were cross-checked and reviewed by the site principal investigators.

At week 10, after completion of all assessments and before unblinding, participants were asked whether they thought they had been using the ‘active’ or ‘sham’ tDCS device and how certain they were, as measured by a rating on a scale from 1 (‘very uncertain’) to 5 (‘very certain’). Once this had been completed, the research team member accessed the online remote monitoring system to unblind allocation and informed the participant of group allocation. At the point of unblinding, an automatic email notification was sent to the principal investigator and research team members that unblinding had occurred.

### Outcomes

The primary outcome was the adjusted mean group difference in depressive symptom severity between active and sham treatment arms as measured using the 17-item HDRS^23^ at week 10 (end of treatment) compared to baseline.

Depressive symptom severity was measured by clinician-rated scales, the HDRS and MADRS^26^, and self-report scale, the MADRS-s^27^, suicide ideation and attempts using the C-SSRS^60^, and manic symptoms using the YMRS^29^ at baseline and at weeks 1, 4, 7, 10 and 20. Anxiety symptoms were measured using the (HAM-A)^30^ and quality of life was measured using the EQ-5D-3L^58,59^, consisting of five dimensions (mobility, self-care, usual activities, pain and discomfort) at baseline and at weeks 10 and 20.

Secondary outcomes were the adjusted mean group difference in depressive symptom severity between active and sham treatment arms as measured using the MADRS and MADRS-s at week 10 compared to baseline; clinical response defined as a minimum of 50% reduction from baseline in HDRS, MADRS and MADRS-s at week 10; clinical remission defined as an HDRS score of 7 or less, MADRS score of 10 or less and MADRS-s score of 12 or less; and quality of life as measured by the EQ-5D-3L at week 10.

Exploratory outcomes included correlation between adherence to stimulation and HDRS, MADRS decrease in active treatment arm at week 10; changes in anxiety symptoms from baseline to week 10; and presence of hypomanic and manic symptoms at week 10.

Exploratory outcomes in neuropsychological functioning were assessed using the RAVLT^30^ total learning score for memory and verbal learning, and the SDMT^31^ for psychomotor speed and visuospatial attention, assessed at baseline, and then at weeks 10 and 20. Order and versions were counterbalanced. The written SDMT was chosen to reduce the chance of task interference resulting from a poor internet signal. SDMT was mailed to participants, completed using pen and paper during the session, and recorded using a screenshot.

Treatment acceptability was assessed using our treatment acceptability questionnaire^13^ at baseline, and then at weeks 10 and 20. The full description of the exploratory outcomes is presented in Supplementary Tables 16, 19, 21, 23–35, 37, 38 and 46–53 and Supplementary Figs. 1–6 and 10–12.

### Safety

Adverse events were assessed at each visit; participants were able to contact the research team using a dedicated contact number at any time. The tDCS Adverse Events Questionnaire^39^ was administered at weeks 10 and 20.

### Sample size

Sample size calculation was based on Brunoni et al.^36^, with a two-sample *t*-test for the mean difference, with 80% power and one-sided type 1 error (0.025), resulting in a sample size of 176 participants with MDD. To increase power to 87.6%, sample size was increased to 216. Assuming a 20% attrition rate, the total sample size was 270 participants. A prespecified interim analysis was performed when 90 participants with MDD completed week 10, which included both futility assessment and sample size reestimation^62^. The interim analysis was used to modify the trial in two ways for the primary end point, to declare the trial futile and stop enrollment or to specify the number of participants between 100 and 270 to power the trial based on promising zone methodology^63,64^.

### Statistical analysis

The ITT analysis included all randomized participants classified according to the intended treatment. Participants excluded before randomization were considered screen failures. The modified ITT analysis set included ITT participants who received at least one tDCS session (active or sham) and excluded participants randomized in error. The per-protocol analysis set consisted of participants in the modified ITT analysis set, participants with a device failure within the 10-week randomized trial and participants with deviation from the clinical investigation plan caused by the investigational device or by problems regarding tolerability. It excluded participants who took a new medication or treatment during the trial (listed as exclusion criteria), participants who did not meet the inclusion criteria or fulfilled the exclusion criteria, participants who had performed fewer than ten sessions during the first 3 weeks and participants with major protocol violations that would be expected to confound clinical assessment (Supplementary Information—Statistical Analysis Plan, Section 2).

The primary effectiveness outcome was the estimated mean group difference in HDRS scores in participants randomized to active and sham treatments using a mixed model for repeated measures (MMRM). The model included the HDRS baseline value, antidepressant medication status, psychotherapy treatment, age and sex. Missing data were categorized according to the reason for missingness (missing at random or not) and differentially imputed based on that classification. If *P* values were less than a one-sided *P* = 0.025, then the end point would be declared positive (Supplementary Information—Statistical Analysis Plan, Sections 3.1–3.1.4, 4 and 5).

MMRM allows for the inclusion of data from all time points in the model and not only baseline and week 10 end-of-treatment values; it allows for the inclusion of participants with missing week 10 values. The MMRM approach is a direct likelihood approach. The MMRM parameters were estimated using SAS PROC MIXED (SAS Institute) v.9.4 or higher. In a matrix equation, the MMRM can be expressed as *Y*_*i*_ = *X*_*i*_**β** + *Z*_*i*_*u* + **e**_*i*_, where **β** is the vector of the fixed-effect regression parameters (for the overall mean change, the treatment effect *θ*, a vector of post-baseline time effects **τ**, a vector of treatment-by-time interaction effects **η** and a vector of covariate effects **φ** that includes baseline HDRS, and, optionally, other covariates selected a priori). *X* is a design matrix for the fixed effects and *Z* is a design matrix used to account for other random effects *u*, if any are included. Key assumptions are about **e**, the random error vector. The expected value is zero, that is, **E**(**e**) = 0. An unstructured covariance is assumed, requiring estimation of variances at each visit and all pairwise covariances, that is, Var(**e**) = σe2Vunstructured (ref. ^65^).

If the primary end point is met, the secondary end points can be tested based on a hierarchical approach. As specified in the protocol, the Hochberg^66,67^ approach was used to control multiplicity (Supplementary Table 11). The Hochberg correction rank-orders the end points based on the size of the *P* value, ranking them from largest to smallest, and compares those values to a sequentially decreasing alpha level to determine whether the null hypothesis should be rejected. Secondary outcomes were HDRS clinical response and remission, EQ-5D-3L change and change in ratings, response and remission in MADRS and MADRS-s (Supplementary Information—Statistical Analysis Plan, Sections 3.1.5–3.1.9).

Exploratory end points were analyzed through summary statistics as the mean and s.d. or percentages and ORs. The two groups were compared using a Student’s *t*-test or Fisher’s exact test as appropriate. Spearman correlation was used to assess the association between two continuous variables; 95% CIs were presented. The percentages of participants who correctly guessed the arm that they were in were compared using a Fisher’s exact test. Subgroup analyses of primary and secondary end points were conducted through stratification according to antidepressant use at baseline and site (Supplementary Information—Statistical Analysis Plan, Sections 3.1.10 and 8).

Standard deviations are provided based on Cochran’s^68^ conversion of s.e. to s.d. weighted by sample size. Type 1 errors were controlled by only testing the three named secondary end points after meeting the primary end point; nominal *P* values are provided for all other evaluations.

Full description of the statistical analyses and handling of missing data can be found in Supplementary Information.

### Reporting summary

Further information on research design is available in the Nature Portfolio Reporting Summary linked to this article.

## Online content

Any methods, additional references, Nature Portfolio reporting summaries, source data, extended data, supplementary information, acknowledgements, peer review information; details of author contributions and competing interests; and statements of data and code availability are available at 10.1038/s41591-024-03305-y.

## Supplementary information

Supplementary Information Supplementary Notes, Tables 1–53, Figs. 1–13, Statistical Analysis Plan and Study Protocol.

Reporting Summary

## Data Availability

The deidentified individual participant data and the data dictionary that support the findings of this study are available from the academic researchers or the sponsor beginning 6 months after publication because of legal reasons. However, restrictions apply to the availability of these data; thus, they are not publicly available. The Statistical Analysis Plan is available in the Supplementary Information. A data request and brief analysis plan will be required in accordance with the ethics committee requirements. These will be reviewed by the lead, study steering committee and study sponsor. A data transfer agreement will have to be completed before any data being shared. After completion of the data transfer agreement, data will be shared as password-protected files. Data sharing will abide by the rules and policies defined by the sponsor, relevant institutional review boards, as well as local, state and federal laws and regulations. Rights and privacy of individuals participating in the research will be protected at all times. Approval will not be provided for commercial use of the data. Requests can be made to C.H.Y.F. (cynthia.fu@kcl.ac.uk).

## Code availability

The analysis code for the longitudinal model is provided in the Supplementary Information. The full code used for the data analysis will be available from the sponsor beginning 6 months after publication of the trial results.
