# Supplementary material for: Home-based transcranial direct current stimulation treatment for major depressive disorder: a fully remote phase 2 randomized sham-controlled trial
Source: Nat Med. 2024 Oct 21;31(1):87–95. doi: 10.1038/s41591-024-03305-y (PMC11750699; doi:10.1038/s41591-024-03305-y)
Supplement: Supplementary file 1 — Supplementary Notes, Tables 1–53, Figs. 1–13, Statistical Analysis Plan and Study Protocol. [file 41591_2024_3305_MOESM1_ESM.pdf]

nature medicine

Supplementary information https://doi.org/10.1038/s41591-024-03305-y

Home-based transcranial direct current stimulation treatment for major depressive disorder: a fully remote phase 2 randomized sham-controlled trial

In the format provided by the authors and unedited

Supplementary Materials

Home-based transcranial direct current stimulation treatment for major depressive disorder: a fully remote phase 2 randomized sham-controlled trial

Table of Contents

Supplementary Notes....5
Inclusion and exclusion criteria....5
Participant recruitment....7
Major depressive disorder diagnosis and severity....8
Safety and tolerability....8
Interim analysis....9
Statistical methods....9
Site heterogeneity....10
Per-Protocol Analysis....10
Acceptability Analysis....10
Open label transition analysis....11
Analysis Code for Longitudinal Model....12
Supplementary Table 1. Recruitment figures and success rates for sites at each time point....13
Supplementary Table 2. Demographic and clinical characteristics of patients at baseline (complementary information)....14
Supplementary Table 3. Concomitant medications ongoing at randomisation and medical history....15
Supplementary Table 4. Psychotropic combination medications ongoing at randomisation.....16
Supplementary Table 5. Length of time in psychotherapy at baseline for participants in the UK sample....17
Supplementary Table 6. Reasons for study withdrawal in the active and sham tDCS groups during the blinded phase of the trial....18
Supplementary Table 7. withdrawal reasons disaggregated by sex during the 10-week blinded phase of the trial....18
Supplementary Table 8. Hamilton Depression Rating Scale estimated scores at each timepoint from baseline to week 10....19
Supplementary Table 9. Montgomery-Åsberg Depression Rating Scale estimated scores at each timepoint from baseline to week 10....19
Supplementary Table 10. Montgomery-Åsberg Depression Rating Scale-self report estimated scores at each timepoint from baseline to week 10....19
Supplementary Table 11. Multiplicity Adjustment....20
Supplementary Table 12. Hamilton Anxiety Rating Scale scores from baseline to week 10....21
Supplementary Table 13. Young Mania Rating Scale score from baseline to week 10....21
Supplementary Table 14. Neuropsychological measure: verbal learning (Rey Auditory Verbal Learning Test (RAVLT) and information processing speed (Symbol-Digit Modalities Test (SDMT) change scores from baseline to week 10....22

Supplementary Materials

Home-based transcranial direct current stimulation treatment for major depressive disorder: a fully remote phase 2 randomized sham-controlled trial

Supplementary Figure 1. Rey Auditory Verbal Learning Test (RAVLT)[19] scores from baseline to week 10 23

Supplementary Figure 2. Symbol-Digit Modalities Test (SDMT)[18] scores from baseline to week 10 23

Supplementary Table 15. Clinical outcomes for participants who were taking antidepressant medication at week 10 end of treatment 24

Supplementary Table 16. Clinical outcomes for antidepressant medication free participants 25

Supplementary Table 17. Per protocol analysis reasons for exclusion 26

Supplementary Table 18. Per protocol analysis of changes in depressive severity as measured by HDRS, MADRS and MADRS-s and quality of life as measured by EQ-5D-3L following 10-week course of active or sham tDCS 27

Supplementary Table 19. Changes in depressive severity as measured by HDRS, MADRS and MADRS-s and quality of life as measured by EQ-5D-3L at 7 weeks 28

Supplementary Table 20. Changes in depressive severity as measured by HDRS, disaggregated by sex at week 10 29

Supplementary Figure 3. Correlation between change from baseline to 10 weeks in MADRS and MADRS-s 30

Supplementary Figure 4. Correlation between change from baseline to 10 weeks in HDRS and percent of sessions with stimulation in the active group 31

Supplementary Figure 5. Correlation between change from baseline to 10 weeks in MADRS and percent of sessions with stimulation in the active group 32

Supplementary Table 21. Treatment acceptability questionnaire (TAQ) and responses at baseline (n = 174 (active = 87, sham = 87)), at the end of treatment (n = 151 (active = 75, sham = 76)). Italics represent post-treatment phrasing 33

Supplementary Figure 6. Percentage of participants in active and sham groups who endorsed each response in the acceptability questionnaire at baseline and week 10 34

Supplementary Figure 7. Change in individual depressive severity ratings over time. 35

Supplementary Figure 8. Percentage of participants who completed stimulations at each week in the mITT sample (n=173). 36

Supplementary Figure 9. Percentage of participants who completed stimulations at each week for participants who completed the 10 week blinded phase (n=149). 36

Supplementary Table 22. Number of stimulations completed in the active and sham groups at week 10 37

Supplementary Table 23. Number of participants who showed a 'clinically meaningful improvement' at each time point (indicated by a HDRS scores of -3 from baseline) 38

Supplementary Figure 10. Cumulative proportion of responders analysis for HDRS scores 39

Supplementary Figure 11. Cumulative proportion of responders analysis for MADRS scores 40

Supplementary Figure 12. Cumulative proportion of responders analysis for MADRS-s scores 41

Supplementary Table 24. All unanticipated device related adverse events 42

Supplementary Materials

Home-based transcranial direct current stimulation treatment for major depressive disorder: a fully remote phase 2 randomized sham-controlled trial

Supplementary Table 25. All unanticipated device related adverse events rated as mild 43

Supplementary Table 26. All unanticipated device related adverse events rated as moderate 44

Supplementary Table 27. All unanticipated device related adverse events rated as severe 45

Supplementary Table 28. All unanticipated device related adverse events by days post randomisation 46

Supplementary Table 29. All unanticipated adverse events 47

Supplementary Table 30. Response and remission rates at all time points 50

Supplementary Table 31. Changes in depressive severity as measured by HDRS, MADRS and MADRS-s and quality of life as measured by EQ-5D-3L for ITT analysis 51

Supplementary Table 32. Changes in depressive severity as measured by Hamilton Depression Rating Scale using last observation carried forward for missing data, mITT analysis 52

Supplementary Table 33. Response rates as measured by HDRS, MADRS and MADRS-s using response definition as a reduction in score at week 10 of >50% from baseline 53

Supplementary Table 34. Changes in depressive severity as measured by HDRS, MADRS and MADRS-s and quality of life as measured by EQ-5D-3L for participants at the UK site at 10 weeks 54

Supplementary Table 35. Changes in depressive severity as measured by HDRS, MADRS and MADRS-s and quality of life as measured by EQ-5D-3L for participants at the US site at 10 weeks 55

Supplementary Table 36. Changes in depressive severity as measured by HDRS, MADRS and MADRS-s and quality of life as measured by EQ-5D-3L for participants who made the 'guess' that they were receiving the active treatment at 10 weeks 56

Supplementary Table 37. Changes in depressive severity as measured by HDRS, MADRS and MADRS-s and quality of life as measured by EQ-5D-3L for participants who correctly guessed their treatment allocation group 57

Supplementary Table 38. Changes in depressive severity as measured by HDRS, MADRS and MADRS-s and quality of life as measured by EQ-5D-3L for participants who incorrectly guessed their treatment allocation group 58

Supplementary Table 39. Blinding evaluation and guess certainty mITT sample. 59

Supplementary Table 40. Completers analysis (N=149) of changes in depressive severity as measured by HDRS following a 10-week course of active or sham tDCS 60

Supplementary Table 41. Changes in depressive severity at week 10 as measured by HDRS in participants who made a guess that they were receiving active or sham tDCS treatment 60

Supplementary Table 42. Changes in depressive severity at week 10 as measured by HDRS in participants who made a guess that they were receiving active treatment 60

Supplementary Table 43. Changes in depressive severity at week 10 as measured by HDRS shown for participants who made a guess that they were receiving sham treatment 61

Supplementary Materials

Home-based transcranial direct current stimulation treatment for major depressive disorder: a fully remote phase 2 randomized sham-controlled trial

Supplementary Table 44. Distributional changes in severity of depressive symptoms from baseline to week 10 based on Hamilton Depression Rating Scale score for participants who completed the blinded phase (n=149) 62

Supplementary Figure 13. Distribution of depressive symptom severity at week 10 based on Hamilton Depression Rating Scale score for participants who completed the blinded phase (n=149) 63

Supplementary Table 45. Baseline demographic and clinical characteristics of participants who completed the week 20 open-label follow up 64

Supplementary Table 46. Transition analysis of week 10 and week 20 response rates for patients who completed >=660 minutes of stimulation in the open label phase 65

Supplementary Table 47. Transition analysis of week 10 and week 20 response rates for patients who completed >=330 minutes of stimulation in the open label phase 66

Supplementary Table 48. Transition analysis of week 10 and week 20 response rates for patients who completed >=165 minutes of stimulation in the open label phase 67

Supplementary Table 49. Transition analysis of week 10 and week 20 response rates for patients who completed >=80 minutes of stimulation in the open label phase 68

Supplementary Table 50. Transition analysis of week 10 and week 20 response rates for patients who completed >=0 minutes of stimulation in the open label phase 69

Supplementary Table 51. All unanticipated device related adverse events in the open label phase 70

Supplementary Table 52. All unanticipated adverse events in the open label phase 71

Supplementary Table 53. All anticipated adverse events in the open label phase as measured by the tDCS Adverse Events Questionnaire.[22] 72

References 73

Statistical analysis plan - FL001 74

Clinical Study Protocol 95

Supplementary Materials

Home-based transcranial direct current stimulation treatment for major depressive disorder: a fully remote phase 2 randomized sham-controlled trial

Supplementary Notes

Inclusion and exclusion criteria

Inclusion criteria:

1. Age >=18 years.
2. Have a diagnosis of Unipolar MDD with a current depressive episode as defined by the diagnostic criteria in the Diagnostic and statistical manual of mental disorders – 5th edition (DSM-V).[1]
3. Have a Hamilton Depression Rating Score (HDRS)[2] of >=16.
4. For 6 weeks prior to enrolment, are either: a. not taking antidepressant medication or: b. are taking a stable antidepressant regimen with a stable medication source and agree to continue the same regimen throughout study participation.
5. If in psychotherapy, have maintained stable psychotherapy for at least 6 weeks prior to enrolment.
6. Have access to a stable internet connection through which the treatment will be received.
7. Have access to a smartphone or other device running Android 5.0+ or iPhone Operating System (iOS) 12+ (e.g., reasonably new iPhone/iPad or Android phone), used to using the device in their everyday life, and can capably use the study application on the device, as determined by the investigator.
8. Are currently living in England/Wales (UK) or Texas (US).
9. Subject is currently under the care of a psychiatrist or a primary care physician, agrees to be evaluated at regular intervals by a psychiatrist or primary care physician for the duration of study participation, and agrees to promptly inform the study staff of any change of psychiatric or mental health providers during study participation.
10. Subject agrees to allow any and all forms of communication between the investigators/study staff and any healthcare provider who currently provides and/or has provided service to the patient/subject within at least two years of study enrolment.
11. Subject agrees to provide the name and verifiable contact information (email and mailing addresses, mobile and land-line phone numbers, as applicable) of at least two persons ≥ age 18 (22 in the US) who reside within a 60-minute drive of the patient's residence and whom the research staff is at liberty to contact, as they deem necessary, for the duration of study participation
12. Be able to give voluntary, written informed consent to participate and have signed an Informed Consent Form specific to this study.
13. Be willing and able to comply with all study procedures.
14. Subject agrees to meet all of the inclusion criteria throughout their participation in the study. Otherwise, the subject will be discontinued from the study.
15. Subject agrees to a Safety/Suicide Risk Management Protocol, which is intended to reduce the reduce the risk of suicide during study participation.

Exclusion criteria:

1. Are in a current state of mania, as determined by the YMRS[3] or psychosis, as determined by the MINI.[4]
2. Are diagnosed with vitamin or hormonal deficiencies that may mimic mood disorders, as determined by the investigator.
3. Are currently receiving any other interventional therapy for MDD other than a stable regimen of antidepressants or psychotherapy as defined in the inclusion criteria.

4. Considered to have treatment resistant depression as defined by inadequate clinical response to 2 or more trials of antidepressants at an adequate dose and duration.
5. Have a history of electroconvulsive therapy (ECT), transcranial magnetic stimulation (TMS), cranial electrotherapy stimulation (CES), transcranial direct current stimulation (tDCS), deep brain stimulation (DBS), or other brain stimulation.
6. Patient answers Yes to Questions 4, 5 or 6 on the Columbia Suicide Severity Rating Scale (C-SSRS)[5] Triage and Risk Identification Screener.
7. Any previous hospitalization for suicidal behavior.
8. Have chronic severe insomnia (< 4 hours of sleep each night), or depression secondary to chronic insomnia or sleep apnea.
9. Have any structural lesion (e.g., any structural neurological condition, or more subcortical lesions than would be expected for age or have had a stroke that affects stimulated area or connected areas) or any other clinically significant abnormality that might affect safety, study participation, or confound interpretation of study results, as determined by the investigator.
10. Have any implant in the brain (e.g., DBS) or neurocranium, or any other active implantable medical device.
11. Have any neurocranial defect.
12. Have a history of epilepsy or seizures (including history of withdrawal / provoked seizures).
13. Have shrapnel or any ferromagnetic material in the head.
14. Have any disorder that would impair the ability to complete the study questionnaires.
15. Have been diagnosed with autism spectrum disorder.
16. Are actively abusing substances (<1 week prior to enrolment).
17. Have a cognitive impairment (including dementia).
18. Have a history of mania or psychosis.
19. Are currently using any medications that affect cortical excitability (e.g., benzodiazepines, epileptics, etc.).
20. Are currently experiencing symptoms of withdrawal from alcohol or benzodiazepines.
21. Have been diagnosed with Parkinsonism or other movement disorder as determined by the investigator to interfere with treatment.
22. Have ever taken esketamine / ketamine for treatment of depression.
23. Have ever been admitted to hospital for depression.
24. Have ever been diagnosed with obsessive-compulsive disorder (OCD) or bipolar type 1 or 2 disorder.
25. Is diagnosed with an active primary anxiety disorder, or PTSD, agoraphobia, anorexia or bulimia, panic or personality disorder with active symptoms.
26. Have a history of psychosurgery for depression.
27. Have any history of myocardial infarction, coronary artery bypass graft (CABG), coronary heart failure (CHF), or history of other cardiac issues.
28. Are currently experiencing or have a history of intractable migraines.
29. Are a chronic tobacco smoker, as defined by smoking by smoking >100 cigarettes (including hand-rolled cigarettes, cigars, cigarillos, etc.) in their life-time and have smoked every day for the last 7 days.
30. If female and of child-bearing potential, currently pregnant or breastfeeding or planning to become pregnant or breastfeed any time during the study.
31. Are currently a prisoner.
32. Are participating concurrently in another clinical investigation or have participated in a clinical investigation within the last 90 days or intend to participate in another clinical investigation during the study, and where the participation in the other investigation might interfere with the results of this trial as deemed by the PI.

33. Have any medical condition or other circumstances, in the judgment of the investigator, that might interfere with the ability to complete follow-up visits and the self-reported MADRS-s[6] in the app.
34. Have any condition which, in the judgment of the Investigator, would preclude adequate evaluation of the device's safety and performance.
35. A Subject who meets any of the exclusion criteria during study participation will be discontinued from the study.

Previous exclusion criteria which had been amended following trial commencement (protocol version 7, August 31, 2022):

Criteria 24 and 25:

This criterion was amended to the current exclusion criteria 24 and 25. Criteria 24 separated out lifetime OCD and bipolar disorder, and criteria 25 reflected current psychiatric disorders, including anorexia and bulimia:

- Have been diagnosed with obsessive-compulsive disorder (OCD), bipolar type 1 or 2 disorder, an active primary anxiety disorder, PTSD, agoraphobia, panic or personality disorder.

Criterion 8:

This criterion was amended to current exclusion criterion number 8 to clarify that depression was secondary to insomnia or sleep apnea:

- Have chronic or current severe insomnia (< 4 hours of sleep each night), or sleep apnea.

Participant recruitment

Pre-screening assessment consisted of two stages. A potential participant would complete an initial online questionnaire and register in the clinical research organisation (CRO), through the landing page. Participants provided their email, telephone, name and zip code, and answered questions related to current antidepressant treatment and medical history. From the initial questionnaire, a CRO coordinator would arrange a telephone call with the potential participant. During the telephone call, the CRO would ask a series of standardised questions and document the answers in the CRO platform. From the telephone assessment, contact details of potentially eligible participants would be referred to the research site.

The US site had two additional recruitment streams using third party companies. One stream followed the same two pre-screening stages and participants would be referred to the research site if they were deemed potentially eligible after the telephone assessment. The second stream used a software search of their patient database based on study inclusion/exclusion criteria. Potentially suitable patients were identified, and the software sent requests for these patients to fill in a form where they expressed interest in participating in the study. If the patient completed the form, they were automatically added as a participant to be interviewed by the research site.

Supplementary Materials

Home-based transcranial direct current stimulation treatment for major depressive disorder: a fully remote phase 2 randomized sham-controlled trial

Major depressive disorder diagnosis and severity

DSM-5 diagnosis for major depressive episode:

DSM-5 criteria for a major depressive episode includes having 5 or more of the below symptoms, most of the day, nearly every day during a two-week period. Depressed mood or loss of interest/pleasure must be present. The symptoms must be a change from previous functioning and cause significant distress or problems at home, at work, at school, socially, in relationships or in some other important way.

The depressive episode must not be attributable to the physiological effects of a medical condition or a substance, the patient must have no history of manic or hypomanic episode, and the episode is not better explained by a psychotic disorder.

Symptoms:
Depressed mood
Loss of interest or pleasure
Change in appetite/significant change in weight
Insomnia/hypersomnia
Psychomotor agitation or retardation
Fatigue
Feeling worthless or excessive/inappropriate guilt
Decreased concentration
Thoughts of death/suicide

Depression severity based on Hamilton Depression Rating Scale scores:

The 17-item Hamilton Depression Rating Scale is designed to rate the severity of depression in patients. The scale is clinician-administered and contains 17 items pertaining to symptoms of depression experienced over the past week. Scores range from 0 to 52, with higher scores indicating more depression.

Depression severity is categorised using the following scoring categories:

- No depression/remission: 0-7
- Mild: 8-13
- Moderate: 14–18
- Severe: 19–22
- Very severe: ≥23

Safety and tolerability

A safety/suicide risk management protocol was implemented to mitigate suicide risk throughout the study. Prior to participating, all individuals consented to follow the protocol, which involved sharing contact information for at least two adults (age 18 or older in UK, or age 22 or older in USA), residing within an hour's drive, and who could be contacted by the research staff if necessary. The Brown Safety Plan (2012)[7] served as a template. Participants and researchers worked through the plan together to record helpful crisis management strategies and to provide a list of contacts, including the nearest hospital with an emergency department, their GP or psychiatrist contact details, and a suicide prevention helpline.

Supplementary Materials

Columbia-Suicide Severity Rating Scale (C-SSRS)[5] was assessed at each study visit to monitor suicide risk. If a participant had had active suicidal thoughts or any plans, they would be withdrawn from the study, their GP or psychiatrist would be notified, and they would be advised to visit the nearest emergency department or mental health crisis centre. A Hamilton Depression Rating Scale (HDRS)[2] score increase of 30% from baseline would be considered a treatment failure and the participant's GP or psychiatrist would be contacted. The Young Mania Rating Scale (YMRS)[3] was used to assess manic and hypomanic symptoms at each visit.

Interim analysis

An interim analysis was performed when 90 subjects had week 10 data and included both a futility assessment and sample size re-estimation. The futility assessment was based on stochastic curtailing approach by Lachin et al. (2005).[8] The sample size re-estimation will be based on a Promising Zone methodology by Mehta and Pocock (2011)[9] with a Fuzzy design approach introduced by Keenan and Maislin (2014).[10] Separate sample size assessments were performed to evaluate the secondary endpoint of clinical response. Adjustments to the trial for the secondary endpoint were only performed because the primary endpoint met the criteria as dictated by the promising zone criteria. Sample size assessments for the primary and secondary were characterized extensively through simulation analyses and pre-defined methods for controlling operational bias were submitted to FDA for review and agreement prior to the interim analysis be performed. The Interim analysis results had the capacity to modify the trial in two ways for the primary endpoint: 1) Declare the trial futile and stop enrolment, 2) specify a number of subjects between 100 and 270 that is needed for powering the trial. The interim analysis based on the Fuzzy Promising Zone concluded that 6 additional subjects were needed. An analysis was used performed showing that subjects before and after the interim were similar and considered exchangeable.

Statistical methods

Exploratory endpoints were analyzed through summary statistics as means and standard deviations (SD) or percentages and odds ratios. The two groups were compared through Student's t Test or Fisher Exact Test as appropriate. Spearman correlation alone was used to assess the association between two continuous variables. 95% confidence intervals (CI) were presented.

The percentages of subjects that correctly guessed the arm that they were in were compared through Fisher Exact Test.

Subgroup analyses of the primary and secondary endpoints were conducted through stratification by antidepressant usage at baseline and site.

For primary and secondary outcomes in main text and Supplementary Tables 19 and 28, clinical response was defined as >=50% reduction from baseline in HDRS, MADRS and MADRS-s. For all remaining tables in the Supplementary Materials, clinical response was defined as >50% reduction from baseline in HDRS, MADRS and MADRS-s.

Supplementary Materials

Home-based transcranial direct current stimulation treatment for major depressive disorder: a fully remote phase 2 randomized sham-controlled trial

Site heterogeneity

The pre-specified site heterogeneity analysis was performed. The P value for the group x site interaction was p = 0.09. The stratified site analysis shows an estimated change from baseline to Week 10 in HDRS-17 is -2.2 for the US and -2.1 for the UK. Therefore, while statistically observed, the finding is not understood to have clinical interpretation implications, and the sites declared pooled as reported in the manuscript.

Per-Protocol Analysis

The Per Protocol (PP) analysis set was comprised of the following:

- Participants in the mITT analysis set
- Participants with device failure within the 10-week follow-up period
- Participants with deviation from the clinical investigation plan is caused by the investigational device or by problems with respect to tolerability

Participant data which were excluded from the PP analysis set are as follows:

- Participants with major protocol violations that would be expected to confound clinical assessment during follow-up as determined by the PIs.
- Participants who took new pharmaceutical substances or treatments during the clinical investigation which are listed as an exclusion criteria.
- Participants who do not meet the inclusion criteria or exclusion criteria.
- Participants who have performed less than 10 sessions (300 minutes) during the first 3 weeks.

Participants in the PP population were analyzed in the group to which they were randomized.

Acceptability Analysis

A Mann-Whitney U test was conducted on each question in the acceptability questionnaire at baseline and at week 10 to compare the distribution of scores between the active and sham treatment groups.

Participants were asked “How acceptable do you consider the tDCS sessions to be?” at baseline, and “How acceptable did you consider the tDCS sessions to be?” at week 10. At baseline, acceptability was endorsed as being “very acceptable” by the active tDCS group and “quite acceptable” by the sham group and there was no significant difference between the groups (p =0.27). At week 10, both groups rated tDCS as “very acceptable” with no significant difference between group (p = 0.83).

At baseline participants were asked “How helpful do you think the tDCS sessions may be for improving your depressive symptoms?”. Both groups thought that the treatment would be “quite helpful” at baseline with no significant difference in the distribution of scores (p =0.94). When asked at week 10 “How helpful do you think the tDCS sessions were for improving your depressive symptoms?”, the active group thought that the treatment was more helpful than the sham group at week 10, rating the treatment as “quite helpful”, with the sham group rating that the treatment was “a bit helpful”. At baseline, there was a significant difference in the distribution of scores (p = 0.002), with more participants in the sham group rating the treatment and “neither helpful or unhelpful” or one of the three “unhelpful” options.

Supplementary Materials

Home-based transcranial direct current stimulation treatment for major depressive disorder: a fully remote phase 2 randomized sham-controlled trial

Ethicality remained high at “very ethical” for both groups at both time points, however, the distribution of scores across groups was significantly different at both time points. Participants were asked “How ethical do you think the tDCS sessions are?” at both time points. At baseline participants used the full range of scores in the sham group and there was a significant difference in the distribution of scores between the groups (p = 0.03). After receiving the treatment, no participants in either group rated the treatment as “unethical”, and 92% of participants in the active group rated the treatment at “very ethical”, the distribution of scores remained significantly different between groups (p = 0.02).

At baseline participants were asked “How much effort do you think you need to put in for the tDCS sessions?” and both groups median response was a “little bit more than usual” with no significant difference between groups (p = 0.94). This rating remained consistent for the sham group at week 10 when asked “How much effort did you need to put in for the tDCS sessions?”. The active group rated “some more effort than usual” at week 10 but there was no significant difference between the groups (p = 0.06).

Participants were asked “How likely do you think that there will be negative side effects from the tDCS sessions?” at baseline. The active group thought it was “a bit unlikely” that they would be bothered by any side effects from the tDCS and the sham group thought it was “quite unlikely”, there was no significant difference between the groups (p = 0.62) After treatment at week 10, participants were asked “How were you bothered by any negative side effects from the tDCS sessions?”. Both groups median scores reduced by one point with the active group reporting that they were “quite unaffected” by any negative side effects and the sham group reported that they were “very much unaffected” by the side effects. The difference between groups was not significant (p = 0.21).

After receiving the treatment, both groups were asked “Would you recommend the tDCS sessions to others?” at week 10. The active group “would strongly recommend” the tDCS sessions and the sham group “would recommend” the tDCS sessions. The distribution of scores between the groups was not significant (p = 0.15)

Open label transition analysis

A post hoc transition analysis was completed for all participants who completed the blinded phase of the trial and continued in the open label phase of the trial.

Active tDCS treatment arm

- > 95% of participants maintained clinical response from Week 10 to Week 20
- ~40% of participants who did not show a clinical response at Week 10 showed a clinical response at Week 20

Sham tDCS treatment arm

- ~90% of participants maintained clinical response from Week 10 to Week 20
- 50% of participants who did not show a clinical response at Week 10 showed a clinical response at Week 20

The sample size is not large enough to parse out the impact of the amount of simulation time.

Supplementary Materials

Home-based transcranial direct current stimulation treatment for major depressive disorder: a fully remote phase 2 randomized sham-controlled trial

Analysis Code for Longitudinal Model

The following generalized analysis SAS code (SAS Institute, Cary NC Version 9.4) was used for our longitudinal model:

```
proc mixed data = __mi;
        class subject visit [categorical covariates];
        model [change from baseline] = [baseline value] treatment_group [continuous covariates] [categorical covariates] visit treatment * visit / solution;
    repeated visit / subject = subject type = un;
    lsmeans treatment_group * visit / cl diffs;
    by _imputation_;
run;
```

Supplementary Materials

Home-based transcranial direct current stimulation treatment for major depressive disorder: a fully remote phase 2 randomized sham-controlled trial

Supplementary Table 1. Recruitment figures and success rates for sites at each time point

Outcome (N)	Pre-screen questionnaire	Pre-screen telephone interview	Screening interview
**UK**			
Eligible	4954	741	121
Ineligible	5211	900	119
Total	10165	1641	240
Step eligible %	**48.74%**	**45.16%**	**50.42%**
Eligible % vs all	48.74%	7.29%	1.19%
**USA**			
Eligible	1582	238	61
Ineligible	6204	355	67
Total	7786	593	128
Step eligible %	**20.32%**	**40.13%**	**47.66%**
Eligible % vs all	20.32%	3.06%	0.78%
**Total both sites**			
Eligible	6536	979	182
Ineligible	11415	1255	186
Total	17951	2234	368
Step eligible %	**36.41%**	**43.82%**	**49.46%**
Eligible % vs all	36.41%	5.45%	1.01%

8 participants from the UK site who were eligible at the screening interview withdrew before randomisation.

Supplementary Materials

Home-based transcranial direct current stimulation treatment for major depressive disorder: a fully remote phase 2 randomized sham-controlled trial

Supplementary Table 2. Demographic and clinical characteristics of patients at baseline (complementary information)

Characteristic	Active (N=87)	Sham (N=87)
Age by sex		
Men	39.9 ± 11.7	41.7 ± 11.0
Women	35.4 ± 10.5	37.2 ± 10.8
Ethnicity		
Hispanic or Latino	7 (8)	13 (15)
Not Hispanic or Latino	77 (89)	70 (80)
Not reported	3 (3)	3 (3)
Missing	0 (0)	1 (1)

Ethnicity is presented as number of participants with percentage in parentheses. Mean values are presented with '±' standard deviation values. Two-sided significance tests (Fisher exact test for categorical variables and students t-test for continuous variables) found no significant difference between groups for age ((men) p = 0.568, (women) p = 0.339) or ethnicity (p = 0.378).

Supplementary Materials

Home-based transcranial direct current stimulation treatment for major depressive disorder: a fully remote phase 2 randomized sham-controlled trial

Supplementary Table 3. Concomitant medications ongoing at randomisation and medical history

Concomitant medication	Ongoing at randomization		Medical history	
	Active	Sham	Active	Sham
Proton pump inhibitors	0 (0)	1 (1.1)	0 (0)	0 (0)
Aldosterone antagonists	0 (0)	1 (1.1)	0 (0)	0 (0)
Beta blocking agents, non-selective	2 (2.3)	3 (3.4)	1 (1.1)	0 (0)
Thyroid hormones	2 (2.3)	1 (1.1)	0 (0)	0 (0)
Other muscle relaxants, peripherally acting agents	0 (0)	0 (0)	0 (0)	0 (0)
Other centrally acting agents	1 (1.1)	0 (0)	0 (0)	0 (0)
Phenothiazines with piperazine structure	0 (0)	0 (0)	0 (0)	0 (0)
Diazepines, oxazepines, thiazepines and oxepines	0 (0)	1 (1.1)	0 (0)	0 (0)
Other antipsychotics	0 (0)	1 (1.1)	1 (1.1)	0 (0)
Benzodiazepine derivatives	0 (0)	0 (0)	0 (0)	0 (0)
Diphenylmethane derivatives	1 (1.1)	0 (0)	0 (0)	0 (0)
Azaspirodecanedione derivatives	1 (1.1)	0 (0)	0 (0)	0 (0)
Non-selective monoamine reuptake inhibitors	1 (1.1)	3 (3.4)	0 (0)	1 (1.1)
Selective serotonin reuptake inhibitors	40 (46)	35 (40.2)	13 (14.9)	17 (19.5)
Other antidepressants	18 (20.7)	17 (19.5)	4 (4.6)	6 (6.9)
Centrally acting sympathomimetics	2 (2.3)	2 (2.3)	1 (1.1)	1 (1.1)
Phenothiazine derivatives	0 (0)	1 (1.1)	0 (0)	0 (0)

Medical history includes medications that ended in the 6-month period before the randomization date. One participant in the sham group was taking a selective serotonin reuptake inhibitor with unknown start and end dates.

Supplementary Materials

Home-based transcranial direct current stimulation treatment for major depressive disorder: a fully remote phase 2 randomized sham-controlled trial

Supplementary Table 4. Psychotropic combination medications ongoing at randomisation.

Combination medications	Active (N=87)	Sham (N=86)
Antidepressant medication combinations	5 (6)	3 (4)
Antidepressant medication and psychotropic medication	3 (5)	1 (1)
One regular antidepressant medication and regular propranolol	3 (4)	1 (1)
One regular psychotropic medication and additional medications PRN	2 (2)	5 (6)
Two or more PRN medications	1 (1)	0 (0)

PRN, pro re nata. Categories are displayed as number of participants with percentage in parentheses. Antidepressant combinations included two of amitriptyline, bupropion, citalopram, duloxetine, escitalopram, mirtazapine, sertraline or venlafaxine. Antidepressant and psychotropic combinations included one of escitalopram, fluoxetine, sertraline or vortioxetine and one of buspirone, cariprazinem hydroxyzine, lisdexamfetamine, promethazine or trazodone, and one participant was two antidepressant medications and one of a psychotropic medication, Two participants were also taking one medication PRN of mirtazapine or modafinil. One participant was taking two psychotropic medications and one PRN medication. Antidepressants combined with regular propranolol were one of desvenlafaxine, mirtazapine or sertraline. Regular psychotropic medications combined with additional medications PRN were one of buspirone, citalopram, duloxetine, fluoxetine, modafinil, sertraline or venlafaxine regularly, and one of amitriptyline, baclofen, cyclobenzaprine, propranolol, quetiapine or trazadone PRN. Two or more PRN medication were diazepam and prochlorperazine.

Supplementary Materials

Home-based transcranial direct current stimulation treatment for major depressive disorder: a fully remote phase 2 randomized sham-controlled trial

Supplementary Table 5. Length of time in psychotherapy at baseline for participants in the UK sample

Characteristic	Active (N=58)	Sham (N=57)
Individual psychotherapy during trial	6 (10)	8 (14)
Length of time in therapy at baseline		
6 -10 weeks	3 (5)	1 (2)
12 - 52 weeks	3 (5)	5 (9)
> 52 weeks	0 (0)	2 (4)

Categories are displayed as number of participants with percentage in parentheses.

Supplementary Materials

Home-based transcranial direct current stimulation treatment for major depressive disorder: a fully remote phase 2 randomized sham-controlled trial

Supplementary Table 6. Reasons for study withdrawal in the active and sham tDCS groups during the blinded phase of the trial

Withdrawal Reason	Active	Sham
Withdrawn from trial:		
Change in antidepressant treatment during trial	5	4
Change in medications that affect cortical excitability during trial	1	1
Non-compliance with the clinical investigation procedures	3	0
Pregnancy	0	1
Unable to contact participant	2	2
Withdrew consent:		
Withdrew consent due to adverse events	2	2
Withdrew consent due to worsening of condition	0	2
Total	13	12

Supplementary Table 7. withdrawal reasons disaggregated by sex during the 10-week blinded phase of the trial

Withdrawal Reason	Active	Sham
**Women**		
Withdrawn from trial:	6	7
Withdrew consent:	1	3
Total:	7	10
**Men**		
Withdrawn from trial:	5	1
Withdrew consent:	1	1
Total:	6	2

Supplementary Materials

Home-based transcranial direct current stimulation treatment for major depressive disorder: a fully remote phase 2 randomized sham-controlled trial

Supplementary Table 8. Hamilton Depression Rating Scale estimated scores at each timepoint from baseline to week 10

Outcome	Active tDCS (n = 87)	Sham tDCS (n = 86)
**HDRS**		
Baseline score	19.40 ± 3.42	19.13 ± 3.46
Week 1 score	15.45 ± 4.97	14.59 ± 5.04
Week 4 score	11.76 ± 5.50	13.36 ± 5.48
Week 7 score	10.88 ± 5.96	12.20 ± 5.91
Week 10 score	9.58 ± 6.02	11.66 ± 5.96

HDRS, 17-item Hamilton Depression Rating Scale,[2] Mean values are presented with '±' standard deviation values. HDRS scores range from 0 to 52, with higher scores indicating increased depressive symptom severity.

Supplementary Table 9. Montgomery-Åsberg Depression Rating Scale estimated scores at each timepoint from baseline to week 10

Outcome	Active tDCS (n = 87)	Sham tDCS (n = 86)
**MADRS**		
Baseline score	24.80 ± 6.45	23.97 ± 6.52
Week 1 score	20.32 ± 7.78	19.47 ± 7.90
Week 4 score	15.14 ± 8.26	17.75 ± 8.24
Week 7 score	13.88 ± 9.04	16.59 ± 9.00
Week 10 score	12.46 ± 9.40	15.30 ± 9.28

MADRS, Montgomery-Åsberg Depression Rating Scale,[11] Mean values are presented with '±' standard deviation values. MADRS scores range from 0 to 60, with higher scores indicating increased depressive symptom severity.

Supplementary Table 10. Montgomery-Åsberg Depression Rating Scale-self report estimated scores at each timepoint from baseline to week 10

Outcome	Active tDCS (n = 87)	Sham tDCS (n = 86)
**MADRS-s**		
Baseline score	27.35 ± 7.87	26.28 ± 7.95
Week 1 score	22.48 ± 8.94	22.87 ± 9.26
Week 4 score	18.90 ± 9.04	20.83 ± 9.14
Week 7 score	18.94 ± 9.44	19.92 ± 9.72
Week 10 score	16.60 ± 9.33	19.55 ± 9.62

MADRS-s, Montgomery-Åsberg Depression Rating Scale-self report,[6] Mean values are presented with '±' standard deviation values. MADRS-s scores range from 0 to 54, with higher scores indicating increased depressive symptom severity.

Supplementary Materials

Home-based transcranial direct current stimulation treatment for major depressive disorder: a fully remote phase 2 randomized sham-controlled trial

Supplementary Table 11. Multiplicity Adjustment

Given that the primary endpoint has been met, the secondary endpoints can be tested. As specified in the protocol, a Hochberg[13,14] approach is used for controlling multiplicity. The 2-sided p-values (and conversion to 1-sided) are provided along with the corresponding Hochberg-adjusted p-values. After correcting for multiplicity, the secondary endpoints Week 10 HDRS-based remission and Week 10 HDRS-based response are statistically significant based on the covariate-adjusted multiple-imputation-based multiplicity-adjusted 1-sided p-values. The Week 10 EQ-5D-3L endpoint is not statistically significant before or after multiplicity correction.

	**2-sided p-values**	**1-sided p-values**	**1-sided Hochberg adjusted p-values**
Week 10 HDRS response	0.001	0.0005	0.0015
Week 10 HDRS remission	0.004	0.0020	0.0040
Week 10 EQ-5D-3L	0.326	0.1630	0.1630

HDRS, 17-item Hamilton Depression Rating Scale;[2] EQ-5D-3L, quality of life measure.[15–17] HDRS scores range from 0 to 52, with higher scores indicating increased depressive symptom severity. Clinical response was defined as a decrease in the score (indicating less depressive severity) of more than 50% from baseline to week 10. Clinical remission was defined as: HDRS score of 7 or less. Fully Conditional Specification (FCS) approach was used to produce 20 multiply imputed completed data sets. The FCS approach accommodates nonmonotonicity in the pattern of missing data and requires regression models to be specified for each variable with missing values needing imputation. All models included age, sex, in psychotherapy at baseline, use of any antidepressants at baseline and treatment group. The resulting completed datasets were combined using Rubin's Rules.

Supplementary Materials

Home-based transcranial direct current stimulation treatment for major depressive disorder: a fully remote phase 2 randomized sham-controlled trial

Supplementary Table 12. Hamilton Anxiety Rating Scale scores from baseline to week 10

Outcome	Active tDCS (n = 87)	Sham tDCS (n = 86)	Difference	P value
**HAMA**				
Decrease in score	6.6 ± 6.1	4.9 ± 5.9	1.7 (0.2 to 3.7)	0.08
Baseline score	15.4 ± 4.6	14.3 ± 4.6	1.1 (-0.2 to 2.5)	0.11
Week 10 score	8.2 ± 5.7	9.3 ± 4.9	-1.1 (-2.8 to 0.7)	0.23

HAMA, Hamilton Anxiety Rating Scale;[12] tDCS, transcranial direct current stimulation. Plus-minus values are mean ± standard deviation values. P values represent two-sided t-test.

Supplementary Table 13. Young Mania Rating Scale score from baseline to week 10

Outcome	Active tDCS (n = 87)	Sham tDCS (n = 86)	Difference	P value
**YMRS**				
Decrease in score week 10	0.8 ± 2.1	0.2 ± 1.9	0.7 (0.0 to 1.3)	0.05
Baseline score	2.1 ± 1.7	1.9 ± 1.6	0.2 (-0.3 to 0.7)	0.52
Week 1 score	1.6 ± 1.7	1.9 ± 1.6	-0.2 (-0.8 to 0.3)	0.34
Week 4 score	1.5 ± 1.6	1.7 ± 1.4	-0.2 (-0.7 to 0.3)	0.46
Week 7 score	1.6 ± 1.7	1.5 ± 1.6	0.0 (-0.5 to 0.6)	0.92
Week 10 score	1.3 ± 1.4	1.8 ± 1.7	-0.6 (-1.1 to -0.1)	0.03

YMRS, Young Mania Rating Scale;[3] tDCS, transcranial direct current stimulation. Plus-minus values are mean ± standard deviation values. P values represent two-sided t-test.

Supplementary Materials

Home-based transcranial direct current stimulation treatment for major depressive disorder: a fully remote phase 2 randomized sham-controlled trial

Supplementary Table 14. Neuropsychological measure: verbal learning (Rey Auditory Verbal Learning Test (RAVLT) and information processing speed (Symbol-Digit Modalities Test (SDMT) change scores from baseline to week 10

Outcome	Active tDCS	Sham tDCS	Difference	P value
**RAVLT**	**n = 73**	**n = 75**		
Change in score	3.1 ± 9.9	3.6 ± 9.1	-0.5 (-3.6 to 2.5)	0.73
**SDMT**	**n = 69**	**n = 71**		
Change in score	2.5 ± 10.4	2.7 ± 7.5	-0.2 (-3.3 to 2.8)	0.89

SDMT, Symbol Digit Modalities Test;[18] RAVLT, Rey Auditory Verbal Learning Test;[19] tDCS, transcranial direct current stimulation. Plus-minus values are mean ± standard deviation values. RAVLT scores range from 0 to 75. SDMT scores range from 0 to 110. P values represent two-sided t-test.

Supplementary Materials

Home-based transcranial direct current stimulation treatment for major depressive disorder: a fully remote phase 2 randomized sham-controlled trial

Supplementary Figure 1. Rey Auditory Verbal Learning Test (RAVLT)[19] scores from baseline to week 10

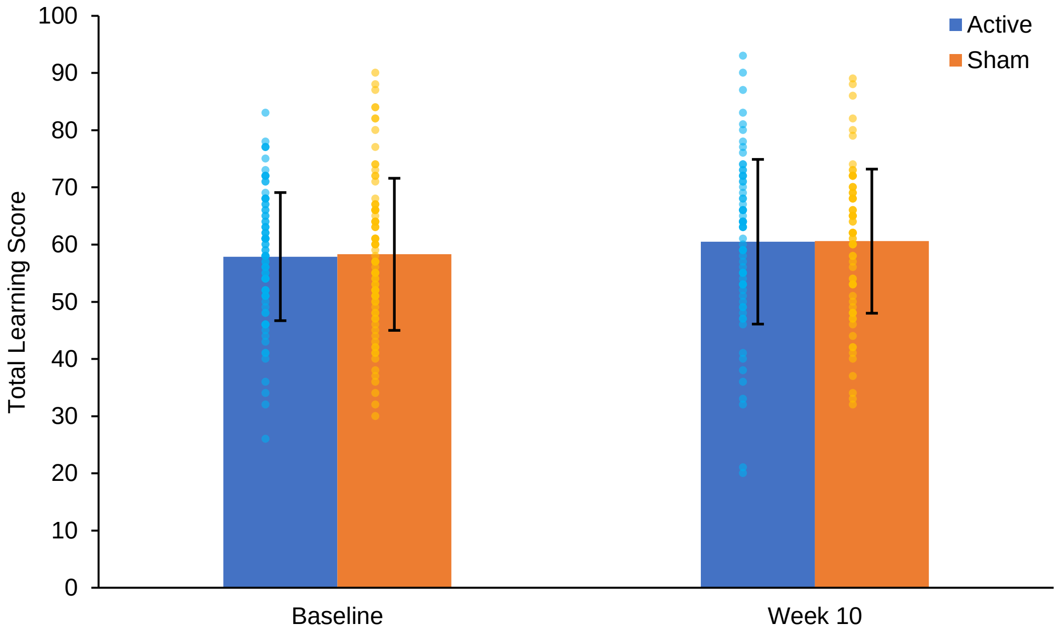

Active group (Baseline n = 87, week 10 n = 73); Sham group (baseline n = 86, week 10 n = 75). Data are presented as mean values with error bars representing +/- 1 standard deviation.

Supplementary Figure 2. Symbol-Digit Modalities Test (SDMT)[18] scores from baseline to week 10

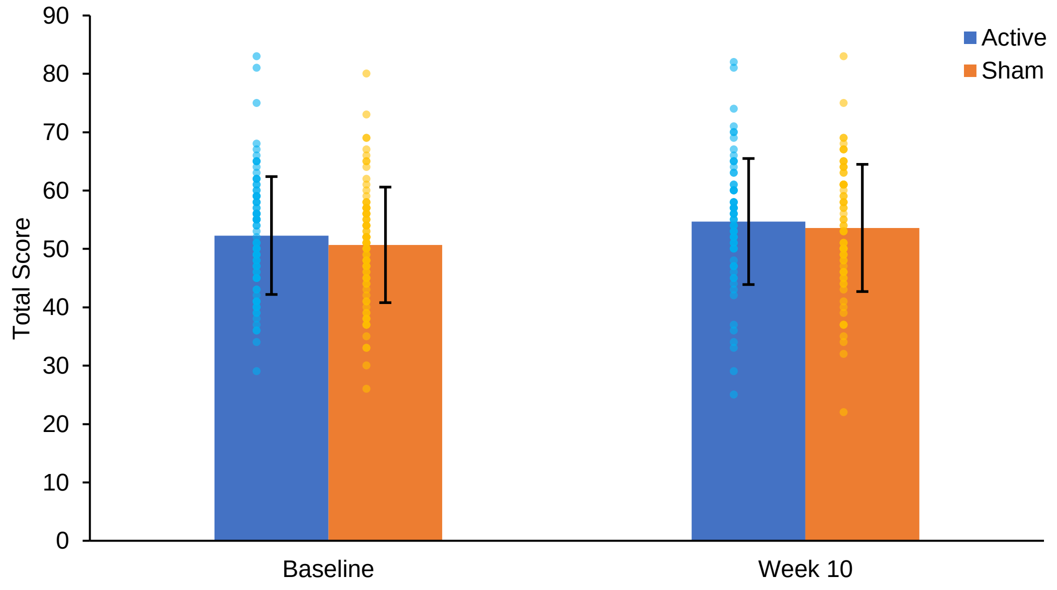

Active group (Baseline n = 85, week 10 n = 69); Sham group (baseline n = 84, week 10 n = 72). Data are presented as mean values with error bars representing +/- 1 standard deviation.

Supplementary Materials

Home-based transcranial direct current stimulation treatment for major depressive disorder: a fully remote phase 2 randomized sham-controlled trial

Supplementary Table 15. Clinical outcomes for participants who were taking antidepressant medication at week 10 end of treatment

Outcome	Active tDCS (n = 56)	Sham tDCS (n = 53)	Difference or Odds Ratio (95% CI)	P value
HDRS				
Decrease in score	10.59 ± 6.26	7.79 ± 5.82	2.81 (0.5 to 5.1)	0.02
Clinical response	29 (63.2%)	15 (35.4%)	3.14 (1.29 to 7.64)	0.01
Clinical remission	25 (53.1%)	11 (25.5%)	3.33 (1.31 to 8.44)	0.009
MADRS				
Decrease in score	12.94 ± 8.96	8.28 ± 8.48	4.66 (1.2 to 8.1)	0.009
Clinical response	33 (70.7%)	18 (43.8%)	3.11 (1.24 to 7.77)	0.02
Clinical remission	30 (67.1%)	17 (42.9%)	2.72 (1.07 to 6.92)	0.04
MADRS-s				
Decrease in score	9.94 ± 9.67	6.38 ± 9.54	3.56 (-0.2 to 7.3)	0.06
Clinical response	20 (49.8%)	10 (26.8%)	2.73 (1.04 to 7.14)	0.04
Clinical remission	22 (57.1%)	14 (31.2%)	2.96 (0.97 to 9.01)	0.05
EQ-5D-3L				
Change in score	0.08 ± 0.16	0.08 ± 0.19	0.04 (-0.01 to 0.09)	0.16

HDRS, Hamilton Depression Rating Scale; MADRS, Montgomery-Åsberg Depression Rating Scale; MADRS-s, Montgomery-Åsberg Depression Rating Scale-self report. EQ-5D-3L, quality of life measure; CI, confidence interval. Mean values are presented with '±' standard deviation values. HDRS, MADRS, MADRS-s change ratings are the change in total ratings from baseline to week 10. Between-group differences are shown for the changes in scores from baseline to week 10, and odds ratios are shown for the outcomes for clinical response and remission. Percentages for clinical response and remission outcomes are estimated based on odds ratios. HDRS scores range from 0 to 52, MADRS scores range from 0 to 60, MADRS-s scores range from 0 to 54, with higher scores indicating increased depressive symptom severity. Clinical response was defined as a decrease in the score (indicating less depressive severity) of more than 50% from baseline to week 10. Clinical remission was defined as: HDRS score of 7 or less; MADRS score of 10 or less; MADRS-s score of 12 or less. Fully Conditional Specification (FCS) approach was used to produce 20 multiply imputed completed data sets. The FCS approach accommodates nonmonotonicity in the pattern of missing data and requires regression models to be specified for each variable with missing values needing imputation. All models included age, sex, in psychotherapy at baseline, use of any antidepressants at baseline and treatment group. The resulting completed datasets were combined using Rubin's Rules.

Supplementary Materials

Home-based transcranial direct current stimulation treatment for major depressive disorder: a fully remote phase 2 randomized sham-controlled trial

Supplementary Table 16. Clinical outcomes for antidepressant medication free participants

Outcome	Active tDCS (n = 31)	Sham tDCS (n = 33)	Difference or Odds Ratio (95% CI)	P value
HDRS				
Decrease in score	7.91 ± 5.96	5.22 ± 6.93	2.69 (0.1 to 5.5)	0.06
Clinical response	12 (49.3%)	6 (10.1%)	8.73 (1.84 to 41.33)	0.001
Clinical remission	9 (36.9%)	6 (9.4%)	5.70 (1.19 to 27.20)	0.02
MADRS				
Decrease in score	8.15 ± 8.76	5.72 ± 9.51	2.43 (-1.6 to 6.4)	0.23
Clinical response	13 (52.3%)	7 (14.0%)	6.76 (1.69 to 26.93)	0.002
Clinical remission	12 (48.6%)	8 (11.3%)	7.41 (1.61 to 34.19)	0.005
MADRS-s				
Decrease in score	10.19 ± 10.44	5.91 ± 10.47	4.27 (-1.1 to 9.6)	0.19
Clinical response	10 (40.2%)	4 (16.7%)	5.05 (1.25 to 20.35)	0.01
Clinical remission	10 (51.4%)	4 (13.2%)	7.02 (1.54 to 32.00)	0.005
EQ-5D-3L				
Change in score	0.05 ± 0.14	0.05 ± 0.14	-0.01 (-0.07 to 0.04)	0.62

HDRS, 17-item Hamilton Depression Rating Scale;[2] MADRS, Montgomery-Åsberg Depression Rating Scale;[11] MADRS-s, Montgomery-Åsberg Depression Rating Scale-self report.[6] EQ-5D-3L, quality of life measure;[15–17] CI, confidence interval. Mean values are presented with '±' standard deviation values. HDRS, MADRS, MADRS-s change ratings are the change in total ratings from baseline to week 10. Between-group differences are shown for the changes in scores from baseline to week 10, and odds ratios are shown for the outcomes for clinical response and remission. Percentages for clinical response and remission outcomes are estimated based on odds ratios. HDRS scores range from 0 to 52, MADRS scores range from 0 to 60, MADRS-s scores range from 0 to 54, with higher scores indicating increased depressive symptom severity. Clinical response was defined as a decrease in the score (indicating less depressive severity) of more than 50% from baseline to week 10. Clinical remission was defined as: HDRS score of 7 or less; MADRS score of 10 or less; MADRS-s score of 12 or less. Fully Conditional Specification (FCS) approach was used to produce 20 multiply imputed completed data sets. The FCS approach accommodates nonmonotonicity in the pattern of missing data and requires regression models to be specified for each variable with missing values needing imputation. All models included age, sex, in psychotherapy at baseline, use of any antidepressants at baseline and treatment group. The resulting completed datasets were combined using Rubin's Rules.

Supplementary Materials

Home-based transcranial direct current stimulation treatment for major depressive disorder: a fully remote phase 2 randomized sham-controlled trial

Supplementary Table 17. Per protocol analysis reasons for exclusion

Per protocol exclusion reason	Number of participants
Participants with major protocol violations that would be expected to confound clinical assessment during follow-up as determined by the PIs.	15
Participants who took new pharmaceutical substances or treatments during the clinical investigation which are listed as an exclusion criteria.	14
Participants who do not meet the inclusion criteria or exclusion criteria.	16
Participants who have performed less than 10 sessions (300 minutes) during the first 3 weeks.	24

Participants could meet more than one exclusion criteria. 2 participants completed less than 10 sessions during the first 3 weeks but were included in the PP analysis due to device failure.

Supplementary Materials

Home-based transcranial direct current stimulation treatment for major depressive disorder: a fully remote phase 2 randomized sham-controlled trial

Supplementary Table 18. Per protocol analysis of changes in depressive severity as measured by HDRS, MADRS and MADRS-s and quality of life as measured by EQ-5D-3L following 10-week course of active or sham tDCS

Outcome	Active tDCS (n = 61)	Sham tDCS (n = 70)	Difference or Odds Ratio (95% CI)	Cohen's D	P value
HDRS					
Decrease in score	10.37 ± 5.86	7.91 ± 5.85	2.47 (0.7 to 4.2)	0.42	0.005
Clinical response	33 (53.6%)	19 (26.4%)	3.23 (1.55 to 6.74)	-	0.002
Clinical remission	28 (44.8%)	15 (20.0%)	3.25 (1.50 to 7.04)	-	0.003
MADRS					
Decrease in score	12.14 ± 8.15	8.93 ± 8.15	3.21 (0.8 to 5.7)	0.39	0.01
Clinical response	37 (60.9%)	23 (31.1%)	3.44 (1.64 to 7.22)	-	0.001
Clinical remission	35 (58.6%)	23 (30.0%)	3.31 (1.54 to 7.13)	-	0.002
MADRS-s					
Decrease in score	10.82 ± 9.43	6.92 ± 9.44	3.90 (0.9 to 6.9)	0.41	0.01
Clinical response	25 (47.3%)	13 (21.8%)	3.22 (1.42 to 7.32)	-	0.005
Clinical remission	28 (53.5%)	16 (21.0%)	4.33 (1.78 to 10.54)	-	0.001
EQ-5D-3L					
Change in score	0.07 ± 0.15	0.06 ± 0.15	0.01 (-0.03 to 0.05)	0.08	0.56

HDRS, Hamilton Depression Rating Scale;[2] MADRS, Montgomery-Åsberg Depression Rating Scale;[11] MADRS-s, Montgomery-Åsberg Depression Rating Scale-self report;[6] EQ-5D-3L, quality of life measure;[15–17] CI, confidence interval. Mean values are presented with '±' standard deviation values. HDRS, MADRS, MADRS-s change ratings are the change in total ratings from baseline to week 10. Between-group differences are shown for the changes in scores from baseline to week 10, and odds ratios are shown for the outcomes for clinical response and remission. Percentages for clinical response and remission outcomes are estimated based on odds ratios. HDRS scores range from 0 to 52; MADRS scores range from 0 to 60; MADRS-s scores range from 0 to 54, with higher scores indicating increased depressive symptom severity. Clinical response was defined as a decrease in the score (indicating less depressive severity) of more than 50% from baseline to week 10. Clinical remission was defined as: HDRS score of 7 or less; MADRS score of 10 or less; MADRS-s score of 12 or less. Fully Conditional Specification (FCS) approach was used to produce 20 multiply imputed completed data sets. The FCS approach accommodates nonmonotonicity in the pattern of missing data and requires regression models to be specified for each variable with missing values needing imputation. All models included age, sex, in psychotherapy at baseline, use of any antidepressants at baseline and treatment group. The resulting completed datasets were combined using Rubin's Rules. Estimated Standard Effect Size (Cohen's D) is the group difference in estimated means divided by pooled within group SD.

Supplementary Materials

Home-based transcranial direct current stimulation treatment for major depressive disorder: a fully remote phase 2 randomized sham-controlled trial

Supplementary Table 19. Changes in depressive severity as measured by HDRS, MADRS and MADRS-s and quality of life as measured by EQ-5D-3L at 7 weeks

Outcome	Active tDCS (n = 87)	Sham tDCS (n = 86)	Difference or Odds Ratio (95% CI)	Cohen's D or NNT	P value
HDRS					
Decrease in score	8.10 ± 6.27	6.60 ± 6.10	1.50 (-0.2 to 3.2)	0.24	0.09
Clinical response	36 (48.7%)	29 (37.9%)	1.55 (0.81 to 3.01)	10	0.20
Clinical remission	27 (37.1%)	15 (20.5%)	2.30 (1.04 to 5.08)	6	0.04
MADRS					
Decrease in score	9.88 ± 8.46	6.46 ± 8.27	3.57 (1.1 to 5.8)	0.41	0.005
Clinical response	40 (53.7%)	26 (33.0%)	2.36 (1.20 to 4.65)	5	0.013
Clinical remission	34 (47.7%)	19 (21.8%)	3.28 (1.49 to 7.20)	4	0.002
MADRS-s					
Decrease in score	7.55 ± 9.10	5.88 ± 9.29	1.67 (-0.9 to 4.3)	0.18	0.21
Clinical response	29 (39.8%)	15 (23.1%)	2.21 (1.06 to 4.63)	6	0.035
Clinical remission	24 (33.2%)	16 (21.5%)	1.81 (0.83 to 3.97)	9	0.14

HDRS, 17-item Hamilton Depression Rating Scale;[2] MADRS, Montgomery-Åsberg Depression Rating Scale;[11] MADRS-s, Montgomery-Åsberg Depression Rating Scale-self report;[6] EQ-5D-3L, quality of life measure;[15–17] CI, confidence interval; NNT, number needed to treat. Mean values are presented with '±' standard deviation values. HDRS, MADRS, MADRS-s change ratings are the change in total ratings from baseline to week 7. Between-group differences are shown for the changes in scores from baseline to week 7, and odds ratios are shown for the outcomes for clinical response and remission. Percentages for clinical response and remission outcomes are estimated based on odds ratios. HDRS scores range from 0 to 52; MADRS scores range from 0 to 60; MADRS-s scores range from 0 to 54, with higher scores indicating increased depressive symptom severity. Clinical response was defined as a decrease in the score (indicating less depressive severity) of 50% or more from baseline to week 10. Clinical remission was defined as: HDRS score of 7 or less; MADRS score of 10 or less; MADRS-s score of 12 or less. Fully Conditional Specification (FCS) approach was used to produce 20 multiply imputed completed data sets. The FCS approach accommodates nonmonotonicity in the pattern of missing data and requires regression models to be specified for each variable with missing values needing imputation. All models included age, sex, in psychotherapy at baseline, use of any antidepressants at baseline and treatment group. The resulting completed datasets were combined using Rubin's Rules. Estimated Standard Effect Size (Cohen's D) is the group difference in estimated means divided by pooled within group SD.

Supplementary Materials

Home-based transcranial direct current stimulation treatment for major depressive disorder: a fully remote phase 2 randomized sham-controlled trial

Supplementary Table 20. Changes in depressive severity as measured by HDRS, disaggregated by sex at week 10

Outcome	Active	Sham
Women	n=47	n=57
HDRS		
Decrease in score	10.28 ± 5.50	7.70 ± 4.86
Clinical response	28 (59.6%)	15 (26.3%)
Clinical remission	24 (51.1%)	12 (21.1%)
Men	n=27	n=18
HDRS		
Decrease in score	10.19 ± 5.15	7.94 ± 5.60
Clinical response	13 (48.1%)	6 (33.3%)
Clinical remission	10 (37.0%)	5 (27.8%)

HDRS, 17-item Hamilton Depression Rating Scale;[2] Mean values are presented with '±' standard deviation values. HDRS change ratings are the change in total ratings from baseline to week 10. Between-group differences are shown for the changes in scores from baseline to week 10. HDRS scores range from 0 to 52, with higher scores indicating increased depressive symptom severity. Clinical response was defined as a decrease in the score (indicating less depressive severity) of more than 50% from baseline to week 10. Clinical remission was defined as HDRS score of 7 or less. Data is for participants with week 10 data (n =149).

Supplementary Materials

Home-based transcranial direct current stimulation treatment for major depressive disorder: a fully remote phase 2 randomized sham-controlled trial

Supplementary Figure 3. Correlation between change from baseline to 10 weeks in MADRS and MADRS-s

The correlation between change from baseline to 10 weeks in MADRS and MADRS-s is an exploratory endpoint in the trial. There are 174 subjects randomized in the FL001 trial. Of the 174 subjects, 45 subjects had missing MADRS or MADRS-s data at week 10. Of the 45 subjects, 22 of the subjects had early withdrawal from the trial, 3 subjects were missing MADRS data at week 10 and 20 subjects were missing MADRS-s data at week 10. The remaining 129 subjects were included in the correlation analysis. Percent of sessions with stimulation was calculated as the observed stimulation time between weeks 1 through 10 divided by the total number of expected minutes based on protocol recommendations (1080 = 30*5*3+30*3*7). The scatter plot shows the change from baseline to 10 weeks in MADRS-s score (madrs_s_score_chg) versus the change from baseline to 10 weeks in MADRS score (madrs_score_chg):

With the 129 observations, there was an observed correlation of 0.49 between the percent of sessions with stimulation and the change from baseline to 10 weeks in MADRS score (two-tailed Pearson correlation *p* = 0.000000005). Therefore, there is a statistically significant correlation between the percent of sessions with stimulation and the change from baseline to 10 weeks in MADRS score. The correlation between the two measures would be considered moderate. From Fantino and Moore,[20] the investigators observed a Pearson's correlation between MADRS and MADRS-s of r=0.54 in subjects with major depressive disorder. The observed correlation of 0.49 in the trial is well aligned with the results from the Fantino and Moore (2009) study.[20]

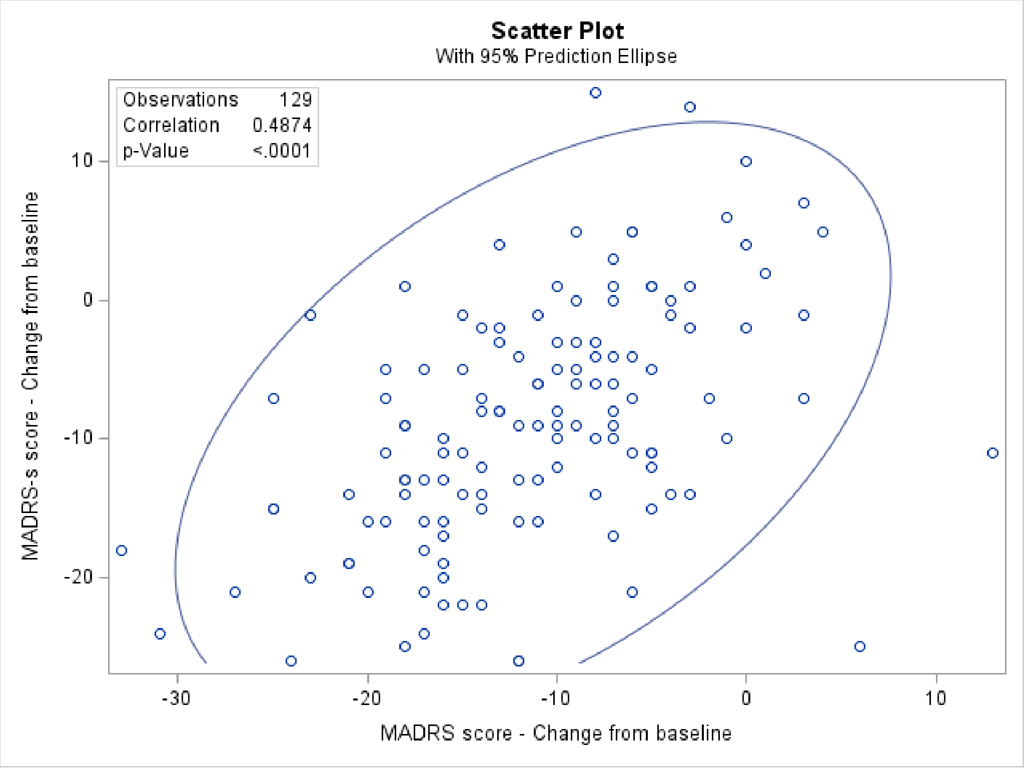

Supplementary Materials

Home-based transcranial direct current stimulation treatment for major depressive disorder: a fully remote phase 2 randomized sham-controlled trial

Supplementary Figure 4. Correlation between change from baseline to 10 weeks in HDRS and percent of sessions with stimulation in the active group

The correlation between change from baseline to 10 weeks in HDRS and percent of sessions with stimulation in the active group is an exploratory endpoint in the trial. There are 87 subjects randomized to the active group in the FL001 trial. Of the 87 subjects, 13 subjects had missing HDRS-17 data at week 10. All 13 of the subjects had early withdrawal from the trial. The remaining 74 subjects were included in the correlation analysis. Percent of sessions with stimulation was calculated as the observed stimulation time between weeks 1 through 10 divided by the total number of expected minutes based on protocol recommendations (1080 = 30*5*3+30*3*7). The scatter plot shows the graphical representation of the percent of sessions with stimulation (pct_sessions) versus the change from baseline to 10 weeks in HDRS-17 score (hdrs_17_score_chg):

With the 74 observations, there was an observed correlation of -0.102 between the percent of sessions with stimulation and the change from baseline to 10 weeks in HDRS-17 score (two-sided Pearson correlation p=0.3888). Therefore, there is not a statistically significant correlation between the percent of sessions with stimulation and the change from baseline to 10 weeks in HDRS-17 score. The correlation between the two measures would be considered weak.

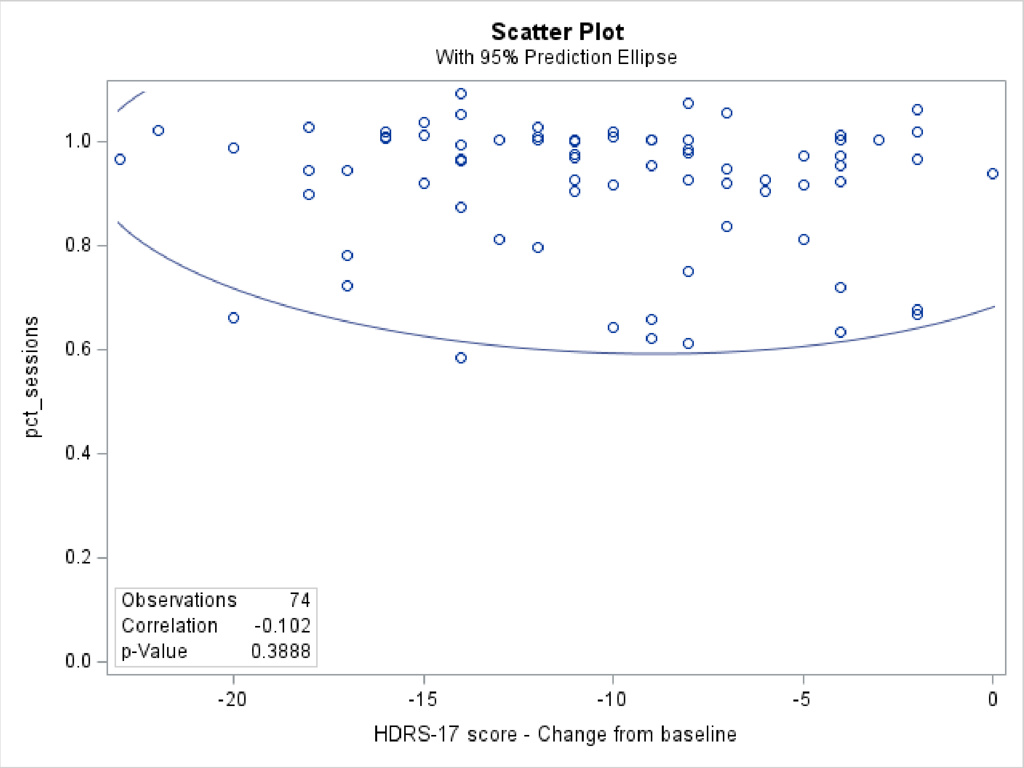

Supplementary Materials

Home-based transcranial direct current stimulation treatment for major depressive disorder: a fully remote phase 2 randomized sham-controlled trial

Supplementary Figure 5. Correlation between change from baseline to 10 weeks in MADRS and percent of sessions with stimulation in the active group

The correlation between change from baseline to 10 weeks in MADRS and percent of sessions with stimulation in the active group is an exploratory endpoint in the trial. There are 87 subjects randomized to the active group in the FL001 trial. Of the 87 subjects, 13 subjects had missing MADRS data at week 10. All 13 of the subjects had early withdrawal from the trial. The remaining 74 subjects were included in the correlation analysis. Percent of sessions with stimulation was calculated as the observed stimulation time between weeks 1 through 10 divided by the total number of expected minutes based on protocol recommendations (1080 = 30*5*3+30*3*7). The scatter plot shows the graphical representation of the percent of sessions with stimulation (pct_sessions) versus the change from baseline to 10 weeks in MADRS score (madrs_score_chg):

With the 74 observations, there was an observed correlation of -0.08 between the percent of sessions with stimulation and the change from baseline to 10 weeks in MADRS score (two-sided Pearson correlation p=0.4919). Therefore, there is not a statistically significant correlation between the percent of sessions with stimulation and the change from baseline to 10 weeks in MADRS score. The correlation between the two measures would be considered weak.

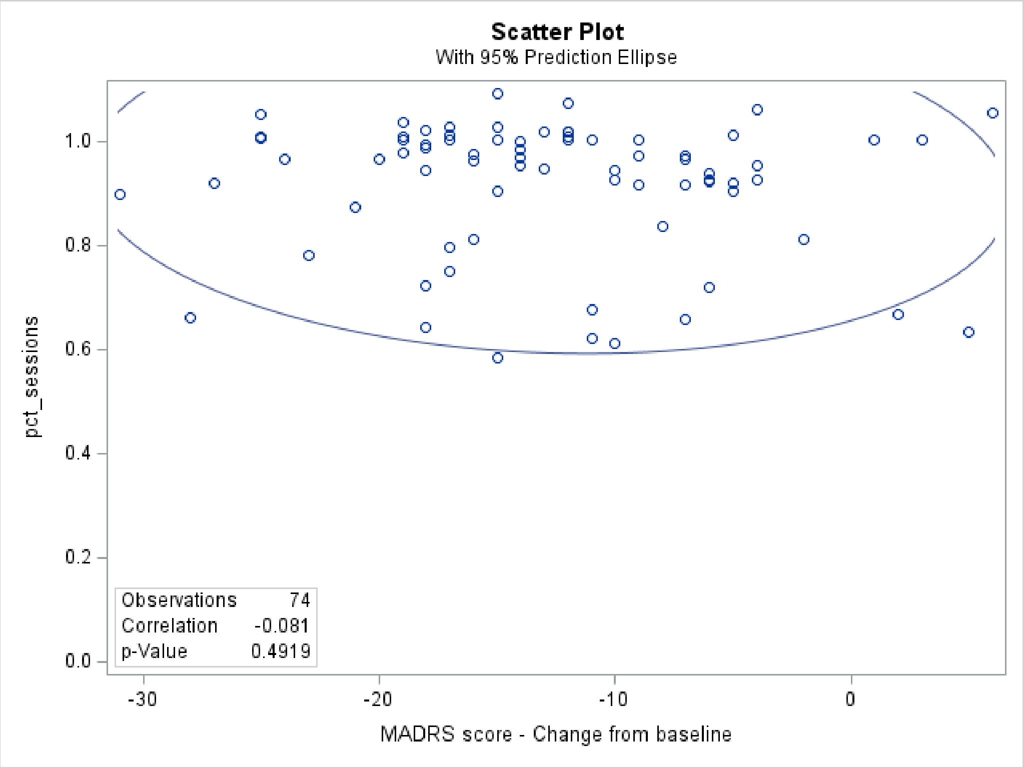

Supplementary Materials

Home-based transcranial direct current stimulation treatment for major depressive disorder: a fully remote phase 2 randomized sham-controlled trial

Supplementary Table 21. Treatment acceptability questionnaire (TAQ) and responses at baseline (n = 174 (active = 87, sham = 87)), at the end of treatment (n = 151 (active = 75, sham = 76)). Italics represent post-treatment phrasing

				Likert Ratings													
	Median (IQR)			1		2		3		4		5		6		7	
**Question**	**Active tDCS**	**Sham tDCS**	**P Value**	**Active tDCS**	**Sham tDCS**	**Active tDCS**	**Sham tDCS**	**Active tDCS**	**Sham tDCS**	**Active tDCS**	**Sham tDCS**	**Active tDCS**	**Sham tDCS**	**Active tDCS**	**Sham tDCS**	**Active tDCS**	**Sham tDCS**
**How acceptable do *(did)* you consider the tDCS sessions to be?**				**Very unacceptable**		**Quite unacceptable**		**Unacceptable**		**Neither**		**Acceptable**		**Quite acceptable**		**Very acceptable**	
Baseline	7 (1)	6 (2)	0.27	1 (1%)	4 (5%)	1 (1%)	2 (2%)	0 (0%)	0 (0%)	11 (13%)	14 (16%)	4 (5%)	5 (6%)	25 (29%)	21 (24%)	45 (52%)	41 (47%)
After 10 weeks treatment	7 (1)	7 (1)	0.83	0 (0%)	0 (0%)	0 (0%)	4 (5%)	0 (0%)	2 (3%)	2 (3%)	3 (4%)	3 (4%)	4 (5%)	24 (32%)	14 (18%)	46 (61%)	49 (64%)
**How helpful do you think the tDCS sessions may be *(were)* for improving your depressive symptoms?**				**Very unhelpful**		**Quite unhelpful**		**Bit unhelpful**		**Neither**		**Bit helpful**		**Quite helpful**		**Very helpful**	
Baseline	6 (1)	6 (1)	0.94	0 (0%)	0 (0%)	1 (1%)	1 (1%)	2 (2%)	1 (1%)	8 (9%)	8 (9%)	26 (30%)	30 (34%)	36 (41%)	31 (36%)	14 (16%)	16 (18%)
After 10 weeks treatment	6 (2)	5 (2)	0.002	0 (0%)	3 (4%)	1 (1%)	3 (4%)	1 (1%)	1 (1%)	9 (12%)	19 (25%)	21 (28%)	20 (26%)	21 (28%)	20 (26%)	22 (29%)	10 (13%)
**How likely do you think that there will be negative side effects from the tDCS sessions? / *How were you bothered by any negative side effects from the tDCS sessions?***				**Very unlikely/ Very much unaffected**		**Quite unlikely/ Quite unaffected**		**Bit unlikely/ Bit unaffected**		**Neither**		**Bit likely/ Bit affected**		**Quite likely/ Quite affected**		**Very likely/ Very affected**	
Baseline	3 (2)	2 (2)	0.62	17 (20%)	15 (17%)	26 (30%)	31 (36%)	14 (16%)	15 (17%)	17 (20%)	18 (21%)	11 (13%)	7 (8%)	1 (1%)	1 (1%)	1 (1%)	0 (0%)
After 10 weeks treatment	2 (2)	1 (1)	0.21	29 (39%)	39 (52%)	26 (35%)	19 (25%)	4 (5%)	2 (3%)	1 (1%)	4 (5%)	14 (19%)	10 (13%)	1 (1%)	1 (1%)	0 (0%)	1 (1%)
**How ethical do you think the tDCS sessions are?**				**Very unethical**		**Quite unethical**		**Bit unethical**		**Neither**		**Bit ethical**		**Quite ethical**		**Very ethical**	
Baseline	7 (1)	7 (1)	0.03	0 (0%)	1 (1%)	0 (0%)	4 (5%)	1 (1%)	3 (3%)	6 (7%)	5 (6%)	0 (0%)	0 (0%)	20 (23%)	27 (31%)	60 (69%)	47 (54%)
After 10 weeks treatment	7 (0)	7 (0)	0.02	0 (0%)	0 (0%)	0 (0%)	0 (0%)	0 (0%)	0 (0%)	2 (3%)	5 (7%)	0 (0%)	1 (1%)	4 (5%)	10 (13%)	69 (92%)	60 (79%)
**How much effort do you think you need *(did you need)* to put in for the tDCS sessions?**				**Very much more than usual**		**Some more than usual**		**Little bit more than usual**		**Same as usual**		**Bit less than usual**		**Quite less than usual**		**Very much less than usual**	
Baseline	3 (2)	3 (2)	0.94	4 (5%)	3 (3%)	22 (25%)	22 (25%)	27 (31%)	29 (33%)	25 (29%)	26 (30%)	4 (5%)	3 (3%)	3 (3%)	2 (2%)	2 (2%)	2 (2%)
After 10 weeks treatment	4 (3)	3 (1)	0.06	0 (0%)	2 (3%)	5 (7%)	7 (9%)	27 (36%)	33 (43%)	19 (25%)	18 (24%)	2 (3%)	3 (4%)	13 (17%)	8 (11%)	9 (12%)	5 (7%)
**Would you recommend the tDCS sessions to others?**				**Would very strongly not recommend**		**Would strongly not recommend**		**Would not recommend**		**Would not for or against**		**Would recommend**		**Would strongly recommend**		**Would very strongly recommend**	
After 10 weeks treatment	6 (2)	5 (1.5)	0.15	1 (1%)	0 (0%)	1 (1%)	1 (1%)	1 (1%)	1 (1%)	6 (8%)	16 (21%)	22 (30%)	20 (27%)	19 (25%)	18 (24%)	25 (33%)	20 (26%)

tDCS, transcranial direct current stimulation; IQR, inter-quartile range.

Supplementary Materials

Home-based transcranial direct current stimulation treatment for major depressive disorder: a fully remote phase 2 randomized sham-controlled trial

Supplementary Figure 6. Percentage of participants in active and sham groups who endorsed each response in the acceptability questionnaire at baseline and week 10

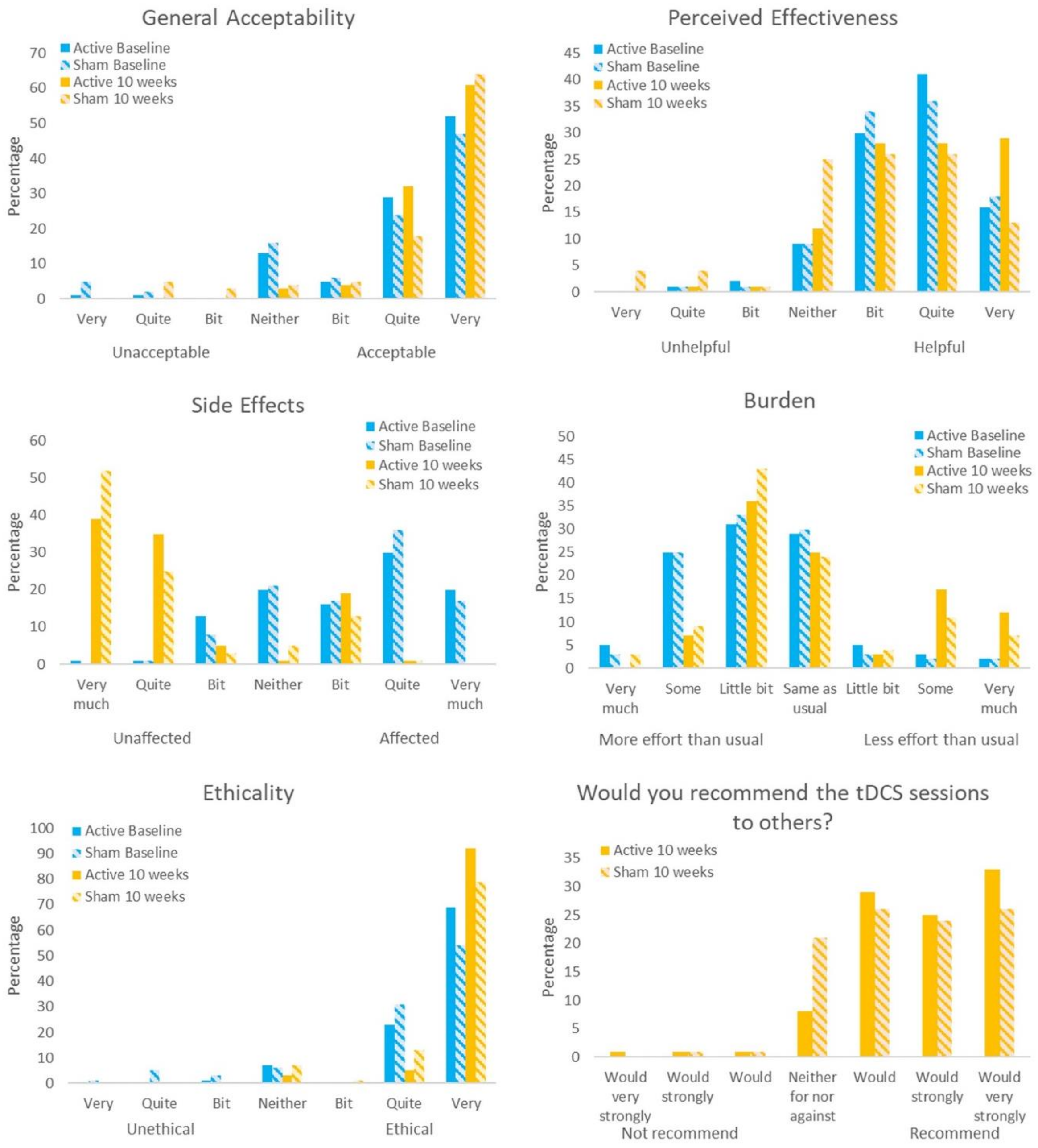

Supplementary Materials

Home-based transcranial direct current stimulation treatment for major depressive disorder: a fully remote phase 2 randomized sham-controlled trial

Supplementary Figure 7. Change in individual depressive severity ratings over time.

Shown are the observed 17-item Hamilton Rating Scale for Depression (HDRS) rating scores from baseline to week 10 in the modified intention-to-treat analysis sample (n=173) for the active tDCS (N=87) and sham tDCS (N=86) treatment arms. Plots contain patient specific summaries, consisting of the data that was used in the primary input model without imputation. The estimated mean HDRS score at each timepoint is shown in green. HDRS scores range from 0 to 52 with higher values indicating more severe depressive symptoms. 30 was selected for the Y axis given that it captured all of the patient data.

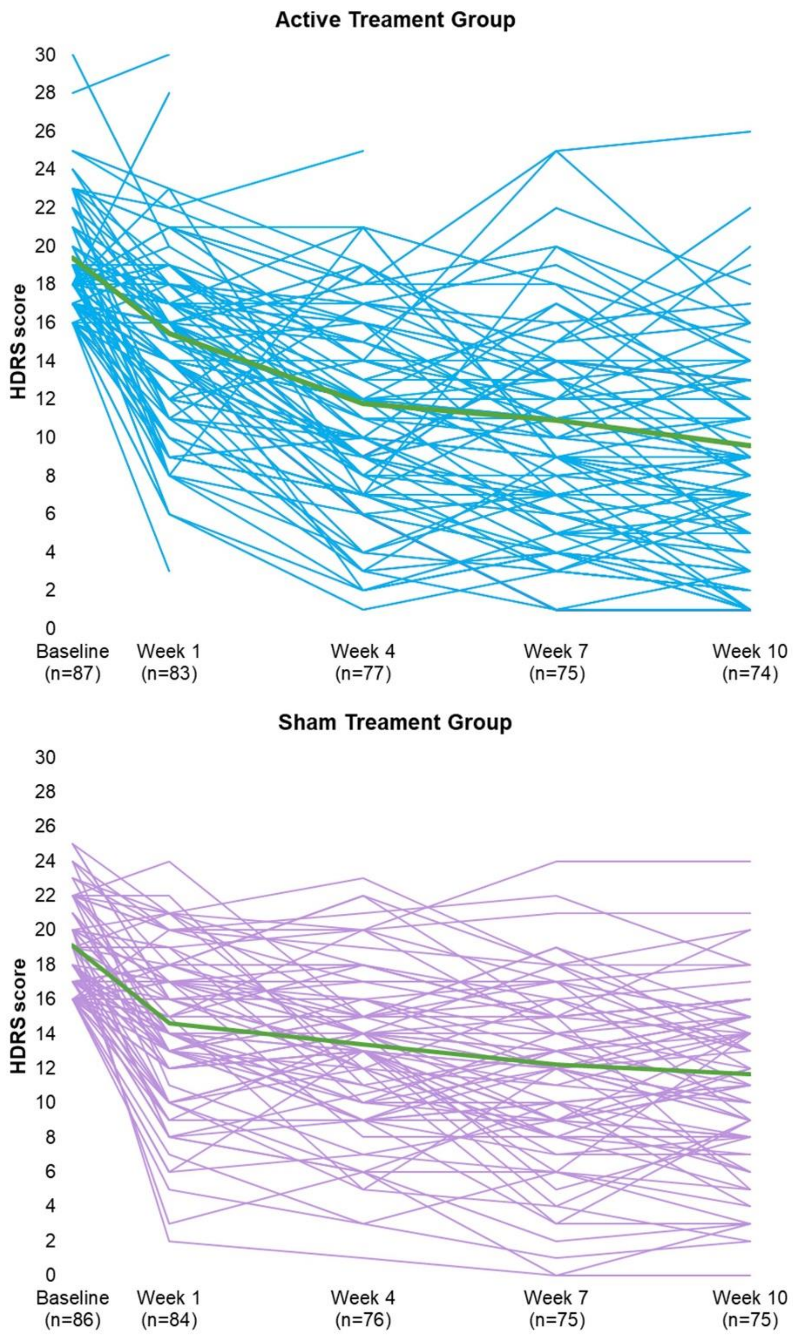

Supplementary Materials

Home-based transcranial direct current stimulation treatment for major depressive disorder: a fully remote phase 2 randomized sham-controlled trial

Supplementary Figure 8. Percentage of participants who completed stimulations at each week in the mITT sample (n=173).

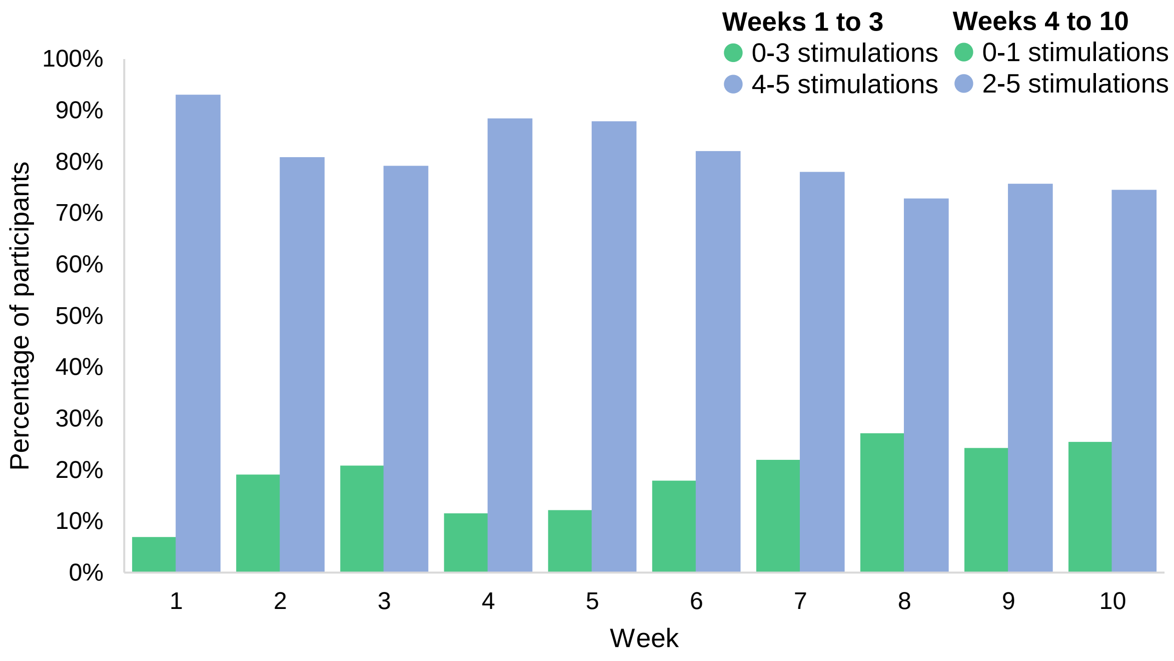

Supplementary Figure 9. Percentage of participants who completed stimulations at each week for participants who completed the 10 week blinded phase (n=149).

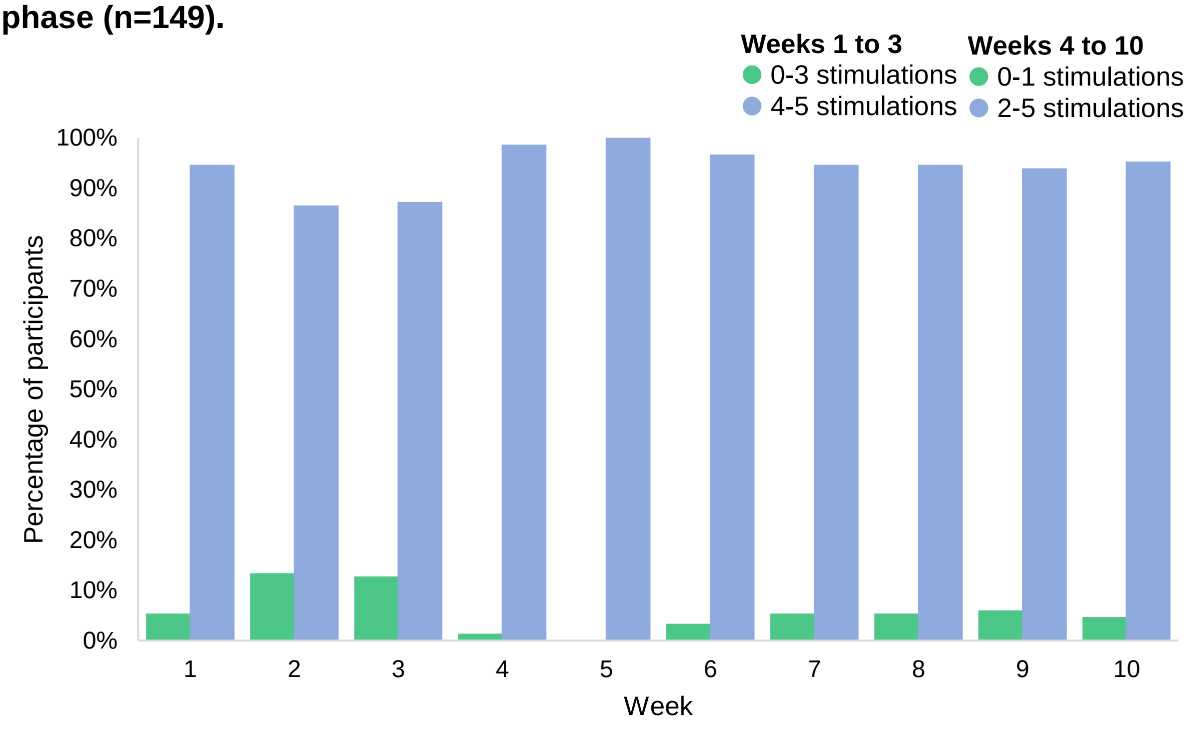

Supplementary Materials

Home-based transcranial direct current stimulation treatment for major depressive disorder: a fully remote phase 2 randomized sham-controlled trial

Supplementary Table 22. Number of stimulations completed in the active and sham groups at week 10

Number of stimulations completed	Active group (n=87)	Sham group (n=86)	P value
Mean number of stimulations	29.94 ± 9.61	31.21 ± 8.00	0.35
100% (36 stimulations)	28 (32.2)	25 (29.1)	
90% or more (32 or more stimulations)	58 (66.7)	61 (70.9)	
60% or more (22 or more stimulations)	76 (87.4)	77 (89.5)	
Less than 60% (Less than 22 stimulations)	11 (12.6)	9 (10.5)	

Mean values are presented with '±' standard deviation values. The number of participants is presented with the percentage in parenthesis. The maximum number of stimulation sessions available was 36 during the 10-week RCT blinded phase. 90% of the maximum number is 32 stimulation sessions. The minimum number of stimulation sessions for a participant to be included in the analysis was 22 stimulation sessions as stated in the Statistical Analysis Plan. P value represents two-sided independent samples t-test.

Supplementary Materials

Home-based transcranial direct current stimulation treatment for major depressive disorder: a fully remote phase 2 randomized sham-controlled trial

Supplementary Table 23. Number of participants who showed a 'clinically meaningful improvement' at each time point (indicated by a HDRS scores of -3 from baseline)

The percentage of subjects for each arm that reached -3 points or more improvement at each time point from week 1 to week 10. -3 points is the smallest change in measurement on the HDRS that signifies a 'clinically meaningful improvement' in an individual patient.

Outcome	Active tDCS (n = 87)	Sham tDCS (n = 86)	Difference	P value
**Clinically meaningful improvement**				
Week 1	49 (59.0%)	56 (66.7%)	-7.6 (-22.2 to 7.0)	0.308
Week 4	62 (80.5%)	59 (77.6%)	2.9 (-10.0 to 15.8)	0.661
Week 7	67 (89.3%)	62 (82.7%)	6.7 (-4.4 to 17.7)	0.239
Week 10	68 (91.9%)	65 (86.7%)	5.2 (-4.7 to 15.1)	0.303

tDCS, transcranial direct current stimulation. P values represent two-sided Fisher exact test.

Supplementary Materials

Home-based transcranial direct current stimulation treatment for major depressive disorder: a fully remote phase 2 randomized sham-controlled trial

Supplementary Figure 10. Cumulative proportion of responders analysis for HDRS scores

A Cumulative Proportion of Responders Analysis (CPRA) was performed per the Statistical Analysis Plan (SAP). The following figure shows:

- X-axis: The change from baseline to Week 10 in HDRS
- Y-axis: The percentage (%) of subjects experiencing a reduction greater than 'k' from the baseline in HDRS.
- The blue line is the Active group, and the red line is Sham.

Supplementary Figure 10 shows the percentage of subjects experiencing a response as a function of the change in the HDRS from baseline. For this endpoint, the "more negative" primary endpoint change from baseline indicates a better outcome subject. The observed cumulative percent response in the treatment group is more negative than the control group for all observed HDRS from baseline.

The difference can be further summarized by the table above with cumulative response group difference by specified cut points. The difference between the groups is depicted by an initial separation of the curves with a maximum at 10 point difference in HDRS from baseline, which resulted in a 22.0% group difference in response.

Please note, for this analysis, the observed values are used and not imputed as in the primary endpoint analysis. Therefore, there may be very slight differences between the results in this analyses and other reported values. This was done for analytical simplicity and has no impact on the interpretation of the results.

In general, the cumulative response shows a better response to the change in HDRS for subjects in the treatment arm versus the control arm, regardless of the MCID (Minimal Clinically Important Difference) chosen.

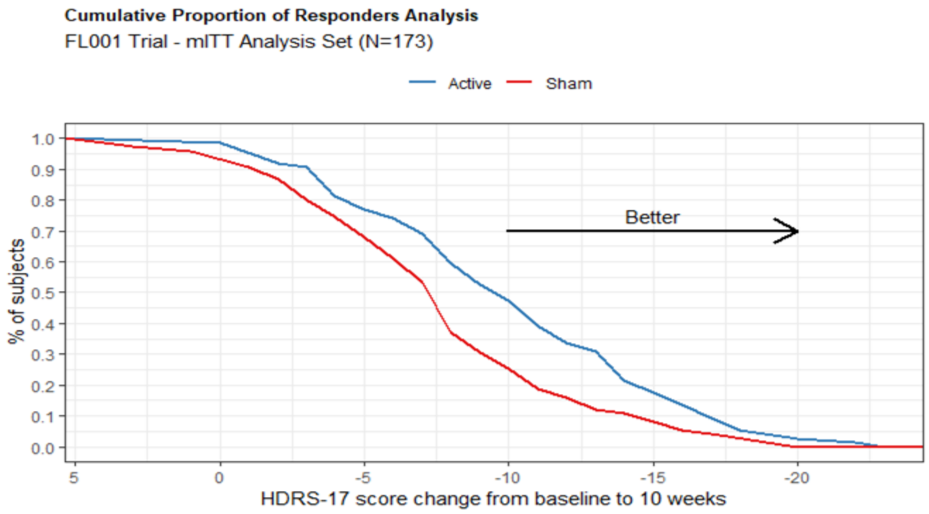

HDRS-17 Score Change	-3	-5	-10	-15	-20
Group Difference	10.6%	9.0%	22.0%	9.6%	2.7%

Supplementary Materials

Home-based transcranial direct current stimulation treatment for major depressive disorder: a fully remote phase 2 randomized sham-controlled trial

Supplementary Figure 11. Cumulative proportion of responders analysis for MADRS scores

A Cumulative Proportion of Responders Analysis (CPRA) was performed per the Statistical Analysis Plan (SAP). The following figure shows:

- X-axis: The change from baseline to Week 10 in MADRS
- Y-axis: The percentage (%) of subjects experiencing a reduction greater than ‘k’ from the baseline in MADRS.
- The blue line is the Active group, and the red line is Sham.

Supplementary Figure 11 shows the percentage of subjects experiencing a response as a function of the change in the MADRS from baseline. For this endpoint, the “more negative” primary endpoint change from baseline indicates a better outcome subject. The observed cumulative percent response in the treatment group is more negative than the control group for all observed MADRS from baseline.

The difference can be further summarized by the table above with cumulative response group difference by specified cut points. The difference between the groups is depicted by an initial separation of the curves with a maximum at 10 point difference in MADRS from baseline, which resulted in a 24.9% group difference in response.

In general, the cumulative response shows a better response to the change in MADRS for subjects in the treatment arm versus the control arm, regardless of the MCID chosen.

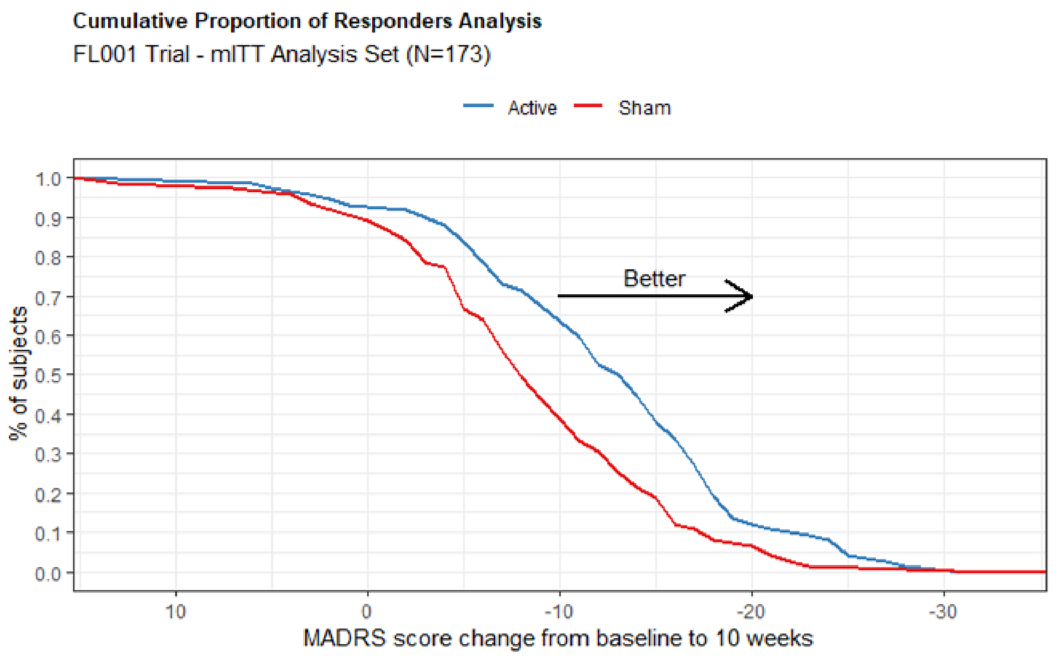

MADRS Score Change	-5	-10	-15	-20
Group Difference	17.1%	24.9%	19.2%	5.5%

Supplementary Materials

Home-based transcranial direct current stimulation treatment for major depressive disorder: a fully remote phase 2 randomized sham-controlled trial

Supplementary Figure 12. Cumulative proportion of responders analysis for MADRS-s scores

A Cumulative Proportion of Responders Analysis (CPRA) was performed per the Statistical Analysis Plan (SAP). The following figure shows:

- X-axis: The change from baseline to Week 10 in MADRS-s
- Y-axis: The percentage (%) of subjects experiencing a reduction greater than ‘k’ from the baseline in MADRS-s.
- The blue line is the Active group, and the red line is Sham.

Supplementary Figure 12 shows the percentage of subjects experiencing a response as a function of the change in the MADRS-s from baseline. For this endpoint, the “more negative” primary endpoint change from baseline indicates a better outcome subject. The observed cumulative percent response in the treatment group is more negative than the control group for all observed MADRS-s from baseline.

The difference can be further summarized by the table above with cumulative response group difference by specified cut points. The difference between the groups is depicted by an initial separation of the curves with a maximum at 10 point difference in MADRS-s from baseline, which resulted in a 23.0% group difference in response.

In general, the cumulative response shows a better response to the change in MADRS-s for subjects in the treatment arm versus the control arm, regardless of the MCID chosen.

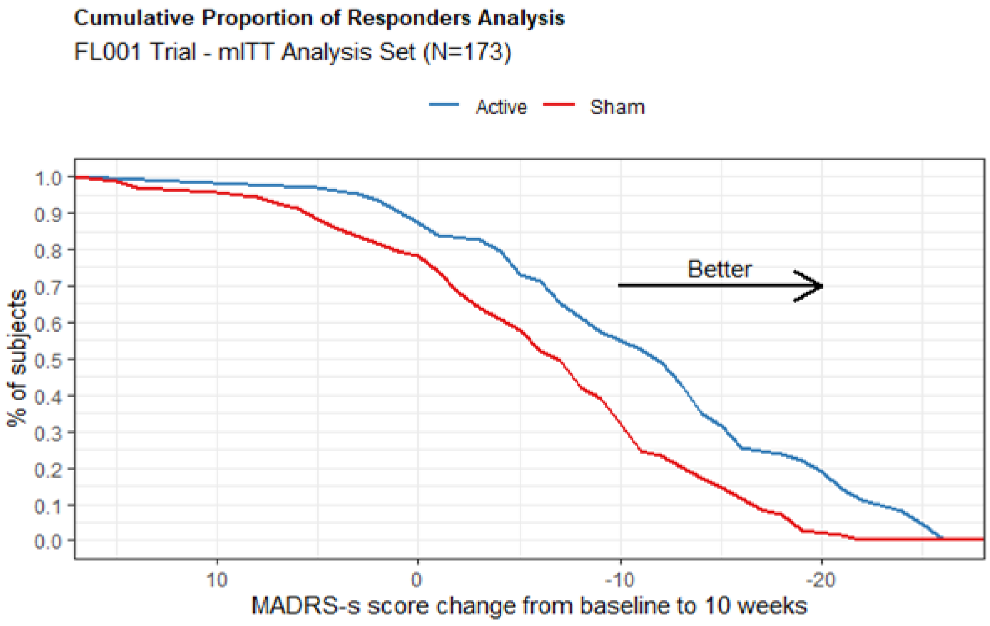

MADRS-s Score Change	-5	-10	-15	-20
Group Difference	15.2%	23.0%	17.4%	17.0%

Supplementary Materials

Home-based transcranial direct current stimulation treatment for major depressive disorder: a fully remote phase 2 randomized sham-controlled trial

Supplementary Table 24. All unanticipated device related adverse events

	Active (N= 87)			Sham (N= 86)			Group Difference			
	Events	Subjs	%	Events	Subjs	%	Δ	LB	UB	P value
All Events	48	30	34.5%	27	21	24.4%	10.1%	-3.7%	23.8%	0.18
Tinnitus	2	2	2.3%	2	2	2.3%	0.0%	-6.2%	6.0%	0.99
Photopsia	2	2	2.3%	1	1	1.2%	1.1%	-4.5%	7.0%	0.99
Vision blurred	2	1	1.1%	0	0	0.0%	1.1%	-3.3%	6.4%	0.99
Nausea	3	2	2.3%	1	1	1.2%	1.1%	-4.5%	7.0%	0.99
Fatigue	1	1	1.1%	0	0	0.0%	1.1%	-3.3%	6.4%	0.99
Malaise	1	1	1.1%	0	0	0.0%	1.1%	-3.3%	6.4%	0.99
Pain	1	1	1.1%	2	2	2.3%	-1.2%	-7.2%	4.4%	0.62
Nasopharyngitis	1	1	1.1%	0	0	0.0%	1.1%	-3.3%	6.4%	0.99
Sinusitis	0	0	0.0%	1	1	1.2%	-1.2%	-6.5%	3.3%	0.50
Burns first degree	2	2	2.3%	0	0	0.0%	2.3%	-2.2%	8.1%	0.50
Burning sensation	1	1	1.1%	0	0	0.0%	1.1%	-3.3%	6.4%	0.99
Dizziness	4	3	3.4%	2	2	2.3%	1.1%	-5.2%	8.0%	0.99
Dysgeusia	0	0	0.0%	1	1	1.2%	-1.2%	-6.5%	3.3%	0.50
Headache	0	0	0.0%	1	1	1.2%	-1.2%	-6.5%	3.3%	0.50
Hyperaesthesia	0	0	0.0%	1	1	1.2%	-1.2%	-6.5%	3.3%	0.49
Migraine	1	1	1.1%	0	0	0.0%	1.1%	-3.3%	6.4%	0.99
Ophthalmic migraine	0	0	0.0%	1	1	1.2%	-1.2%	-6.5%	3.3%	0.50
Paraesthesia	2	2	2.3%	3	3	3.5%	-1.2%	-8.0%	5.1%	0.68
Aggression	0	0	0.0%	1	1	1.2%	-1.2%	-6.5%	3.3%	0.50
Anxiety	1	1	1.1%	1	1	1.2%	0.0%	-5.5%	5.3%	0.99
Depressed mood	1	1	1.1%	0	0	0.0%	1.1%	-3.3%	6.4%	0.99
Insomnia	1	1	1.1%	2	2	2.3%	-1.2%	-7.2%	4.4%	0.62
Panic attack	1	1	1.1%	0	0	0.0%	1.1%	-3.3%	6.4%	0.99
Tension	1	1	1.1%	0	0	0.0%	1.1%	-3.3%	6.4%	0.99
Acne	1	1	1.1%	1	1	1.2%	0.0%	-5.5%	5.3%	0.99
Alopecia	1	1	1.1%	0	0	0.0%	1.1%	-3.3%	6.4%	0.99
Dry skin	9	9	10.3%	4	4	4.7%	5.7%	-2.5%	14.8%	0.25
Pruritus	0	0	0.0%	1	1	1.2%	-1.2%	-6.5%	3.3%	0.50
Rash	1	1	1.1%	1	1	1.2%	0.0%	-5.5%	5.3%	0.99
Skin irritation	7	6	6.9%	0	0	0.0%	6.9%	1.9%	14.5%	0.03
Hot flush	1	1	1.1%	0	0	0.0%	1.1%	-3.3%	6.4%	0.99

An adverse event was present if the participant rated that it was at least possibly associated with the intervention. Participants rated the severity of the adverse events as mild, moderate, or severe, which were assessed by the investigator. P values represent difference between groups assessed by two-sided Fisher exact test. LB, lower bound; UB, upper bound.

Supplementary Materials

Home-based transcranial direct current stimulation treatment for major depressive disorder: a fully remote phase 2 randomized sham-controlled trial

Supplementary Table 25. All unanticipated device related adverse events rated as mild

	Active (N = 87)			Sham (N = 86)			Group Difference			
	**Events**	**Subjs**	**%**	**Events**	**Subjs**	**%**	**Δ**	**LB**	**UB**	**p**
**All Events**	**29**	**21**	**24.1%**	**16**	**14**	**16.3%**	**7.9%**	**-4.5%**	**20.3%**	**0.26**
**Ear and labyrinth disorders**	**2**	**2**	**2.3%**	**1**	**1**	**1.2%**	**1.1%**	**-4.5%**	**7.0%**	**0.99**
Tinnitus	2	2	2.3%	1	1	1.2%	1.1%	-4.5%	7.0%	0.99
**Eye disorders**	**4**	**3**	**3.4%**	**1**	**1**	**1.2%**	**2.3%**	**-3.3%**	**8.9%**	**0.62**
Photopsia	2	2	2.3%	1	1	1.2%	1.1%	-4.5%	7.0%	0.99
Vision blurred	2	1	1.1%	0	0	0.0%	1.1%	-3.3%	6.4%	0.99
**Gastrointestinal disorders**	**3**	**2**	**2.3%**	**1**	**1**	**1.2%**	**1.1%**	**-4.5%**	**7.0%**	**0.99**
Nausea	3	2	2.3%	1	1	1.2%	1.1%	-4.5%	7.0%	0.99
**Injury, poisoning and procedural complications**	**1**	**1**	**1.1%**	**0**	**0**	**0.0%**	**1.1%**	**-3.3%**	**6.4%**	**0.99**
Burns first degree	1	1	1.1%	0	0	0.0%	1.1%	-3.3%	6.4%	0.99
**Nervous system disorders**	**6**	**5**	**5.7%**	**6**	**6**	**7.0%**	**-1.2%**	**-9.6%**	**6.8%**	**0.77**
Burning sensation	1	1	1.1%	0	0	0.0%	1.1%	-3.3%	6.4%	0.99
Dizziness	4	3	3.4%	0	0	0.0%	3.4%	-1.0%	9.8%	0.25
Dysgeusia	0	0	0.0%	1	1	1.2%	-1.2%	-6.5%	3.3%	0.50
Headache	0	0	0.0%	1	1	1.2%	-1.2%	-6.5%	3.3%	0.50
Hyperaesthesia	0	0	0.0%	1	1	1.2%	-1.2%	-6.5%	3.3%	0.50
Ophthalmic migraine	0	0	0.0%	1	1	1.2%	-1.2%	-6.5%	3.3%	0.50
Paraesthesia	1	1	1.1%	2	2	2.3%	-1.2%	-7.2%	4.4%	0.62
**Psychiatric disorders**	**2**	**1**	**1.1%**	**2**	**2**	**2.3%**	**-1.2%**	**-7.2%**	**4.4%**	**0.62**
Aggression	0	0	0.0%	1	1	1.2%	-1.2%	-6.5%	3.3%	0.50
Anxiety	0	0	0.0%	1	1	1.2%	-1.2%	-6.5%	3.3%	0.50
Depressed mood	1	1	1.1%	0	0	0.0%	1.1%	-3.3%	6.4%	0.99
Tension	1	1	1.1%	0	0	0.0%	1.1%	-3.3%	6.4%	0.99
**Skin and subcutaneous tissue disorders**	**10**	**10**	**11.5%**	**5**	**5**	**5.8%**	**5.7%**	**-3.3%**	**15.0%**	**0.28**
Acne	0	0	0.0%	1	1	1.2%	-1.2%	-6.5%	3.3%	0.50
Dry skin	4	4	4.6%	4	4	4.7%	-0.1%	-7.5%	7.3%	0.99
Rash	1	1	1.1%	0	0	0.0%	1.1%	-3.3%	6.4%	0.99
Skin irritation	5	5	5.7%	0	0	0.0%	5.7%	1.0%	12.9%	0.06
**Vascular disorders**	**1**	**1**	**1.1%**	**0**	**0**	**0.0%**	**1.1%**	**-3.3%**	**6.4%**	**0.99**
Hot flush	1	1	1.1%	0	0	0.0%	1.1%	-3.3%	6.4%	0.99

An adverse event was present if the participant rated that it was at least possibly associated with the intervention. Participants rated the severity of the adverse events as mild, moderate, or severe, which were assessed by the investigator. P values represent difference between groups assessed by two-sided Fisher exact test. LB, lower bound; UB, upper bound.

Supplementary Materials

Home-based transcranial direct current stimulation treatment for major depressive disorder: a fully remote phase 2 randomized sham-controlled trial

Supplementary Table 26. All unanticipated device related adverse events rated as moderate

	Active (N= 87)			Sham (N= 86)			Group Difference			
	Events	Subjs	%	Events	Subjs	%	Δ	LB	UB	p
**All Events**	**16**	**13**	**14.9%**	**10**	**8**	**9.3%**	**5.6%**	**-4.5%**	**16.1%**	**0.35**
**Ear and labyrinth disorders**	**0**	**0**	**0.0%**	**1**	**1**	**1.2%**	**-1.2%**	**-6.5%**	**3.3%**	**0.50**
Tinnitus	0	0	0.0%	1	1	1.2%	-1.2%	-6.5%	3.3%	0.50
**General disorders and administration site conditions**	**3**	**3**	**3.4%**	**2**	**2**	**2.3%**	**1.1%**	**-5.2%**	**8.0%**	**0.99**
Fatigue	1	1	1.1%	0	0	0.0%	1.1%	-3.3%	6.4%	0.99
Malaise	1	1	1.1%	0	0	0.0%	1.1%	-3.3%	6.4%	0.99
Pain	1	1	1.1%	2	2	2.3%	-1.2%	-7.2%	4.4%	0.62
**Infections and infestations**	**1**	**1**	**1.1%**	**1**	**1**	**1.2%**	**0.0%**	**-5.5%**	**5.3%**	**0.99**
Nasopharyngitis	1	1	1.1%	0	0	0.0%	1.1%	-3.3%	6.4%	0.99
Sinusitis	0	0	0.0%	1	1	1.2%	-1.2%	-6.5%	3.3%	0.50
**Injury, poisoning and procedural complications**	**1**	**1**	**1.1%**	**0**	**0**	**0.0%**	**1.1%**	**-3.3%**	**6.4%**	**0.99**
Burns first degree	1	1	1.1%	0	0	0.0%	1.1%	-3.3%	6.4%	0.99
**Nervous system disorders**	**1**	**1**	**1.1%**	**2**	**2**	**2.3%**	**-1.2%**	**-7.2%**	**4.4%**	**0.62**
Dizziness	0	0	0.0%	1	1	1.2%	-1.2%	-6.5%	3.3%	0.50
Paraesthesia	1	1	1.1%	1	1	1.2%	0.0%	-5.5%	5.3%	0.99
**Psychiatric disorders**	**3**	**3**	**3.4%**	**2**	**2**	**2.3%**	**1.1%**	**-5.2%**	**8.0%**	**0.99**
Anxiety	1	1	1.1%	0	0	0.0%	1.1%	-3.3%	6.4%	0.99
Insomnia	1	1	1.1%	2	2	2.3%	-1.2%	-7.2%	4.4%	0.62
Panic attack	1	1	1.1%	0	0	0.0%	1.1%	-3.3%	6.4%	0.99
**Skin and subcutaneous tissue disorders**	**7**	**6**	**6.9%**	**2**	**2**	**2.3%**	**4.6%**	**-2.2%**	**12.5%**	**0.28**
Acne	1	1	1.1%	0	0	0.0%	1.1%	-3.3%	6.4%	0.99
Alopecia	1	1	1.1%	0	0	0.0%	1.1%	-3.3%	6.4%	0.99
Dry skin	4	4	4.6%	0	0	0.0%	4.6%	0.1%	11.4%	0.12
Pruritus	0	0	0.0%	1	1	1.2%	-1.2%	-6.5%	3.3%	0.50
Rash	0	0	0.0%	1	1	1.2%	-1.2%	-6.5%	3.3%	0.50
Skin irritation	1	1	1.1%	0	0	0.0%	1.1%	-3.3%	6.4%	0.99

An adverse event was present if the participant rated that it was at least possibly associated with the intervention. Participants rated the severity of the adverse events as mild, moderate, or severe, which were assessed by the investigator. P values represent difference between groups assessed by two-sided Fisher exact test. LB, lower bound; UB, upper bound.

Supplementary Materials

Home-based transcranial direct current stimulation treatment for major depressive disorder: a fully remote phase 2 randomized sham-controlled trial

Supplementary Table 27. All unanticipated device related adverse events rated as severe

	Active (N= 87)			Sham (N= 86)			Group Difference			
	Events	Subjs	%	Events	Subjs	%	Δ	LB	UB	p
**All Events**	**3**	**3**	**3.4%**	**1**	**1**	**1.2%**	**2.3%**	**-3.3%**	**8.9%**	**0.62**
**Nervous system disorders**	**1**	**1**	**1.1%**	**1**	**1**	**1.2%**	**0.0%**	**-5.5%**	**5.3%**	**0.99**
Dizziness	0	0	0.0%	1	1	1.2%	-1.2%	-6.5%	3.3%	0.50
Migraine	1	1	1.1%	0	0	0.0%	1.1%	-3.3%	6.4%	0.99
**Skin and subcutaneous tissue disorders**	**2**	**2**	**2.3%**	**0**	**0**	**0.0%**	**2.3%**	**-2.2%**	**8.1%**	**0.50**
Dry skin	1	1	1.1%	0	0	0.0%	1.1%	-3.3%	6.4%	0.99
Skin irritation	1	1	1.1%	0	0	0.0%	1.1%	-3.3%	6.4%	0.99

An adverse event was present if the participant rated that it was at least possibly associated with the intervention. Participants rated the severity of the adverse events as mild, moderate, or severe, which were assessed by the investigator. P values represent difference between groups assessed by two-sided Fisher exact test. LB, lower bound; UB, upper bound.

Supplementary Materials

Home-based transcranial direct current stimulation treatment for major depressive disorder: a fully remote phase 2 randomized sham-controlled trial

Supplementary Table 28. All unanticipated device related adverse events by days post randomisation

	Days Post-randomization																			
	Missing		<0		0-7		7-14		14-28		28-42		42-56		56-70		>70		Total	
	A	S	A	S	A	S	A	S	A	S	A	S	A	S	A	S	A	S	A	S
**All Events**	**0**	**1**	**0**	**0**	**18**	**7**	**3**	**2**	**9**	**6**	**8**	**1**	**4**	**6**	**4**	**2**	**2**	**2**	**48**	**27**
**Ear and labyrinth disorders**	**0**	**0**	**0**	**0**	**0**	**0**	**0**	**0**	**0**	**2**	**0**	**0**	**0**	**0**	**1**	**0**	**1**	**0**	**2**	**2**
Tinnitus	0	0	0	0	0	0	0	0	0	2	0	0	0	0	1	0	1	0	2	2
**Eye disorders**	**0**	**1**	**0**	**0**	**1**	**0**	**1**	**0**	**1**	**0**	**1**	**0**	**0**	**0**	**0**	**0**	**0**	**0**	**4**	**1**
Photopsia	0	1	0	0	1	0	0	0	0	0	1	0	0	0	0	0	0	0	2	1
Vision blurred	0	0	0	0	0	0	1	0	1	0	0	0	0	0	0	0	0	0	2	0
**Gastrointestinal disorders**	**0**	**0**	**0**	**0**	**1**	**0**	**0**	**0**	**2**	**0**	**0**	**0**	**0**	**1**	**0**	**0**	**0**	**0**	**3**	**1**
Nausea	0	0	0	0	1	0	0	0	2	0	0	0	0	1	0	0	0	0	3	1
**General disorders and administration site conditions**	**0**	**0**	**0**	**0**	**2**	**0**	**0**	**0**	**0**	**1**	**0**	**0**	**0**	**1**	**0**	**0**	**1**	**0**	**3**	**2**
Fatigue	0	0	0	0	1	0	0	0	0	0	0	0	0	0	0	0	0	0	1	0
Malaise	0	0	0	0	0	0	0	0	0	0	0	0	0	0	0	0	1	0	1	0
Pain	0	0	0	0	1	0	0	0	0	1	0	0	0	1	0	0	0	0	1	2
**Infections and infestations**	**0**	**0**	**0**	**0**	**0**	**0**	**0**	**0**	**1**	**1**	**0**	**0**	**0**	**0**	**0**	**0**	**0**	**0**	**1**	**1**
Nasopharyngitis	0	0	0	0	0	0	0	0	1	0	0	0	0	0	0	0	0	0	1	0
Sinusitis	0	0	0	0	0	0	0	0	0	1	0	0	0	0	0	0	0	0	0	1
**Injury, poisoning and procedural complications**	**0**	**0**	**0**	**0**	**0**	**0**	**0**	**0**	**1**	**0**	**1**	**0**	**0**	**0**	**0**	**0**	**0**	**0**	**2**	**0**
Burns first degree	0	0	0	0	0	0	0	0	1	0	1	0	0	0	0	0	0	0	2	0
**Nervous system disorders**	**0**	**0**	**0**	**0**	**5**	**5**	**1**	**0**	**0**	**0**	**0**	**0**	**1**	**3**	**1**	**1**	**0**	**0**	**8**	**9**
Burning sensation	0	0	0	0	0	0	0	0	0	0	0	0	1	0	0	0	0	0	1	0
Dizziness	0	0	0	0	3	1	0	0	0	0	0	0	0	1	1	0	0	0	4	2
Dysgeusia	0	0	0	0	0	1	0	0	0	0	0	0	0	0	0	0	0	0	0	1
Headache	0	0	0	0	0	0	0	0	0	0	0	0	0	0	0	1	0	0	0	1
Hyperaesthesia	0	0	0	0	0	0	0	0	0	0	0	0	0	1	0	0	0	0	0	1
Migraine	0	0	0	0	0	0	1	0	0	0	0	0	0	0	0	0	0	0	1	0
Ophthalmic migraine	0	0	0	0	0	1	0	0	0	0	0	0	0	0	0	0	0	0	0	1
Paraesthesia	0	0	0	0	2	2	0	0	0	0	0	0	0	1	0	0	0	0	2	3
**Psychiatric disorders**	**0**	**0**	**0**	**0**	**4**	**0**	**1**	**1**	**0**	**1**	**0**	**1**	**0**	**0**	**0**	**0**	**0**	**1**	**5**	**4**
Aggression	0	0	0	0	0	0	0	0	0	1	0	0	0	0	0	0	0	0	0	1
Anxiety	0	0	0	0	1	0	0	1	0	0	0	0	0	0	0	0	0	0	1	1
Depressed mood	0	0	0	0	1	0	0	0	0	0	0	0	0	0	0	0	0	0	1	0
Insomnia	0	0	0	0	0	0	1	0	0	0	0	1	0	0	0	0	0	1	1	2
Panic attack	0	0	0	0	1	0	0	0	0	0	0	0	0	0	0	0	0	0	1	0
Tension	0	0	0	0	1	0	0	0	0	0	0	0	0	0	0	0	0	0	1	0
**Skin and subcutaneous tissue disorders**	**0**	**0**	**0**	**0**	**5**	**2**	**0**	**1**	**3**	**1**	**6**	**0**	**3**	**1**	**2**	**1**	**0**	**1**	**19**	**7**
Acne	0	0	0	0	0	1	0	0	0	0	0	0	1	0	0	0	0	0	1	1
Alopecia	0	0	0	0	0	0	0	0	0	0	0	0	1	0	0	0	0	0	1	0
Dry skin	0	0	0	0	1	1	0	0	2	0	5	0	0	1	1	1	0	1	9	4
Pruritus	0	0	0	0	0	0	0	1	0	0	0	0	0	0	0	0	0	0	0	1
Rash	0	0	0	0	0	0	0	0	0	1	1	0	0	0	0	0	0	0	1	1
Skin irritation	0	0	0	0	4	0	0	0	1	0	0	0	1	0	1	0	0	0	7	0
**Vascular disorders**	**0**	**0**	**0**	**0**	**0**	**0**	**0**	**0**	**1**	**0**	**0**	**0**	**0**	**0**	**0**	**0**	**0**	**0**	**1**	**0**
Hot flush	0	0	0	0	0	0	0	0	1	0	0	0	0	0	0	0	0	0	1	0

The number of events is presented for active and shame groups (A = active, S = sham). An adverse event was present if the participant rated that it was at least possibly associated with the intervention.

Supplementary Materials

Home-based transcranial direct current stimulation treatment for major depressive disorder: a fully remote phase 2 randomized sham-controlled trial

Supplementary Table 29. All unanticipated adverse events

	Active (N= 87)			Sham (N= 86)			Group Difference			
	Events	Subjs	%	Events	Subjs	%	Δ	LB	UB	p
**All Events**	**90**	**53**	**60.9%**	**55**	**37**	**43.0%**	**17.9%**	**2.0%**	**32.5%**	**0.02**
**Blood and lymphatic system disorders**	**1**	**1**	**1.1%**	**0**	**0**	**0.0%**	**1.1%**	**-3.3%**	**6.4%**	**0.99**
Lymphadenopathy	1	1	1.1%	0	0	0.0%	1.1%	-3.3%	6.4%	0.99
**Ear and labyrinth disorders**	**2**	**2**	**2.3%**	**2**	**2**	**2.3%**	**0.0%**	**-6.2%**	**6.0%**	**0.99**
Tinnitus	2	2	2.3%	2	2	2.3%	0.0%	-6.2%	6.0%	0.99
**Eye disorders**	**4**	**3**	**3.4%**	**1**	**1**	**1.2%**	**2.3%**	**-3.3%**	**8.9%**	**0.62**
Photopsia	2	2	2.3%	1	1	1.2%	1.1%	-4.5%	7.0%	0.99
Vision blurred	2	1	1.1%	0	0	0.0%	1.1%	-3.3%	6.4%	0.99
**Gastrointestinal disorders**	**8**	**6**	**6.9%**	**1**	**1**	**1.2%**	**5.7%**	**-0.4%**	**13.6%**	**0.12**
Diarrhoea	1	1	1.1%	0	0	0.0%	1.1%	-3.3%	6.4%	0.99
Irritable bowel syndrome	2	2	2.3%	0	0	0.0%	2.3%	-2.2%	8.1%	0.50
Nausea	3	2	2.3%	1	1	1.2%	1.1%	-4.5%	7.0%	0.99
Vomiting	2	1	1.1%	0	0	0.0%	1.1%	-3.3%	6.4%	0.99
**General disorders and administration site conditions**	**3**	**3**	**3.4%**	**3**	**3**	**3.5%**	**0.0%**	**-6.8%**	**6.8%**	**0.99**
Chest pain	0	0	0.0%	1	1	1.2%	-1.2%	-6.5%	3.3%	0.50
Fatigue	1	1	1.1%	0	0	0.0%	1.1%	-3.3%	6.4%	0.99
Malaise	1	1	1.1%	0	0	0.0%	1.1%	-3.3%	6.4%	0.99
Pain	1	1	1.1%	2	2	2.3%	-1.2%	-7.2%	4.4%	0.62
**Infections and infestations**	**25**	**22**	**25.3%**	**25**	**19**	**22.1%**	**3.2%**	**-9.8%**	**16.1%**	**0.72**
Bacterial infection	0	0	0.0%	1	1	1.2%	-1.2%	-6.5%	3.3%	0.50
COVID-19	6	6	6.9%	5	5	5.8%	1.1%	-7.0%	9.3%	0.99
Cystitis	1	1	1.1%	0	0	0.0%	1.1%	-3.3%	6.4%	0.99
Gastroenteritis viral	1	1	1.1%	1	1	1.2%	0.0%	-5.5%	5.3%	0.99
Influenza	4	3	3.4%	6	6	7.0%	-3.5%	-11.5%	3.7%	0.33
Lower respiratory tract infection	1	1	1.1%	1	1	1.2%	0.0%	-5.5%	5.3%	0.99
Nasopharyngitis	12	11	12.6%	8	7	8.1%	4.5%	-5.1%	14.5%	0.46
Pneumonia	0	0	0.0%	1	1	1.2%	-1.2%	-6.5%	3.3%	0.50

Supplementary Materials

Home-based transcranial direct current stimulation treatment for major depressive disorder: a fully remote phase 2 randomized sham-controlled trial

Sinusitis	0	0	0.0%	1	1	1.2%	-1.2%	-6.5%	3.3%	0.50
Viral infection	0	0	0.0%	1	1	1.2%	-1.2%	-6.5%	3.3%	0.50
**Injury, poisoning and procedural complications**	**4**	**4**	**4.6%**	**0**	**0**	**0.0%**	**4.6%**	**0.1%**	**11.4%**	**0.12**
Burns first degree	2	2	2.3%	0	0	0.0%	2.3%	-2.2%	8.1%	0.50
Head injury	1	1	1.1%	0	0	0.0%	1.1%	-3.3%	6.4%	0.99
Limb injury	1	1	1.1%	0	0	0.0%	1.1%	-3.3%	6.4%	0.99
**Metabolism and nutrition disorders**	**1**	**1**	**1.1%**	**0**	**0**	**0.0%**	**1.1%**	**-3.3%**	**6.4%**	**0.99**
Gout	1	1	1.1%	0	0	0.0%	1.1%	-3.3%	6.4%	0.99
**Neoplasms benign, malignant and unspecified (incl cysts and polyps)**	**1**	**1**	**1.1%**	**0**	**0**	**0.0%**	**1.1%**	**-3.3%**	**6.4%**	**0.99**
Acrochordon	1	1	1.1%	0	0	0.0%	1.1%	-3.3%	6.4%	0.99
**Nervous system disorders**	**13**	**9**	**10.3%**	**9**	**8**	**9.3%**	**1.0%**	**-8.5%**	**10.6%**	**0.99**
Burning sensation	1	1	1.1%	0	0	0.0%	1.1%	-3.3%	6.4%	0.99
Dizziness	5	3	3.4%	2	2	2.3%	1.1%	-5.2%	8.0%	0.99
Dysgeusia	0	0	0.0%	1	1	1.2%	-1.2%	-6.5%	3.3%	0.50
Headache	0	0	0.0%	1	1	1.2%	-1.2%	-6.5%	3.3%	0.50
Hyperaesthesia	0	0	0.0%	1	1	1.2%	-1.2%	-6.5%	3.3%	0.50
Migraine	4	3	3.4%	0	0	0.0%	3.4%	-1.0%	9.8%	0.25
Migraine with aura	1	1	1.1%	0	0	0.0%	1.1%	-3.3%	6.4%	0.99
Ophthalmic migraine	0	0	0.0%	1	1	1.2%	-1.2%	-6.5%	3.3%	0.50
Paraesthesia	2	2	2.3%	3	3	3.5%	-1.2%	-8.0%	5.1%	0.68
**Psychiatric disorders**	**5**	**4**	**4.6%**	**4**	**4**	**4.7%**	**-0.1%**	**-7.5%**	**7.3%**	**0.99**
Aggression	0	0	0.0%	1	1	1.2%	-1.2%	-6.5%	3.3%	0.50
Anxiety	1	1	1.1%	1	1	1.2%	0.0%	-5.5%	5.3%	0.99
Depressed mood	1	1	1.1%	0	0	0.0%	1.1%	-3.3%	6.4%	0.99
Insomnia	1	1	1.1%	2	2	2.3%	-1.2%	-7.2%	4.4%	0.62
Panic attack	1	1	1.1%	0	0	0.0%	1.1%	-3.3%	6.4%	0.99
Tension	1	1	1.1%	0	0	0.0%	1.1%	-3.3%	6.4%	0.99
**Reproductive system and breast disorders**	**0**	**0**	**0.0%**	**1**	**1**	**1.2%**	**-1.2%**	**-6.5%**	**3.3%**	**0.50**
Heavy menstrual bleeding	0	0	0.0%	1	1	1.2%	-1.2%	-6.5%	3.3%	0.50
**Respiratory, thoracic and mediastinal disorders**	**1**	**1**	**1.1%**	**2**	**1**	**1.2%**	**0.0%**	**-5.5%**	**5.3%**	**0.99**
Asthma	0	0	0.0%	1	1	1.2%	-1.2%	-6.5%	3.3%	0.50

Dyspnoea	0	0	0.0%	1	1	1.2%	-1.2%	-6.5%	3.3%	0.50
Oropharyngeal pain	1	1	1.1%	0	0	0.0%	1.1%	-3.3%	6.4%	0.99
**Skin and subcutaneous tissue disorders**	**19**	**17**	**19.5%**	**7**	**7**	**8.1%**	**11.4%**	**1.0%**	**22.3%**	**0.05**
Acne	1	1	1.1%	1	1	1.2%	0.0%	-5.5%	5.3%	0.99
Alopecia	1	1	1.1%	0	0	0.0%	1.1%	-3.3%	6.4%	0.99
Dry skin	9	9	10.3%	4	4	4.7%	5.7%	-2.5%	14.8%	0.25
Pruritus	0	0	0.0%	1	1	1.2%	-1.2%	-6.5%	3.3%	0.50
Rash	1	1	1.1%	1	1	1.2%	0.0%	-5.5%	5.3%	0.99
Skin irritation	7	6	6.9%	0	0	0.0%	6.9%	1.9%	14.5%	0.03
**Vascular disorders**	**3**	**2**	**2.3%**	**0**	**0**	**0.0%**	**2.3%**	**-2.2%**	**8.1%**	**0.50**
Hot flush	1	1	1.1%	0	0	0.0%	1.1%	-3.3%	6.4%	0.99
Hypertension	2	1	1.1%	0	0	0.0%	1.1%	-3.3%	6.4%	0.99

All unanticipated adverse events that were reported up to week 10 including those that weren't associated with the intervention. Participants rated the severity of the adverse events as mild, moderate, or severe, which were assessed by the investigator. P values represent difference between groups assessed by two-sided Fisher exact test. LB, lower bound; UB, upper bound.

Supplementary Materials

Home-based transcranial direct current stimulation treatment for major depressive disorder: a fully remote phase 2 randomized sham-controlled trial

Supplementary Table 30. Response and remission rates at all time points

Outcome	Active tDCS (N=87)	Sham tDCS (N=86)	Odds Ratio (95% Confidence Interval)	P Value
**Week 1**				
**HDRS**				
response	10 (12.5%)	13 (15.3%)	0.79 (0.32 to 1.96)	0.62
remission	4 (4.7%)	6 (6.5%)	0.70 (0.18 to 2.73)	0.66
**MADRS**				
response	10 (12.2%)	8 (9.2%)	1.38 (0.50 to 3.81)	0.54
remission	7 (4.4%)	9 (3.7%)	1.21 (0.36 to 4.08)	0.81
**MADRS-S**				
response	10 (12.2%)	7 (8.3%)	1.45 (0.50 to 4.19)	0.51
remission	11 (8.6%)	9 (5.7%)	1.59 (0.51 to 4.94)	0.52
**Week 4**				
**HDRS**				
response	30 (40.7%)	19 (26.0%)	1.95 (0.98 to 3.91)	0.06
remission	18 (23.8%)	11 (13.6%)	1.99 (0.85 to 4.66)	0.13
**MADRS**				
response	32 (41.4%)	16 (19.6%)	2.90 (1.40 to 6.01)	0.004
remission	25 (32.7%)	11 (12.5%)	3.42 (1.45 to 8.09)	0.004
**MADRS-S**				
response	22 (27.4%)	11 (15.2%)	2.11 (0.93 to 4.81)	0.08
remission	23 (29.3%)	13 (16.2%)	2.15 (0.95 to 4.89)	0.07
**Week 7**				
**HDRS**				
response	36 (48.7%)	29 (37.9%)	1.55 (0.81 to 3.01)	0.20
remission	27 (37.1%)	15 (20.5%)	2.30 (1.05 to 5.08)	0.04
**MADRS**				
response	40 (53.7%)	26 (33.0%)	2.36 (1.20 to 4.65)	0.013
remission	34 (47.7%)	19 (21.8%)	3.28 (1.49 to 7.20)	0.002
**MADRS-S**				
response	29 (39.8%)	15 (23.1%)	2.21 (1.06 to 4.63)	0.035
remission	24 (33.2%)	16 (21.5%)	1.81 (0.83 to 3.97)	0.14
**Week 10**				
**HDRS**				
response	44 (58.3%)	29 (37.8%)	2.31 (1.17 to 4.55)	0.017
remission	34 (44.9%)	17 (21.8%)	2.93 (1.41 to 6.09)	0.004
**MADRS**				
response	47 (64.2%)	26 (32.3%)	3.76 (1.83 to 7.74)	0.0002
remission	42 (57.5%)	25 (29.4%)	3.26 (1.53 to 6.94)	0.002
**MADRS-S**				
response	32 (51.8%)	15 (25.1%)	3.22 (1.50 to 6.94)	0.002
remission	32 (53.8%)	18 (23.4%)	3.83 (1.61 to 9.13)	0.002

tDCS, transcranial direct current stimulation; HDRS, 17-item Hamilton Depression Rating Scale;[2] MADRS, Montgomery-Åsberg Depression Rating Scale;[11] MADRS-s, Montgomery-Åsberg Depression Rating Scale-self report.[6] Response defined as decrease (indicating fewer depressive symptoms) of 50% or more in total scores from baseline. Remission defined as score of 7 or fewer points in HDRS or as 10 or fewer points in MADRS or as 12 or fewer points in MADRS-s at 10 weeks. Percentages estimated based on odds ratios. HDRS scores range from 0 to 52; MADRS scores range from 0 to 60; MADRS-s scores range from 0 to 54, with higher scores indicating increased depressive symptom severity. P values represent difference between groups. Fully Conditional Specification (FCS) approach was used to produce 20 multiply imputed completed data sets. The FCS approach accommodates nonmonotonicity in the pattern of missing data and requires regression models to be specified for each variable with missing values needing imputation. All models included age, sex, in psychotherapy at baseline, use of any antidepressants at baseline and treatment group. The resulting completed datasets were combined using Rubin's Rules.

Supplementary Materials

Home-based transcranial direct current stimulation treatment for major depressive disorder: a fully remote phase 2 randomized sham-controlled trial

Supplementary Table 31. Changes in depressive severity as measured by HDRS, MADRS and MADRS-s and quality of life as measured by EQ-5D-3L for ITT analysis

Outcome	Active (n = 87)	Sham (n = 87)	Difference or Odds Ratio (95% CI)	P value
HDRS				
Decrease in score	9.40 ± 6.25	7.11 ± 6.00	2.29 (0.58 to 4.00)	0.009
Clinical response	41 (54.6%)	21 (28.1%)	3.09 (1.55 to 6.17)	0.001
Clinical remission	34 (45.1%)	17 (22.0%)	2.92 (1.43 to 5.97)	0.003
MADRS				
Decrease in score	11.29 ± 8.84	7.68 ± 8.32	3.60 (1.16 to 6.05)	0.004
Clinical response	46 (63.4%)	25 (32.3%)	3.64 (1.79 to 7.40)	<0.001
Clinical remission	42 (57.7%)	25 (30.3%)	3.14 (1.48 to 6.68)	0.002
MADRS-s				
Decrease in score	9.86 ± 9.01	6.13 ± 9.24	3.73 (1.00 to 6.45)	0.008
Clinical response	30 (49.1%)	14 (22.3%)	3.37 (1.52 to 7.44)	0.002
Clinical remission	32 (54.2%)	18 (22.7%)	4.04 (1.69 to 9.66)	<0.001
EQ-5D-3L				
Change in score	0.07 ± 0.15	0.07 ± 0.17	0.02 (-0.02 to 0.06)	0.32

HDRS, 17-item Hamilton Depression Rating Scale;[2] MADRS, Montgomery-Åsberg Depression Rating Scale;[11] MADRS-s, Montgomery-Åsberg Depression Rating Scale-self report;[6] EQ-5D-3L, quality of life measure;[15–17] CI, confidence interval. Mean values are presented with '±' standard deviation values. HDRS, MADRS, MADRS-s change ratings are the change in total ratings from baseline to week 10. Between-group differences are shown for the changes in scores from baseline to week 10, and odds ratios are shown for the outcomes for clinical response and remission. Percentages for clinical response and remission outcomes are estimated based on odds ratios. HDRS scores range from 0 to 52; MADRS scores range from 0 to 60; MADRS-s scores range from 0 to 54, with higher scores indicating increased depressive symptom severity. Clinical response was defined as a decrease in the score (indicating less depressive severity) of more than 50% from baseline to week 10. Clinical remission was defined as: HDRS score of 7 or less; MADRS score of 10 or less; MADRS-s score of 12 or less. Fully Conditional Specification (FCS) approach was used to produce 20 multiply imputed completed data sets. The FCS approach accommodates nonmonotonicity in the pattern of missing data and requires regression models to be specified for each variable with missing values needing imputation. All models included age, sex, in psychotherapy at baseline, use of any antidepressants at baseline and treatment group. The resulting completed datasets were combined using Rubin's Rules.

Supplementary Materials

Home-based transcranial direct current stimulation treatment for major depressive disorder: a fully remote phase 2 randomized sham-controlled trial

Supplementary Table 32. Changes in depressive severity as measured by Hamilton Depression Rating Scale using last observation carried forward for missing data, mITT analysis.

Outcome	Active (n = 87)	Sham (n = 86)	Difference	P value
Decrease in HDRS score	10.24 ± 5.34	7.76 ± 5.00	2.08 (0.37 to 3.80)	0.018

HDRS, 17-item Hamilton Depression Rating Scale;[2] HDRS change ratings are the change in total ratings from baseline to week 10. HDRS scores range from 0 to 52, with higher scores indicating increased depressive symptom severity.

Supplementary Materials

Home-based transcranial direct current stimulation treatment for major depressive disorder: a fully remote phase 2 randomized sham-controlled trial

Supplementary Table 33. Response rates as measured by HDRS, MADRS and MADRS-s using response definition as a reduction in score at week 10 of >50% from baseline

Measure	Active (n = 87)	Sham (n = 86)	Odds Ratio (95% CI)	NNT	P value
**Secondary Outcomes**					
HDRS Clinical response	41 (54.4%)	21 (26.9%)	3.25 (1.57 to 6.74)	4	0.001
MADRS Clinical response	46 (63.0%)	25 (31.6%)	3.70 (1.82 to 7.52)	4	0.0002
MADRS-s Clinical response	30 (49.1%)	14 (24.0%)	3.06 (1.43 to 6.56)	4	0.004

HDRS, 17-item Hamilton Depression Rating Scale;[2] MADRS, Montgomery-Åsberg Depression Rating Scale;[11] MADRS-s, Montgomery-Åsberg Depression Rating Scale-self report;[6] Percentages for clinical response and remission outcomes are estimated based on odds ratios. Clinical response was defined as a decrease in the score (indicating less depressive symptom severity) of more than 50% from baseline to week 10. Fully Conditional Specification (FCS) approach was used to produce 20 multiply imputed completed data sets. The FCS approach accommodates nonmonotonicity in the pattern of missing data and requires regression models to be specified for each variable with missing values needing imputation. All models included age, sex, in psychotherapy at baseline, use of any antidepressants at baseline and treatment group. The resulting completed datasets were combined using Rubin's Rules.

Supplementary Materials

Home-based transcranial direct current stimulation treatment for major depressive disorder: a fully remote phase 2 randomized sham-controlled trial

Supplementary Table 34. Changes in depressive severity as measured by HDRS, MADRS and MADRS-s and quality of life as measured by EQ-5D-3L for participants at the UK site at 10 weeks

Outcome	Active (n = 58)	Sham (n = 57)	Difference or Odds Ratio (95% CI)	P value
HDRS				
Decrease in score	8.00 ± 5.56	5.94 ± 5.49	2.06 (0.2 to 3.9)	0.03
Clinical response	24 (47.2%)	11 (20.0%)	3.57 (1.46 to 8.71)	0.004
Clinical remission	18 (34.8%)	7 (12.5%)	3.73 (1.34 to 10.34)	0.01
MADRS				
Decrease in score	11.14 ± 8.91	7.64 ± 8.61	3.49 (0.5 to 6.5)	0.02
Clinical response	28 (54.9%)	16 (31.6%)	2.63 (1.14 to 6.08)	0.02
Clinical remission	25 (48.5%)	14 (26.1%)	2.67 (1.13 to 6.34)	0.03
MADRS-s				
Decrease in score	9.12 ± 8.87	5.58 ± 9.46	3.53 (0.3 to 6.8)	0.04
Clinical response	16 (41.2%)	7 (14.7%)	4.06 (1.45 to 11.42)	0.006
Clinical remission	17 (43.2%)	10 (15.3%)	4.22 (1.44 to 12.39)	0.008
EQ-5D-3L				
Change in score	0.07 ± 0.17	0.05 ± 0.15	0.04 (0.00 to 0.09)	0.08

HDRS, 17-item Hamilton Depression Rating Scale;[2] MADRS, Montgomery-Åsberg Depression Rating Scale;[11] MADRS-s, Montgomery-Åsberg Depression Rating Scale-self report;[6] EQ-5D-3L, quality of life measure;[15–17] CI, confidence interval. Mean values are presented with '±' standard deviation values. HDRS, MADRS, MADRS-s change ratings are the change in total ratings from baseline to week 10. Between-group differences are shown for the changes in scores from baseline to week 10, and odds ratios are shown for the outcomes for clinical response and remission. Percentages for clinical response and remission outcomes are estimated based on odds ratios. HDRS scores range from 0 to 52; MADRS scores range from 0 to 60; MADRS-s scores range from 0 to 54, with higher scores indicating increased depressive symptom severity. Clinical response was defined as a decrease in the score (indicating less depressive severity) of more than 50% from baseline to week 10. Clinical remission was defined as: HDRS score of 7 or less; MADRS score of 10 or less; MADRS-s score of 12 or less. Fully Conditional Specification (FCS) approach was used to produce 20 multiply imputed completed data sets. The FCS approach accommodates nonmonotonicity in the pattern of missing data and requires regression models to be specified for each variable with missing values needing imputation. All models included age, sex, in psychotherapy at baseline, use of any antidepressants at baseline and treatment group. The resulting completed datasets were combined using Rubin's Rules.

Supplementary Materials

Home-based transcranial direct current stimulation treatment for major depressive disorder: a fully remote phase 2 randomized sham-controlled trial

Supplementary Table 35. Changes in depressive severity as measured by HDRS, MADRS and MADRS-s and quality of life as measured by EQ-5D-3L for participants at the US site at 10 weeks

Outcome	Active (n = 29)	Sham (n = 29)	Difference or Odds Ratio (95% CI)	P value
HDRS				
Decrease in score	11.67 ± 7.54	9.46 ± 8.54	2.21 (2.0 to 6.5)	0.30
Clinical response	17 (67.5%)	10 (44.8%)	2.57 (0.73 to 9.04)	0.13
Clinical remission	16 (67.7%)	10 (43.6%)	2.72 (0.75 to 9.86)	0.11
MADRS				
Decrease in score	11.61 ± 9.27	8.24 ± 9.72	3.37 (-1.5 to 8.2)	0.17
Clinical response	18 (77.3%)	9 (37.5%)	5.79 (1.26 to 26.62)	0.01
Clinical remission	17 (76.0%)	11 (41.4%)	4.58 (0.88 to 23.80)	0.05
MADRS-s				
Decrease in score	11.91 ± 11.18	8.82 ± 9.92	3.09 (-2.9 to 9.1)	0.31
Clinical response	14 (65.7%)	7 (43.2%)	2.54 (0.70 to 9.27)	0.16
Clinical remission	15 (73.0%)	8 (42.5%)	3.69 (0.84 to 16.19)	0.07
EQ-5D-3L				
Change in score	0.07 ± 0.12	0.11 ± 0.19	-0.03 (-0.10 to 0.04)	0.38

HDRS, 17-item Hamilton Depression Rating Scale;[2] MADRS, Montgomery-Åsberg Depression Rating Scale;[11] MADRS-s, Montgomery-Åsberg Depression Rating Scale-self report;[6] EQ-5D-3L, quality of life measure;[15–17] CI, confidence interval. Mean values are presented with '±' standard deviation values. HDRS, MADRS, MADRS-s change ratings are the change in total ratings from baseline to week 10. Between-group differences are shown for the changes in scores from baseline to week 10, and odds ratios are shown for the outcomes for clinical response and remission. Percentages for clinical response and remission outcomes are estimated based on odds ratios. HDRS scores range from 0 to 52; MADRS scores range from 0 to 60; MADRS-s scores range from 0 to 54, with higher scores indicating increased depressive symptom severity. Clinical response was defined as a decrease in the score (indicating less depressive severity) of more than 50% from baseline to week 10. Clinical remission was defined as: HDRS score of 7 or less; MADRS score of 10 or less; MADRS-s score of 12 or less. Fully Conditional Specification (FCS) approach was used to produce 20 multiply imputed completed data sets. The FCS approach accommodates nonmonotonicity in the pattern of missing data and requires regression models to be specified for each variable with missing values needing imputation. All models included age, sex, in psychotherapy at baseline, use of any antidepressants at baseline and treatment group. The resulting completed datasets were combined using Rubin's Rules.

Supplementary Materials

Home-based transcranial direct current stimulation treatment for major depressive disorder: a fully remote phase 2 randomized sham-controlled trial

Supplementary Table 36. Changes in depressive severity as measured by HDRS, MADRS and MADRS-s and quality of life as measured by EQ-5D-3L for participants who made the 'guess' that they were receiving the active treatment at 10 weeks

Outcome	Active tDCS (n = 66)	Sham tDCS (n = 48)	Difference or Odds Ratio (95% CI)	P value
HDRS				
Decrease in score	10.30 ± 5.94	7.97 ± 5.76	2.33 (0.23 to 4.44)	0.03
Clinical response	35 (61.1%)	16 (37.1%)	2.66 (1.14 to 6.19)	0.02
Clinical remission	29 (51.2%)	12 (26.5%)	2.91 (1.21 to 7.00)	0.01
MADRS				
Decrease in score	12.55 ± 8.59	8.67 ± 8.22	3.87 (0.83 to 6.92)	0.01
Clinical response	40 (70.2%)	18 (41.0%)	3.40 (1.35 to 8.56)	0.009
Clinical remission	36 (63.4%)	17 (35.7%)	3.10 (1.24 to 7.75)	0.01
MADRS-s				
Decrease in score	11.12 ± 8.47	8.62 ± 8.46	2.50 (-0.70 to 5.69)	0.13
Clinical response	25 (51.9%)	9 (25.7%)	3.12 (1.20 to 8.11)	0.01
Clinical remission	28 (60.2%)	13 (28.7%)	3.78 (1.30 to 11.02)	0.009
EQ-5D-3L				
Change in score	0.08 ± 0.16	0.09 ± 0.15	0.02 (-0.03 to 0.07)	0.38

HDRS, Hamilton Depression Rating Scale;[2] MADRS, Montgomery-Åsberg Depression Rating Scale;[11] MADRS-s, Montgomery-Åsberg Depression Rating Scale-self report;[6] EQ-5D-3L, quality of life measure;[15–17] CI, confidence interval. Mean values are presented with '±' standard deviation values. HDRS, MADRS, MADRS-s change ratings are the change in total ratings from baseline to week 10. Between-group differences are shown for the changes in scores from baseline to week 10, and odds ratios are shown for the outcomes for clinical response and remission. Percentages for clinical response and remission outcomes are estimated based on odds ratios. HDRS scores range from 0 to 52; MADRS scores range from 0 to 60; MADRS-s scores range from 0 to 54, with higher scores indicating increased depressive symptom severity. Clinical response was defined as a decrease in the score (indicating less depressive severity) of more than 50% from baseline to week 10. Clinical remission was defined as: HDRS score of 7 or less; MADRS score of 10 or less; MADRS-s score of 12 or less. Fully Conditional Specification (FCS) approach was used to produce 20 multiply imputed completed data sets. The FCS approach accommodates nonmonotonicity in the pattern of missing data and requires regression models to be specified for each variable with missing values needing imputation. All models included age, sex, in psychotherapy at baseline, use of any antidepressants at baseline and treatment group. The resulting completed datasets were combined using Rubin's Rules.

Supplementary Materials

Home-based transcranial direct current stimulation treatment for major depressive disorder: a fully remote phase 2 randomized sham-controlled trial

Supplementary Table 37. Changes in depressive severity as measured by HDRS, MADRS and MADRS-s and quality of life as measured by EQ-5D-3L for participants who correctly guessed their treatment allocation group

Outcome	Active (n = 66)	Sham (n = 33)	Difference or Odds Ratio (95% CI)	P value
HDRS				
Decrease in score	10.05 ± 6.15	5.90 ± 6.05	4.15 (1.9 to 6.4)	0.0004
Clinical response	35 (60.6%)	5 (12.6%)	10.77 (3.25 to 35.64)	0.0003
Clinical remission	29 (49.5%)	5 (13.2%)	6.45 (2.06 to 20.18)	0.00009
MADRS				
Decrease in score	12.43 ± 9.59	6.50 ± 9.24	5.93 (2.4 to 9.5)	0.001
Clinical response	40 (69.7%)	7 (18.6%)	10.08 (3.35 to 30.28)	0.0000002
Clinical remission	36 (62.2%)	8 (19.9%)	6.63 (2.23 to 19.76)	0.00004
MADRS-s				
Decrease in score	11.16 ± 9.03	3.06 ± 9.02	8.10 (4.5 to 11.7)	0.00002
Clinical response	25 (50.4%)	5 (17.8%)	4.71 (1.51 to 14.66)	0.002
Clinical remission	28 (56.6%)	5 (15.1%)	7.35 (2.15 to 25.09)	0.00009
EQ-5D-3L				
Change in score	0.08 ± 0.16	0.04 ± 0.19	0.05 (-0.01 to 0.11)	0.08

HDRS, 17-item Hamilton Depression Rating Scale;[2] MADRS, Montgomery-Åsberg Depression Rating Scale;[11] MADRS-s, Montgomery-Åsberg Depression Rating Scale-self report;[6] EQ-5D-3L, quality of life measure;[15–17] CI, confidence interval. Mean values are presented with '±' standard deviation values. HDRS, MADRS, MADRS-s change ratings are the change in total ratings from baseline to week 10. Between-group differences are shown for the changes in scores from baseline to week 10, and odds ratios are shown for the outcomes for clinical response and remission. Percentages for clinical response and remission outcomes are estimated based on odds ratios. HDRS scores range from 0 to 52; MADRS scores range from 0 to 60; MADRS-s scores range from 0 to 54, with higher scores indicating increased depressive symptom severity. Clinical response was defined as a decrease in the score (indicating less depressive severity) of more than 50% from baseline to week 10. Clinical remission was defined as: HDRS score of 7 or less; MADRS score of 10 or less; MADRS-s score of 12 or less. Fully Conditional Specification (FCS) approach was used to produce 20 multiply imputed completed data sets. The FCS approach accommodates nonmonotonicity in the pattern of missing data and requires regression models to be specified for each variable with missing values needing imputation. All models included age, sex, in psychotherapy at baseline, use of any antidepressants at baseline and treatment group. The resulting completed datasets were combined using Rubin's Rules.

Supplementary Materials

Home-based transcranial direct current stimulation treatment for major depressive disorder: a fully remote phase 2 randomized sham-controlled trial

Supplementary Table 38. Changes in depressive severity as measured by HDRS, MADRS and MADRS-s and quality of life as measured by EQ-5D-3L for participants who incorrectly guessed their treatment allocation group

Outcome	Active (n = 19)	Sham (n = 48)	Difference or Odds Ratio (95% CI)	P value
HDRS				
Decrease in HDRS score	7.08 ± 6.99	9.43 ± 6.90	2.35 (-5.8 to 1.1)	0.18
Clinical response	6 (40.6%)	16 (41.7%)	0.94 (0.28 to 3.22)	0.94
Clinical remission	5 (34.3%)	12 (30.9%)	1.17 (0.31 to 4.38)	0.83
MADRS				
Decrease in score	7.86 ± 8.81	9.21 ± 8.27	1.35 (-5.7 to 3.0)	0.54
Clinical response	6 (34.1%)	18 (37.6%)	0.86 (0.22 to 3.26)	0.84
Clinical remission	6 (37.3%)	17 (35.5%)	1.08 (0.28 to 4.09)	0.91
MADRS-s				
Decrease in score	8.90 ± 9.95	9.64 ± 9.42	0.74 (-6.7 to 5.3)	0.81
Clinical response	5 (55.0%)	9 (29.0%)	3.00 (0.67 to 13.40)	0.19
Clinical remission	4 (46.5%)	13 (34.4%)	1.66 (0.30 to 9.08)	0.59
EQ-5D-3L				
Change in score	0.05 ± 0.14	0.09 ± 0.15	-0.03 (-0.09 to 0.03)	0.37

HDRS, 17-item Hamilton Depression Rating Scale;[2] MADRS, Montgomery-Åsberg Depression Rating Scale;[11] MADRS-s, Montgomery-Åsberg Depression Rating Scale-self report;[6] EQ-5D-3L, quality of life measure;[15–17] CI, confidence interval. Mean values are presented with '±' standard deviation values. HDRS, MADRS, MADRS-s change ratings are the change in total ratings from baseline to week 10. Between-group differences are shown for the changes in scores from baseline to week 10, and odds ratios are shown for the outcomes for clinical response and remission. Percentages for clinical response and remission outcomes are estimated based on odds ratios. HDRS scores range from 0 to 52; MADRS scores range from 0 to 60; MADRS-s scores range from 0 to 54, with higher scores indicating increased depressive symptom severity. Clinical response was defined as a decrease in the score (indicating less depressive severity) of more than 50% from baseline to week 10. Clinical remission was defined as: HDRS score of 7 or less; MADRS score of 10 or less; MADRS-s score of 12 or less. Fully Conditional Specification (FCS) approach was used to produce 20 multiply imputed completed data sets. The FCS approach accommodates nonmonotonicity in the pattern of missing data and requires regression models to be specified for each variable with missing values needing imputation. All models included age, sex, in psychotherapy at baseline, use of any antidepressants at baseline and treatment group. The resulting completed datasets were combined using Rubin's Rules.

Supplementary Materials

Home-based transcranial direct current stimulation treatment for major depressive disorder: a fully remote phase 2 randomized sham-controlled trial

Supplementary Table 39. Blinding evaluation and guess certainty mITT sample.

	Treatment arm		
**Arm guessed**	**Active**	**Sham**	**Total**
Total sample			
‘Active’	66 (77.6%)	48 (59.3%)	104
‘Sham’	19 (22.4%)	33 (40.7%)	52
Total	85	81	p=0.012
'Very certain' (4 or 5 out of 5)			
‘Active’	38 (57.6%)	20 (41.7%)	58
‘Sham’	8 (42.1%)	10 (30.3%)	18
Total	46	30	p=0.17
'Very uncertain' (1 or 2 out of 5)			
‘Active’	11 (16.7%)	9 (18.8%)	20
‘Sham’	4 (21.1%)	11 (33.3%)	15
Total	15	20	p=0.17

The number of participants is presented with the percentage in parenthesis. Total n=166 consisting of participants who completed week 10 or who had an early termination visit. 8 participants did not complete a blinding analysis. P values represent two-sided Fisher exact test which compares how many participants guessed active or sham in both groups. At the end of the 10-week blinded phase of the trial, participants were asked whether they thought they had been using the 'active' or 'inactive' or ‘sham’ tDCS device and how certain they were, as measured by a rating on a scale from 1 ('very uncertain') to 5 ('very certain').

Supplementary Materials

Home-based transcranial direct current stimulation treatment for major depressive disorder: a fully remote phase 2 randomized sham-controlled trial

Supplementary Table 40. Completers analysis (N=149) of changes in depressive severity as measured by HDRS following a 10-week course of active or sham tDCS

Measure	Guessed Arm Active (N=100)	Guessed Arm Sham (N=49)	Difference	p-value
Change in HDRS score	-10.04	-6.86	3.18	0.0005

HDRS, 17-item Hamilton Depression Rating Scale; tDCS, transcranial direct current stimulation. Baseline HDRS scores for completers sample (active = 19.03, sham = 18.77). P value assessed with ANOVA.

Table 40 shows the change in HDRS score for the active and sham treatment arms for all participants who completed the 10-week RCT blinded phase (N=149). The results are consistent with the primary outcome of the trial in which the change in HDRS score was greater in active treatment arm as compared to the sham treatment arm.

Supplementary Table 41. Changes in depressive severity at week 10 as measured by HDRS in participants who made a guess that they were receiving active or sham tDCS treatment

Measure	Treatment Arm Active (N=75)	Treatment Arm Sham (N=74)	Difference	p-value
Change in HDRS score	-10.24	-7.76	2.48	0.004

HDRS, 17-item Hamilton Depression Rating Scale; tDCS, transcranial direct current stimulation. P value assessed with ANOVA.

Table 41 shows the change in HDRS score for participants who made guesses that they were receiving active or sham treatment for all participants who completed the 10-week blinded phase of the trial (N=149). The results show that the change in HDRS score was greater in participants who made a guess of active treatment arm as compared to sham treatment arm. We would expect this outcome as participants who had experienced an improvement in their depressive symptoms are more likely to believe that they were receiving the active treatment.

Supplementary Table 42. Changes in depressive severity at week 10 as measured by HDRS in participants who made a guess that they were receiving active treatment

	Actual Treatment arm			
Measure	Active	Sham	Difference	p-value
Total sample				
Change in HDRS score	-11.14 ± 5.01	-8.58 ± 4.71	-2.56 (0.60 to 4.52)	0.01
N	57	43		
Certainty (guess 3 to 5 out of 5)				
Decrease in HDRS score	-11.78 ± 4.89	-9.00 ± 5.03	-2.78 (0.63 to 4.93)	0.01
N	50	36		

HDRS, 17-item Hamilton Depression Rating Scale. P values assessed with ANOVA.

Table 42 shows the change in HDRS score at week 10 for participants who made a guess that they were receiving the active stimulation and grouped into their real allocation of active treatment (indicating a correct guess) and sham treatment (indicating an incorrect guess).

If there were only a placebo effect, we would expect participants who believed that they were receiving the active treatment to have a similar improvement in depressive symptoms as the expectation bias would be the same for both groups. However, this analysis shows a greater decrease in HDRS scores for participants who made a guess that they were receiving active stimulation and were in the actual active treatment arm as compared to participants who made a guess that they were receiving active stimulation but were in the sham treatment arm.

It is possible that there was a greater placebo effect in participants who endorsed a higher certainty of their guess. When the analysis is limited to participants who endorsed a certainty of 3-5 out of a scale of 1-5 (1=very uncertain, 5=very certain), the analysis shows a greater decrease in HDRS scores in participants who were certain of their guess that they were receiving active stimulation and were in the actual active treatment arm as compared to participants who were certain of their guess that they were receiving active stimulation but were in the sham treatment arm.

Supplementary Table 43. Changes in depressive severity at week 10 as measured by HDRS shown for participants who made a guess that they were receiving sham treatment

	Actual Treatment arm			
Measure	Active	Sham	Difference	p-value
Total sample				
Change in HDRS score	-7.24 ± 5.45	-6.66 ± 5.25	0.58 (-2.63 to 3.79)	0.72
N	17	32		
Certainty (guess 3 to 5 out of 5)				
Change in HDRS score	-7.46 ± 5.52	-6.52 ± 5.51	0.94 (-3.03 to 4.90)	0.63
N	13	21		

HDRS, 17-item Hamilton Depression Rating Scale. P values assessed with ANOVA.

Table 43 shows the change in HDRS score at week 10 for participants who made a guess that they were receiving the sham stimulation and grouped into their real allocation of active treatment (indicating an incorrect guess) and sham treatment (indicating a correct guess).

Supplementary Materials

Home-based transcranial direct current stimulation treatment for major depressive disorder: a fully remote phase 2 randomized sham-controlled trial

Supplementary Table 44. Distributional changes in severity of depressive symptoms from baseline to week 10 based on Hamilton Depression Rating Scale score for participants who completed the blinded phase (n=149)

**Active group**						
		**Week 10 severity**				
**Total baseline severity**		Very severe	Severe	Moderate	Mild	Remission
Very severe	11	1	1	3	1	5
Severe	24	0	2	4	10	8
Moderate	39	0	0	5	13	21
Total at week 10		1	3	12	24	34

**Sham group**						
		**Week 10 severity**				
**Total baseline severity**		Very severe	Severe	Moderate	Mild	Remission
Very severe	8	0	1	4	2	1
Severe	26	1	1	8	8	8
Moderate	41	0	1	11	21	8
Total at week 10		1	3	23	31	17

Severity is based on the following 17-item Hamilton Depression Rating Scale scores. Remission, 0-7; mild, 8-13; moderate, 14-18; severe, 19-22; very severe, >=23.

Supplementary Materials

Home-based transcranial direct current stimulation treatment for major depressive disorder: a fully remote phase 2 randomized sham-controlled trial

Supplementary Figure 13. Distribution of depressive symptom severity at week 10 based on Hamilton Depression Rating Scale score for participants who completed the blinded phase (n=149)

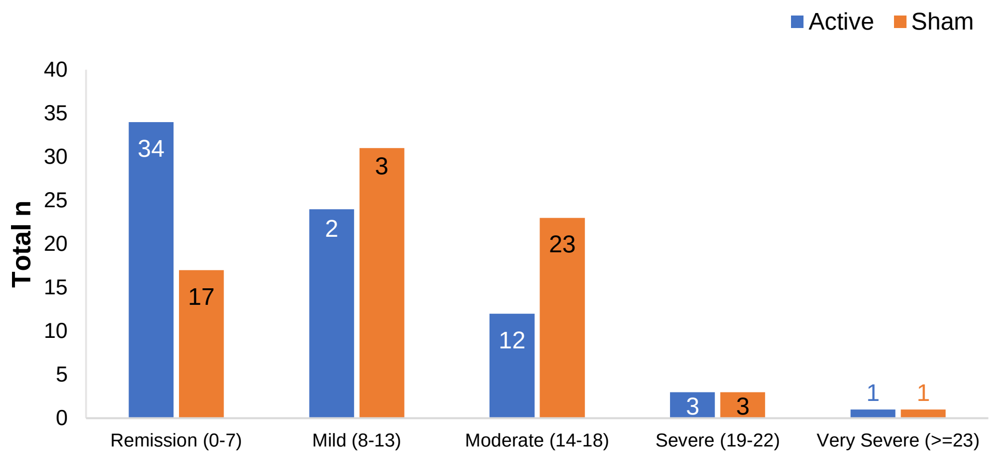

Supplementary Materials

Home-based transcranial direct current stimulation treatment for major depressive disorder: a fully remote phase 2 randomized sham-controlled trial

Supplementary Table 45. Baseline demographic and clinical characteristics of participants who completed the week 20 open-label follow up

Characteristic	Active (N=67)	Sham (N=71)
Age	37.5 ± 10.6	37.6 ± 10.4
Sex		
Women	42 (63)	54 (76)
Race		
Asian	5 (7)	2 (3)
Black or African American	3 (4)	1 (1)
Native Hawaiian or Other	0 (0)	0 (0)
White	56 (84)	60 (85)
Other	3 (4)	8 (11)
Missing	0 (0)	0 (0)
Ethnicity		
Hispanic or Latino	6 (9)	8 (11)
Not Hispanic or Latino	59 (88)	59 (83)
Not reported	2 (3)	3 (4)
Missing	0 (0)	1 (1)
Educational Level		
Less than High School/Secondary School	1 (1)	0 (0)
Some College	15 (22)	15 (21)
Diploma	5 (7)	6 (8)
Bachelor's or Professional Degree	28 (42)	32 (45)
Master's or Doctoral Degree	18 (27)	17 (24)
Prefer not to answer/Missing	0 (0)	1 (1)
Clinical ratings		
HDRS	19.0 ± 2.7	18.8 ± 2.6
MADRS	24.5 ± 4.3	23.9 ± 5.2
MADRS-s	26.2 ± 6.6	25.6 ± 6.3
HAMA	15.2 ± 4.4	14.4 ± 4.7
YMRS	2.1 ± 1.7	2.1 ± 1.6
RAVLT	57.5 ± 11.3	57.0 ± 12.6
SDMT	51.4 ± 9.9	51.0 ± 10.3

Categorical variables are presented as number of participants with percentage in parentheses for sex, race, ethnicity and educational level. Mean values are presented with '±' standard deviation values. HDRS, 17-item Hamilton Depression Rating Scale;[2] MADRS, Montgomery-Åsberg Depression Rating Scale;[11] MADRS-s, Montgomery-Åsberg Depression Rating Scale-self report;[6] HAMA, Hamilton Anxiety Rating Scale;[12] YMRS, Young Mania Rating Scale;[3] RAVLT, Rey Auditory Verbal Learning Test;[19] SDMT, Symbol Digit Modalities Test.[18] SDMT active, n=65, SDMT sham, n=65. HDRS scores range from 0 to 52; MADRS scores range from 0 to 60; MADRS-s scores range from 0 to 54, with higher scores indicating increased depressive symptom severity. RAVLT scores range from 0 to 75. SDMT scores range from 0 to 110.

Supplementary Materials

Home-based transcranial direct current stimulation treatment for major depressive disorder: a fully remote phase 2 randomized sham-controlled trial

Supplementary Table 46. Transition analysis of week 10 and week 20 response rates for patients who completed >=660 minutes of stimulation in the open label phase

Table 46 shows response rates at week 10 and week 20 for all participants in the active and sham groups who completed a minimum of 60% sessions (22 sessions) in the 10-week blinded phase of the trial and a minimum of 60% sessions (22 sessions; 660 minutes of active stimulation) in the open label phase of the trial.

In the active group, all participants who responded to treatment in the 10-week blinded phase, continued to be responders at week 20. Of the 8 participants who did not show a response to treatment during the blinded phase, 3 participants showed a treatment response at week 20.

In the sham group, 8 of the 9 participants who showed a treatment response in the 10-week blinded phase continued to show a treatment response at week 20 after receiving the active treatment. Of the 40 participants who did not show a treatment response during the 10-week blinded phase, 20 participants showed a treatment response at week 20 after receiving the active treatment.

Active Group				Sham Group			
**Week 10**	**Week 20**			**Week 10**	**Week 20**		
**N<br>Row %<br>Column %**	**Clinical Response**	**No clinical response**	**Total**	**N<br>Row %<br>Column %**	**Clinical Response**	**No clinical response**	**Total**
**Clinical Response**	17<br>100%<br>85%	0<br>0%<br>0%	17	**Clinical Response**	8<br>88.89%<br>28.57%	1<br>11.11%<br>4.76%	9
**No clinical response**	3<br>37.5%<br>15%	5<br>62.5%<br>100%	8	**No clinical response**	20<br>50.00%<br>71.43%	20<br>50.00<br>95.24%	40
**Total**	20<br>80%	5<br>20%	25<br>100%	**Total**	28<br>57.14%	21<br>42.86%	49<br>100%
**Frequency Missing = 4**				**Frequency Missing = 4**			

Clinical response, >50% reduction from baseline in Hamilton Depression Rating Scale score.

Supplementary Materials

Home-based transcranial direct current stimulation treatment for major depressive disorder: a fully remote phase 2 randomized sham-controlled trial

Supplementary Table 47. Transition analysis of week 10 and week 20 response rates for patients who completed >=330 minutes of stimulation in the open label phase

Table 47 shows response rates at week 10 and week 20 for all participants in the active and sham groups who completed a minimum of 60% sessions (22 sessions) in the 10-week blinded phase of the trial and a minimum of 11 sessions (330 minutes of active stimulation) in the open label phase of the trial.

In the active group, 26 of the 27 participants who showed a treatment response in the 10-week blinded phase continued to show a treatment response at week 20. Of the 15 participants who did not show a treatment response in the blinded phase, 7 participants showed a treatment response at week 20.

In the sham group, 9 of the 11 participants who showed a treatment response in the 10-week blinded phase continued to show a treatment responses at week 20. Of the 41 participants who did not show a treatment response in the blinded phase, 21 participants showed a treatment response at week 20.

Active Group				Sham Group			
**Week 10**	**Week 20**			**Week 10**	**Week 20**		
**N<br>Row %<br>Column %**	**Clinical Response**	**No clinical response**	**Total**	**N<br>Row %<br>Column %**	**Clinical Response**	**No clinical response**	**Total**
**Clinical Response**	26<br>96.3%<br>78.79%	1<br>3.7%<br>11.11%	27	**Clinical Response**	9<br>81.82%<br>30%	2<br>18.18%<br>9.09%	11
**No clinical response**	7<br>46.67%<br>21.21%	8<br>53.33%<br>88.89%	15	**No clinical response**	21<br>51.22%<br>70%	20<br>48.78%<br>90.91%	41
**Total**	33<br>78.57%	9<br>21.43%	42<br>100%	**Total**	30<br>57.69%	22<br>42.31%	52<br>100%
**Frequency Missing = 9**				**Frequency Missing = 5**			

Clinical response, >50% reduction from baseline in Hamilton Depression Rating Scale score.

Supplementary Materials

Home-based transcranial direct current stimulation treatment for major depressive disorder: a fully remote phase 2 randomized sham-controlled trial

Supplementary Table 48. Transition analysis of week 10 and week 20 response rates for patients who completed >=165 minutes of stimulation in the open label phase

Table 48 shows response rates at week 10 and week 20 for all participants in the active and sham groups who completed a minimum of 60% sessions (22 sessions) in the 10-week blinded phase of the trial and a minimum of 165 minutes of active stimulation in the open label phase of the trial.

In the active group, 28 of the 29 participants who showed a treatment response in the 10-week blinded phase continued to show a treatment response at week 20. Of the 20 participants who did not show a treatment response in the blinded phase, 9 participants showed a treatment response at week 20.

In the sham group, 9 of the 11 participants who showed a treatment response in the 10-week blinded phase continued to show a treatment response at week 20. Of the 42 participants who did not show a treatment response in the blinded phase, 21 participants showed a treatment response at week 20.

Active Group				Sham Group			
**Week 10**	**Week 20**			**Week 10**	**Week 20**		
**N<br>Row %<br>Column %**	**Clinical Response**	**No clinical response**	**Total**	**N<br>Row %<br>Column %**	**Clinical Response**	**No clinical response**	**Total**
**Clinical Response**	28<br>96.55%<br>75.68%	1<br>3.45%<br>8.33%	29	**Clinical Response**	9<br>81.82%<br>30%	2<br>18.18%<br>8.70%	11
**No clinical response**	9<br>45%<br>24.32%	11<br>55%<br>91.67%	20	**No clinical response**	21<br>50%<br>70%	21<br>50%<br>91.30%	42
**Total**	37<br>75.51%	12<br>24.49%	49<br>100%	**Total**	30<br>56.60%	23<br>43.40%	53<br>100%
**Frequency Missing = 10**				**Frequency Missing = 10**			

Clinical response, >50% reduction from baseline in Hamilton Depression Rating Scale score.

Supplementary Table 49. Transition analysis of week 10 and week 20 response rates for patients who completed >=80 minutes of stimulation in the open label phase

Table 49 shows response rates at week 10 and week 20 for all participants in the active and sham groups who completed a minimum of 60% sessions (22 sessions) in the 10-week blinded phase of the trial and a minimum of 80 minutes of active stimulation in the open label phase of the trial.

In the active group, 29 of the 30 participants who showed a treatment response in the 10-week blinded phase continued to show a treatment response at week 20. Of the 20 participants who did not show a treatment response in the blinded phase, 9 participants showed a treatment response at week 20.

In the sham group, 9 of the 11 participants who showed a treatment response in the 10-week blinded phase continued to show a treatment response at week 20. Of the 42 participants who did not show a treatment response in the blinded phase, 21 participants showed a treatment response at week 20.

**Active Group**				**Sham Group**			
**Week 10**	**Week 20**			**Week 10**	**Week 20**		
**N<br>Row %<br>Column %**	**Clinical Response**	**No clinical response**	**Total**	**N<br>Row %<br>Column %**	**Clinical Response**	**No clinical response**	**Total**
**Clinical Response**	29<br>96.67%<br>76.32%	1<br>3.33%<br>8.33%	30	**Clinical Response**	9<br>81.82%<br>30%	2<br>18.18%<br>8.70%	11
**No clinical response**	9<br>45%<br>23.68%	11<br>55%<br>91.67%	20	**No clinical response**	21<br>50%<br>70%	21<br>50%<br>91.30%	42
**Total**	38<br>76%	12<br>24%	50<br>100%	**Total**	30<br>56%	23<br>43.40%	53<br>100%
**Frequency Missing = 11**				**Frequency Missing = 13**			

Clinical response, >50% reduction from baseline in Hamilton Depression Rating Scale score.

Supplementary Table 50. Transition analysis of week 10 and week 20 response rates for patients who completed >=0 minutes of stimulation in the open label phase

Table 50 shows response rates at week 10 and week 20 for all participants in the active and sham groups who completed a minimum of 60% sessions (22 sessions) in the 10-week blinded phase of the trial and a >=0 minutes of active stimulation in the open label phase of the trial.

In the active group, 30 of the 31 participants who showed a treatment response in the 10-week blinded phase continued to show a treatment response at week 20. Of the 24 participants who did not show a treatment response in the blinded phase, 9 participants showed a treatment response at week 20.

In the sham group, 10 of the 12 participants who showed a treatment response in the 10-week blinded phase continued to show a treatment response at week 20. Of the 44 participants who did not show a treatment response in the blinded phase, 22 participants showed a treatment response at week 20.

Active Group				Sham Group			
**Week 10**	**Week 20**			**Week 10**	**Week 20**		
**N<br>Row %<br>Column %**	**Clinical Response**	**No clinical response**	**Total**	**N<br>Row %<br>Column %**	**Clinical Response**	**No clinical response**	**Total**
**Clinical Response**	30<br>96.77%<br>76.92%	1<br>3.23%<br>6.25%	31	**Clinical Response**	10<br>83.33%<br>31.25%	2<br>16.67%<br>8.33%	12
**No clinical response**	9<br>37.50%<br>23.08%	15<br>62.50%<br>93.75%	24	**No clinical response**	22<br>50%<br>68.75%	22<br>50%<br>91.67%	44
**Total**	39<br>70.91%	16<br>29.09%	55<br>100%	**Total**	32<br>57.14%	24<br>42.86%	56<br>100%
**Frequency Missing = 32**				**Frequency Missing = 30**			

Clinical response, >50% reduction from baseline in Hamilton Depression Rating Scale score.

Supplementary Materials

Home-based transcranial direct current stimulation treatment for major depressive disorder: a fully remote phase 2 randomized sham-controlled trial

Supplementary Table 51. All unanticipated device related adverse events in the open label phase

	Active (N= 67)			Sham (N= 71)			Group Difference			
	Events	Subjs	%	Events	Subjs	%	Δ	LB	UB	p
**All Events**	**3**	**3**	**4.5%**	**6**	**6**	**8.5%**	**4.0%**	**-13.5%**	**5.2%**	**0.50**
**Ear and labyrinth disorders**	**1**	**1**	**1.5%**	**1**	**1**	**1.4%**	**0.1%**	**-6.3%**	**6.9%**	**0.99**
Tinnitus	1	1	1.5%	1	1	1.4%	0.1%	-6.3%	6.9%	0.99
**Gastrointestinal disorders**	**0**	**0**	**0.0%**	**1**	**1**	**1.4%**	**1.4%**	**-7.7%**	**4.4%**	**0.99**
Nausea	0	0	0.0%	1	1	1.4%	1.4%	-7.7%	4.4%	0.99
**Nervous system disorders**	**1**	**1**	**1.5%**	**0**	**0**	**0.0%**	**1.5%**	**-3.8%**	**8.2%**	**0.49**
Headache	1	1	1.5%	0	0	0.0%	1.5%	-3.8%	8.2%	0.49
**Psychiatric disorders**	**1**	**1**	**1.5%**	**0**	**0**	**0.0%**	**1.5%**	**-3.8%**	**8.2%**	**0.49**
Mood Altered	1	1	1.5%	0	0	0.0%	1.5%	-3.8%	8.2%	0.49
**Skin and subcutaneous tissue disorders**	**0**	**0**	**0.0%**	**4**	**4**	**5.6%**	**5.6%**	**-13.8%**	**0.2%**	**0.12**
Dry skin	0	0	0.0%	4	4	5.6%	5.6%	-13.8%	0.2%	0.12

Adverse events are reported for participants that completed >0 minutes of stimulation during the open label phase. An adverse event was present if the participant rated that it was at least possibly associated with the intervention. Participants rated the severity of the adverse events as mild, moderate, or severe, which were assessed by the investigator. P values represent difference between groups assessed by two-sided Fisher exact test. LB, lower bound; UB, upper bound.

Supplementary Materials

Home-based transcranial direct current stimulation treatment for major depressive disorder: a fully remote phase 2 randomized sham-controlled trial

Supplementary Table 52. All unanticipated adverse events in the open label phase

	Active (N= 67)			Sham (N= 71)			Group Difference			
	Events	Subjs	%	Events	Subjs	%	Δ	LB	UB	p
**All Events**	**6**	**6**	**9.0%**	**14**	**12**	**16.9%**	**7.9%**	**20.1%**	**3.7%**	**0.21**
**Blood and lymphatic system disorders**	**0**	**0**	**0.0%**	**1**	**1**	**1.4%**	**1.4%**	**-7.7%**	**4.4%**	**0.99**
Anaemia	0	0	0.0%	1	1	1.4%	1.4%	-7.7%	4.4%	0.99
**Ear and labyrinth disorders**	**1**	**1**	**1.5%**	**1**	**1**	**1.4%**	**0.1%**	**-6.3%**	**6.9%**	**0.99**
Tinnitus	1	1	1.5%	1	1	1.4%	0.1%	-6.3%	6.9%	0.99
**Gastrointestinal disorders**	**0**	**0**	**0.0%**	**1**	**1**	**1.4%**	**1.4%**	**-7.7%**	**4.4%**	**0.99**
Nausea	0	0	0.0%	1	1	1.4%	1.4%	-7.7%	4.4%	0.99
**General disorders and administration site conditions**	**0**	**0**	**0.0%**	**2**	**2**	**2.8%**	**2.8%**	**10.0%**	**3.1%**	**0.50**
Asthenia	0	0	0.0%	1	1	1.4%	1.4%	-7.7%	4.4%	0.99
Illness	0	0	0.0%	1	1	1.4%	1.4%	-7.7%	4.4%	0.99
**Immune system disorders**	**1**	**1**	**1.5%**	**0**	**0**	**0.0%**	**1.5%**	**-3.8%**	**8.2%**	**0.49**
Seasonal allergy	1	1	1.5%	0	0	0.0%	1.5%	-3.8%	8.2%	0.49
**Infections and infestations**	**1**	**1**	**1.5%**	**4**	**4**	**5.6%**	**4.1%**	**12.5%**	**3.1%**	**0.37**
COVID-19	0	0	0.0%	2	2	2.8%	2.8%	10.0%	3.1%	0.50
Lower respiratory tract infection	0	0	0.0%	2	2	2.8%	2.8%	10.0%	3.1%	0.50
Sinusitis	1	1	1.5%	0	0	0.0%	1.5%	-3.8%	8.2%	0.49
**Musculoskeletal and connective tissue disorders**	**1**	**1**	**1.5%**	**0**	**0**	**0.0%**	**1.5%**	**-3.8%**	**8.2%**	**0.49**
Pain in extremity	1	1	1.5%	0	0	0.0%	1.5%	-3.8%	8.2%	0.49
**Nervous system disorders**	**1**	**1**	**1.5%**	**0**	**0**	**0.0%**	**1.5%**	**-3.8%**	**8.2%**	**0.49**
Headache	1	1	1.5%	0	0	0.0%	1.5%	-3.8%	8.2%	0.49
**Psychiatric disorders**	**1**	**1**	**1.5%**	**0**	**0**	**0.0%**	**1.5%**	**-3.8%**	**8.2%**	**0.49**
Mood Altered	1	1	1.5%	0	0	0.0%	1.5%	-3.8%	8.2%	0.49
**Reproductive system and breast disorders**	**0**	**0**	**0.0%**	**1**	**1**	**1.4%**	**1.4%**	**-7.7%**	**4.4%**	**0.99**
Pelvic pain	0	0	0.0%	1	1	1.4%	1.4%	-7.7%	4.4%	0.99
**Skin and subcutaneous tissue disorders**	**0**	**0**	**0.0%**	**4**	**4**	**5.6%**	**5.6%**	**13.8%**	**0.2%**	**0.12**
Dry skin	0	0	0.0%	4	4	5.6%	5.6%	13.8%	0.2%	0.12

All unanticipated adverse events that were reported between week 10 and week 20 including those that weren't associated with the intervention for participants that completed >0 minutes of stimulation during the open label phase. Participants rated the severity of the adverse events as mild, moderate, or severe, which were assessed by the investigator. P values represent difference between groups assessed by two-sided Fisher exact test. LB, lower bound; UB, upper bound.

Supplementary Materials

Home-based transcranial direct current stimulation treatment for major depressive disorder: a fully remote phase 2 randomized sham-controlled trial

Supplementary Table 53. All anticipated adverse events in the open label phase as measured by the tDCS Adverse Events Questionnaire.[22]

	Active (N=56)				Sham (N=57)				
**Adverse event category**	**Total**	**Mild**	**Moderate**	**Severe**	**Total**	**Mild**	**Moderate**	**Severe**	**P Value**
Headache	12 (21%)	8 (14%)	3 (5%)	1 (2%)	10 (18%)	7 (12%)	3 (5%)	0 (0%)	0.64
Neck Pain	1 (2%)	1 (2%)	0 (0%)	0 (0%)	2 (4%)	0 (0%)	2 (4%)	0 (0%)	1.00
Scalp pain	10 (18%)	8 (14%)	2 (4%)	0 (0%)	14 (25%)	10 (18%)	4 (7%)	0 (0%)	0.49
Itching	26 (46%)	19 (34%)	6 (11%)	1 (2%)	23 (40%)	19 (33%)	3 (5%)	1 (2%)	0.57
Burning sensation	22 (39%)	20 (36%)	2 (4%)	0 (0%)	26 (46%)	20 (35%)	6 (11%)	0 (0%)	0.57
Skin redness	34 (61%)	28 (50%)	6 (11%)	0 (0%)	30 (53%)	30 (53%)	0 (0%)	0 (0%)	0.45
Sleepiness	6 (11%)	4 (7%)	2 (4%)	0 (0%)	9 (16%)	8 (14%)	1 (25)	0 (0%)	0.58
Trouble concentrating	5 (9%)	2 (4%)	3 (5%)	0 (0%)	2 (4%)	2 (4%)	0 (0%)	0 (0%)	0.27
Acute mood change	1 (2%)	1 (2%)	0 (0%)	0 (0%)	0 (0%)	0 (0%)	0 (0%)	0 (0%)	0.50

Values are number of participants with percentage in parentheses. An adverse event was present if the participant rated that it was at least remotely possible that it was associated with the intervention. Participants rated the severity of the adverse events as mild, moderate, or severe. P values represent group differences of the total number of events per event category assessed by two-sided Fisher exact test.

Supplementary Materials

Home-based transcranial direct current stimulation treatment for major depressive disorder: a fully remote phase 2 randomized sham-controlled trial

References

1. American Psychiatric Association. *Diagnostic and Statistical Manual of Mental Disorders*. (American Psychiatric Association, 2013). doi:10.1176/appi.books.9780890425596.
2. Hamilton, M. A rating scale for depression. *J. Neurol. Neurosurg. Psychiatry* 23, 56–62 (1960).
3. Young, R. C., Biggs, J. T., Ziegler, V. E. & Meyer, D. A. A rating scale for mania: reliability, validity and sensitivity. *Br. J. Psychiatry* 133, 429–435 (1978).
4. Sheehan, D. V. *et al.* The Mini-International Neuropsychiatric Interview (M.I.N.I.): the development and validation of a structured diagnostic psychiatric interview for DSM-IV and ICD-10. *J. Clin. Psychiatry* 59 Suppl 20, 22-33;quiz 34-57 (1998).
5. Posner, K. *et al.* The Columbia-Suicide Severity Rating Scale: Initial validity and internal consistency findings from three multisite studies with adolescents and adults. *Am. J. Psychiatry* 168, 1266–1277 (2011).
6. Svanborg, P. & Åsberg, M. A comparison between the Beck Depression Inventory (BDI) and the self-rating version of the Montgomery Åsberg Depression Rating Scale (MADRS). *J. Affect. Disord.* 64, 203–216 (2001).
7. Stanley, B. & Brown, G. K. Safety Planning Intervention: A Brief Intervention to Mitigate Suicide Risk. *Cogn. Behav. Pract.* 19, 256–264 (2012).
8. Lachin, J. M. A review of methods for futility stopping based on conditional power. *Stat. Med.* 24, 2747–2764 (2005).
9. Mehta, C. R. & Pocock, S. J. Adaptive increase in sample size when interim results are promising: A practical guide with examples. *Stat. Med.* 30, 3267–3284 (2011).
10. Keenan, B. T. & Maislin, G. The use of 'Fuzzy Promising Zones' to mitigate against operational bias in adaptive sample size re-estimation designs. in (Boston, Massachusetts, 2014).
11. Montgomery, S. A. & Åsberg, M. A new depression scale designed to be sensitive to change. *Br. J. Psychiatry J. Ment. Sci.* 134, 382–389 (1979).
12. Hamilton, M. The assessment of anxiety states by rating. *Br. J. Med. Psychol.* 32, 50–55 (1959).
13. Hochberg, Y. A sharper bonferroni procedure for multiple tests of significance. *Biometrika* 75, 800–802 (1988).
14. Dmitrienko, A. & D'Agostino Sr, R. Traditional multiplicity adjustment methods in clinical trials. *Stat. Med.* 32, 5172–5218 (2013).
15. Rabin, R. & Charro, F. de. EQ-5D: a measure of health status from the EuroQol Group. *Ann. Med.* 33, 337–343 (2001).
16. Brooks, R. EuroQol: the current state of play. *Health Policy* 37, 53–72 (1996).
17. EuroQol - a new facility for the measurement of health-related quality of life. *Health Policy* 16, 199–208 (1990).
18. Smith, A. *Symbol Digit Modalities Test*. (Western Psychological Services, Los Angeles, CA, 1991).
19. Rey, A. *L'Examen Clinique En Psychologie*. (Presses Universitaires de France, Paris, 1964).
20. Fantino, B. & Moore, N. The self-reported Montgomery-Åsberg depression rating scale is a useful evaluative tool in major depressive disorder. *BMC Psychiatry* 9, 26 (2009).
21. Lang, T. A. & Stroup, D. F. Who knew? The misleading specificity of 'double-blind' and what to do about it. *Trials* 21, 697 (2020).
22. Brunoni, A. R. *et al.* A systematic review on reporting and assessment of adverse effects associated with transcranial direct current stimulation. *Int. J. Neuropsychopharmacol.* 14, 1133–1145 (2011).

Statistical analysis plan - FL001

Version history:

1. 21Mar2022: ver1: Added details of MMRM and Missing data handling.
2. 07Dec2022: ver2: Re-organize document; added symbolic hypotheses; added interim analysis details. Clarified secondary endpoints.
3. 13Jan2023: Adjustments to align with new Adaptive design report.

1. Trial Overview **3**

2. Study Populations **3**

2.1. Intent-to-Treat Analysis Set (ITT) 3

2.2. As Treated Analysis Set (AT) 4

2.3. Modified Intent-to-Treat Analysis Set (mITT) 4

2.4. Per Protocol Analysis Set (PP) 4

3. Study Endpoints **4**

3.1. Primary Effectiveness Endpoint 4

3.1.1. Testing of Primary Effectiveness Hypotheses 4

3.1.2. Multiple Imputation Analysis 5

3.1.3. Secondary Endpoints 5

3.1.4. Discussion on FDA Requested secondary endpoints and clarification of the endpoints controlled for multiplicity 5

3.1.5. Secondary Endpoint #1: Change in MADRS score 5

3.1.5.1. Endpoint Definition 6

3.1.5.2. Hypothesis Test 6

3.1.5.3. Analysis Set 6

3.1.5.4. Missing Data Analysis 6

3.1.6. Secondary Endpoint #2: Clinical Response 6

3.1.6.1. Endpoint Definition 6

3.1.6.2. Hypothesis Test 6

3.1.6.3. Analysis Set 7

3.1.6.4. Missing Data Analysis 7

3.1.7. Secondary Endpoint #3: Remission 7

3.1.7.1. Endpoint Definition 7

3.1.7.2. Hypothesis Test 7

3.1.7.3. Analysis Set 7

3.1.7.4. Missing Data Analysis 8

3.1.8. Secondary Endpoint #4: Quality of Life Improvement 8

3.1.8.1. Endpoint Definition 8

3.1.8.2. Hypothesis Test 8

3.1.8.3. Analysis Set 8

3.1.8.4. Missing Data Analysis 8

3.1.8.5. Additional analyses 8

3.1.9. Multiplicity Control for Secondary Endpoints 8

3.1.9.1. Hochberg Algorithm 9

3.1.10. Exploratory Endpoints 10

4. Details of the MMRM 11

5. Missing data 12

5.1. Procedures For Minimizing The Occurrence Of Missing Data 12

5.2 Data Collection For Subjects Who Discontinue From Randomized Treatment Or Discontinue From The Study 13

5.2.1. DISCONTINUATION DEFINITIONS AS PROVIDED IN THE PROTOCOL 13

5.2.2. DISCONTINUATION FROM STUDY BY SUBJECT 13

5.2.3. DISCONTINUATION FROM RANDOMIZED TREATMENT BY INVESTIGATOR 14

5.2.4. TEMPORARY DISCONTINUATION BY SUBJECT 14

5.2.5. DISCONTINUATION OF INADVERTENTLY ENROLLED SUBJECTS 14

5.2.6. LOST TO FOLLOW-UP (LTFU) 14

5.2.7. USE OF DATA FROM EARLY DISCONTINUATION CASES 14

5.3. SPECIFICATION OF THE ESTIMAND 15

5.3.1. PRIMARY ESTIMAND 15

5.4. Handling Of Missing Data 16

5.4.1. HANDLING DATA FOLLOWING SUBJECT WITHDRAWAL DUE TO LACK OF TREATMENT EFFECTIVENESS 16

5.4.2. HANDLING OF SUBJECTS REASONABLY MISSING AT RANDOM 16

5.4.3. HANDLING OF SUBJECTS WITH MISSINGNESS FOR ALL OTHER REASONS 16

5.4.4. MULTIPLE IMPUTATION - GENERAL 17

5.4.5. DEVELOPMENT OF MI MODEL 17

5.4.6. COVARIATES IN THE MI MODEL 17

5.4.7. CHOICE OF MI MODEL 17

5.4.8. BASELINE COVARIATES 18

5.4.9. LONGITUDINAL HDRS-17 18

5.5. Sensitivity Analysis 19

5.5.1. ALTERNATIVE MODELING APPROACHES 19

6. Interim Analysis 19

7. Sample Size Assumptions and Calculation 19

8. Additional Analyses 20

8.1. Evaluation of Blinding 20

8.2. Device Survival 20

8.3. Poolability Analysis 20

8.4. Safety Analyses 20

1. Trial Overview

This trial is a double-blind, placebo-controlled, randomized, superiority, remote trial for individuals with major depressive disorder. The primary objective of this trial is to show that active stimulation with the Flow FL-100 device is superior to sham stimulation for the treatment of major depressive disorder when used at-home. The study is expected to take approximately 10 months from first participant enrolled to the last open-label visit, which consists of a 5 months enrollment period (estimation) and 5 months treatment and open-label. The follow-up period during the clinical investigation shall permit the demonstration of performance over a period, sufficient to represent a realistic test of the performance of the investigational device and allow any risks associated with adverse device effects over that period to be identified and assessed. For each participant, the trial will be conducted in 2 phases: blinded and open label. During the treatment phase, the participants will be randomized into one of the two arms: active and sham. The participants will be divided equally between the arms and will not be informed of their assignment. Subjects will be followed for 10 weeks, at which point the primary endpoint will be assessed.

2. Study Populations

2.1. Intent-to-Treat Analysis Set (ITT)

All randomized subjects will be included in the “intent-to-treat” (ITT) analysis set and classified according to their intended treatment regardless of failure to complete the required follow-up examinations. Subjects excluded prior to randomization will be considered screening failures.

2.2. As Treated Analysis Set (AT)

The As Treated Analysis Set (AT) will consist of ITT subjects who receive at least 1 treatment of study device (Active or Sham). Subjects will be analyzed according to the study treatment received. The As Treated Analysis Set will be used for all summaries of safety and tolerability data.

2.3. Modified Intent-to-Treat Analysis Set (mITT)

The modified intent-to-treat (mITT) analysis set will include ITT subjects who receive at least 1 treatment of study device (Active or Sham) and will exclude patients who were randomized in error. The number of exclusions from the mITT analysis set is expected to be small and will only be based on information that was known or could have been known prior to the first treatment. Subjects in the mITT population will be analyzed in the group to which they were randomized.

2.4. Per Protocol Analysis Set (PP)

The Per Protocol (PP) analysis set will be comprised of the following:

- Subjects in the mITT analysis set
- Subjects with device failure within the 10-week follow-up period
- Subjects with deviation from the clinical investigation plan is caused by the investigational device or by problems with respect to tolerability

Subjects who will be excluded from the PP analysis set are as follows:

- Subjects with major protocol violations that would be expected to confound clinical assessment during follow-up as determined by the PIs.
- Subjects who took new pharmaceutical substances or treatments during the clinical investigation which are listed as an exclusion criteria;
- Subjects who do not meet the inclusion criteria or exclusion criteria.
- Users who have performed less than 10 sessions (300 minutes) during the first 3 weeks will be excluded from the analysis.

Subjects in the PP population will be analyzed in the group to which they were randomized.

3. Study Endpoints

3.1. Primary Effectiveness Endpoint

3.1.1. Testing of Primary Effectiveness Hypotheses

The primary effectiveness endpoint is the change in adjusted mean group difference in the HDRS-17 scores between 10 weeks and baseline between subjects randomized to the active study device relative to those subjects randomized to sham. The formal statistical hypothesis is as follows:

H_o: $d_{flow} – d_{sham} \leq 0$ H_a: $d_{flow} –$
$d_{sham} > 0$

Where d_{flow} and d_{sham} are the adjusted mean group difference in HDRS-17 scores in subjects randomized to Active and Sham groups, respectively.

The mITT population will be the analysis population used to analyze the primary effectiveness endpoint. The endpoint will be analyzed when all randomized subjects have completed their 10-week visit, been withdrawn from the trial or passed the end of their 10-week follow-up visit window. The difference in the group mean differences will be assessed using a mixed model for repeated measures. If the p-value of the difference in the group mean differences are less than one-sided p= 0.025, then the endpoint declared a success.

3.1.2. Multiple Imputation Analysis

The mixed model for repeated measures (MMRM) allows for inclusion of patients with missing Week 10 values. If all subjects have at least one follow-up evaluation, the following MMRM will be utilized for the primary superiority test. If there are subjects without at least one post baseline assessment, the multiple imputation analysis summarized below will be the primary analysis method. Least squares estimated values from the MMRM will be reported for HDRS-17 at each visit and as such these estimates include implicit imputations of missing values.

3.1.3. Secondary Endpoints

There are eight secondary endpoints. **Three of the eight secondary endpoints will be secondary endpoints controlled for multiplicity, the remaining five secondary endpoints will be provided descriptively**. Endpoints #2a, 3a, and 4 will be controlled for multiplicity:

1. The mean change in points for MADRS score from baseline to Week 10.
2. Clinical Response will be analyzed as responder endpoints. The 3 Clinical Response rates are defined as
    a. a >50% reduction from the baseline HDRS-17 at Week 10.
    b. a >50% reduction in MADRS score at Week 10.
    c. a >50% reduction from the baseline MADRS-s at Week 10.
3. Remission will be analyzed as a responder endpoint where remission is defined as:
    a. HDRS-17 score ≤7 at Week 10
    b. MADRS score ≤10 at Week 10
    c. MADRS-s score ≤12 at Week 10
4. Quality of life improvement as measured by EQ-5D-3L in the active compared to sham arm at week 10 compared to baseline.

3.1.4. Discussion on FDA Requested secondary endpoints and clarification of the endpoints controlled for multiplicity

The eight (8) endpoints listed above are consistent with the original IDE submission except for some re-organization for clarity (i.e., reordering and using numbers instead of bullets). Endpoint #5 (EQ-5D) was listed as an exploratory endpoint in the IDE submission. As per FDA's study design consideration (G210328/S001), EQ-5D has been moved to the secondary endpoint section and is now a multiplicity-controlled secondary endpoint.

3.1.5. Secondary Endpoint #1: Change in MADRS score

This endpoint is described within this section but is not considered one of the three endpoints multiplicity controlled.

3.1.5.1. Endpoint Definition

Secondary endpoint #1 is the adjusted mean change in group difference in the MADRAS scores between 10 weeks and baseline between subjects randomized to the active study device relative to those subjects randomized to sham.

3.1.5.2. Hypothesis Test

The formal statistical hypothesis is as follows:

H_o: $d_{flow} – d_{sham} \leq 0$
H_a: $d_{flow} – d_{sham} > 0$

Where d_{flow} and d_{sham} are the mean change in group difference in the MADRAS scores in subjects randomized to Active and Sham groups, respectively.

3.1.5.3. Analysis Set

The mITT population will be the analysis population used to analyze secondary endpoint #1. The endpoint will be analyzed when all randomized subjects have completed their 10-week visit, been withdrawn from the trial or passed the end of their 10-week follow-up visit window. The mean change in group difference in the MADRAS scores will be assessed using a mixed model for repeated measures (MMRM). The significance level for this endpoint will be determined using the Hochberg procedure, which is described in Section 3.2.5.1. Details on the statistical model for MMRM are also described in Section 4.

3.1.5.4. Missing Data Analysis

The mixed model for repeated measures (MMRM) allows for inclusion of patients with missing Week 10 values. If all subjects have at least one follow-up evaluation, the following MMRM will be utilized for the primary superiority test. If there are subjects without at least one post baseline assessment, the multiple imputation analysis summarized below will be the primary analysis method. Least squares estimated values from the MMRM will be reported for each MADRAS score and as such these estimates include implicit imputations of missing values.

3.1.6. Secondary Endpoint #2: Clinical Response

3.1.6.1. Endpoint Definition

Secondary endpoint #2 is the clinical response between subjects randomized to the active study device relative to those subjects randomized to sham. The three Clinical Response rates are defined as a >50% reduction from the baseline HDRS-17 and MADRS and MADRS-s score at Week 10.

The clinical response per HDRS-17 is controlled for multiplicity (see below). This endpoint is listed as #2a in the list of endpoints above. This endpoint based on HDRS-17 is the endpoint referred to as “Endpoint #2” in the Adaptive Design Report. The clinical response based on MADRS and MADRS-S are listed as endpoints #2b and #2c in the list of endpoints above. Those two endpoints will be provided with descriptive statistics.

3.1.6.2. Hypothesis Test

The formal statistical hypothesis is as follows:

H_o: $p_{flow} – p_{sham} \leq 0$

H_a: $p_{flow} – p_{sham} > 0$

Where p_{flow} and p_{sham} are the clinical responses in subjects randomized to Active and Sham groups, respectively.

3.1.6.3. Analysis Set

The mITT population will be the analysis population used to analyze secondary endpoint #2. The endpoint will be analyzed when all randomized subjects have completed their 10-week visit, been withdrawn from the trial or passed the end of their 10-week follow-up visit window. The endpoint will be tested using a two-group large-sample normal approximation test of proportions. The significance level for this endpoint will be determined using the Hochberg procedure, which is described in Section 3.2.5.1.

3.1.6.4. Missing Data Analysis

Multilple Imputation will be used through SAS PROC MI with age and sex as covariates along with longtiduinal responses from all other timepoints. The 20 imputations will be combined using Rubin's Rules through SAS PROC MIANALYZE.

3.1.7. Secondary Endpoint #3: Remission

3.1.7.1. Endpoint Definition

Secondary endpoint #3 is the difference in remission proportion between subjects randomized to the active study device relative to those subjects randomized to sham. The three remission rates will be analyzed as responder endpoints where remission is defined as an HDRS-17 score ≤7, a MADRS score ≤10, or MADRS-s score of ≤12.

Remission per HDRS-17 is controlled for multiplicity (see below). This endpoint is listed as #3a in the list of endpoints above. Remission based on MADRS and MADRS-S are listed as endpoints #3b and #3c in the list of endpoints above. Those two endpoints will be provided with descriptive statistics.

3.1.7.2. Hypothesis Test

The formal statistical hypothesis is as follows:

H_o: $p_{flow} – p_{sham} \leq 0$ H_a: $p_{flow} –$
$p_{sham} > 0$

Where p_{flow} and p_{sham} are the remission proportion in subjects randomized to Active and Sham groups, respectively.

3.1.7.3. Analysis Set

The mITT population will be the analysis population used to analyze secondary endpoint #3. The endpoint will be analyzed when all randomized subjects have completed their 10-week visit, been withdrawn from the trial or passed the end of their 10-week follow-up visit window. The endpoint will be tested using a two-group large-sample normal approximation test of proportions. The significance level for this endpoint will be determined using the Hochberg procedure, which is described in Section 3.2.5.1.

3.1.7.4. Missing Data Analysis

Multilple Imputation will be used through SAS PROC MI with age and sex as covariates along with longitudinal responses from all other timepoints. The 20 imputations will be combined using Rubin's Rules through SAS PROC MIANALYZE.

3.1.8. Secondary Endpoint #4: Quality of Life Improvement

3.1.8.1. Endpoint Definition

Secondary endpoint #5 is the quality of life improvement as measured by EQ-5D-3L in the active compared to sham arm at week 10. This is one of the three second endpoints controlled for multiplicity.

3.1.8.2. Hypothesis Test

The formal statistical hypothesis is as follows:

H_o: $d_{flow} – d_{sham} \leq 0$ H_a: $d_{flow} –$
$d_{sham} > 0$

Where d_{flow} and d_{sham} are the mean change in group difference in the EQ-5D-3L scores in subjects randomized to Active and Sham groups, respectively.

3.1.8.3. Analysis Set

The mITT population will be the analysis population used to analyze secondary endpoint #4. The endpoint will be analyzed when all randomized subjects have completed their 10-week visit, been withdrawn from the trial or passed the end of their 10-week follow-up visit window. The mean change in group difference in the EQ-5D-3L scores will be assessed using a mixed model for repeated measures (MMRM). The significance level for this endpoint will be determined using the Hochberg procedure, which is described in section 3.2.5.1.

3.1.8.4. Missing Data Analysis

The mixed model for repeated measures (MMRM) allows for inclusion of patients with missing Week 10 values. If all subjects have at least one follow-up evaluation, the following MMRM will be utilized for the primary superiority test. If there are subjects without at least one post baseline assessment, the multiple imputation analysis summarized below will be the primary analysis method. Least squares estimated values from the MMRM will be reported for each EQ-5D-3L score and as such these estimates include implicit imputations of missing values.

3.1.8.5. Additional analyses

EQ-5D will be provided using descriptive statistics including an analysis based on health states.

3.1.9. Multiplicity Control for Secondary Endpoints

Should the primary endpoint be achieved, the next endpoint that will be tested is the clinical response endpoint based on HDRS-17 50%. Should that endpoint be achieved, the remaining endpoints in this document will be tested using the methodology in this section.

The Sponsor understands that multiplicity correction and Type I error in general are not the most relevant or complete statistical quantities of interest in all statistical perspectives. As summarized by Vanderbilt University and frequent FDA Statistical Advisor, Dr. Frank Harrell.

Statisticians have convinced regulators that long-run operating characteristics of a testing procedure should rule the day, e.g., if we did 1000 clinical trials where efficacy was always zero, we want no more than 50 of these trials to be judged as ""positive"." Never mind that this type I error operating characteristic does not refer to making a correct judgment for the clinical trial at hand. Still, there is a belief that type I error is the probability of 'regulator's regret (a false positive), i.e., that the treatment is not effective when the data indicate it is. In fact, clinical trialists have been sold a bill of goods by statisticians. No probability derived from an assumption that the treatment has zero effect can provide evidence about that effect. Nor does it measure the chance of the error actually in question. All probabilities are conditional on something, and to be useful they must condition on the right thing. This usually means that what is conditioned upon must be knowable.

The probability of 'regulator's regret is the probability that a treatment 'doesn't work given the data. So the probability we really seek is the probability that the treatment has no effect or that it has a backwards effect. This is precisely one minus the Bayesian posterior probability of efficacy.[1]

The following strategy is specified to achieve two primary goals:

- Provide FDA pre-specified statistical quantities to enable a straightforward regulatory review of the PMA data.
- Provide comprehensive statistical summaries of the PMA data which exist beyond multiplicity adjusted p-values.

Multiplicity for the three secondary endpoints will be controlled using Hochberg correction implemented through the following algorithm:

3.1.9.1. Hochberg Algorithm

A Hochberg[2] "step-up" approach will be utilized to control the overall type I error at the desired 5% level across the three secondary efficacy analyses. To implement the Hochberg method, the p-values for the set of multiple null hypotheses are ordered from largest to smallest, and each p-value is compared to a sequentially decreasing alpha-level to determine whether the null hypothesis (and, potentially, subsequent hypotheses) should be rejected. Symbolically, for the set of p-values $\{p_1, \ldots p_k\}$ ordered from largest to smallest and testing the corresponding set of null hypotheses $\{H_{o1}, \ldots H_{ok}\}$, the Hochberg procedure is implemented as:

Step 1: Evaluate whether $p_1 < \alpha$. If yes, reject H_{o1} and all subsequent null hypotheses $\{H_{o2}, \ldots H_{ok}\}$. Else, do not reject H_{o1} and go to Step 2.

Step 2: Evaluate whether $p_2 < \alpha/2$. If yes, reject H_{o2} and all subsequent null hypotheses $\{H_{o3}, \ldots H_{ok}\}$. Else, do not reject H_{o2} and go to Step 3.

[…]

Step k: Evaluate whether $p_k < \alpha/k$. If yes, reject H_{ok}. Else, none of the null hypotheses $\{H_{o1}, \ldots H_{ok}\}$ are rejected and stop.

---

[1] Dr. Frank Harrell, p-values and Type I Errors are Not the Probabilities We Need, Last updated on 2020-09-15 and Accessed 2021-11-18, https://www.fharrell.com/post/pvalprobs/

[2] Hochberg Y. A sharper Bonferroni procedure for multiple tests of significance. Biometrika. 1988; 75:800–802

As only the smallest p-value is compared to the traditional Bonferroni-corrected α-level, the Hochberg method is more statistically powerful for controlling Type I error in the context of testing multiple null hypotheses. The Hochberg approach to Type I error control has been shown to be uniformly more powerful than the Bonferroni and Holm procedures and have non-restrictive distributional assumptions that are often met. Therefore, it is always preferred when hypotheses are not ordered.[3] Should the assumption that the endpoints are not negatively correlated not be met, a bootstrapping approach for multiplicity will be provided in addition to the Hochberg correction. The adjusted p-values from the Hochberg procedure will be considered the primary study results.

The specified multiplicity algorithm maintains overall Type I error at the desired α-level across a set of null hypotheses while also improving statistical power over other approaches, and importantly, allows for regulatory review of the secondary endpoints.

3.1.10. Exploratory Endpoints

The following list of secondary endpoints will be analyzed using the mITT population and will be summarized based on the type of data: Continuous (CONT), Binary (BIN) or Correlation (CORR).

In general, for all endpoints, the values over time (at each timepoint) and the change from baseline (at each timepoint) will be presented. The following list contains the specific endpoints that will be provided using descriptive tabular and graphical methods:

- Montgomery–Åsberg Depression Rating Scale (MADRS) response rate at all follow-up time points (CONT)
- Montgomery–Åsberg Depression Rating Scale - Self-report (MADRS-s) scores of the two arms (response and remission rates) at all time points (CONT)
- Hamilton Anxiety Rating Scale (HAM-A) at all time points (CONT)
- Young Mania Rating Scale (YMRS) at all time points (CONT)
- tDCS Adverse Events Questionnaire (AEQ) at 10 and 20 weeks (CONT)
- HDRS-17 (CONT), MADRS (CONT), MADRS-s mean decrease (CONT), remission (BIN), and response rate (BIN) at 6 weeks comparison between groups.
- HDRS-17 (CONT), MADRS (CONT), MADRS-s (CONT) at 20 weeks (after open-label)
- Correlation between MADRS and MADRS-s. (CORR)
- Correlation between the percent of sessions using the FLOW FL-100 and HDRS-17/MADRS decrease in the active group at 10 weeks. (CORR)

- The within-patient clinically meaningful improvement as defined as at least -3 points on the HDRS-17 scale. The percentage of subjects for each arm that reaches -3 points or more improvement.
- Cumulative Proportion of Responders Analysis- Cumulative Distribution Functions for the Change in HDRS-17 Score from Baseline to Primary end-point by arm will be provided as per FDA Guidance on reporting patient reported outcomes[4]

[3] Dmitrienko, A., & D'Agostino, R. (2013). Traditional multiplicity adjustment methods in clinical trials. Statistics in Medicine, 32(29), 5172–5218.
[4] PATIENT-FOCUSED DRUG DEVELOPMENT GUIDANCE PUBLIC WORKSHOP Incorporating Clinical Outcome Assessments into Endpoints for Regulatory Decision-Making; FDA Workshop- December 6, 2019

For continuous variables (CONT), results will be summarized with the numbers of observations, means, standard deviation, median, minimums and maximums. Differences between the two groups, where specified, will be summarized with the differences of the two means, and 95% confidence intervals for the difference between the means. These calculations will be done under the assumption that data for the two groups are independent and approximately normally distributed. The confidence interval for the difference of two means will be calculated under the assumption of unequal variances. If the asymptotic assumptions fail, then nonparametric summary statistics (medians, 25th and 75th percentile) may be displayed as an alternative.

For binary variables (BIN), results will be summarized with patient counts, percentages/rates. Differences between the two groups, where specified, will be summarized with the difference in percent and the Newcombe score 95% confidence interval for the difference of two percentages.

For correlation analysis, Spearman correlation will be used to assess the association between two continuous variables. The correlation coefficient and the 95% confidence intervals will be reported.

4. Details of the MMRM

Assessment of change from baseline in HDRS-17 will be evaluated at Week 10. As discussed above, a mixed model for repeated measures (MMRM) allows for inclusion of patients with missing Week 10 values. For example, patients dropping out after Week 2 or after Week 8 will be included in the primary effectiveness analysis.

The MMRM used to test the primary effectiveness hypothesis will include the baseline value for HDRS-17, a treatment group factor (Investigational or Sham), a categorical factor for visit, and treatment group by visit. The MMRM approach is a direct likelihood approach[5] requiring specialized statistical software. For this study, all MMRM parameters will be estimated using SAS PROC MIXED (SAS Institute, Cary NC Version 9.4 or higher). The MMRM model is notable for its ability to include all available outcome data from all subjects including drop-outs and does not require their exclusion as in complete case analysis or arbitrary assignment of some value as in last observation carried forward (LOCF). The MMRM generally includes a factor for group by visit interaction to allow treatment group differences in mean changes to vary over time. Inclusion of outcome data from earlier time points informs the implicit imputation of values missing at later time points (e.g., at Week 10) through the covariance matrix used to model the random effects.

In a matrix equation, the MMRM can be expressed as: $Y_i = X_i\beta + Z_iu + e_i$; where β is the vector of fixed-effect regression parameters (for the overall mean change, the treatment effect θ, a vector of post-baseline time effects τ, a vector of treatment-by-time interaction effects η, and a vector of covariate effects φ that includes baseline HDRS-17 and optionally, other a priori selected covariates. X is a design matrix for the fixed effects, Z is a design matrix used to account for other random effects u, if any were included. Key assumptions are about e, the random error vector. It is assumed that the expected values are zero, i.e., E(e) = 0. An unstructured covariance is assumed requiring estimation of variances at each visit and all pairwise covariances, i.e., Var (e)=$\sigma_e^2 V_{unstructured}$.

[5] Verbeke G. and Molenberghs G. Linear Mixed Models for Longitudinal Data. New York: Springer 2000.

The parameter estimates from this model will be used to construct an estimate of the treatment group difference in mean improvements at Week 10 between Investigational and sham. The advantages of MMRM have been outlined in Siddiuqui, Hung, and O'Neill (2009).[6]

"According to Rubin (1976) and Little and Rubin (2002), data are considered missing completely at random (MCAR) if, conditional upon the independent variables in the analytic model, missingness is independent of both unobserved and observed outcomes of the variable being analyzed; data are missing at random (MAR) if, conditional upon the independent variables in the analytic model, missingness depends on the observed data of the variable being analyzed but is independent of the unobserved outcomes of the variable being analyzed; data are missing not at random (MNAR) if, conditional upon the independent variables in the analytic model, the missingness depends on the unobserved outcomes of the variable being analyzed."

"A missingness mechanism is called "ignorable" if a likelihood-based analysis [such as MMRM] provides valid inferences of the model parameters even when the missingness mechanism is ignored; otherwise, it is called "nonignorable" missingness mechanism. Laird (1988) shows that MCAR and MAR are ignorable missingness, and likelihood-based analyses that ignore the missing data mechanism remain valid. For nonignorable missing data, however, likelihood-based analyses that ignore the missing data mechanism potentially produce biased results."

MMRM produces unbiased parameter estimates under MAR, while complete case analysis only produces unbiased estimates under the much more restrictive assumption of MCAR. That is, MMRM produces unbiased parameter estimates if conditional on the baseline HDRS-17 and the change scores up to the visit at which the patient drops out (and other baseline covariates, if any) the probability of missing is independent of the missing value itself. In contrast, MCAR requires that the probability of missing is unconditionally independent of the missing value itself, an assumption that is likely violated in practice. The MAR implicit imputation of missing values can be improved by including additional baseline or site effects that are associated with the probability of having a missing value. In contrast, "the LOCF approach is simple, but it makes two strong assumptions that: (i) missing data due to dropouts follow MCAR and (ii) the responses following a patient dropping out remain constant at the last observed value prior to drop out. Both of the assumptions are often unrealistic in clinical trials."

The handling of missing data is described in the next section.

5. Missing data

5.1. Procedures For Minimizing The Occurrence Of Missing Data

The preferred and often only fully satisfactory approach to addressing missing data is maximizing prevention. This trial defines the following proactive approaches to be implemented to minimize the number of patients who are not assessed at Week 10.

Among these approaches are the following:

[6] Siddiqui OS, Hung HMJ, and O'Neill R. MMRM vs LOCF: A comprehensive comparison based on simulation study and 25 NDA data sets. Journal of Biopharmaceutical Statistics,2009, 19: 227-246.

- The study protocol properly distinguishes between reasons for nonadherence (that is, for not receiving randomized therapy and hence for being "off study treatment") versus non-retention (that is, for not obtaining outcome information and hence for being "off study").
- The study protocol recognizes valid reasons for subject discontinuation of follow-up: subject withdrawal of consent, death, achievement of all required efficacy and safety endpoint information, or loss to follow-up.
- The term' withdrawal of consent' is used only when the patient no longer wishes to participate in the trial and no longer authorizes the investigators to make efforts to continue to obtain their outcome data.
- Patients are educated during the informed consent process about the continued scientific relevance of their data even if they discontinue treatment and the deleterious effect that missing data has on trial integrity and credibility.
- All patients will be followed until death or trial completion, even after discontinuing study treatment or initiating other treatment.
- It is recognized that protocol-specified increases in sample size to adjust for missing data address the influence of missingness on the precision of estimates, but not its influence on bias induced in those estimates.
- The protocol specifies performance standards to be met to achieve high quality of trial conduct, including specification of targeted levels of data capture.
- Creative and effective procedures are being implemented during enrollment and follow-up to enhance achieving pre-specified targeted retention levels.
- One of these procedures will be the engagement of investigators committed to following all patients until their death or capturing primary endpoint data, even if patients have discontinued randomized treatment or initiated other interventions.
- An oversight process by the sponsor is in place during trial conduct to ensure the achievement of performance standards, including targeted levels of data capture.

5.2 Data Collection For Subjects Who Discontinue From Randomized Treatment Or Discontinue From The Study

5.2.1. DISCONTINUATION DEFINITIONS AS PROVIDED IN THE PROTOCOL

In every instance where a subject does not complete the study, the Investigator will document the primary reason for discontinuation in the subject's records. In the case of early discontinuation of randomized treatment, the investigator will make all efforts to complete the final study assessments (unless contraindicated) before the subject's exit from the study.

5.2.2. DISCONTINUATION FROM STUDY BY SUBJECT

Subjects have the right to withdraw from the clinical investigation at any time and for any reason without prejudice to their future medical care by the investigation team or investigational site.

The Principal Investigator will ask the reason for a subject's withdrawal from the study and will record all information regarding the subject's discontinuation. If a subject expresses a desire to withdraw their consent for the study, the site should attempt to obtain written documentation for their study records.

Regardless of the reason for withdrawal, data available for the subject at the time of withdrawal, including the reason for withdrawal, will be entered on the Case Report Forms. A subject that has been withdrawn from the study after treatment will not be replaced.

5.2.3. DISCONTINUATION FROM RANDOMIZED TREATMENT BY INVESTIGATOR

A Subject may be withdrawn from randomized treatment by the Investigator for the following reasons:

- Any unanticipated adverse event that, in the Principal Investigator's opinion, is related to the treatment and will endanger the well-being of the subject if treatment is continued.
- Development of any intercurrent illness(es), infection or condition(s) that might interfere with the continuation of treatment.
- Non-compliance with the clinical investigation procedures deemed by the Principal Investigator to be sufficient to cause discontinuation of treatment.
- The investigator determines that continuing the randomized study treatment is not in the subject's best interest.
- Any other problem deemed by the Investigator to be sufficient to cause discontinuation from study treatment.

For subjects that discontinued from study treatment by the Investigator, all reasonable efforts should be made to ensure the assessment of the primary and key secondary endpoints. PROs will be collected in the event of an unscheduled visit; these will be included in the sensitivity analysis defined below.

5.2.4. TEMPORARY DISCONTINUATION BY SUBJECT

Not applicable in this study.

5.2.5. DISCONTINUATION OF INADVERTENTLY ENROLLED SUBJECTS

If the Sponsor or Investigator identifies a subject who did not meet enrollment criteria and was inadvertently treated, that subject will be followed. The subject will be asked to return for all protocol-specified visits and complete all assessments (unless contraindicated). Any eligibility violation would be captured as a protocol deviation in the database.

5.2.6. LOST TO FOLLOW-UP (LTFU)

For any nonresponsive subjects, site staff will perform and document a minimum of three attempts to contact them via phone and one attempt to reach them via certified mail. The staff will document the date and type of attempted communication.

5.2.7. USE OF DATA FROM EARLY DISCONTINUATION CASES

Study data collected previously for subjects who withdraw from the study or are LTFU will be included in the data analysis and Clinical Study Report.

5.3. SPECIFICATION OF THE ESTIMAND

Primary and key secondary efficacy data (e.g., HDRS-17 scores) will be captured using approaches that are creative and proper.

One important consideration is the handling of Discontinuation of randomized treatment due to AEs, lack of treatment effectiveness, or other reasons that may occur. These events have the potential to influence the study results. This section details how these will be handled and mitigated for this trial.

Such events are called "intercurrent events" in the FDA Draft Guidance E9(R1). "[I]ntercurrent events such as discontinuation or switching of treatment, or use of rescue medication, may in some circumstances render the later measurements of the variable irrelevant or difficult to interpret even when it can be collected." To provide specificity about the estimand, it is necessary to specify how data from subjects with intercurrent events will be handled.

E9(R1) discusses several approaches to handling data after an intercurrent event. A preferred approach to handling intercurrent events is to adopt the "Treatment Policy" estimand. This approach requires data collection after the intercurrent event. This approach enables preservation of the integrity of randomization and obtains a direct assessment of the clinically relevant comparison between the intention to deliver the experimental intervention vs. the intention to deliver the control intervention in the context of real-world supportive care.

Supportive analyses will be obtained using alternative approaches to handling intercurrent events. One of these is to incorporate the intercurrent event as a failure mode in a composite endpoint regarding clinically meaningful improvements, especially if this endpoint is assessed in a responder or time-to-event analysis.

Another approach to handling intercurrent events is the hypothetical approach to "envisage in which the intercurrent event would not occur." For example, the "treatment effect of interest might concern the outcome if the subsequent active treatment had not been administered." This is specified as the secondary estimand, see below.

This section describes the influence of the estimand of interest on the capture of primary outcome data. For this study, the estimand of interest is the improvement in HDRS-17 in subjects at Week 10 relative to baseline compared to Control.

5.3.1. PRIMARY ESTIMAND

The primary estimand of interest is the relative difference between investigational subjects and those who received the control treatment. All recorded scores in the database will be used regardless of additional therapy that may be sought during the course of the trial. All subjects who are missing Week 10 HDRS-17 will be included in the primary analysis through Multiple Imputation that attempts (defined below) to replicate what would have been captured had the patient been assessed on that date.

As described below, the subjects will be differentially imputed in a manner that considers what is known about the reason for the discontinuation from follow-up and what is known about the subject's outcome performance through the time of their discontinuation. The CRF will collect the specific reasons for discontinuing therapy to identify these subjects.

A sensitivity analysis will be provided for this estimand that uses LOCF prior to the discontinuation.

5.4. Handling Of Missing Data

Subjects lost to follow-up may produce bias in this study. Reasons for discontinuation from treatment and from follow-up will be evaluated and characterized by tabulating the reasons for missingness within the randomized treatment group overall and according to the study week. Any subject who is missing their primary Week 10 HDRS-17 evaluation will be included in the primary study assessment through Multiple Imputation (MI).

5.4.1. HANDLING DATA FOLLOWING SUBJECT WITHDRAWAL DUE TO LACK OF TREATMENT EFFECTIVENESS

Subjects who indicate, "Withdrawn due to lack of treatment effectiveness" and are missing the Week 10 evaluations will be imputed by replacing every subsequent follow-up visit with the baseline score. This methodology is understood to be a "Jump to Baseline." Provided that all treatments will be provided as medically necessary, there are expected to be very few subjects like this. Instead, subjects are expected to be recorded as having taken additional treatments but remain in the study.

5.4.2. HANDLING OF SUBJECTS REASONABLY MISSING AT RANDOM

Subjects who can be reasonably understood to be missing at random, that is, the reasons for missingness for subjects who appear pseudo-independent from their clinical status, for example, moved to a different state during the trial, will be imputed within their randomized treatment group.

5.4.3. HANDLING OF SUBJECTS WITH MISSINGNESS FOR ALL OTHER REASONS

This category likely would include most of the subjects with missing data for the primary endpoint and secondary endpoints at Week 10. With this approach, for each patient and each endpoint, the patients' actual trajectory for that endpoint will be used over the interval that it would be available, ideally from randomization through the time of their discontinuation from the trial. The remaining trajectory for that patient, for that endpoint, would be imputed using the trajectory for control patients over the window from their discontinuation from the trial (or from the last calendar time when it would be known for that patient) until the time of endpoint assessment, (i.e., Week 10). That control trajectory would be assumed to be piecewise linear between follow-up timepoints.

Given the expected high rate of follow-up achieved by the procedures for reducing missing data outlined above and given that the primary endpoint is evaluated at Week 10, very few subjects may be missing and eligible for this treatment of missing data. Pre-specified sensitivity analysis will provide additional insight into this handling of missing data; see below.

5.4.4. MULTIPLE IMPUTATION - GENERAL

If any patients are missing their primary Week 10 evaluation (or other earlier timepoint), multiple imputation (MI) (Rubin and Schenker 1991) will be used to create completed datasets appropriate for regulatory evaluation of the primary endpoint.

5.4.5. DEVELOPMENT OF MI MODEL

One of the key assumptions for a valid application of Multiple Imputation is that the probability of missingness is independent of the missing value itself, unconditionally or conditionally. If unconditionally, this situation is referred to as Missing Completely at Random or MCAR (Rubin 1976). If independence is conditional on observed data, the situation is referred to as Missing at Random or MAR. It is widely believed that MCAR is not a reasonable assumption, but MAR might be a reasonable assumption in many instances. If either MCAR or MAR holds, the missing mechanism is said to be ignorable (Little and Rubin 2002). Many conventional missing data strategies, including Mixed Models for Repeated Measures (MMRM) and Multiple Imputation (MI) require an ignorable missing value mechanism. When this assumption does not hold, the situation is referred to as Missing Not at Random, or MNAR. When MNAR holds, the application of MI is biased.

The situation is nearly as MNAR as any situation could get regarding subjects who discontinue treatment due to lack of efficacy. For this reason, subjects in both groups who discontinue due to lack of treatment effectiveness will be imputed using the Jump to Baseline methodology. For the subjects who are reasonably understood to be missing at random will be included in the MI model within their randomized treatment group. For the remaining subjects in both groups, missing values will be imputed from a model including only Control subjects. The covariates and model specifications are detailed in the following two sections.

5.4.6. COVARIATES IN THE MI MODEL

The covariates chosen for the MI model are:

- Sex
- Age
- Antidepressants use at baseline
- In Psychotherapy at baseline
- Baseline HDRS-17
- Longitudinal HDRS-17 scores

Should convergence issues arise during the modeling procedure, sex will be removed from the model, and only the continuous variables used.

5.4.7. CHOICE OF MI MODEL

The selected MI model will impute continuous primary endpoints over time. By imputing continuous HDRS-17 values over time, the secondary endpoints evaluating values over time and change from baseline may be analyzed using the same imputed dataset. This is expected to produce consistency and

coherence between the various inferential and descriptive statistics presented in the Clinical Study Report.

It is assumed that missing data may be non-monotonic. When missing data is non-monotonic, it is necessary to specify an appropriate modeling methodology to handle it. Further, because the specified covariates are both continuous and categorical, the choice of models additionally limited. For these reasons, the fully conditional specification (FCS) approach, as implemented in SAS PROC MI will be used to obtain 20 multiply imputed completed data sets. The FCS requires the specification of the predictive models for each variable.

Fully Conditional Specification (FCS) is an example of a "chained equations" approach. It is also known as "regression switching" approach. It is commonly referred to as MICE or Multivariate Imputation By Chained Equations. The FCS approach differs from other MI approaches in that it does not explicitly involve the joint distribution of the variables but focuses on the marginal distributions. It is like MCMC and Gibbs sampling in this regard, except that typically many fewer iterations are needed to determine the required distributions. The following describes the approach:

"The fully conditional approach to imputation is a more flexible method that does not rely on the assumption of multivariate normality (9, 11). Conditional distributions (regression models) are specified for each variable with missing values, conditional on all of the other variables in the imputation model. Imputations are generated by estimating each conditional distribution in turn, using observed cases for the variable being considered and imputed values for the other variables at that iteration and imputing missing values (again allowing for uncertainty in model parameters). The approach is appealing, since it does not restrict the conditional distributions to being normal, so that univariate regression models can be tailored appropriately—for example, using logistic regression for binary variables and ordered logistic regression for ordinal variables. However, it is possible for some of the conditional distributions to be incompatible with each other, potentially leading to unsound imputations (14). The FCS approach is also available in a number of statistical packages, including Stata (13), SAS (11), and R (21), and is increasingly appearing in practice (22, 23)." (Lee and Carlin 2010) See also Buuren SV. Flexible Imputation of Missing Data. New York: CRC Press 2012.

5.4.8. BASELINE COVARIATES

Subjects missing a baseline covariate (e.g., sex, age, or baseline HDRS-17) will have their baseline covariates within the FCS model.

5.4.9. LONGITUDINAL HDRS-17

An aim of this missing data modeling is to preserve the temporal nature of the longitudinal HDRS-17 values. To effectuate this:

1. For the model that imputes subjects presumed missing at random, HDRS-17 values at each timepoint will be included in the MI model. For each timepoint, the HDRS-17 values at all other timepoints, along with the baseline covariates will predict the missing values. This imputation is geared to incorporate subject-level clinical trajectories, which, as stated above, can theoretically provide more information and aid in the imputation.
2. For the model that impute the remaining subjects within the control group, the timepoint immediately prior to each visit along with covariates will predicted the missing value. That is,

   - Week 10 ~ Week 7 + covar_1 + covar_k
   - …
   - Week 1 ~ Baseline + covar_1 + covar_k

5.5. Sensitivity Analysis

5.5.1. ALTERNATIVE MODELING APPROACHES

Sensitivity analyses for handling missing data are specified for the Primary Endpoint, which will produce specific and unique estimates of success. The sensitivity analysis will calculate Week 10 change scores after LOCF is applied prior to their missing visit. Subjects who discontinue will have an additional PRO evaluation recorded during an unscheduled visit. When this value exists, it will be subject to this LOCF procedure and presumably add additional information.

6. Interim Analysis

An interim analysis will be performed when 90 subjects have week 10 data, which will include both a futility assessment and sample size re-estimation. The futility assessment will be based on stochastic curtailing approach by Lachin et al (2005). The sample size re-estimation will be based on a Promising Zone methodology by Mehta and Pocock (2011) with a Fuzzy design approach introduced by Keenan and Maislin (2014). SeparateAdditional details about the interim analysis are documented in an Adaptive Design Report [ref].

At a high level, the Interim analysis may modify the trial in two ways:

- Declare the trial futile and stop enrollment.
- Specify a number of subjects less than 270 that is needed for powering the trial.

7. Sample Size Assumptions and Calculation

The sample size calculation is based on data from Brunoni et al (2017). The calculation is based on a two-sample t-test for mean difference with the following assumptions:

- Group difference in means = 3.2
- Standard deviation of Investigational and Sham groups = 7.1 and 7.9, respectively
- One-sided type I error = 0.025
- 80% power

The resulting sample size is 176 subjects. To obtain an increase in statistical power (87.6%), 216 subjects were chosen. Accounting for 20% attrition, the overall sample size will be 270 subjects.

Based on the results of the interim analysis, the trial size may be adjusted in the following ways:

- If the trial is declared futile, enrollment will stop
- If less than 270 subjects are needed for statistically powering the study, the trial will only enroll up the the number of subjects specified by the Promising Zone technology (see the section on the Interim Analysis and the Adaptive Design Report)

8. Additional Analyses

8.1. Evaluation of Blinding

An evaluation of blinding will be performed. Prior to subjects unblinding through the system they will respond to the question “Which treatment arm do you think you are in?” (answers: sham/active), followed by the question “How certain are you of this?”, with the options 1 to 5 where 5 is very certain and 1 is very uncertain. The counts and percentages of the subjects responding correctly will be tabulated and reported.

8.2. Device Survival

Kaplan-Meier survival analysis will be used to characterize device failure time. Device failure in this study is defined as the device not operating as intended without unexpected failure. Unexpected failure is defined as a failure that is not related to device functionality. For example, the device accidentally falling out of a window would be categorized as an unexpected device failure. In contrast, the device failing to turn on would be considered an expected device failure. As the device is not expected to have any unexpected failures, unexpected failures will only be reported if they occur. Lifetables will be tabulated indicating the number of “failures" and the number of “at- risk” subjects over time for all failures, unexpected failures and expected failures. If there are more than one of each expected and unexpected failures, a log-rank test will be performed to compare the device failure time distribution.

8.3. Poolability Analysis

Poolability will be assessed through stratification of the primary endpoint by Country (OUS and US). Within country point estimates will be calculated along with nominal descriptive 95% Confidence Intervals. The focus of this analysis will be on the similarity of the country specific point estimates. Differences will be described in the Clinical Study Report.

Statistical evaluation of the primary endpoint will be evaluated by adding site by treatment group term to the primary MMRM model. A p-value of <= 0.10 will be considered significant for this poolability assessment. Should a significant site x treatment group interaction be present, the patient factors of age, sex, and baseline scores will be assessed to understand if differences in case-mix factors explain differences observed between sites.

8.4. Safety Analyses

Assessment of the safety of the investigational device will include an evaluation of the incidence and severity of complications and adverse reactions associated with the treatment.

Adverse event rates will be summarized by type of AE and for specific AEs in two ways:

1. per subject incidence of specific AEs and classes of AEs and
2. by event, summarizing event counts by visit interval over time and in accordance with FDA Guidance (CDRH 2004)[7].

Device and procedure-related events will be summarized by severity and relatedness. Events listings that include details such as relatedness, severity, onset, and resolution status will be provided for all events and relevant subsets of events such as serious events and related events. Primary safety comparisons

---

[7] CDRH. Guidance for Industry and FDA Staff: Clinical Data Presentations for Orthopedic Device Applications, December 2, 2004.

will be performed using the AT analysis set. The summary tables will show the adverse events (in coded terms), the total number of events, and the number and the percentage of subjects affected in each treatment group ("subject wise evaluation") and stratified by relation to device and severity. Data of dropouts will be presented separately, and possible bias will be discussed in the final report.

All AEs will be summarized in listings for the AT analysis set. The following AE listings will be constructed:

- AE Listing 1 All AEs Sorted by Group and Event Type
- AE Listing 2 All AEs Sorted by Group and Subject
- AE Listing 3 Serious AEs
- AE Listing 4 Severe AEs
- AE Listing 5 Device Related AEs
- AE Listing 6 Serious Device Related AEs
- AE Listing 7 Severe Device Related AEs
- AE Listing 8 Other AEs
- AE Listing 9 AEs among subjects discontinued early
- AE Listing 10 AEs among subjects who died

Clinical Study Protocol

Transcranial Direct Current Stimulation in Major Depressive Disorder: a Double-blind, Placebo-controlled, Randomized, Superiority Trial

Protocol Number: *FL001*

Version: 9

Date: 10Jan2023

Sponsor:

Flow Neuroscience AB

This document contains confidential information for use only by investigators participating in the clinical investigation. This document should be maintained in a secure location and should not be copied or made available for review by unauthorized personnel.

2 REVISION HISTORY

Version	Date	Summary of Changes
1.0	10 Nov 2021	First Version for IDE submission
2	26 Nov 2021	- Removed upper age limit.<br>- MADRS-s of 20 or above added to inclusion criteria.<br>- Improved references.<br>- Clarified that the participants must be living in England, Wales or Texas.<br>- Added optional early termination visit.
2	13 Dec 2021	Incorporation of FDA comments from IDE application<br>SDMT baseline moved to week 0 visit.
2	05 Jan 2022	- Change to PHQ-9 form MADRS-s for the first screening assessment after consent.<br>- PHQ-9 of 10 points or above added to inclusion criteria.<br>- MADRS-s of 20 or above removed from inclusion criteria.
3	19Feb2022	- Inclusion criteria 12 update age to ≥ age 18<br>- Exclusion criteria 28 updated to: Are a chronic tobacco smoker, as defined by smoking by smoking >100 cigarettes (including hand-rolled cigarettes, cigars, cigarillos, etc.) in their life-time and have smoked every day for the last 7 days.
4	23Feb2022	- Updated exclusion criteria 31 to: Are participating concurrently in another clinical investigation or have participated in a clinical investigation within the last 90 days or intend to Participate in another clinical investigation during the study, and where the participation in the other investigation might interfere with the results of this trial as deemed by the PI.<br>- Clarified that for inclusion criteria 11 the age is 18 for UK and 22 for the US.
5	16Mar2022	- Updated device traceability (section 8.3) to reflect that UK participants are allowed to keep the device after trial completion.
6	04Apr2022	- Removed PHQ-9 from screening.<br>- Clarified which AEs will be followed-up in more detail. Anticipated AEs will be recorded using AEQ.<br>- Moved some sections regarding how missing data is handled in the statistical analysis plan to a new document (Statistical analysis plan - FL001).<br>- Site staff will consent subjects not Curebase staff.

7	31Aug2022	- Clarified insomnia & sleep apnea as an exclusion criteria, added eating disorders.<br>- Changed some excl. criteria based comorbidities to not be life-long.<br>- Clarified primary endpoint as requested by FDA IDE feedback.
8	07Dec2022	- Added reference to adaptive design report (22.15)<br>- Updated primary endpoint formulation again as requested by the FDA.<br>- EQ-5D-3L is now a secondary endpoint as requested by the FDA.<br>- Added within-patient clinically meaningful change exploratory endpoint.<br>- Added clarification of when the randomization happens (22.4)<br>- Added section 13.4 describing routines regarding video calls, as requested by the FDA.
9	10Jan2023	- Removed need for CRO representative to sign this protocol since they are not involved in the development of this protocol.

3 INVESTIGATOR PROTOCOL SIGNATURE PAGE

The signature below documents the receipt and review of the Study Protocol and any attachments and provides the necessary assurances that this study will be conducted according to all stipulations of the protocol, including all statements regarding confidentiality, and according to the local legal and regulatory requirements and applicable United States Federal Regulations, ISO 14155:2020, ICH and GCP guidelines, and the latest version of the Declaration of Helsinki.

I have read and understand this protocol and will conduct the study in accordance with this protocol, all attachments and amendments, applicable Food and Drug Administration regulations, HIPAA, IRB/EC requirements, and the policies of the institutions where the study will take place.

In my formal capacity as Investigator, my duties include ensuring the safety of the study subjects enrolled under my supervision and providing Flow Neuroscience AB with complete and timely information, as outlined in the protocol. It is understood that all information pertaining to the study will be held strictly

Protocol Number: FL001   Protocol Version: 9   Protocol Date: 10jan2023

Protocol Name: Transcranial Direct Current Stimulation in Major Depressive Disorder: A Double-blind, Placebo-controlled, Randomized, Superiority Trial

Investigator:

____
(Print Name)

____
(Signature)   Date (dd/mmm/yyyy)

____
Sponsor Representative Name

____
Sponsor Signature   Date (dd/mmm/yyyy)

____
Legal Representative Name

____
Legal Representative Signature   Date (dd/mmm/yyyy)

Upon signing, send a copy of this page to Curebase and retain a copy for your files.

TABLE OF CONTENTS

1 TITLE PAGE 1
2 REVISION HISTORY 2
3 INVESTIGATOR PROTOCOL SIGNATURE PAGE 5
4 LIST OF ABBREVIATIONS 10
5 Study Summary 12
6 Schedule of Procedures 19
7 Introduction 21
7.1 Background 21
7.1.1 Major Depressive Disorder (MDD) 21
7.1.2 MDD Diagnosis 21
7.1.3 Other Treatment Options 21
7.1.3.1 Antidepressants 21
7.1.3.2 Psychological Treatments 23
7.1.3.3 Electroconvulsive Therapy (ECT) 23
7.1.3.4 Transcranial Magnetic Stimulation (TMS) 24
7.1.3.5 Cranial Electrotherapy Stimulation (CES) 24
7.1.3.6 Transcranial Direct Current Stimulation (tDCS) 24
7.2 Investigational Device 25
7.2.1 Flow FL-100 25
7.2.2 The Flow app 26
7.3 Device Traceability 28
7.4 Supply and Accountability of Investigational Devices 29
7.5 Device Use Experience and Training 29
8 Benefit-Risk Analysis 29
8.1 Risks 29
8.2 Risk Mitigation 30
8.2.1 Monitors 30
8.2.2 Stimulation Parameter Space 31
8.2.3 Usage Limits 31
8.3 Anticipated Benefits 31
8.4 Risk-to-Benefit Ratio 31
9 Study Objectives 31
9.1 Background 31
9.2 Primary Objective 31
10 Study Design 32
10.1 Study Duration 32
10.2 Treatment/Control Groups 32
10.3 Blinded phase 33
10.4 Open label phase 33

10.5 Primary Endpoints 34
10.6 Secondary Endpoints 34
10.7 Exploratory Endpoints 34
11 Selection of Study Population 34
11.1 Participant Recruitment 34
11.2 Inclusion Criteria 35
11.3 Exclusion Criteria 36
11.4 Study Enrollment Procedures 37
11.4.1 Participant Enrollment 38
11.5 Screen Failures 38
11.6 Participant Treatment Assignment 38
12 Study Procedures 39
12.1 Week -3 to -1: Informed Consent (ICF) 39
12.2 Week 0 40
12.3 Weeks 1, 4 & 7 40
12.4 Week 10 40
12.5 Week 20 Follow-Up Visit - Open-Label Only 41
12.6 Video calls 42
12.7 Late and Missed Visits 42
12.8 Unscheduled Visits 42
12.9 Study Completion/Discontinuation 43
13 Description of Study Procedures 43
14 Early Discontinuation: Participants 45
14.1 Technical Failures 45
14.2 Treatment Failures 45
14.3 Lost to Follow-Up (LTFU) 45
14.4 Withdrawals 45
14.5 Documentation of Early Discontinuation and Pregnancy 46
14.6 Use of Data from Early Discontinuation Cases 47
14.7 Ongoing Treatment for Early Discontinuation Cases 47
15 Early Discontinuation: Study 47
15.1 Procedure for Suspension or Early Termination 47
15.2 Procedure for Resuming the Clinical Investigation After Temporary Suspension 48
15.3 Routine Close-Out 48
16 Protocol Deviations 48
17 Concomitant Medications/Therapies 48
18 Adverse Event & Device Deficiency Reporting 48
18.1 Definitions 49
18.2 Classification of an AE 51
18.2.1 Severity 51

18.2.2 Assessment of Relationship to Use of the Investigational Device or Procedure 52
18.2.3 SAE Relationship Assessment 52
18.2.4 SAE Reporting 53
18.2.5 Determination and Reporting of Unanticipated Adverse Device Effects (US Sites) 54
18.3 Treatment of AEs 55
18.4 Safety Oversight: Clinical Events Committee (CEC) 55
18.5 Safety/Suicide Risk Management Plan 55
19 Stopping rules 55
20 Clinical Monitoring 56
20.1 Site Monitoring 56
20.2 Monitoring Activities 57
20.3 Frequency of Visits 58
20.4 Audit and Inspections 58
21 Statistical analysis 58
21.1 Study Hypothesis 58
21.2 Sample Size Assumptions and Calculations 58
21.3 Study Populations 59
21.3.1 Intent-to-Treat Analysis Set (ITT) 59
21.3.2 As Treated Analysis Set (AT) 59
21.3.3 Modified Intent-to-Treat Analysis Set (mITT) 59
21.3.4 Per Protocol Analysis Set (PP) 59
21.4 Randomization 60
21.5 Testing of Primary Effectiveness Hypotheses 60
21.5.1 Multiple Imputation Analysis 60
21.6 Missing Data/Sensitivity Analysis 60
21.7 Covariates/Covariate Analysis 61
21.8 Secondary Effectiveness Endpoints 61
21.9 Exploratory Endpoints 61
21.10 Multiplicity Adjustment 62
21.11 Evaluation of Blinding 62
21.12 Device Survival 62
21.13 Poolability Analysis 63
21.14 Safety Analyses 63
21.15 Interim Analyses 64
22 Ethics & Protection of Human Subjects 64
22.1 Ethical Review 64
22.2 Regulatory Considerations 64
22.3 Study Amendments 64
22.4 Insurance 64
23 Data Handling and Recordkeeping 65

23.1 EDC and eCRFs 65
23.2 Capture of patient reported outcomes 65
23.3 Study Records Retention 65
23.4 Publication and Data Sharing Policy 66
23.5 Participant and Data Confidentiality 67
23.6 Investigator Records 67
23.7 Participant Records 67
23.8 Investigator Records and Reports 68

4 LIST OF ABBREVIATIONS

**ADE**	Adverse Device Effect
**AE**	Adverse Event
**AEQ**	tDCS Adverse Events Questionnaire
**ATHF**	Antidepressant Treatment History Form
**ATT**	Average Treatment effect on the Treated
**CA**	Competent Authority
**CABG**	Coronary Artery Bypass Graft
**CDC**	Center for Disease Control and Prevention
**CEC**	Clinical Events Committee
**CES**	Cranial Electrotherapy Stimulation
**CFR**	Code of Federal Regulations
**CHF**	Coronary Heart Failure
**CMP**	Clinical Monitoring Plan
**CRO**	Contract Research Organization
**ClinRO**	Clinician Reported Outcome
**C-SSRS**	Columbia Suicide Severity Rating Scale
**DBS**	Deep Brain Stimulation
**DHHS**	U.S. Department of Health and Human Services
**DLPFC**	Dorsolateral Prefrontal Cortex
**DMP**	Data Management Plan
**EC**	Ethics Committee
**eCRF**	Electronic Case Report Form
**ECT**	Electroconvulsive Therapy
**EDC**	Electronic Data Capture
**EOB**	Explanation of Benefits
**FDA**	U.S. Food and Drug Administration
**GCP**	Good Clinical Practice
**HAM-A**	Hamilton Anxiety Rating Scale
**HDRS-17**	Hamilton Depression Rating Score
**HIPAA**	Health Insurance Portability and Accountability Act
**ICF**	Informed Consent Form
**ICH**	International Conference on Harmonization of Technical Requirements for Registration of Pharmaceuticals for Human Use
**IDE**	Investigational Device Exemption
**IFU**	Instructions for Use
**iOS**	iPhone Operating System
**IRB**	Institutional Review Board
**ISF**	Investigator Site File

**ITT**	Intent-to-Treat
**LB**	Lower Bound
**LTFU**	Lost to Follow-Up
**MADRS**	Montgomery–Åsberg Depression Rating Scale
**MADRS-s**	Montgomery–Åsberg Depression Rating Scale - Self-report
**MAOIs**	Monoamine oxidase inhibitors
**MDD**	Major Depressive Disorder
**MI**	Multiple Imputation
**MINI**	Mini International Neuropsychiatric Interview
**NASSA**	Noradrenaline and Specific Serotonergic Antidepressant
**mITT**	Modified Intent to Treat
**NRS**	Numeric Rating Scale
**NSAIDS**	Nonsteroidal anti-inflammatory drugs
**OCD**	Obsessive-Compulsive Disorder
**OHRP**	U.S. Office of Human Research Protections
**PMA**	Premarket Approval
**PP**	Per-Protocol
**PRO**	Patient Reported Outcome
**PS**	Propensity Score
**QOL**	Quality of Life
**RAVLT**	Rey Auditory Verbal Learning Test
**SAE**	Serious Adverse Event
**SAP**	Statistical Analysis Plan
**SDMT**	Symbol-Digit Modalities Test
**SDW**	Source Document Worksheet
**SMD**	Standardized Mean Differences
**SNRI**	Serotonin and Noradrenaline Reuptake Inhibitor
**SOC**	Standard of Care
**SSRI**	Selective Serotonin Reuptake Inhibitor
**SOP**	Standard Operating Procedure
**TAQ**	Treatment Acceptability Questionnaire
**TCA**	Tricyclic Antidepressant
**tDCS**	Transcranial Direct Current Stimulation
**TEM**	Treatment-Emergent Mania/hypomania
**TMS**	Transcranial Magnetic Stimulation
**UADE**	Unanticipated Adverse Device Effect
**UB**	Upper Bound
**WHO**	World Health Organization
**YMRS**	Young Mania Rating Scale

5 Study Summary

Study Full Title<br><br>Study Sponsor<br>Study Number	Transcranial Direct Current Stimulation in Major Depressive Disorder: A Double-blind, Placebo-controlled, Randomized, Superiority Trial<br><br>Flow Neuroscience AB<br>FL001
Device Name	Flow FL-100
Protocol Date	10Jan2023
STUDY OVERVIEW	
Study Design<br><br>Purpose	Double-blind, placebo-controlled, randomized, superiority, remote.<br>The purpose of this trial is to investigate the use of the Flow FL-100 device at-home to treat Unipolar Major Depressive Disorder (MDD) in a double-blind RCT.
Expected Study Duration	10 months
Evaluation Schedule	Participants will undergo evaluations at the following study visits. Various evaluations regarding depression severity, suicidal ideation will be performed (see Schedule of Evaluations for more detail):<br><br>● Pre-Treatment Phase<br>– Week -3<br>– Week -2<br>– Week -1<br>● Blinded Phase<br>– Week 0<br>– Week 1<br>– Week 4<br>– Week 7<br>– Week 10<br>● Open-Label Phase<br>– Week 20<br><br>At week 0, 1, 4, 7, 10, 20, participants will also complete the MADRS-s on their personal smartphone or other device running Android 5.0+ or iPhone Operating System (iOS) 12+.
ELIGIBILITY CRITERIA	

Intended Participant Population	Up to 270 participants will be randomized at 2 sites, one located in the United Kingdom and the other in the United States. The recruitment will be capped to 155 for each of the sites.<br><br>1:1 randomization: Flow FL-100 vs. Sham
Diagnosis and Inclusion Criteria	To be eligible to participate in this study, participants must meet all the following criteria:<br><br>1. Be ≥18 years.<br>2. Have a diagnosis of Unipolar MDD with a current depressive episode as defined by the diagnostic criteria in the Diagnostic and statistical manual of mental disorders – 5th edition (DSM-V)<br>3. Have a Hamilton Depression Rating Score (HDRS-17) of ≥ 16.<br>4. For 6 weeks prior to enrollment, are either:<br>a. not taking antidepressant medication or:<br>b. are taking a stable antidepressant regimen with a stable medication source and agree to continue the same regimen throughout study participation.<br>5. If in psychotherapy, have maintained stable psychotherapy for at least 6 weeks prior to enrollment.<br>6. Have access to a stable internet connection through which the treatment will be received.<br>7. Have access to a smartphone or other device running Android 5.0+ or iPhone Operating System (iOS) 12+ (e.g., reasonably new iPhone/iPad or Android phone), used to using the device in their everyday life, and can capably use the study application on the device, as determined by the investigator.<br>8. Are currently living in England/Wales (UK) or Texas (US).<br>9. Subject is currently under the care of a psychiatrist or a primary care physician, agrees to be evaluated at regular intervals by a psychiatrist or primary care physician for the duration of study participation, and agrees to promptly inform the study staff of any change of psychiatric or mental health providers during study participation.

	10. Subject agrees to allow any and all forms of communication between the investigators/study staff and any healthcare provider who currently provides and/or has provided service to the patient/subject within at least two years of study enrollment.<br>11. Subject agrees to provide the name and verifiable contact information (email and mailing addresses, mobile and land-line phone numbers, as applicable) of at least two persons ≥ age 18 (22 in the US) who reside within a 60-minute drive of the patient's residence and whom the research staff is at liberty to contact, as they deem necessary, for the duration of study participation.<br>12. Be able to give voluntary, written informed consent to participate and have signed an Informed Consent Form specific to this study.<br>13. Be willing and able to comply with all study procedures.<br>14. Subject agrees to meet all of the inclusion criteria throughout their participation in the study. Otherwise, the subject will be discontinued from the study.<br>15. Subject agrees to a Safety/Suicide Risk Management Protocol, which is intended to reduce the reduce the risk of suicide during study participation.
Exclusion Criteria	Participants who meet any of the following criteria will be excluded from participating in this study:<br><br>1. Are in a current state of mania, as determined by the YMRS or psychosis, as determined by the MINI.<br>2. Are diagnosed with vitamin or hormonal deficiencies that may mimic mood disorders, as determined by the investigator.<br>3. Are currently receiving any other interventional therapy for MDD other than a stable regimen of antidepressants or psychotherapy as defined in the inclusion criteria.<br>4. Considered to have treatment resistant depression as defined by inadequate clinical response to 2 or more trials of antidepressants at an adequate dose and duration.<br>5. Have a history of electroconvulsive therapy (ECT), transcranial magnetic stimulation (TMS), cranial

	electrotherapy stimulation (CES), transcranial direct current stimulation (tDCS), deep brain stimulation (DBS), or other brain stimulation.<br>6. Patient answers Yes to Questions 4, 5 or 6 on the Columbia Suicide Severity Rating Scale (C-SSRS) Triage and Risk Identification Screener.<br>7. Any previous hospitilization for suicidal behavior.<br>8. Have chronic severe insomnia (< 4 hours of sleep each night), or depression secondary to chronic insomnia or sleep apnea.<br>9. Have any structural lesion (e.g., any structural neurological condition, or more subcortical lesions than would be expected for age or have had a stroke that affects stimulated area or connected areas) or any other clinically significant abnormality that might affect safety, study participation, or confound interpretation of study results, as determined by the investigator.<br>10. Have any implant in the brain (e.g., DBS) or neurocranium, or any other active implantable medical device.<br>11. Have any neurocranial defect.<br>12. Have a history of epilepsy or seizures (including history of withdrawal / provoked seizures).<br>13. Have shrapnel or any ferromagnetic material in the head.<br>14. Have any disorder that would impair the ability to complete the study questionnaires.<br>15. Have been diagnosed with autism spectrum disorder.<br>16. Are actively abusing substances (<1 week prior to enrollment).<br>17. Have a cognitive impairment (including dementia).<br>18. Have a history of mania or psychosis.<br>19. Are currently using any medications that affect cortical excitability (e.g., benzodiazepines, epileptics, etc.).<br>20. Are currently experiencing symptoms of withdrawal from alcohol or benzodiazepines.<br>21. Have been diagnosed with Parkinsonism or other movement disorder as determined by the investigator to interfere with treatment.

	22. Have ever taken esketamine / ketamine for treatment of depression.<br>23. Have ever been admitted to hospital for depression.<br>24. Have ever been diagnosed with obsessive-compulsive disorder (OCD) or bipolar type 1 or 2 disorder.<br>25. Is diagnosed with an active primary anxiety disorder, or PTSD, agoraphobia, anorexia or bulimia, panic or personality disorder with active symptoms.<br>26. Have a history of psychosurgery for depression.<br>27. Have any history of myocardial infarction, coronary artery bypass graft (CABG), coronary heart failure (CHF), or history of other cardiac issues.<br>28. Are currently experiencing or have a history of intractable migraines.<br>29. Are a chronic tobacco smoker, as defined by smoking by smoking >100 cigarettes (including hand-rolled cigarettes, cigars, cigarillos, etc.) in their life-time and have smoked every day for the last 7 days.<br>30. If female and of child-bearing potential, currently pregnant or breastfeeding or planning to become pregnant or breastfeed any time during the study.<br>31. Are currently a prisoner.<br>32. Are participating concurrently in another clinical investigation or have participated in a clinical investigation within the last 90 days or intend to Participate in another clinical investigation during the study, and where the participation in the other investigation might interfere with the results of this trial as deemed by the PI.<br>33. Have any medical condition or other circumstances, in the judgment of the investigator, that might interfere with the ability to complete follow-up visits and the self-reported MADRS-s in the app.<br>34. Have any condition which, in the judgment of the Investigator, would preclude adequate evaluation of the device's safety and performance.<br>35. A Subject who meets any of the exclusion criteria during study participation will be discontinued from the study.

FL-100 treatment sessions	FL-100 will be used by patients remotely for five sessions per week for 3 weeks, followed by three sessions per week for 7 weeks. In total, thirty-six sessions over 10 weeks during the blinded phase.
STUDY ENDPOINTS	
Primary Endpoint	The primary effectiveness endpoint is the adjusted mean group difference in the HDRS-17 scores at 10 weeks compared to baseline for subjects in the Active device and the Sham device groups.
Secondary Endpoints	• Arm difference in HDRS-17 response and remission rate at week 10.<br>• Arm difference in MADRS average score change, and response and remission rate at week 10.<br>• Arm difference in MADRS-s average score change, and response and remission rate at week 10.<br>• Quality of life improvement as measured by EQ-5D-3L in the active compared to sham arm at week 10.
Exploratory Endpoints	• Young Mania Rating Scale (YMRS) at 10 weeks.<br>• tDCS Adverse Events Questionnaire (AEQ) at 10 and 20 weeks<br>• Hamilton Anxiety Rating Scale (HAM-A) change at week 10.<br>• HDRS-17, MADRS, MADRS-s mean decrease, and remission and response rate at 20 weeks (after open-label) compared to open-label start and baseline.<br>• Correlation between MADRS and MADRS-s.<br>• Correlation between adherence to stimulation and HDRS-17/MADRS decrease in the active group at 10 weeks.<br>• Neuropsychological measure: verbal learning (Rey Auditory Verbal Learning Test (RAVLT) and information processing speed (Symbol-Digit Modalities Test (SDMT)<br>• Treatment acceptability questionnaire (TAQ) as developed by UEL. There is one form at baseline and another at week 10 and 20.<br>• The within-patient clinically meaningful improvement as defined as at least -3 points on the HDRS-17 scale. The percentage of subjects for each arm that

	reaches -3 points or more improvement and also the Cumulative Distribution Function Curves of Change in HDRS-17 Score from Baseline to Primary end-point by arm.
STATISTICAL CONSIDERATIONS	
Sample Size	270
Statistical Plan	A modified intent to treat will be used. Users who have performed less than 10 sessions (300 minutes) during the first 3 weeks will be excluded from the analysis.
STUDY MANAGEMENT	
Principal Investigator UK	Professor of Affective Neuroscience Cynthia Fu, MD, PhD, University East London
Principal Investigator US	Associate Professor of Psychiatry Sudhakar Selvaraj, MD, PhD, University of Texas
Clinical Research Organization (CRO)	Curebase, Inc.

6 Schedule of Procedures

Table 1. Schedule of Procedures. Activities in blue cells are performed by trained graduate researchers supervised by a trained psychiatrist, and red cells are to be completed by the participant.

Activity	Pre-Treatment	Blinded Phase					Open-Label Phase		Early termination[8]
	Week -3 to -1 (± 3 days)	Week 0	Week 1 (± 3 days)	Week 4 (± 3 days)	Week 7 (± 3 days)	Week 10 (± 3 days) Final blinded visit[1]	Week 10 (± 3 days) Open Label Start	Week 20 (± 3 days)	Week 1-20
Informed Consent	X								
Screening and baseline Video Call	X								
Diagnostic Assessment for MDD	X[9]								
Intervention Kit Shipped to subject	X[5]								
Randomization[6]		X							
Technical onboarding		X							
Initial Visit		X							
Video call	X	X	X	X	X	X		X	X
MINI	X								
HDRS-17	X		X	X	X	X		X	X
MADRS	X		X	X	X	X		X	X
MADRS-s		X[4]	X	X	X	X		X	X
C-SSRS	X		X	X	X	X		X	X
YMRS	X		X	X	X	X		X	X
HAM-A	X					X		X	X
EQ-5D-3L	X					X		X	X
RAVLT	X					X		X	X
SDMT		X[2]				X[2]		X[2]	
TAQ	X					X		X	X
AEQ						X		X	X
Healthcare Visit Survey[3]						X		X	X
FLOW FL-100 use[7]		X	X	X	X	X	X	X	
Record/Review Concomitant Medications	X	X	X	X	X	X		X	X
Record/Review Adverse Events		X	X	X	X	X		X	X

[1] The week 10 visit includes the study Primary end-point. This will be the final blinded visit and will be the start of the open label phase of the study. Participants in the Sham arm will be informed of their assignment and will restart with active stimulation for 10 weeks; participants in the Flow FL-100 arm will be allowed to continue maintenance treatment for 10 more weeks at 3 sessions per week. The unblinding will occur after the primary end-point data has been recorded.
[2] Optional
[3] Participants will be surveyed about the number of times he/she has sought healthcare due to depression during the previous 10 weeks.
[4] Participants will be required to answer the nine questions of the MADRS-s form in the app to start the first stimulation session during the technical onboarding.
[5] Participants will be required to start treatment within 24 days from completing the baseline surveys.
[6] Completed after eligibility is confirmed by the investigator; when the participant signs up with their email in the trial app they will automatically be randomized to the sham or active treatment arm (with 50% probability).
[7] Five sessions per week for 3 weeks, followed by three sessions per week for 7 weeks. In total, thirty-six sessions during 10 weeks during the blinded phase. Please refer to Table 2 and Table 3 below for more details.
[8] In case there is an early termination the research staff can request to have an early termination interview, if the subject agrees.
9 Diagnostic Assessment for MDD will be performed by Investigator-Psychiatrist according to the most recent APA Practice Guideline for treatment of MDD.

Table 2. Stimulation schedule during blinded phase.

**Week**	**1**	**2**	**3**	**4**	**5**	**6**	**7**	**8**	**9**	**10**
**Number of sessions**	5	5	5	3	3	3	3	3	3	3

Table 3. Stimulation schedule during open-label phase.

**Week**	**Previous assignment**	**1**	**2**	**3**	**4**	**5**	**6**	**7**	**8**	**9**	**10**
**Number of sessions**	Active	3	3	3	3	3	3	3	3	3	3
	Sham	5	5	5	3	3	3	3	3	3	3

7 Introduction

7.1 Background

7.1.1 Major Depressive Disorder (MDD)

Major depressive disorder (MDD) is a common psychiatric condition, mostly treated with antidepressant drugs, which are limited for issues such as effectiveness and adverse effects.

7.1.2 MDD Diagnosis

The diagnosis of MDD is based on the diagnostic criteria in the Diagnostic and statistical manual of mental disorders – 5th edition (DSM-V). Patients should receive a thorough diagnostic assessment in order to establish the diagnosis of major depressive disorder, identify other psychiatric or general medical conditions that may require attention. The psychiatrist should develop a comprehensive plan for treatment. This evaluation generally includes a history of the present illness and current symptoms that may trigger or exacerbate depressive symptoms. Several clinically validated standard scales are available for the assessment of the severity of depressive symptoms. Based on cut-offs from these scales, the severity of MDD can be classified as mild, moderate, and severe.

7.1.3 Other Treatment Options

7.1.3.1 Antidepressants

Antidepressants are psychiatric drugs used for the treatment of MDD and other conditions, including dysthymia, anxiety disorders, OCD, eating disorders, chronic pain, neuropathic pain and, in some cases, dysmenorrhea, attention-deficit hyperactivity disorder, addiction, and sleep disorders. They may be prescribed alone or in combination with other medications. The Center for Disease Control and Prevention (CDC) reported that antidepressant usage in the United States among people aged 12 or more years rose by 400% between 1988-1994 and 2005-2008. Antidepressants are given to improve depressive symptoms.

There are several types of antidepressants, depending to the main neurotransmitter system they target:

- Selective Serotonin Reuptake Inhibitors (SSRIs) and Serotonin and Noradrenaline Reuptake Inhibitors (SNRIs): SSRIs and SNRIs block the reuptake of serotonin or serotonin and noradrenaline in the brain. SSRIs are more widely prescribed than SNRIs, are effective in treating depression, and have fewer side-effects than older antidepressant classes [1]. One of the largest studies on MDD to date, the STAR*D study, showed remission rates up

[1] Anderson HD, Pace WD, Libby AM, West DR, Valuck RJ. Rates of 5 common antidepressant side effects among new adult and adolescent cases of depression: a retrospective US claims study. Clin Ther. 2012 Jan;34(1):113-23. doi: 10.1016/j.clinthera.2011.11.024.

to 28% with SSRIs alone [2]. SSRIs and SNRIs may have the following side effects: hypoglycemia, low sodium, nausea, rash, dry mouth, constipation, diarrhea, weight loss, sweating, tremor, sedation, sexual dysfunction, insomnia, headache, dizziness, anxiety, agitation, and abnormal thinking.

- Monoamine oxidase inhibitors (MAOIs): MAOIs inhibit the action of monoamine oxidase, the enzyme that breaks down monoaminergic neurotransmitter (e.g., serotonin, noradrenaline), thus increasing their levels in the brain. MAOIs are usually prescribed as second-line antidepressants as they present several side effects including blurred vision, rash, seizures, edema, weight disturbances, sexual dysfunction, diarrhea, nausea, constipation, anxiety, insomnia, drowsiness, headache, dizziness, arrhythmia, fainting and hypertension.
- Noradrenaline and Specific Serotonergic Antidepressants (NASSAs): This class of antidepressants block adrenergic and specific serotonergic receptors. NASSAs have the following side effects: constipation, dry mouth, weight gain, drowsiness, sedation, blurred vision, and dizziness. More serious adverse reactions include seizures, white blood cell reduction, fainting, and allergic reactions.
- Tricyclic Antidepressants (TCA): This is the oldest class of antidepressants which are not typically used as first-line treatment because they have a less favorable tolerability and safety profile than more recent antidepressants. TCAsmay have the following side effects: seizures, insomnia, anxiety, arrhythmia, hypertension, rash, nausea, vomiting, abdominal cramps, weight loss, constipation, urinary retention, eye pressure, and sexual dysfunction.

Current practice for selecting pharmacological antidepressant treatments are limited by low response and remission rates in MDD. The UK National Institute for Health and Care Excellence (NICE) CG90 guidelines[3] indicate that antidepressants should not be routinely used for the initial treatment of mild depression, because the risk-benefit ratio is poor. Antidepressant treatment should be considered for people with a history of moderate or severe depression, and for those with mild depression that has been present for a long period.

The guidelines further note that antidepressant treatment should be used in combination with psychosocial interventions, should be continued for at least six months to reduce the risk of relapse, and that SSRIs are typically better tolerated than other antidepressants[4]. The American

---

[2] Sinyor M, Schaffer A, Levitt A. The sequenced treatment alternatives to relieve depression (STAR*D) trial: a review. Can J Psychiatry. 2010 Mar;55(3):126-35. doi: 10.1177/070674371005500303.

[3] Depression: The treatment and Management of depression in adults (updated edition)" NICE guidelines [CG90].

[4] Depression: The treatment and Management of depression in adults (updated edition)" NICE guidelines [CG90].

Psychiatric Association treatment guidelines[5] recommend antidepressant medication as an initial treatment choice in people with mild or moderate major depression and should be given to all patients with severe depression. Moreover, treatment should be individually tailored and should consider all available options that may include pharmacotherapy (other symptomatic treatments), psychotherapy, ECT, TMS or tDCS.

7.1.3.2 Psychological Treatments

Many psychological treatments are available for MDD. These include but not limited to, psychoeducation, supportive psychotherapy, various group and individual therapies. Common components of these treatments involve (a) information about the disorder and its treatment; (b) practical support; (c) support for improving family communication; and (d) improving social and problem-solving skills and addressing maladaptive patterns of thinking about themselves and others[6]. The two most common structured psychological interventions are cognitive-behavioral therapy (CBT) and interpersonal therapy (IPT) that target maladaptive thoughts and behaviors using different techniques. A further psychological approach is psychodynamic psychotherapy (PDT) which is based on the principles of psychoanalysis. It aims to help patients increase awareness of their internal thoughts and patterns of behavior which might be contributing to their condition. Psychotherapy is considered a reasonable alternative to medication for mild depression and as an adjunct treatment in more severe depression where it can enhance response and reduce relapse [7,8,9]. However, psychological interventions require the active participation of patients as well as access to specialist therapies which might limit their real-world availability and efficacy.

7.1.3.3 Electroconvulsive Therapy (ECT)

Electroconvulsive therapy (ECT) is also referred to as electroshock or shock treatment mainly in the lay literature. During ECT, an electrical current is passed through the brain to produce an epileptic fit (otherwise known as convulsion or seizure). To minimize any discomfort, ECT is administered under general anesthesia. The current strength for ECT is usually around 800mA, which is 400 times higher than the current used for tDCS. Immediately after treatment, many patients experience headache, muscle aches and temporary amnesia about the period just prior to

---

[5] Practice Guideline for the Treatment of Patients with Major Depressive Disorder. Third Edition, 2010. https://www.psychiatry.org/patients-families/depression/what-is-depression

[6] National Alliance on Mental Illness. https://nami.org/About-Mental-Illness/Treatments/Psychosocial-Treatments

[7] "Depression: The treatment and Management of depression in adults (updated edition)" Clinical guideline. NICE guidelines [CG90]. National Institute for Health and Care Excellence (UK). Published: October 2009, updated 2019. https://www.ncbi.nlm.nih.gov/books/NBK63748/pdf/Bookshelf_NBK63748.pdf.

[8] Practice Guideline for the Treatment of Patients with Major Depressive Disorder. Third Edition, 2010. https://www.psychiatry.org/patients-families/depression/what-is-depression

[9] https://psychiatryonline.org/pb/assets/raw/sitewide/practice_guidelines/guidelines/mdd.pdf

the ECT. Confusion may also occur, mostly in elderly patients, that may last for up to 3 days (ref), and long-term memory dysfunction has been reported in patients. The ECT procedure was first conducted in 1938 and was initially used for a variety of mental health disorders conditions. Currently it is used mainly in patients with treatment-resistant depression. A meta-analysis on the effectiveness of ECT in MDD and bipolar depression conducted in 2012 reported overall remission rates of 51.5% for MDD and 50.9% bipolar depression. As of 2001, it was estimated that about one million people received ECT annually around the world.

7.1.3.4 Transcranial Magnetic Stimulation (TMS)

Transcranial magnetic stimulation (TMS) is a noninvasive procedure approved for the treatment of depression in Europe, Australia, and the US [10]. TMS is typically reserved for patients that have shown minimal or partial response to antidepressants and for patients for whom antidepressant treatment is not suitable. Each treatment session involves placing a purpose-made electromagnetic coil against the scalp in order to induce electric currents in the brain using repetitive magnetic pulses, hence the name repetitive TMS (rTMS). The treatment requires specialist equipment and medical supervision and is therefore not suitable for home use.

7.1.3.5 Cranial Electrotherapy Stimulation (CES)

Cranial electrotherapy stimulation (CES) (also referred to as cranial electrostimulation, electrosleep therapy, and electronarcosis) is a non-pharmacological, non-invasive treatment in which low-intensity electrical current is applied to the scalp. The main difference from tDCS is that CES uses alternating current (AC) rather than direct current (DC) electricity. There is currently a wide variety of CES devices. The Fisher Wallace Stimulator is a CES device which has FDA-approval for the treatment of anxiety, depression, and insomnia, and sold in the EU and Canada without a prescription, directly to consumers to be used at home.

7.1.3.6 Transcranial Direct Current Stimulation (tDCS)

Flow is based on transcranial direct current stimulation (tDCS), a non-invasive brain stimulation technique where a weak direct current is applied on the scalp through electrodes. The current modulates the underlying neural activity. In principle, tDCS electrodes can be placed anywhere on the scalp. Changes in the tDCS electrode placement, also called montage, change the distribution of the induced electrical fields in the brain. Different montages are used depending on the indication. Empirical evidence indicates that a montage that targets the prefrontal cortex can reduce depression symptoms (see section 3.1.3 of the IDE (Attachment 001) for clinical data).

[10] Carpenter LL, Janicak PG, Aaronson ST, et al. Transcranial magnetic stimulation (TMS) for major depression: a multisite, naturalistic, observational study of acute treatment outcomes in clinical practice. Depress Anxiety. 2012 Jul;29(7):587-96. doi: 10.1002/da.21969.

7.2 Investigational Device

7.2.1 Flow FL-100

Neuromodulation is a technique that alters neural activity through the targeted delivery of chemical or electrical stimulus. In particular, brain neuromodulation may be achieved through the direct application of stimulation via implanted deep brain stimulation (DBS) or non-invasively by TMS, CES or tDCS. Direct and non-invasive therapies have been tested for neurologic disorders such as Parkinson's disease, schizophrenia, chronic pain, and MDD, with MDD being the most studied disorder (Brunoni et al. 2010).

TMS to the dorsolateral prefrontal cortex (DLPFC) is shown in the literature to be safe and effective in treating MDD and as a result has received FDA clearance for MDD treatment (Cole et. al., 2019). TMS has proven to be commercially viable, however the treatment has some limitations. The limitations of TMS include the expensive capital equipment required for the delivery of treatment, which can only be delivered with specialist training. Patients being treated with TMS are required to attend multiple clinic visits, sometimes daily or multiple times daily, for weeks at a time, depending on their prescription.

Building from the understanding of the mechanisms, effects and targeting from TMS therapy, and removing some of the logistical hurdles that prevent access to TMS therapy, it is the intent of this study to investigate whether the area of the brain that is being modulated by TMS treatment can be similarly modulated by a home-use tDCS device, thereby offering a more affordable, accessible option for patients with MDD.

Flow Neuroscience has several years of experience developing and marketing tDCS devices for depression treatment. Its device, the Flow FL-100, is a CE-marked class IIa medical device for the treatment of Major Depressive Disorder (MDD). Flow FL-100 has been on the European market since mid-2019 and has been used by over 3000 patients. The product is currently available over the counter in the EU. Market approval has also been gained in Brazil.

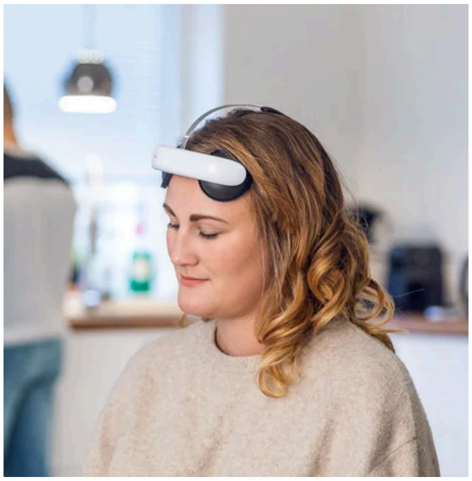

Figure 1. The Flow FL-100 Headset

The purpose of this trial is to investigate the use of the Flow FL-100 device to treat MDD at-home in a double-blind RCT.

7.2.2 The Flow app

The Flow app is used to control the Bluetooth connected Flow tDCS headset and acquire data about its use. The app is connected to a server that stores all data in a secure PostgreSQL database.

For the trial, a dedicated installation of the server and app will be used to isolate all trial data from data collected from retail Flow users. Trial participants will be given a link to the dedicated trial app (Google Play or Apple App store) before starting the trial. In the trial app, participants register with their email. No other identifying information is collected in the app. Only the Flow research team and Curebase will be able to log in to the Flow trial system and database and view the participant emails.

During the trial, the user will answer the nine questions of the MADRS-s form in the app. Answering the form is mandatory to continue with the stimulation. The app also continuously sends headset usage data to the server.

The trial personnel (at Curebase and at the sites in the UK and US) will have access to an administrative web interface where it can follow how much and when users have stimulated. This way they can monitor the usage of each patient and contact them if they start to miss sessions. This retention management is in addition to the normal Curebase retention management.

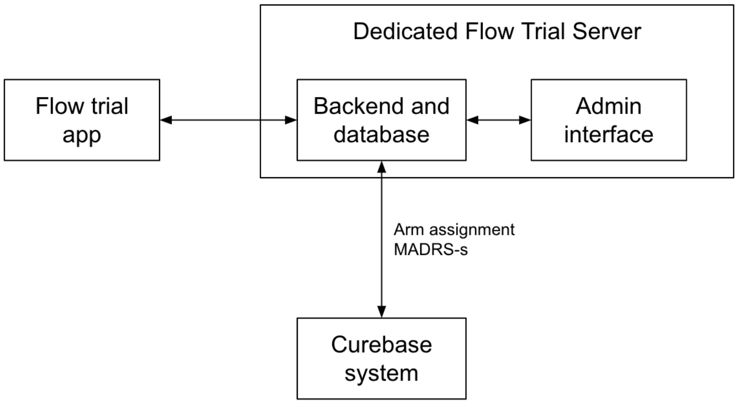

Figure 2. The Flow system and its components. A dedicated app and server will be used. Trial personnel will have access to an admin interface where they can view the activity of participants, unblind them, and move them to the open-label phase.

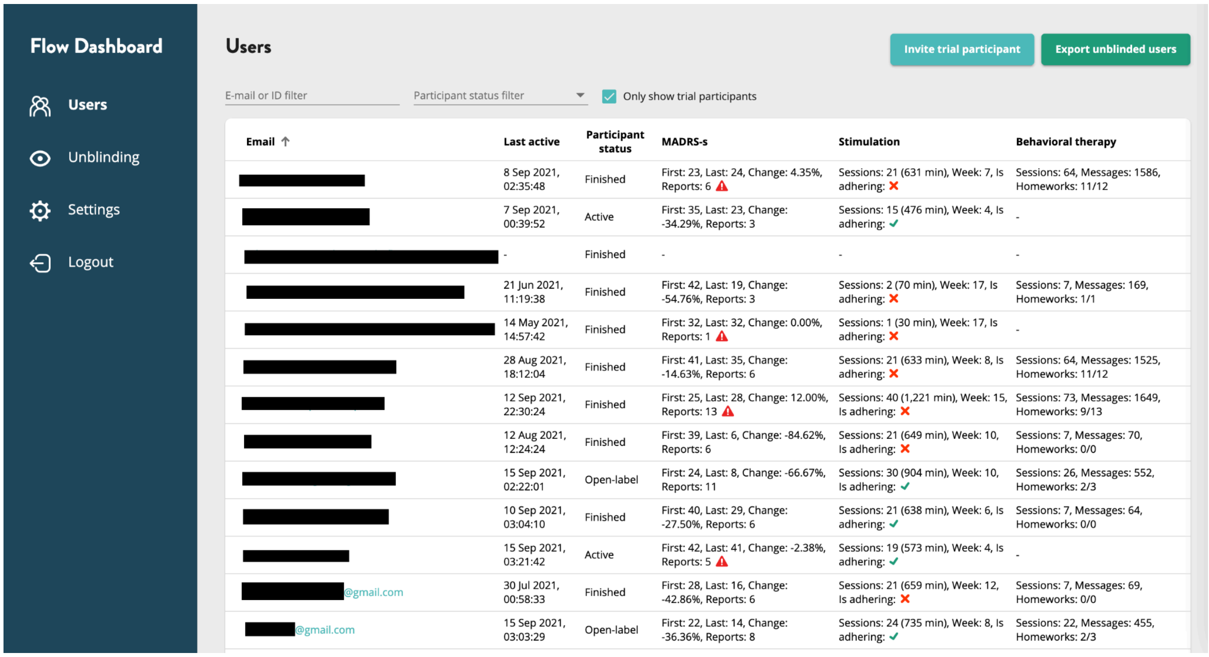

Figure 3 Flow trial admin interface - List of trial participants. Participants are identified through their email.

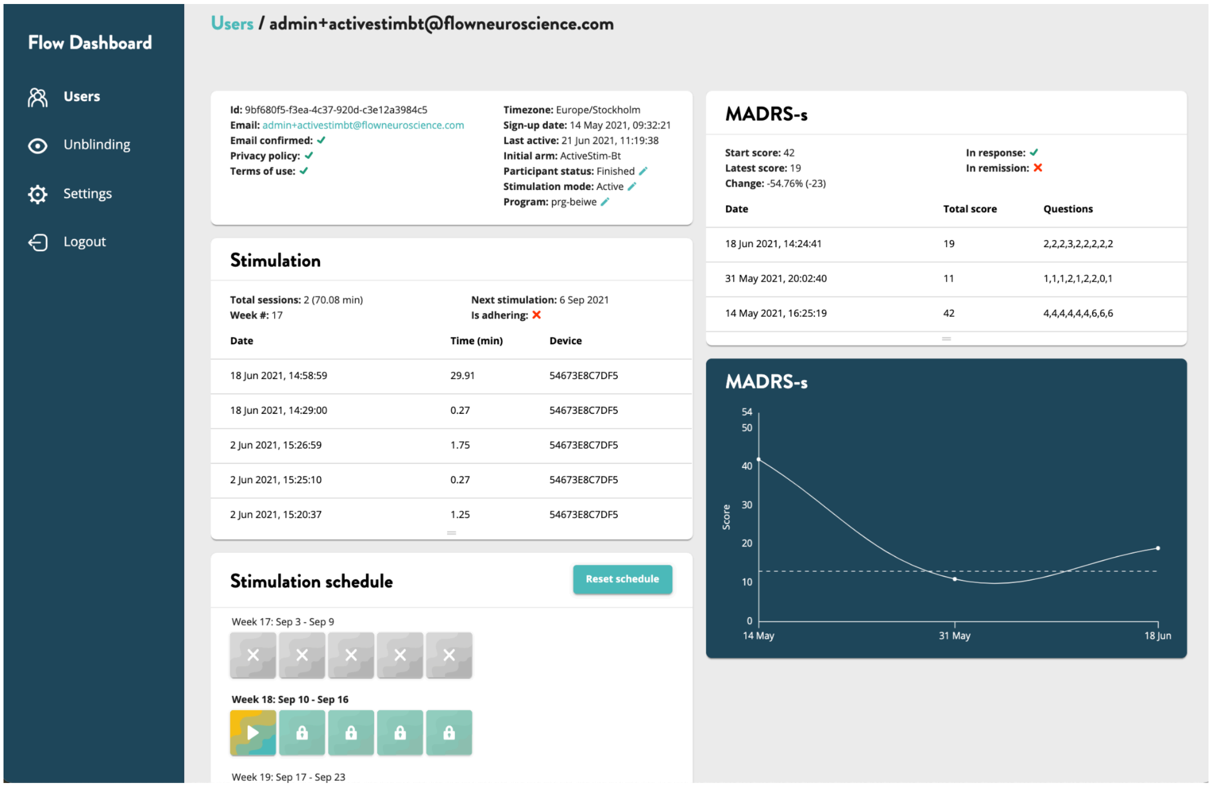

Figure 4 Flow trial admin interface - View of an individual unblinded participant's data. Their MADRS-s scores and stimulation sessions are displayed. After unbinding the user's stimulation mode can be edited.

7.3 Device Traceability

The US warehouse will maintain a Device Accountability Log to document the date of receipt, serial number, date of use, and final disposition of each device. The log will be available for review during monitoring visits. The Flow system also allows the trial personnel to see exactly which device has been used by which participant, and when.

Tracking of the investigational product used in this study will be consistent with 21 CFR Part 821 and ISO 14155:2020, and in accordance with local regulations. Devices will be stored in a secure location at the warehouses assigned for the trial (one in the US and one in Sweden).

At the end of the trial, all devices used in the US will be remotely disabled. Furthermore, a mechanism is in place to ensure that US trial devices cannot be used in the customer-facing Flow app. UK participants will be allowed to keep their devices.

7.4 Supply and Accountability of Investigational Devices

The full name and address are needed for shipping the devices to the participants' homes. There will be one warehouse in Sweden and one in the US. The warehouse in Sweden is the same as used for normal Flow customers, and the warehouse in the US will be dedicated to the trial. To ensure that only limited personnel at Flow have access to shipping information, special accounts in the shipping partners' software systems will be set up. This will isolate information from the normal operations of Flow. Only the CTO from Flow Neuroscience will be able to access the shipping information of participants.

The sponsor is:
Flow Neuroscience AB

7.5 Device Use Experience and Training

The study Sponsor will ensure appropriate training for each investigator prior to initiation of the study at each investigational site. Investigators will be trained on the Flow FL-100 device by the Sponsor, and/or qualified representatives of the Flow FL-100 device prior to their participant's first use. This training will address topics such as the indications and contraindications for the use of the device, device handling, instructions for use and device instrumentation.

In addition, the Sponsor will provide training on the protocol, participant selection criteria, AE management, and follow-up.

8 Benefit-Risk Analysis

8.1 Risks

The study device will deliver a maximum of 2 mA tDCS for 30 minutes at a time. Published data has shown conventional sessions to be ≤ 40 minutes at ≤ 4mA, and the tolerability and dropout rates between sham and active stimulation groups are equal in the literature.

As of August 2021, the most common side effects reported in the literature and from post-market data on Flow FL-100 are:

Table 4. Common side effects from Flow FL-100

Side Effect	% of users
Skin irritation, redness	0.80%
Strong tingling, stinging, or itching	0.14%

Headache	1.1%
Increased tinnitus	0.45%

Most people also experience skin redness at the stimulation site which passes within 30 minutes after the stimulation. Small skin burns are also known from the literature due to the use of tap water instead of physiological saline to wet the stimulation electrodes[11]. Therefore, Flow is delivered with disposable pre-moistened sponges so that the users do not need to handle saline themselves.

The tDCS studies in participants with MDD have reported mostly consistent adverse events with large studies on healthy participants[12]. In addition to this, there have been four studies that have published the appearance of treatment-emergent mania/hypomania (TEM) in eleven cases total[13]. Most cases of TEM have been where tDCS has been combined with antidepressant drugs, which are known to increase the risk of a manic switch[14]. As such, a causal relationship has not been determined. The observed cases have also been mild and in general not required any intervention (correspondence with authors). In the internal data from over 3,000 Flow FL-100 users, no instance of TEM has been discovered or reported. In accordance with the recommendation from Antal et al. (2017), participants with a history of bipolar disorder will be excluded from this study due to their increased risk of manic switch. As an additional safety measure, the YMRS will be administered at each follow-up visit.

8.2 Risk Mitigation

An evaluation of the potential risks associated with use of the Flow FL-100 device has been performed to minimize the risks associated with the device. The following are some of the ways in which risks have been or will be minimized:

8.2.1 Monitors

All Flow devices are controlled by the app and the app is connected to the Flow server over the internet. It is possible to disable devices remotely; this feature ensures patient safety in case of malfunction. This feature is not available for other tDCS devices. The frequency of use of each

[11] Palm U, Keeser D, Schiller C, et al. Skin lesions after treatment with transcranial direct current stimulation. Brain Stimul 2008;1:386e7. [7] Frank E, Wilfurth S, Landgrebe M, et al. Anodal skin lesions after treatment with transcranial direct current stimulation. Brain Stimul 2010;3:58e9.

[12] Antal A, Alekseichuk I, Bikson M, Brockmöller J, Brunoni AR, Chen R, Cohen LG, Dowthwaite G, Ellrich J, Flöel A, Fregni F, George MS, Hamilton R, Haueisen J, Herrmann CS, Hummel FC, Lefaucheur JP, Liebetanz D, Loo CK, McCaig CD, Miniussi C, Miranda PC, Moliadze V, Nitsche MA, Nowak R, Padberg F, Pascual-Leone A, Poppendieck W, Priori A, Rossi S, Rossini PM, Rothwell J, Rueger MA, Ruffini G, Schellhorn K, Siebner HR, Ugawa Y, Wexler A, Ziemann U, Hallett M, Paulus W. Low intensity transcranial electric stimulation: Safety, ethical, legal regulatory and application guidelines. Clin Neurophysiol. 2017 Sep;128(9):1774-1809. doi: 10.1016/j.clinph.2017.06.001. Epub 2017 Jun 19. PMID: 28709880; PMCID: PMC5985830.

[13] André R. Brunoni, Adriano H. Moffa, Bernardo Sampaio-Júnior, Verònica Gálvez, & Colleen K. Loo (2017). Treatment-emergent mania/hypomania during antidepressant treatment with transcranial direct current stimulation (tDCS): A systematic review and meta-analysis. *Brain Stimulation, 10(2), 260-262.*

[14] Patel R, Reiss P, Shetty H, Broadbent M, Stewart R, McGuire P, Taylor M. Do antidepressants increase the risk of mania and bipolar disorder in people with depression? A retrospective electronic case register cohort study. BMJ Open. 2015 Dec 14;5(12):e008341. doi: 10.1136/bmjopen-2015-008341. PMID: 26667012; PMCID: PMC4679886.

individual Flow device is remotely monitored and therefore it is possible to assess adherence to treatment. We expect the Flow device to enhance patient clinical care in general, given the ability for physicians to have remote access to real-time patient data.

8.2.2 Stimulation Parameter Space

Stimulation will always have a maximum output of 2 mA.

8.2.3 Usage Limits

Since Flow is controlled by the accompanying app it limits how often the device can be used and therefore this risk is mitigated. The treatment protocol is however the same for both devices with up to 5 sessions per week the first 3 weeks and then 3 sessions per week. With a maximum of one 30-minute session per day.

8.3 Anticipated Benefits

Participants may experience a clinically meaningful improvement in MDD as has been shown in previous placebo controlled clinical studies with similar therapy. Based on the self-reporting Flow FL-100 users complete every week, over 80% report having some improvement in their depression and around 30% reach remission in 6 weeks. It is also possible that participants will not experience a clinical benefit from participation, although there may be a beneficial addition to the body of knowledge for this therapy. There may be benefits that are unforeseen at this time. Flow also gives participants access to a type of treatment that is in general only available at clinics.

The remission rate of 30% is consistent with what has been found in the literature on tDCS for depression treatment using other devices.

8.4 Risk-to-Benefit Ratio

All applicable risks have been addressed through appropriate testing and clinical monitoring. Any residual risks are acceptable when weighed against the potential benefits to the participant. The device is appropriate for the intended use and is designed to protect the health and safety of the participant, user, and environment.

Therefore, the risk/benefit profile of the Flow FL-100 in the intended use justifies the conduct of the proposed clinical investigation.

9 Study Objectives

9.1 Background

To conduct a double-blind, placebo-controlled, randomized, superiority, remote trial for individuals with major depressive disorder.

9.2 Primary Objective

To show that active stimulation with the Flow FL-100 device is superior to sham stimulation for the treatment of major depressive disorder when used at-home.

10 Study Design

The study will be lead by Professor of Affective Neuroscience Cynthia Fu, PhD, MD, University East London (UK site) and Associate Professor of Psychiatry Sudhakar Selvaraj, PhD, MD, University of Texas Health Sciences center at Houston (UTHealth) (US site), both with extensive experience in managing clinical trials and treating MDD. The other co-investigators are Dr. Jair Soares, PhD, MD, Chairman and Professor of Psychiatry at UTHealth, Houston and Dr. Marsal Sanches, PhD, MD, Associate Professor of Psychiatry at UTHealth, Houston, both have a long history of leading and managing clinical trials and treating MDD and Psychiatric disorders. The other study site investigators are well trained researchers with graduate or postgraduate degrees with training and experience in clinical studies. The study PIs and Co-Is will provide day-to-day supervision to the study investigators and responsible for the overall conduct of the study.

10.1 Study Duration

The study is expected to take approximately 10 months from first participant enrolled to the last open-label visit.

- 5 months enrollment period (estimation)
- 5 months treatment and open-label

The follow-up period during the clinical investigation shall permit the demonstration of performance over a period, sufficient to represent a realistic test of the performance of the investigational device and allow any risks associated with adverse device effects over that period to be identified and assessed.

10.2 Treatment/Control Groups

For each participant, the trial will be conducted in 2 phases: blinded and open label.

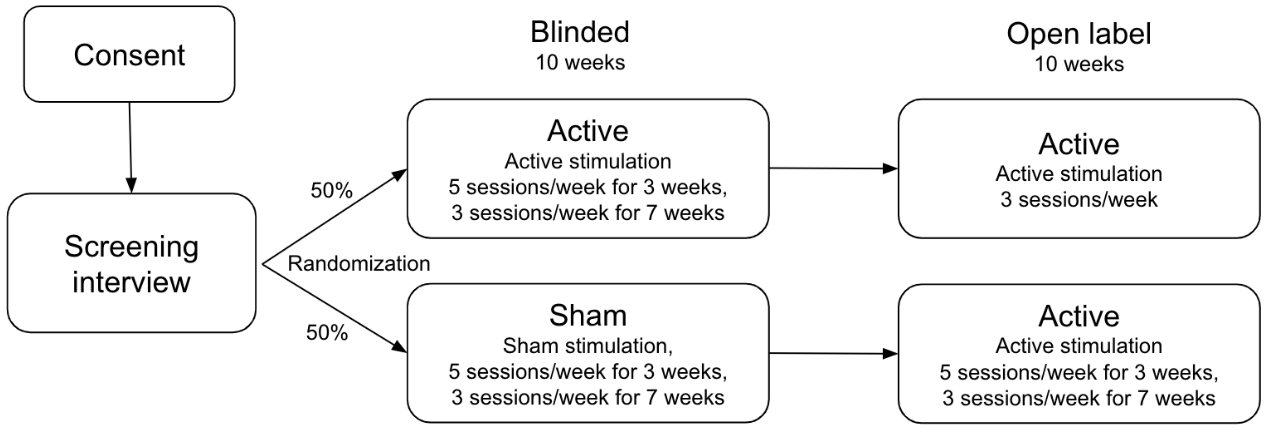

Figure 5 Participant Activity Flowchart

10.3 Blinded phase

During the treatment phase, the participants will be randomized into one of the two arms: active and sham. The participants will be divided equally between the arms and will not be informed of their assignment. The participants will be informed during the consent process that some of them will have sham stimulation during their initial 10-week participation, depending on their randomization assignment.

The sham group receives sham stimulation following how sham tDCS is normally implemented in clinical trials. At the beginning of each 30-minute stimulation session, the current is ramped up to 1mA over 30 seconds. However, when it has reached its maximum, it is again slowly ramped down to 0 mA over 15 seconds. By the end of the session, the current is then again ramped up to 1 mA over 30 seconds and then ramped down to 0 mA over 15 seconds.

The 1 mA current, compared to 2 mA for active stimulation, allows the user to feel the tingling of the stimulation but while still minimizing the amount of current they are exposed to.

When in sham, the app user interface behaves exactly as if the stimulation was active. This way the participants will be blind to the mode of stimulation. If for some reason the stimulation is paused during stimulation the same ramp up and down procedure is done again when the stimulation is resumed.

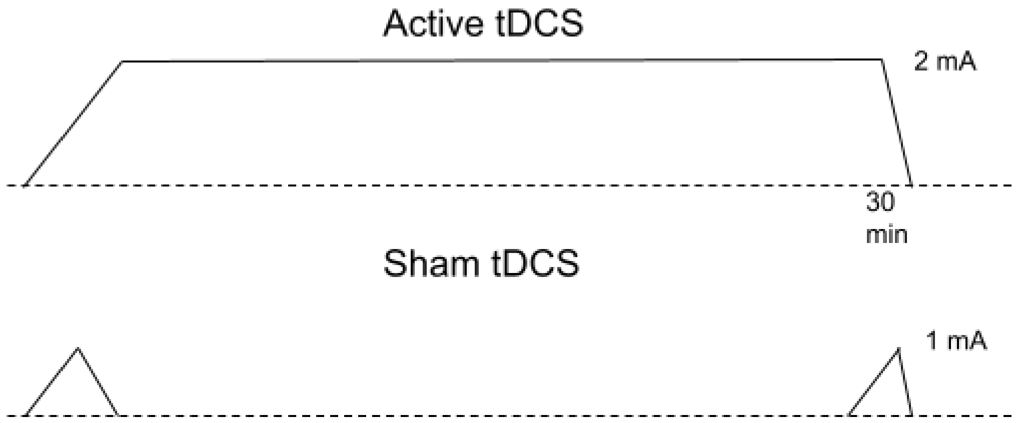

Figure 6. Active vs sham stimulation current envelope.

After the trial period, all user's devices will start working, and users who received sham stimulation can choose to restart the treatment with active stimulation.

10.4 Open label phase

After the completion of the blinded treatment phase all participants will be offered to continue the treatment. Members of the sham group will be offered to restart the treatment with active

stimulation, while members of the active group will be offered to continue maintenance treatment for 10 more weeks at 3 sessions per week.

10.5 Primary Endpoints

The primary effectiveness endpoint is the adjusted mean group difference in the HDRS-17 scores at 10 weeks compared to baseline for subjects in the Active device and the Sham device groups.

10.6 Secondary Endpoints

- Arm difference in HDRS-17 response and remission rate at week 10.
- Arm difference in MADRS average score change, and response and remission rate at week 10.
- Arm difference in MADRS-s average score change, and response and remission rate at week 10.
- Quality of life improvement as measured by EQ-5D-3L in the active compared to sham arm at week 10.

10.7 Exploratory Endpoints

- Young Mania Rating Scale (YMRS) at 10 weeks.
- tDCS Adverse Events Questionnaire (AEQ) at 10 and 20 weeks
- Hamilton Anxiety Rating Scale (HAM-A) change at week 10.
- HDRS-17, MADRS, MADRS-s mean decrease, and remission and response rate at 20 weeks (after open-label) compared to open-label start and baseline.
- Correlation between MADRS and MADRS-s.
- Correlation between adherence to stimulation and HDRS-17/MADRS decrease in the active group at 10 weeks.
- Neuropsychological measure: verbal learning (Rey Auditory Verbal Learning Test (RAVLT) and information processing speed (Symbol-Digit Modalities Test (SDMT)
- Treatment acceptability questionnaire (TAQ) as developed by UEL. There is one form at baseline and another at week 10 and 20.
- The within-patient clinically meaningful improvement as defined as at least -3 points on the HDRS-17 scale. The percentage of subjects for each arm that reaches -3 points or more improvement and also the Cumulative Distribution Function Curves of Change in HDRS-17 Score from Baseline to Primary end-point by arm.

11 Selection of Study Population

11.1 Participant Recruitment

Participants in this study will be recruited via the Flow Neuroscience website, email lists, and online marketing. Potential participants will be directed to a pres-creening form hosted by Curebase, they

will then be required to sign up/register with the Curebase platform to complete a pre-screening survey. This information will primarily be used to screen potential participants for eligibility. Users will be given general information about the trial, and that is it will be blinded.

Participants who seem to meet preliminary eligibility will have a virtual pre-screening visit with a Curebase coordinator prior to being sent to a site investigator for informed consent and further screening procedures. Participants must meet all the following inclusion criteria and none of the exclusion criteria. The investigator maintains exclusive responsibility for the inclusion and exclusion of any potential study participant.

11.2 Inclusion Criteria

To be eligible to participate in this study, participants must meet all the following criteria:

1. Be ≥18 years.
2. Have a diagnosis of Unipolar MDD with a current depressive episode as defined by the diagnostic criteria in the Diagnostic and statistical manual of mental disorders – 5th edition (DSM-V
3. Have a Hamilton Depression Rating Score (HDRS-17) of ≥ 16.
4. For 6 weeks prior to enrollment, are either:
    a. not taking antidepressant medication or:
    b. are taking a stable antidepressant regimen with a stable medication source and agree to continue the same regimen throughout study participation
5. If in psychotherapy, have maintained stable psychotherapy for at least 6 weeks prior to enrollment.
6. Have access to a stable internet connection through which the treatment will be received.
7. Have access to a smartphone or other device running Android 5.0+ or iPhone Operating System (iOS) 12+ (e.g., reasonably new iPhone/iPad or Android phone), used to using the device in their everyday life, and can capably use the study application on the device, as determined by the investigator.
8. Are currently living in England/Wales (UK) or Texas (US).
9. Subject is currently under the care of a psychiatrist or a primary care physician, agrees to be evaluated at regular intervals by a psychiatrist or primary care physician for the duration of study participation, and agrees to promptly inform the study staff of any change of psychiatric or mental health providers during study participation.
10. Subject agrees to allow any and all forms of communication between the investigators/study staff and any healthcare provider who currently provides and/or has provided service to the patient/subject within at least two years of study enrollment.
11. Subject agrees to provide the name and verifiable contact information (email and mailing addresses, mobile and land-line phone numbers, as applicable) of at least two persons ≥ age 18 who reside within a 60-minute drive of the patient's residence and whom the research staff is at liberty to contact, as they deem necessary, for the duration of study participation.

12. Be able to give voluntary, written informed consent to participate and have signed an Informed Consent Form specific to this study.
13. Be willing and able to comply with all study procedures.
14. Subject agrees to meet all of the inclusion criteria throughout their participation in the study. Otherwise, the subject will be discontinued from the study.
15. Subject agrees to a Safety/Suicide Risk Management Protocol, which is intended to reduce the reduce the risk of suicide during study participation.

11.3 Exclusion Criteria

Participants who meet any of the following criteria will be excluded from participating in this study:

1. Are in a current state of mania, as determined by the YMRS or psychosis, as determined by the MINI.
2. Are diagnosed with vitamin or hormonal deficiencies that may mimic mood disorders, as determined by the investigator.
3. Are currently receiving any other interventional therapy for MDD other than a stable regimen of antidepressants or psychotherapy as defined in the inclusion criteria.
4. Considered to have treatment resistant depression as defined by inadequate clinical response to 2 or more trials of antidepressants at an adequate dose and duration.
5. Have a history of electroconvulsive therapy (ECT), transcranial magnetic stimulation (TMS), cranial electrotherapy stimulation (CES), transcranial direct current stimulation (tDCS), deep brain stimulation (DBS), or other brain stimulation.
6. Patient answers Yes to Questions 4, 5 or 6 on the Columbia Suicide Severity Rating Scale (C-SSRS) Triage and Risk Identification Screener.
7. Any previous hospitilization for suicidal behavior.
8. Have chronic severe insomnia (< 4 hours of sleep each night), or depression secondary to chronic insomnia or sleep apnea
9. Have any structural lesion (e.g., any structural neurological condition, or more subcortical lesions than would be expected for age or have had a stroke that affects stimulated area or connected areas) or any other clinically significant abnormality that might affect safety, study participation, or confound interpretation of study results, as determined by the investigator.
10. Have any implant in the brain (e.g., DBS) or neurocranium, or any other active implantable medical device.
11. Have any neurocranial defect.
12. Have a history of epilepsy or seizures (including history of withdrawal / provoked seizures).
13. Have shrapnel or any ferromagnetic material in the head.
14. Have any disorder that would impair the ability to complete the study questionnaires.
15. Have been diagnosed with autism spectrum disorder.
16. Are actively abusing substances (<1 week prior to enrollment).
17. Have a cognitive impairment (including dementia).

18. Have a history of mania or psychosis.
19. Are currently using any medications that affect cortical excitability (e.g., benzodiazepines, epileptics, etc.).
20. Are currently experiencing symptoms of withdrawal from alcohol or benzodiazepines.
21. Have been diagnosed with Parkinsonism or other movement disorder as determined by the investigator to interfere with treatment.
22. Have ever taken esketamine / ketamine for treatment of depression.
23. Have ever been admitted to hospital for depression.
24. Have ever been diagnosed with obsessive-compulsive disorder (OCD) or bipolar type 1 or 2 disorder.
25. Is diagnosed with an active primary anxiety disorder, or PTSD, agoraphobia, anorexia or bulimia, panic or personality disorder with active symptoms.
26. Have a history of psychosurgery for depression.
27. Have any history of myocardial infarction, coronary artery bypass graft (CABG), coronary heart failure (CHF), or history of other cardiac issues.
28. Are currently experiencing or have a history of intractable migraines.
29. Are a chronic tobacco smoker, as defined by smoking by smoking >100 cigarettes (including hand-rolled cigarettes, cigars, cigarillos, etc.) in their life-time and have smoked every day for the last 7 days.
30. If female and of child-bearing potential, currently pregnant or breastfeeding or planning to become pregnant or breastfeed any time during the study.
31. Are currently a prisoner.
32. Are participating concurrently in another clinical investigation or have participated in a clinical investigation within the last 90 days or intend to Participate in another clinical investigation during the study, and where the participation in the other investigation might interfere with the results of this trial as deemed by the PI.
33. Have any medical condition or other circumstances, in the judgment of the investigator, that might interfere with the ability to complete follow-up visits and the self-reported MADRS-s in the app.
34. Have any condition which, in the judgment of the Investigator, would preclude adequate evaluation of the device's safety and performance.
35. A Subject who meets any of the exclusion criteria during study participation will be discontinued from the study

11.4 Study Enrollment Procedures

All participants with MDD will be considered for screening into the study. Participants expressing an interest in participation will proceed with the consent process as per section 13.1 Informed Consent. A participant is not considered officially enrolled in the study until after signing the study consent form.

11.4.1 Participant Enrollment

Participants who consent to participate shall be assigned a unique Participant Identification Number to de-identify their information. This Participant Identification Number will be captured in the Curebase platform and used to identify them on all source documents and eCRFs thereafter.

A record of all screened participants will be maintained in the Curebase platform. The date of screening, results of screening (included or not) and, if not eligible, the primary reason for excluding the participant, enrollment status (enrolled or not) and, if not enrolled, the primary reason will be recorded.

Please note, for purposes of the study a participant is considered enrolled at informed consent.

NOTE: If a participant does not meet the eligibility criteria during their initial screening, the participant cannot be re-evaluated for entry into the study later.

11.5 Screen Failures

A screen failure participant is a consented participant that did not receive the Flow FL-100 treatment due to withdrawing consent, being withdrawn from the study, or not meeting eligibility requirements for treatment.

If a participant signs the ICF but is found ineligible for inclusion in the study prior to receiving the Flow FL-100, the participant will not be advanced any further in this clinical investigation. The participant's signed ICF and completed inclusion/exclusion criteria will be retained by the Investigator, and the participant will be notified.

Screening data from the Screening Visit including the reason for exclusion, will be collected but may not be complete in cases where the participant is determined to be a screen failure early in the screening process. The participant's exclusion from the study, and reason for ineligibility, will be documented in the Screening & Enrollment Log.

11.6 Participant Treatment Assignment

During the technical onboarding session at Week 0, the participant will receive the link to the app and a code to access the app. The participant will download the app and sign up in the app with their email, password, and app access code. When the participant creates their account in the trial app they will automatically be randomized to the sham or active treatment arm (with 50% probability). Block randomization is used with randomly permuted block sizes of 4 and 6. This is done by the trial server and stored in its dedicated database. No one who is working with the trial will have access to the raw data until it has ended.

12 Study Procedures

12.1 Week -3 to -1: Informed Consent (ICF)

After passing the screener survey, prospective participants will be directed to review and sign an electronic HIPAA, and an IRB/EC-approved consent (eConsent) form that are hosted in the Curebase system. The eConsent form will explain the nature and scope of the study, the procedures to be performed as part of the study, the potential risks, and benefits of participation, expected duration of participation, possible treatment alternatives, and an overview of his/her rights as a study participant in lay terms. Participants will have the opportunity to discuss the study with their family or surrogates or think about it before agreeing to participate. Participants will be informed that participation is voluntary and will be assured that they may withdraw from the study at any time and for any reason, without repercussion.

After viewing the eConsent materials, participants will have an opportunity to email or call the research team with any questions they may have and will be given as much time as needed to receive answers before making a final decision regarding study participation. Participants will receive a telephone number, email, and will have access to contact support staff as needed.

When the individual is ready to confirm their willingness to participate, an electronic signature will be collected to complete the eConsent formalities. The participant will receive a copy of the fully signed HIPAA, and eConsent forms via email to retain for their records, and a digital copy will be retained within the Curebase system as part of the study records.

Upon successful documentation of informed consent, a screening video call will be performed and will serve as a baseline evaluation to confirm patient eligibility.

- Clinician Reported Outcome (ClinRO)
    - Hamilton Depression Rating Score (HDRS-17)
    - Montgomery–Åsberg Depression Rating Scale (MADRS)
    - Mini International Neuropsychiatric Interview (MINI)
    - Hamilton Anxiety Rating Scale (HAM-A)
    - Young Mania Rating Scale (YMRS)
    - Rey Auditory Verbal Learning Test (RAVLT)
- Patient Reported Outcome (PRO) Questionnaires
    - EQ-5D-3L
    - Symbol-Digit Modalities Test (SDMT)
    - Treatment Acceptability Questionnaire (TAQ)
    - tDCS Adverse Events Questionnaire (AEQ)

    - Columbia Suicide Severity Rating Scale (C-SSRS)
- Review of concomitant medications
- Final review of inclusion/exclusion criteria
- Intervention kit will be shipped to the participant

12.2 Week 0

To allow the Investigator's assessment of the participant's progress, the participant will complete a video call with study staff and undergo the following:

- Review of concomitant medications
- Record any new or updated adverse events and device deficiencies, if applicable
- SDMT baseline
- Technical onboarding & device training session
- Randomization

12.3 Weeks 1, 4 & 7

To allow the Investigator's assessment of the participant's progress, the participant will complete a video call with study staff and undergo the following:

- ClinROs
    - HDRS-17
    - MADRS
    - YMRS
    - C-SSRS
- Review of concomitant medications
- Record any new or updated adverse events and device deficiencies, if applicable

12.4 Week 10

To allow the Investigator's assessment of the participant's progress, the participant will complete a video call with study staff and undergo the following:

- ClinROs/PROs
    - HDRS-17
    - MADRS
    - HAM-A
    - YMRS

  - EQ-5D-3L
  - RAVLT
  - SDMT
  - TAQ
  - AEQ
  - C-SSRS
- Review of concomitant medications
- Record any new or updated adverse events and device deficiencies, if applicable

For participants in the Sham Arm:

- Participants in the Sham arm will be informed of their assignment and will be offered to start active, Open-Label stimulation for 10 weeks, with 5 sessions the first 3 weeks and then up to 3 sessions per week.

For participants in the Active stimulation Arm:

- Participants in the Active arm will be offered to continue maintenance treatment for 10 more weeks at 3 sessions per week.

12.5 Week 20 Follow-Up Visit - *Open-Label Only*

To allow the Investigator's assessment of the participant's progress, the participant will complete a video call with study staff and undergo the following:

- ClinROs/PROs
  - HDRS-17
  - MADRS
  - HAM-A
  - YMRS
  - EQ-5D-3L
  - RAVLT
  - SDMT
  - TAQ
  - AEQ
  - C-SSRS
- Review of concomitant medications
- Record any new or updated adverse events and device deficiencies, if applicable.

12.6 Video calls

All trial visits will be conducted remotely using real-time video conferencing.

The site personnel who will conduct remote visits will be trained on how to conduct real-time video conferencing visits (e.g., trained on the use of telemedicine for remote clinical trial visits).

Both the researcher and the trial participant should confirm their respective identities with one another before engaging in a visit by showing a photo ID to the camera.

The date and time of a visit should be documented in the trial records.

12.7 Late and Missed Visits

If a participant fails to return for a scheduled study visit within the visit window defined in the study protocol but completes the visit prior to the beginning of the next visit window, that visit is considered to have been completed late. A protocol deviation should be documented on the appropriate eCRF.

If a participant fails to return for a scheduled study visit within the visit window defined in the study protocol and the next scheduled study visit window opens, that visit is considered to have been missed. A protocol deviation should be documented on the appropriate eCRF.

All attempts should be made to schedule the participant for the study visit as soon as possible so that it is captured late rather than missed completely.

12.8 Unscheduled Visits

Participants may be seen for unscheduled visits as needed. During the unscheduled visit, the Investigator will assess the participant's progress and perform the following:

- ClinROs
  - HDRS-17
  - MADRS
  - YMRS
  - C-SSRS
- Review of concomitant medications
- Record any new or updated adverse events and device deficiencies, if applicable

12.9 Study Completion/Discontinuation

The Primary Endpoints will be evaluated at 10 weeks. Participants will complete study participation at 10 weeks unless consented and enrolled in an additional 10-week open-label portion of the study. Participants enrolled in the open-label phase will complete the trial after 20-weeks.

13 Description of Study Procedures

Demographics

At screening, participant baseline characteristics including, but not limited to, age and gender, will be recorded.

Past Medical History

At screening, each participant's past medical conditions and surgical procedures will be recorded.

Pregnancy Determination

Prior to treatment, female participants of childbearing potential will be asked during the Screening visit whether they are pregnant or breastfeeding or plan to become pregnant or breastfeed during the trial.

Clinician and Patient Reported Outcome Questionnaires (ClinROs/PROs)

The following Clinician or Patient Reported Outcome questionnaires will be completed and used to compare participant outcomes at follow-up time points compared to baseline:

Montgomery–Åsberg Depression Rating Scale (MADRS) - ClinRO

The Montgomery–Åsberg Depression Rating Scale (MADRS) is a ten-item diagnostic questionnaire which psychiatrists use to measure the severity of depressive episodes in patients with mood disorders.

Montgomery–Åsberg Depression Rating Scale (MADRS-s) - PRO

MADRS-s is a self-rating version of MADRS and correlates well with expert ratings. The MADRS-s instrument has nine questions, with an overall score ranging from 0 to 54 points. During the trial, each participant reports their MADRS-s score in the same way as normal customers would. The MADRS-s uses cut-off points detailed below to classify the severity of depressive symptoms:

- 0-12 points: None or very mild depression
- 13-19 points: Mild depression
- 20-34 points: Moderate depression
- ≥ 35 points: Severe depression

Hamilton Depression Rating Score (HDRS-17)

The HDRS (also known as the Ham-D) is the most widely used clinician-administered depression assessment scale. The original version contains 17 items pertaining to symptoms of depression

experienced over the past week. The questionnaire is designed to be used by clinicians to rate the severity of depressive symptoms in adults by probing mood, feelings of guilt, suicide ideation, insomnia, agitation or retardation, anxiety, weight loss, and somatic symptoms.

Mini International Neuropsychiatric Interview (MINI) - ClinRO
The Mini-International Neuropsychiatric Interview (M.I.N.I.) is a short structured diagnostic interview, developed jointly by psychiatrists and clinicians in the United States and Europe, for DSM-IV and ICD-10 psychiatric disorders.

Columbia Suicide Severity Rating Scale (C-SSRS) - ClinRo
The Columbia Suicide Severity Rating Scale, or C-SSRS, is a rating scale to evaluate suicide risk. The scale identifies specific behaviors which may be indicative of an individual's intent to kill oneself.

Hamilton Anxiety Rating Scale (HAM-A) - ClinRo
The HAM-A was one of the first rating scales developed to measure the severity of anxiety symptoms and is still widely used today in both clinical and research settings. The scale consists of 14 items, each defined by a series of symptoms, and measures both psychic anxiety (mental agitation and psychological distress) and somatic anxiety (physical complaints related to anxiety).

Young Mania Rating Scale (YMRS) - ClinRo
The Young Mania Rating Scale (YMRS) is one of the most frequently utilized rating scales to assess manic symptoms. The scale has 11 items and is based on the patient's subjective report of his or her clinical condition over the previous 48 hours.

Rey Auditory Verbal Learning Test (RAVLT) - ClinRo
The Rey Auditory Verbal Learning Test (RAVLT) is a neuropsychological assessment designed to evaluate verbal memory in patients, 16 years of age and older. The RAVLT can be used to evaluate the nature and severity of memory dysfunction and to track changes in memory function over time.

Symbol-Digit Modalities Test (SDMT) - PRO
The Symbol Digit Modalities Test (SDMT) detects cognitive impairment. It is a way for clinicians to screen for organic cerebral dysfunction in both children (eight years and older) and adults.
The SDMT is brief, easy to administer, and has demonstrated sensitivity in detecting not only the presence of brain damage, but also changes in cognitive functioning over time and in response to treatment.

Treatment Acceptability Questionnaire (TAQ) - PRO
TAQ is a questionnaire for probing how acceptable participants of tDCS clinical trial think the treatment is. At baseline it measures participant's expectations about the trial, e.g., treatment outcome, effort needed, ethics, etc., and then follow-up on these expectations at the end of the trial.

tDCS Adverse Events Questionnaire (AEQ) - PRO
AEQ is a specialized questionnaire developed to capture the adverse events during tDCS trials.

EQ-5D-3L - PRO
The EQ-5D-3L is a standardized measure of health-related quality of life.

14 Early Discontinuation: Participants

14.1 Technical Failures

In case the Flow device or the participant's smartphone breaks, and it cannot be replaced within 10 days, and the participant has not already completed 22 sessions, this will be considered a technical failure, and the participant will be withdrawn from the trial.

14.2 Treatment Failures

The following situations will be considered treatment failures and participants will be withdrawn from the trial:

- 30% increase in HDRS-17 score compared to baseline.
- Suicidal attempts / completion
- Device related SAE

14.3 Lost to Follow-Up (LTFU)

Participants who miss the final, 10-week study visit will be regarded as Lost to Follow-Up. As described in the Statistical Analysis Plan, these subjects will be included in the primary analysis through Multiple Imputation. Therefore, this definition of LTF only applies to study operations and not data analysis. Curebase or site staff should perform and document a minimum of three attempts to contact them via phone to complete the final study visit prior to considering the participant LTFU. The Curebase or site staff should document the date and type of attempted communication.

All efforts should be made to have participants complete all follow-up visits.

14.4 Withdrawals

Participants have the right to withdraw from the clinical investigation at any time and for any reason. The Principal Investigator will ask the reason for their withdrawal and will record all information regarding the participant discontinuation.

A Participant may be withdrawn from the clinical investigation for the following reasons:

- Participants may choose to withdraw from the clinical investigation under the terms of the Declaration of Helsinki and their consent documentation without having to give a reason;

- Any unanticipated adverse event which, in the opinion of the Principal Investigator, is related to the treatment and will endanger the well-being of the participant if treatment is continued;
- Development of any intercurrent illness(es), infection or condition(s) that might interfere with the clinical investigation;
- Non-compliance with the clinical investigation procedures deemed by the Principal Investigator to be sufficient to cause discontinuation;
- Any problem deemed by the Investigator to be sufficient to cause discontinuation.

Participants will be withdrawn from the trial if they do not comply with the intervention, namely if they are unable to have a minimum of 60% of the tDCS session (22 (660 minutes) out of 36 sessions at week 10) during the blinded phase, or if they develop serious adverse effects from any part of the study. Recording of the reasons for withdrawal will be made. Their GP will be informed about their withdrawal, and they will have a telephone follow up at 1 month following their withdrawal.

If a participant is withdrawn, their device is disabled to make sure they cannot continue the treatment.

Regardless of the reason for withdrawal, data available for the participant at the time of withdrawal, including the reason for withdrawal, will be entered on the Case Report Forms. The research staff will request to have an optional early termination visit to collect the same data as at the week 10 and week 20 interview. The Principal Investigator will treat all participants discontinued from the investigation due to an unanticipated adverse event, directly related to the investigation, until the reaction resolves. A participant that has been withdrawn from the study after treatment start will not be replaced.

14.5 Documentation of Early Discontinuation and Pregnancy

In every instance where a participant does not complete the study, the Investigator will document the primary reason for discontinuation in the participant's records.

All participants are free to withdraw from participation at any time, for any reason, specified or unspecified, and without prejudice. However, if a participant expresses a desire to withdraw their consent for the study, the site should attempt to obtain written documentation for their study records.

For participants that are discontinued by the Investigator, the Investigator must notify them of their discontinuation from the study in writing.

To ensure the safety of participants who become pregnant during the study, pregnant participants will be withdrawn from the study.

14.6 Use of Data from Early Discontinuation Cases

Study data collected previously for participants who withdraw from the study, are discontinued from the study by the Investigator or LTFU will be included in the data analysis and clinical study report.

14.7 Ongoing Treatment for Early Discontinuation Cases

Participants who withdraw voluntarily or are discontinued by the Investigator will remain eligible for SOC treatment by the Investigator and study staff.

15 Early Discontinuation: Study

15.1 Procedure for Suspension or Early Termination

The Sponsor may suspend or prematurely terminate this clinical investigation either at an individual investigational site or at all sites. A Principal Investigator, IRB/EC, or regulatory authority may suspend or prematurely terminate participation in this clinical investigation at the investigational sites for which they are responsible.

If suspicion of an unacceptable risk to participants arises during the clinical investigation, or when so instructed by the IRB/EC or regulatory authorities, the Sponsor will suspend the clinical investigation while the risk is assessed. If an unacceptable risk is confirmed, the Sponsor will terminate the clinical investigation.

The Sponsor will consider terminating or suspending the participation of a particular investigational site or Investigator if monitoring or auditing identifies serious or repeated deviations on the part of an Investigator.

If suspension or premature termination occurs, the terminating party will justify its decision in writing and promptly inform the other parties with whom they are in direct communication.

If suspension or premature termination occurs:

The Sponsor will remain responsible for providing resources to fulfill the obligations from this protocol and existing agreements for following the participants enrolled in the clinical investigation, and the site's Principal Investigator or authorized designee will promptly inform the enrolled participants at his/her investigational site

If the study is terminated, Flow Neuroscience AB must comply with all applicable government regulations and the protocol-required participant follow-up. If discontinuation of the study should occur, the Principal Investigator must return all clinical investigation materials (including devices) to the Sponsor and provide a written statement to the IRB/EC explaining the reasons for the premature termination. All applicable clinical investigation documents shall be participant to the same retention policies, as detailed in Section 24.3.

15.2 Procedure for Resuming the Clinical Investigation After Temporary Suspension

When the Sponsor concludes an analysis of the reason(s) for the suspension, implements the necessary corrective actions, and decides to lift the temporary suspension, the Sponsor will inform the relevant parties of the rationale and provide them with the relevant data supporting this decision. Concurrence will be obtained from the IRB/ECs and, where appropriate, regulatory authorities before the clinical investigation resumes. If participants have been informed of the suspension, the Principal Investigator or authorized designee will inform them of the reasons for resumption.

15.3 Routine Close-Out

Routine close-out activities will be conducted to ensure that the Principal Investigator's records are complete, all documents needed for the Sponsor's files are retrieved, remaining clinical investigation materials are destroyed, previously identified issues have been resolved, and all parties are notified.

16 Protocol Deviations

Conformance to the protocol is essential to the quality and integrity of the clinical study. Every effort should be made to avoid any deviation from the clinical protocol. A protocol deviation is an event whereby the clinical investigator or site personnel did not conduct the study according to the protocol.

Under emergency circumstances, deviations from the protocol to protect the rights, safety and well-being of human subjects may proceed without prior approval of the sponsor and the IRB/EC. Such deviations shall be documented and reported to the sponsor in the EDC within 5 days of knowledge. The IRB/EC should be notified of the deviation as required by IRB/EC reporting guidance.

17 Concomitant Medications/Therapies

For the purposes of this clinical study, only medications taken or administered for the following will be recorded *(Ensure medications reflected in incl/excl are listed here)*:

- Muscle relaxation;
- Other medications related to or given for the participant's MDD.

18 Adverse Event & Device Deficiency Reporting

Subjects will be carefully monitored during the study for possible adverse events (AEs) from the time they sign the Informed Consent Form to the time of study completion. All medical events and conditions prior to this time point are to be captured as medical history.

Any observed unanticipated AE will be fully investigated by the Investigator and classified in line with the definitions of ISO 14155:2020 below. The frequency and severity of anticipated AEs will instead be captured using the tDCS Adverse Events Questionnaire (AEQ).

All unanticipated adverse events that occur during this study will be recorded on the adverse event eCRFs and must include the following information at minimum:

- Event Description
- Date of Onset
- Date of Resolution
- Severity
- Seriousness
- Relationship to Study Device/Procedure
- Outcome

Adverse events will be reported and classified by the Investigator using the specific signs, symptoms or abnormal laboratory values, or medical diagnosis if no signs, symptoms, or abnormal laboratory values can be identified.

Significant new information and updates should continue to be captured in the subject's records and in the EDC as they become available and the adverse event should be followed until it is resolved or no further improvement is expected, or the subject is lost to follow-up.

Adverse events that meet the definitions of seriousness in Section 19.1 below should also be reported to the Sponsor per the instructions provided in Section 19.2.4 below.

18.1 Definitions

The following definitions are from the ISO 14155:2020 and MDCG 2020-10:

Adverse Event (AE) – An adverse event (AE) is any untoward medical occurrence, unintended disease or injury, or untoward clinical signs (including abnormal laboratory findings.) in subjects, users, or other persons, whether or not related to the investigational device and whether anticipated or unanticipated.

NOTE 1: This definition includes events related to the investigational medical device or the comparator.
NOTE 2: This definition includes events related to the procedures involved.
NOTE 3: For users or other persons, this definition is restricted to events related to the use of investigational medical devices.

Anticipated Adverse Events – Anticipated adverse events are adverse events that have been identified as adverse events related to the investigational device or study procedure. The anticipated events of this clinical investigation are outlined in tDCS Adverse Events Questionnaire (AEQ). The frequency and severity of anticipated AEs will be captured using AEQ.

Anticipated adverse events are:

- Headache
- Neck pain
- Scalp pain
- Tingling
- Itching
- Burning sensation
- Skin redness
- Sleepiness
- Trouble concentrating
- Acute mood change

Adverse Device Effect (ADE) – An adverse device effect (ADE) is any adverse event related to the use of an investigational device. This definition includes:

- Adverse events resulting from insufficient or inadequate instructions for use, deployment, implantation, installation, or operation, or any malfunction of the investigational medical device.
- Any event resulting from use error or from intentional misuse of the investigational medical device.
- "Comparator" if the comparator is a medical device.

Serious Adverse Event (SAE) – A Serious Adverse Event is an AE which:

1. Led to a death,
2. Led to serious deterioration in the health of the subject, users, or other persons that either resulted in:
   a) A life-threatening illness or injury, or
   b) A permanent impairment of a body structure or a body function including chronic diseases, or
   c) In-patient or prolonged hospitalization, or
   d) Medical or surgical intervention to prevent life threatening illness or injury or permanent impairment to a body structure or a body function.
3. Led to fetal distress, fetal death or a congenital abnormality or birth defect including physical or mental impairment (not anticipated in this study as pregnant women are excluded from the study).

NOTE: Planned hospitalization for any pre-existing condition, or a procedure required by the Clinical Investigation Plan, without a serious deterioration in health, is not considered a serious adverse event.

Serious Adverse Device Effect (SADE) – a Serious Adverse Device Effect is an adverse device effect that has resulted in any of the consequences characteristic of a serious adverse event.

Anticipated Serious Adverse Device Effect (ASADE) – an Anticipated Serious Adverse Effect is an effect which by its nature, incidence, severity, or outcome has been identified in the risk analysis report.

Unanticipated Serious Adverse Device Effect (USADE) – this is a serious adverse device effect which by its nature, incidence, severity, or outcome has not been identified in the current version of the risk analysis report. This is also referred to as an Unanticipated Adverse Device Effect (UADE) in the US. See Section 19.2.4 for details regarding determination and reporting requirements in the US.

Device Deficiency (DD) - A device deficiency is an inadequacy of a medical device with respect to its identity, quality, durability, reliability, safety, or performance. Device deficiencies include malfunctions, use errors, and inadequacy in the information supplied by the manufacturer including labelling.

Malfunction – Failure of an investigational medical device to perform in accordance with its intended purpose when used in accordance with the instructions for use or protocol.

Use Error – User action or lack of user action while using the medical device that leads to a different result than that intended by the manufacturer or expected by the user.

NOTE1: Use error includes the inability of the user to complete a task.
NOTE 2: Use errors can result from a mismatch between the characteristics of the user, user interface, task or use environment.
NOTE 3: Users might be aware or unaware that a use error has occurred.
NOTE 4: An unexpected physiological response of the patient is not by itself considered a use error.
NOTE 5: A malfunction of a medical device that causes an unexpected result is not considered a use error.

18.2 Classification of an AE

18.2.1 Severity

Investigators will assess the severity of the adverse event and classify it according to the following definitions:

- **Mild**: Awareness of a sign or symptom that does not interfere with the patient's usual activity or is transient, resolved without treatment and with no sequelae.
- **Moderate**: Interferes with the patient's usual activity and/or requires symptomatic treatment.
- **Severe**: Symptom(s) causing severe discomfort and significant impact on the patient's usual activity and requires treatment.

These definitions are for descriptive purposes only and are independent of the judgment of whether an event is classified as an AE or an SAE.

18.2.2 Assessment of Relationship to Use of the Investigational Device or Procedure

The relationship between the use of the medical device (including the medical - surgical procedure) or control procedure the occurrence of each unanticipated adverse event shall be assessed and categorized. During causality assessment, clinical judgment shall be used, and the Clinical Protocol shall be consulted, as all the foreseeable adverse events and the potential risks are listed and assessed there. The presence of confounding factors, such as concomitant medication/treatment, the natural history of the underlying disease, other concurrent illness or risk factors shall also be considered.

18.2.3 SAE Relationship Assessment

Per MDCG 2020-10/1 (May 2020), Investigators will assess the potential relationship of the SAE to the use of the investigational device (including the surgical procedure) or control procedure and classify the causality of the event according to the following definitions.

- **Not Related**: Relationship to the device or procedures can be excluded when:
  - the event has no temporal relationship with the use of the investigational device, or the procedures related to application of the investigational device;
  - the serious event does not follow a known response pattern to the medical device (if the response pattern is previously known) and is biologically implausible;
  - the discontinuation of medical device application or the reduction of the level of activation/exposure - when clinically feasible - and reintroduction of its use (or increase of the level of activation/exposure), do not impact on the serious adverse event;
  - the event involves a body-site or an organ that cannot be affected by the device or procedure;
  - the serious event can be attributed to another cause (e.g., an underlying or concurrent illness/ clinical condition, an effect of another device, drug, treatment, or other risk factors);
  - the event does not depend on a false result given by the investigational device used for diagnosis, when applicable;
  - harms to the subject are not clearly due to use error;

  - In order to establish the non-relatedness, not all the criteria listed above might be met at the same time, depending on the type of device/procedures and the serious adverse event.
- **Possible:** The relationship with the use of the investigational device or the relationship with the procedure is weak but cannot be ruled out completely. Alternative causes are also possible (e.g., an underlying or concurrent illness/ clinical condition or/and an effect of another device, drug, or treatment). Cases where relatedness cannot be assessed or no information has been obtained should also be classified as possible.
- **Probable**: The relationship with the use of the investigational device or the relationship with the procedure, seems relevant and/or the event cannot reasonably be explained by another cause.
- **Causal Relationship**: The serious adverse event is associated with the investigational device or with procedures beyond reasonable doubt when:
  - the event is a known side effect of the product category the device belongs to or of similar devices and procedures;
  - the event has a temporal relationship with investigational device use/application or procedures;
  - the event involves a body-site or organ that
    - the investigational device or procedures are applied to;
    - the investigational device or procedures have an effect on;
  - the serious event follows a known response pattern to the medical device (if the response pattern is previously known);
  - the discontinuation of medical device application (or reduction of the level of activation/exposure) and reintroduction of its use (or increase of the level of activation/exposure), impact on the serious event (when clinically feasible);
  - other possible causes (e.g., an underlying or concurrent illness/ clinical condition or/and an effect of another device, drug, or treatment) have been adequately ruled out;
  - harm to the subject is due to error in use;
  - the event depends on a false result given by the investigational device used for diagnosis, when applicable;
  - In order to establish the relatedness, not all the criteria listed above might be met at the same time, depending on the type of device/procedures and the serious event.

Where the Investigator or Sponsor remains uncertain about classifying the event, it should not exclude the relatedness and the Investigator should classify the event as “Possibly Related”.

18.2.4 SAE Reporting

Flow Neuroscience AB is responsible for the classification and reporting of adverse events and ongoing safety evaluation of the clinical investigation in line with ISO 14155:2020 and US/UK regulatory requirements.

Investigator will report "to the Sponsor, without unjustified delay, all serious adverse events and device deficiencies that could have led to a serious adverse device effect; this information shall be promptly followed by detailed written reports" [ISO 14155:2020 § 9.8 b]. Device malfunctions and use errors should also be reported without unjustified delay.

In the event of becoming aware of any SAE (including all device deficiencies), anticipated or unanticipated, the Investigator will report the event to Flow Neuroscience AB and Curebase immediately (at least within 24 hours after first knowledge of the event) by entering SAE information in the EDC. In the event the EDC is unavailable for any reason, notification to Flow Neuroscience AB should be made via email as follows:

E-mail: —

The Investigator should provide additional information on the SAE by updating the information in the EDC as updates become available. Flow Neuroscience AB may also ask for additional clinical reports including redacted source documents to be provided by the Investigator to assist in the assessment of the event. Significant new information and updates should continue to be submitted promptly to and entered in the EDC as they become available, and the Investigator should follow the SAE until it is resolved, or no further improvement is expected.

It is the responsibility of each Investigator to report all SAEs and/or SADEs to the IRB/EC, according to national regulations and IRB/EC requirements. A copy of the IRB/EC report should be forwarded to Flow Neuroscience AB. In countries that require the Sponsor to report safety events rather than investigators, the Sponsor (Flow Neuroscience AB) will report SAEs and/or SADEs to the EC.

Flow Neuroscience AB will ensure that all SAEs and device deficiencies that could have led to a SAE are reported to the Competent Authorities in accordance with European Medical Devices directives and all applicable national regulations.

Flow Neuroscience AB will ensure that all events that meet the vigilance requirements as described in MDCG 2020-10/1 (Safety reporting in clinical investigations of medical devices under the Regulation (EU) 2017/745) are reported to the Competent Authorities in accordance with European Medical Devices directives and all applicable national regulations.

18.2.5 Determination and Reporting of Unanticipated Adverse Device Effects (US Sites)

The Sponsor shall review all reported SAEs to evaluate whether they meet the criteria for an Unanticipated Adverse Device Effect. For adverse events that are determined to be UADEs, the Sponsor will submit an expedited safety report to the FDA's Center for Devices and Radiological Health (CDRH). The expedited safety report will be submitted to the FDA as soon as possible and, in no event, later than ten (10) business days after the Sponsor first receives notice of the UADE. A copy of this safety report will be provided to all participating study Investigators.

If, following receipt and investigation of follow-up information regarding an adverse event that was previously determined not to be a UADE, the Sponsor determines that the event does meet the requirements for expedited reporting, the Sponsor will submit a report as soon as possible, but in no event later than **ten (10) business days** after this is determined.

18.3 Treatment of AEs

The Investigator will treat all participants who experience an adverse event directly related to the device.

18.4 Safety Oversight: Clinical Events Committee (CEC)

The Clinical Events Committee (CEC) will adjudicate all AEs and protocol deviations. SAEs and AEs deemed "definitely," "probably," and "possibly related" or with an "unknown relationship" to the device or procedure, primary endpoint failure, and the following neurological AEs, including neurologic deterioration will be reviewed.

18.5 Safety/Suicide Risk Management Plan

All study participants will be provided a safety crisis action plan for suicide risk at the time of study enrollment. The crisis action plan will include the following information;

- Name, address, and telephone number of the closest hospital emergency department near the study participant's residence
- Contact details of next of kin/friends for support
- Study participant's regular treating physician's name and contact information
- National Suicide Prevention Lifeline 800-273-8255
- Strategies for coping with suicidal ideation/crisis.
- Further, subjects will be notified that study personnel will contact their psychiatrist in the case of worsening depression/suicidality (worsening depression is defined as 30% increase in HDRS-17 score compared to baseline).

All participants will be informed about the warning signs of suicidal ideation and behavior at the start of the study. Throughout the study, subjects will be monitored by study psychiatrists.

If the C-SSRS questions 4, 5, and 6b are positive during the study assessments, the participant will be asked to go to the nearest emergency department or mental health crisis center.

The suicide risk management plan document (attachment "Brown_StanleySafetyPlan") will be completed with patients.

19 Stopping rules

The following events will result in the study stopping:

- If two subjects commit suicide
- If three subjects attempt suicide

- If ≥ 2 serious adverse events (SAEs) occur that are device-related, as determined by investigators, the IRB, the FDA, or the Clinical Events Committee (CEC)

If any of the stopping rules are reached, enrollment will be paused and the FDA will be notified in a supplement to enable review of the information.

20 Clinical Monitoring

20.1 Site Monitoring

The study will be monitored to ensure that it is conducted in conformance with the monitoring plan by an independent CRO to assess continued compliance with the protocol, recognized Good Clinical Practices, FDA's 2013 guidance document "Guidance for Industry Oversight of Clinical Investigations — A Risk-Based Approach to Monitorings, and federal regulations outlined in 21 CFR Part 812

- Subpart A
- Subpart B
- Subpart C -
    - 812.3 Definitions
    - 812.25 Investigational Plan
    - 812.28 Acceptance of data from clinical investigations conducted outside the United States
    - 812.30 FDA action on applications
    - 812.35 Supplemental applications
    - 812.36 Treatment use of an investigational device
    - 812.40 General responsibilities of sponsors
    - 812.43 Selecting investigators and monitors
    - 812.46 Monitoring investigations
    - 812.47 Emergency research under 50.24 of this chapter
    - 812.140 Records
    - 812.150 Reports

In addition, the monitor verifies that study records are adequately maintained, that data are reported in a satisfactory manner with respect to timeliness, adequacy, and accuracy, and that the Investigator continues to have sufficient staff and facilities to conduct the study safely and effectively.

The study may also be subject to a quality assurance audit by the Sponsor or its designees, as well as inspection by appropriate regulatory authorities. It is important that the Investigator and relevant study personnel are available during on-site monitoring visits or audits and that sufficient time is devoted to the process.

The organization responsible for monitoring the study is:

Curebase Inc.
335 S Van Ness Ave
San Francisco, CA 94103-3627
(519)590-8490
www.curebase.com

In addition to ensuring adequate communication between the Investigators and the Sponsor, the CRO's duties include on-site visits and review of study documents and reported data. The CRO study representatives will be provided with appropriate device and protocol training prior to the study and will follow a Monitoring Plan for all study-related monitoring activities.

20.2 Monitoring Activities

Monitoring visits include a pre-study Site Initiation Visit, periodic Interim Monitoring Visits, and a Close-Out Visit at the end of the site's participation in the study.

Each site's Investigator will allocate adequate time for monitoring activities. The Investigator will also ensure that the monitor or other compliance or quality assurance reviewer is given access to the study-related documents and study-related facilities and has adequate space to conduct the monitoring visit.

Monitoring visits will be documented on monitoring visit reports, and will aim to verify that:

- Compliance with the clinical protocol and applicable regulations is being maintained
- Source data is verified and signed-off upon as accurate
- Participant files are accurate and complete
- Participant withdrawal has been documented (if applicable)
- Participant non-compliance has been documented (if applicable)
- The Investigator and site staff are informed and knowledgeable of all relevant document updates concerning the clinical investigation
- Only authorized individuals are performing study-related functions
- The investigational device is being used according to the protocol and instructions for use
- Adequacy of staffing and facilities
- Signed and dated ICFs have been obtained from each participant
- eCRFs and queries are complete
- All adverse events are reported to the Sponsor
- All serious and unanticipated adverse device events are reported to the Sponsor and the IRB/EC

- All other required IRB/EC reports, notifications, applications, submissions, and correspondence are maintained in the Investigator's files and are accurate
- Corrective and preventive actions have been implemented (if applicable)

20.3 Frequency of Visits

To ensure that the study is conducted in accordance with the terms of the clinical protocol, study monitors must visit each investigational site at routine intervals throughout the duration of the study. The exact frequency of visits shall be determined on an individual site basis as detailed in the Clinical Monitoring Plan, and shall depend upon the following factors:

- Rate of participant enrollment
- Experience of the Investigator in conducting clinical studies
- Record of previous site compliance

20.4 Audit and Inspections

Participation as an Investigator in this study implies acceptance of potential inspection by government regulatory authorities. The Investigator must also be prepared to permit study-related audits and inspections by the Sponsor, CRO, IRB/EC and the site's institutional compliance and quality assurance groups. The Investigator will ensure the capability for inspections of applicable study-related facilities, records, and reports.

21 Statistical analysis

21.1 Study Hypothesis

The primary clinical outcome of the study will be to compare the adjusted mean group differencebased on HDRS-17 scores of the two arms at 10 weeks compared to baseline.

The primary efficacy hypothesis is the change in HDRS-17 scores between 10 weeks and baseline between subjects randomized to the study device relative to those subjects randomized to Sham. The formal statistical hypothesis is as follows:

Ho: $d_{flow} – d_{sham} \leq 0$
Ha: $d_{flow} – d_{sham} > 0$

Where d_{flow} and d_{sham} are the adjusted mean group difference in HDRS-17 scores in subjects randomized to Flow and Sham groups, respectively.

21.2 Sample Size Assumptions and Calculations

The sample size calculation is based on data from Brunoni et al (2017). The calculation is based on a two- sample t-test for mean difference with the following assumptions:

- Group difference in means = 3.2
- Standard deviation of Investigational and Sham groups = 7.1 and 7.9, respectively
- One-sided type I error = 0.025
- 80% power

The resulting sample size is 176 subjects. To obtain an increase in statistical power (87.6%), 216 subjects were chosen. Accounting for 20% attrition, the overall sample size will be 270 subjects.

21.3 Study Populations

21.3.1 Intent-to-Treat Analysis Set (ITT)

All randomized subjects will be included in the "intent-to-treat" (ITT) analysis set and classified according to their intended treatment regardless of failure to complete the required follow-up examinations. Subjects excluded prior to randomization will be considered screening failures.

21.3.2 As Treated Analysis Set (AT)

The As Treated Analysis Set (AT) will consist of ITT subjects who receive at least 1 treatment of study device (Active or Sham). Subjects will be analyzed according to the study treatment received. The As Treated Analysis Set will be used for all summaries of safety and tolerability data.

21.3.3 Modified Intent-to-Treat Analysis Set (mITT)

The modified intent-to-treat (mITT) analysis set will include ITT subjects who receive at least 1 treatment of study device (Active or Sham) and will exclude patients who were randomized in error. The number of exclusions from the mITT analysis set is expected to be small and will only be based on information that was known or could have been known prior to the first treatment. Subjects in the mITT population will be analyzed in the group to which they were randomized.

21.3.4 Per Protocol Analysis Set (PP)

The Per Protocol (PP) analysis set will be comprised of the following:

- Subjects in the mITT analysis set
- Subjects with device failure within the 10-week follow-up period
- Subjects with deviation from the clinical investigation plan is caused by the investigational device or by problems with respect to tolerability

Subjects who will be excluded from the PP analysis set are as follows:

- Subjects with major protocol violations that would be expected to confound clinical assessment during follow-up as determined by the PIs.

- Subjects who took new pharmaceutical substances or treatments during the clinical investigation which are listed as an exclusion criteria;

- Subjects who do not meet the inclusion criteria or exclusion criteria.
- Users who have performed less than 10 sessions (300 minutes) during the first 3 weeks will be excluded from the analysis.

Subjects in the PP population will be analyzed in the group to which they were randomized.

21.4 Randomization

After a subject has met all inclusion criteria and none of the exclusion criteria, and creates an account in the Flow app, the subject will be randomized. Randomization will be performed in a 1:1 ratio to the Active and Sham groups. Randomization will be performed using randomly permuted block sizes of 4 and 6 and done independently for the two sites.

21.5 Testing of Primary Effectiveness Hypotheses

The primary effectiveness endpoint is the change in adjusted mean group difference in the HDRS-17 scores between 10 weeks and baseline between subjects randomized to the active study device relative to those subjects randomized to sham. The formal statistical hypothesis is as follows:

H_o: $d_{flow} – d_{sham} \leq 0$
H_a: $d_{flow} – d_{sham} > 0$

Where d_{flow} and d_{sham} are the adjusted mean group difference in HDRS-17 scores in subjects randomized to Active and Sham groups, respectively.

The mITT population will be the analysis population used to analyze the primary effectiveness endpoint. The endpoint will be analyzed when all randomized subjects have completed their 10-week visit, been withdrawn from the trial or passed the end of their 10-week follow-up visit window. The difference in the group mean differences will be assessed using a mixed model for repeated measures. If the p-value of the difference in the group mean differences is less than one-sided p= 0.025, then the trial declared a success.

21.5.1 Multiple Imputation Analysis

The mixed model for repeated measures (MMRM) allows for inclusion of patients with missing Week 10 values. If all subjects have at least one follow-up evaluation, the following MMRM will be utilized for the primary superiority test. If there are subjects without at least one post baseline assessment, the multiple imputation analysis summarized below will be the primary analysis method. Least squares estimated values from the MMRM will be reported for HDRS-17 at each visit and as such these estimates include implicit imputations of missing values.
Details of the MMRM used to evaluate the primary endpoint are described in the attached Statistical analysis plan.

21.6 Missing Data/Sensitivity Analysis

Details of the handling are in the attached Statistical analysis plan.

21.7 Covariates/Covariate Analysis

The heterogeneity of treatment effects for the primary effectiveness endpoint will be examined for subgroups using baseline variables. These will include, but are not limited to, age (split at median), and sex at birth. Subjects on antidepressants and/or psychotherapy drugs during the course of the trial will constitute their own subgroup and be characterized and described in the clinical study report. The mixed model for repeated measures will be run to see the impact of the treatment effect by each subgroup of interest separately. The results will be reported as mean differences and 95% confidence intervals for each subgroup. These results will be graphically displayed in a forest plot.

21.8 Secondary Effectiveness Endpoints

Secondary effectiveness endpoints will be analyzed descriptively.

For continuous variables (CONT), results will be summarized with the numbers of observations, means, standard deviation, median, minimums and maximums. Differences between the two groups, where specified, will be summarized with the differences of the two means, and 95% confidence intervals for the difference between the means. These calculations will be done under the assumption that data for the two groups are independent and approximately normally distributed. The confidence interval for the difference of two means will be calculated under the assumption of unequal variances. If the asymptotic assumptions fail, then nonparametric summary statistics (medians, 25th and 75th percentile) may be displayed as an alternative.

For binary variables (BIN), results will be summarized with patient counts, percentages/rates. Differences between the two groups, where specified, will be summarized with the difference in percent and the Newcombe score 95% confidence interval for the difference of two percentages.

For correlation analysis, Spearman correlation will be used to assess the association between two continuous variables. The correlation coefficient and the 95% confidence intervals will be reported.

There are three secondary endpoints controlled multiplicity (see below):

- The mean change in points for MADRS score from baseline to Week 10. This will be evaluated using the same statistical methodology as the primary endpoint.
- Clinical Response will be analyzed as a responder endpoint. Clinical Response is defined as a >50% reduction from the baseline HDRS-17 or MADRS score, MADRS-s at Week 10.
- Remission will be analyzed as a responder endpoint where remission is defined as an HDRS-17 score ≤7 or a MADRS score ≤10). The endpoint will be tested using a two-group large-sample normal approximation test of proportions with a one-sided 0.025 considered significant.
- Quality of life improvement as measured by EQ-5D-3L in the active compared to sham arm at week 10 compared to baseline.

21.9 Exploratory Endpoints

The following list of secondary endpoints will be analyzed using the mITT population and will be summarized based on the type of data: Continuous (CONT), Binary (BIN) or Correlation (CORR):

- Montgomery–Åsberg Depression Rating Scale (MADRS) response rate at all follow-up time points (CONT)

- Montgomery–Åsberg Depression Rating Scale - Self-report (MADRS-s) scores of the two arms (response and remission rates) at all time points (CONT)
- Hamilton Anxiety Rating Scale (HAM-A) at all time points (CONT)
- Young Mania Rating Scale (YMRS) at all time points (CONT)
- tDCS Adverse Events Questionnaire (AEQ) at 10 and 20 weeks (CONT)
- HDRS-17 (CONT), MADRS (CONT), MADRS-s mean decrease (CONT), remission (BIN), and response rate (BIN) at 6 weeks comparison between groups.
- HDRS-17 (CONT), MADRS (CONT), MADRS-s (CONT) at 20 weeks (after open-label)
- Correlation between MADRS and MADRS-s. (CORR)
- Correlation between the percent of sessions using the FLOW FL-100 and HDRS-17/MADRS decrease in the active group at 10 weeks.

- The within-patient clinically meaningful improvement as defined as at least -3 points on the HDRS-17 scale. The percentage of subjects for each arm that reaches -3 points or more improvement and also the Cumulative Distribution Function Curves of Change in HDRS-17 Score from Baseline to Primary end-point by arm.

21.10 Multiplicity Adjustment

The secondary endpoints will only be tested if the primary endpoint demonstrates superiority. By pre-specifying the order in which the primary and conditional effectiveness hypotheses are performed, there is no type 1 error inflation and nominal alpha levels may be used on the primary endpoint.

Should the primary endpoint be met, the clinical response endpoint (Endpoint #1 in SAP version 2) will be tested. Should the secondary Endpoint #1 be met, the remaining endpoints will be tested using Hochberg correction for multiplicity. Complete details for multiplicity adjustment are provided in the SAP.

21.11 Evaluation of Blinding

An evaluation of blinding will be performed. Prior to subjects unblinding through the system they will respond to the question “Which treatment arm do you think you are in?” (answers: sham/active), followed by the question “How certain are you of this?”, with the options 1 to 5 where 5 is very certain and 1 is very uncertain. The counts and percentages of the subjects responding correctly will be tabulated and reported.

21.12 Device Survival

Kaplan-Meier survival analysis will be used to characterize device failure time. Device failure in this study is defined as the device not operating as intended without unexpected failure. Unexpected failure is defined as a failure that is not related to device functionality. For example, the device accidentally falling out of a window would be categorized as an unexpected device failure. In contrast, the device failing to turn on would be considered an expected device failure. As the device is not expected to have any unexpected failures, unexpected failures will only be reported if they occur. Lifetables will be tabulated indicating the number of “failures" and the number of “at- risk” subjects over time for all failures, unexpected failures and expected failures. If there are more than one of each expected and unexpected failures, a log-rank test will be performed to compare the device failure time distribution.

21.13 Poolability Analysis

Poolability will be assessed through stratification of the primary endpoint by Country (OUS and US). Within country point estimates will be calculated along with nominal descriptive 95% Confidence Intervals. The focus of this analysis will be on the similarity of the country specific point estimates. Differences will be described in the Clinical Study Report.

Statistical evaluation of the primary endpoint will be evaluated by adding site by treatment group term to the primary MMRM model. A p-value of <= 0.10 will be considered significant for this poolability assessment. Should a significant site x treatment group interaction be present, the patient factors of age, gender, and baseline scores will be assessed to understand if differences in case-mix factors explain differences observed between sites.

21.14 Safety Analyses

Assessment of the safety of the investigational device will include an evaluation of the incidence and severity of complications and adverse reactions associated with the treatment.

Adverse event rates will be summarized by type of AE and for specific AEs in two ways:

1. per subject incidence of specific AEs and classes of AEs and
2. by event, summarizing event counts by visit interval over time and in accordance with FDA Guidance (CDRH 2004)[15].

Device and procedure-related events will be summarized by severity and relatedness. Events listings that include details such as relatedness, severity, onset, and resolution status will be provided for all events and relevant subsets of events such as serious events and related events. Primary safety comparisons will be performed using the AT analysis set. The summary tables will show the adverse events (in coded terms), the total number of events, and the number and the percentage of subjects affected in each treatment group ("subject wise evaluation") and stratified by relation to device and severity. Data of dropouts will be presented separately, and possible bias will be discussed in the final report.

All AEs will be summarized in listings for the AT analysis set. The following AE listings will be constructed:

- AE Listing 1 All AEs Sorted by Group and Event Type
- AE Listing 2 All AEs Sorted by Group and Subject
- AE Listing 3 Serious AEs
- AE Listing 4 Severe AEs
- AE Listing 5 Device Related AEs
- AE Listing 6 Serious Device Related AEs
- AE Listing 7 Severe Device Related AEs
- AE Listing 8 Other AEs
- AE Listing 9 AEs among subjects discontinued early
- AE Listing 10 AEs among subjects who died

[15] CDRH. Guidance for Industry and FDA Staff: Clinical Data Presentations for Orthopedic Device Applications, December 2, 2004.

21.15 Interim Analyses

An interim analysis testing for futility, sample size re-estimation, and claiming study success with a sample size smaller than the maximum specified is specified. Please see the details in the Adaptive Design Report detailing all elements of this process.

22 Ethics & Protection of Human Subjects

22.1 Ethical Review

The study will be performed in accordance with the standard guidance on clinical investigations with medical devices on human subjects and recommendations guiding physicians in biomedical research involving human subjects adopted by the 18th World Medical Assembly, Helsinki, Finland, 1964 and later revisions.

The clinical investigational plan, informed consent, any other study specific study documents as required by regulations and all amendments to these study documents will be reviewed and approved by the FDA and appropriate IRB/EC before enrollment of any patient. In addition, the sponsor will keep the regulatory authorities informed of any SAEs throughout the study course, if applicable.

22.2 Regulatory Considerations

This study will be conducted in accordance with the Good Clinical Practice (GCP) guidelines and other applicable regulatory requirements including but not limited to:

- FDA Regulations on Investigational Device Exemption (21 CFR 812)
- FDA Regulations on research with human beings (21 CFR 50, 54 and 56)
- Health and Human Services (DHHS) Regulations on research with human beings (45 CFR 46 Subparts A, B, C, and D)
- International Conference on Harmonization (ICH) Guidance for Industry-E6 Good Clinical Practice: Consolidated Guideline
- The Declaration of Helsinki

22.3 Study Amendments

All changes to the protocol that impact validity of data, scientific soundness, the rights safety, or welfare of the participants, must be documented in the format of an amendment with justification statements in the cover letter to the IRB/EC and FDA. All amendments must be submitted to the IRB/EC and regulatory authority for review and approval. Following approval, the protocol amendment will be distributed to all protocol recipients at each investigational site.

22.4 Insurance

The Sponsor has issued clinical trial insurance with appropriate coverage for the continuation of the entire study.

23 Data Handling and Recordkeeping

23.1 EDC and eCRFs

eCRFs will be completed in a 21 CFR Part 11 compliant electronic data capture (EDC) system for each patient enrolled into the clinical study. The study coordinator will enter the source data onto the eCRFs, and study monitors will verify that the source documents match the eCRFs. Once verified, the study monitor will lock the field via a monitoring approval step so the data cannot be changed unless requested.

All procedures for the handling and analysis of data will be conducted using good clinical data management practices within systems meeting FDA guidelines for the handling, storage, and analysis of data for clinical studies.

The Investigator will sign off on the Eligibility Criteria, all Adverse Event, Protocol Deviation and Study Completion eCRFs and will sign each patient's casebook once the patient completes the study to attest that all data entered on the eCRFs are complete and accurate. All of the above signatures will be completed digitally within the EDC system using the system's Part 11 compliant digital signature system function.

Any required data clarifications will be handled within the EDC system's query management system. The EDC will be programmed to automatically place data clarification queries on missing values and values out of acceptable ranges. Study monitors and data managers will also be able to add data clarification queries to data points within the system. Study coordinators will have the opportunity to correct data and/or respond to the query for review by monitors and data managers.

23.2 Capture of patient reported outcomes

Patient reported outcomes (PROs) will be collected via the participant's personal smartphone or other device running Android 5.0+ or iOS 12+ and the data will be transmitted directly into the EDC. As the data is originated in the participant's device, it is considered to be electronic source and the data is automatically locked as soon as it enters the EDC to ensure that it is secure.

23.3 Study Records Retention

The Investigator and the Sponsor will maintain accurate, complete, and current records relating to participation in this clinical investigation. If the Investigator wishes to assign the responsibility of maintaining the study files to someone else or move them to another location, he/she should consult with the study sponsor in writing regarding the change. The Investigator will take measures to ensure that these essential documents are not accidentally damaged or destroyed.

Upon study completion, the study files must be maintained in a known location for a maximum of a 2 years following the date a marketing application is approved for the medical device for the indication for which it is being investigated; or, if no application is to be filed or if the application is

not approved for such indication, until 2 years after the investigation is discontinued and FDA is notified.

The Investigator and Sponsor will maintain records in accordance with 21 CFR 812, Subpart G, to include:

- Current and past versions of the IRB/EC-approved clinical protocol and amendments and corresponding IRB/EC-approved consent form(s) and, if applicable, participant recruitment advertisements
- FDA correspondence related to the IDE application; including supplemental IDE applications, current investigator lists, progress reports
- IRB/EC correspondence (including submissions and approval notifications) including safety and protocol deviation reports, and annual or interim reports
- Signed Clinical Trial Agreement
- Signed Investigator Agreements and financial disclosure forms for participating Investigators
- Curriculum vitae (Investigator and Sub-Investigators)
- Certificates of required training for Investigators and Sub-Investigators, including human subject protection and Good Clinical Practice
- Instructions for handling the investigational device and other study-related materials
- Device tracking logs
- Signed ICFs
- eCRF and Source Documents
- Monitoring visit confirmation and follow-up letters
- Copies of relevant Sponsor-Investigator correspondence, including notifications of adverse event information
- Screening and Enrollment Log
- Signed informed consent forms
- Final clinical study report

23.4 Publication and Data Sharing Policy

Data generated from the conduct of this study will be used to support an application by Flow Neuroscience AB as an original Premarket Approval (PMA) from FDA. Publication of the results of the study will follow Flow Neuroscience AB 's Publication and Presentation Policy.

23.5 Participant and Data Confidentiality

Participant confidentiality will be maintained throughout the study in a way that ensures that data can always be tracked back to the source data. For this purpose, a unique participant identification code (two-digit site identification number and three-digit participant name identification number code assigned sequentially) will be used that allows identification of all data reported for each participant without traceability back to the actual participant.

The Principal Investigator or institution shall provide direct access to source data during and after the clinical investigation for monitoring, auditing, IRB/EC review and regulatory authority inspections.

For investigational sites within the United States, “Protected Health Information” will be treated and maintained in compliance with the Health Insurance Portability and Accountability Act of 1996 (HIPAA) Privacy rule.

The duration of storage time of personal data at the investigational sites will be in accordance with national regulations. Electronic Case Report Form (eCRF) data captured and maintained in Curebase for each participant. Curebase stores all data in a secure cloud server of Amazon Web Services (AWS) and complies with most of HIPAA and GDPR standards, including but not limited to: password protection, platform encryption, and 2-factor verification. Curebase takes as much action as possible to maximize the protection and security of subjects’ sensitive information. All data pertaining to a clinical study, including protected health information (PHI), will be retained for the duration of the study, and for 2 years thereafter, in compliance with FDA monitoring obligations. At this point, the identifiable PHI obtained during the study will be deleted. Curebase will share pseudonymized data collected from subjects with authorized representatives.
After 2 years have elapsed since the completion of a clinical investigation, Curebase will retain a de-identified version of the data obtained during that study. Curebase may retain this data indefinitely and may use it for a variety of purposes that will include, but not be limited to, making improvements to the Curebase product.

Information obtained while executing this study, including still and motion photography, may be presented for regulatory, clinical, or educational purposes if no participant is identified. The data collected is the property of Flow Neuroscience AB.

23.6 Investigator Records

The investigator is responsible for the preparation and retention of the records cited below. All these records, except for participant’s case history records, should be filed in the Investigator Site File.

23.7 Participant Records

- All Source Documentation Worksheets
- Supporting data (e.g., medical records, clinic charts)

23.8 Investigator Records and Reports

The investigator is responsible for the timely preparation and submission of the reports cited below.

Table 5: Investigator Reports

Report	Sent To	Timing of Report
Unanticipated adverse device effect (UADE)	Flow Neuroscience AB, IRB/EC	UADE must be reported as soon as possible, but in no event later than 10 business days after the investigator first learns of the event
Withdrawal of IRB/EC approval	Flow Neuroscience AB	Reported within 5 business days
Progress reports	Flow Neuroscience AB, IRB/EC	Reported as requested by Flow Neuroscience AB or the reviewing IRB/EC, but at least yearly
Deviations from the investigation plan	Flow Neuroscience AB, IRB/EC (FDA)	A deviation to protect the life or physical well-being of a participant in an emergency must be reported no later than 5 business days after the emergency occurred. Deviations that may affect the scientific soundness of the investigational plan or the rights, safety, or welfare of the participant (and are not an emergency) must receive prior approval by Flow Neuroscience AB and be reported to the FDA and IRB/ECs.
Device use without informed consent	Flow Neuroscience AB, IRB/EC	This deviation must be reported within 5 business days after the use occurs.
Final Report	Flow Neuroscience AB, IRB/EC	This report must be submitted within 3 months after termination or completion of the investigation or the investigator's part of the investigation.
Other	FDA, IRB/EC	Upon request, accurate, complete, and current information about any aspect of the investigation must be reported.
