# Supplementary material for: Home-based transcranial direct current stimulation treatment for major depressive disorder: a fully remote phase 2 randomized sham-controlled trial
Source: Nat Med. 2024 Oct 21;31(1):87–95. doi: 10.1038/s41591-024-03305-y (PMC11750699; doi:10.1038/s41591-024-03305-y)
Supplement: Supplementary file 2 — Reporting Summary [file 41591_2024_3305_MOESM2_ESM.pdf]

nature portfolio

Corresponding author(s): Cynthia H.Y. Fu

Last updated by author(s): Sep 10, 2024

Reporting Summary

Nature Portfolio wishes to improve the reproducibility of the work that we publish. This form provides structure for consistency and transparency in reporting. For further information on Nature Portfolio policies, see our Editorial Policies and the Editorial Policy Checklist.

Statistics

For all statistical analyses, confirm that the following items are present in the figure legend, table legend, main text, or Methods section.

n/a	Confirmed	
☐	☒	The exact sample size (*n*) for each experimental group/condition, given as a discrete number and unit of measurement
☐	☒	A statement on whether measurements were taken from distinct samples or whether the same sample was measured repeatedly
☐	☒	The statistical test(s) used AND whether they are one- or two-sided<br>*Only common tests should be described solely by name; describe more complex techniques in the Methods section.*
☐	☒	A description of all covariates tested
☐	☒	A description of any assumptions or corrections, such as tests of normality and adjustment for multiple comparisons
☐	☒	A full description of the statistical parameters including central tendency (e.g. means) or other basic estimates (e.g. regression coefficient) AND variation (e.g. standard deviation) or associated estimates of uncertainty (e.g. confidence intervals)
☐	☒	For null hypothesis testing, the test statistic (e.g. F, t, r) with confidence intervals, effect sizes, degrees of freedom and P value noted<br>*Give P values as exact values whenever suitable.*
☒	☐	For Bayesian analysis, information on the choice of priors and Markov chain Monte Carlo settings
☒	☐	For hierarchical and complex designs, identification of the appropriate level for tests and full reporting of outcomes
☐	☒	Estimates of effect sizes (e.g. Cohen's d, Pearson's r), indicating how they were calculated

Our web collection on statistics for biologists contains articles on many of the points above.

Software and code

Policy information about availability of computer code

Data collection	Data was collected via the Clinical Research Organisation, Curebase. The Curebase platform was used for completing informed consent, data collection and for the completion of self-report questionnaires by participants. The study specific installation of the Flow Neuroscience app collected data about tDCS stimulations and MADRS-s responses.
Data analysis	The clinical outcomes were analysed using SAS 9.4M8.The clinical outcomes were analysed using SAS 9.4M8. The analysis code for the longitudinal model is provided in the Supplementary Information. The full code used for the data analysis will be available from the Sponsor beginning 6 months after publication of the trial results.

For manuscripts utilizing custom algorithms or software that are central to the research but not yet described in published literature, software must be made available to editors and reviewers. We strongly encourage code deposition in a community repository (e.g. GitHub). See the Nature Portfolio guidelines for submitting code & software for further information.

Data

Policy information about availability of data

All manuscripts must include a data availability statement. This statement should provide the following information, where applicable:

- Accession codes, unique identifiers, or web links for publicly available datasets
- A description of any restrictions on data availability
- For clinical datasets or third party data, please ensure that the statement adheres to our policy

The deidentified individual participant data and the data dictionary that support the findings of this study are available from the academic researchers or the sponsor beginning 6months after publication because of legal reasons. However, restrictions apply to the availability of these data and so are not publicly available. The Statistical Analysis Plan is available in the Supplementary Materials. A data request and brief analysis plan will be required in accordance with Ethics Committee requirements. These will be reviewed by the lead, study steering committee and study sponsor. A data transfer agreement (DTA) will have to be completed prior to any data being shared. Following completion of the DTA, data will be shared as password-protected files. Data sharing will abide by the rules and policies defined by the sponsor, relevant institutional review boards, and local, state and federal laws and regulations. Rights and privacy of individuals participating in the research will be protected at all times. Approval will not be provided for commercial use of the data. Requests can be made to Professor Cynthia H.Y. Fu (c.fu@uel.ac.uk, cynthia.fu@kcl.ac.uk).

Research involving human participants, their data, or biological material

Policy information about studies with human participants or human data. See also policy information about sex, gender (identity/presentation), and sexual orientation and race, ethnicity and racism.

Reporting on sex and gender	We use the term sex throughout the main text and the Supplementary Information. Sex was self-reported by participants. Participants were asked their sex during the initial video screen and this was recorded by researchers. There was no upper limit on the number of men of women who could enroll. Results disaggregated by sex have been reported in the supplementary information. Sex and age of participants in the active and sham groups have been reported in Table 1 of the main text.
Reporting on race, ethnicity, or other socially relevant groupings	Participants were aged 18 and older with no upper age limit. There was no limit on sex or gender, race, ethnicity, marital status or social class. Sex, race and ethnicity were determined by self-report.
Population characteristics	Inclusion criteria: Participants were adults >=18 years, with MDD and in current depressive episode based on Diagnostic and Statistical Manual of Mental Disorders, Fifth Edition (DSM-5) criteria26 by structured assessment, Mini-International Neuropsychiatric Interview (MINI; Version 7.0.2). Inclusion criteria included: having at least moderate severity of depressive symptoms, as measured by score >=16 on 17-item Hamilton Depression Rating Scale (HDRS); being treatment-free or taking stable antidepressant medication or in psychotherapy for at least 6 weeks prior to enrolment and agreeable to maintaining same treatment throughout the trial; under care of GP or psychiatrist.<br>Exclusion criteria included: having treatment resistant depression, defined as inadequate clinical response to two or more trials of antidepressant medication at an adequate dose and duration; significant suicide risk based on Columbia Suicide Severity Rating Scale (C-SSRS) Triage and Risk Identification Screener; comorbid psychiatric disorder; taking medications that affect cortical excitability (e.g., benzodiazepines, epilepsy medication); and contraindications to tDCS. Full inclusion and exclusion criteria are presented in Supplementary Information document.
Recruitment	Participants were recruited through Flow Neuroscience website, email lists and social media posts. Individuals completed an online pre-screening form, hosted by a contract research organization (CRO), followed by a telephone call with a CRO member. Individuals then provided written informed consent and had an assessment with a research team member by Microsoft (MS) Teams videoconference. All participants in the trial self-referred to participate in the trial, which is potential for a self-selection bias.
Ethics oversight	The clinical trial received local approval at both study sites and ethical approval was provided by South Central-Hampshire B Research Ethics Committee, UK (ref. 22/SC/0023), and WIRB-Copernicus Group International Review Board, USA (ref. 1324775).

Note that full information on the approval of the study protocol must also be provided in the manuscript.

Field-specific reporting

Please select the one below that is the best fit for your research. If you are not sure, read the appropriate sections before making your selection.

☒ Life sciences ☐ Behavioural & social sciences ☐ Ecological, evolutionary & environmental sciences

For a reference copy of the document with all sections, see nature.com/documents/nr-reporting-summary-flat.pdf

Life sciences study design

All studies must disclose on these points even when the disclosure is negative.

Sample size	Sample size calculation was based on Brunoni et al, with two-sample t-test for mean difference with 80%

Sample size	power and one-sided Type 1 error 0.025, resulting in a sample size of 176 MDD participants. To increase power to 87.6%, sample size was increased to 216. Assuming 20% attrition rate, total sample size was 270 participants. A pre-specified interim analysis was performed when 90 MDD participants completed week 10, which included both futility assessment and sample size re-estimation. The interim analysis was able to modify the trial in two ways for the primary endpoint, to declare the trial futile and stop enrolment or to specify the number of participants between 100 and 270 for powering the trial based on promising zone methodology.
Data exclusions	368 participants were assessed for eligibility via video conference and 194 participants were excluded.<br>174 participants were randomized to active or sham treatment groups. One participant in the sham treatment group did not received any stimulations and therefore was not included in the modified intention-to-treat analysis.<br>Inclusion criteria: Participants were adults >=18 years, with MDD in current depressive episode based on Diagnostic and Statistical Manual of Mental Disorders, Fifth Edition (DSM-5) criteria by structured assessment, Mini-International Neuropsychiatric Interview (MINI; Version 7.0.2). Inclusion criteria included: at least a moderate severity of depressive symptoms, as measured by score >=16 on 17-item Hamilton Depression Rating Scale (HDRS); being treatment free, or taking stable antidepressant medication, or in psychotherapy, for at least 6 weeks prior to enrolment, and agreeable to maintaining same treatment throughout the trial; under care of GP or psychiatrist.<br>Exclusion criteria: having treatment resistant depression, defined as inadequate clinical response to two or more trials of antidepressant medication at an adequate dose and duration; significant suicide risk based on Columbia Suicide Severity Rating Scale (C-SSRS) Triage and Risk Identification Screener; comorbid psychiatric disorder; taking medications that affect cortical excitability (e.g., benzodiazepines, epileptics); and contraindications to tDCS.
Replication	We did not repeat the study to test the reproducibility because it is a large multicentre trial. We have described all procedures in detail to allow for it to be reproduced.
Randomization	The trial consisted of a 10-week blinded treatment phase followed by 10-week open label phase. The blinded phase consisted of random assignment to sham or active tDCS treatment in 1:1 ratio, performed independently at each site. Block randomization was used with permuted block sizes of 4 and 6, conducted by the trial server and stored in dedicated database.
Blinding	Participants and research team members were blind to group allocation. We sought to have same research team member present for same participant at each study visit. A second research team member joined clinical reviews for independent ratings and would not be present whilst adverse events or stimulation was discussed in order to prevent any potential bias. Ratings were crosschecked and reviewed by principal site investigators. At week 10, following completion of all assessments and prior to unblinding, participants were asked whether they thought they had been using the 'active' or 'inactive' tDCS device and how certain they were, as measured by a rating on a scale from 1 (very uncertain) to 5 (very certain). Once this had been completed, then the research team member accessed the online remote-monitoring system to unblind allocation and informed the participant of group allocation. At point of unblinding, an automatic email notification was sent to principal investigator and research team members that unblinding had occurred.

Reporting for specific materials, systems and methods

We require information from authors about some types of materials, experimental systems and methods used in many studies. Here, indicate whether each material, system or method listed is relevant to your study. If you are not sure if a list item applies to your research, read the appropriate section before selecting a response.

Materials & experimental systems

n/a	Involved in the study
☒	☐ Antibodies
☒	☐ Eukaryotic cell lines
☒	☐ Palaeontology and archaeology
☒	☐ Animals and other organisms
☐	☒ Clinical data
☒	☐ Dual use research of concern
☒	☐ Plants

Methods

n/a	Involved in the study
☒	☐ ChIP-seq
☒	☐ Flow cytometry
☒	☐ MRI-based neuroimaging

Clinical data

Policy information about clinical studies

All manuscripts should comply with the ICMJE guidelines for publication of clinical research and a completed CONSORT checklist must be included with all submissions.

Clinical trial registration	Clinicaltrail.gov reference: NCT05202119. CONSORT 2010 checklist included.
Study protocol	The study protocol is available in the Supplementary Information.
Data collection	Participants were recruited through Flow Neuroscience website, email lists and social media posts. Individuals completed an online pre-screening form, hosted by a contract research organization (CRO), followed by a telephone call with a CRO member. Individuals then provided written informed consent and had an assessment with a research team member by Microsoft (MS) Teams videoconference. All subsequent appointments with the research team took place by MS Teams videoconference. tDCS stimulation was provided using a study specific installation of the app which connected to headset via Bluetooth. Researchers had access to remote monitoring with real-time data use to monitor compliance. Researchers received training to use the headset and were

present by videoconference for initial session to support participants who were at home, with the app-guided training to demonstrate electrode placement, consisting of video and augmented reality via device camera. All remaining tDCS sessions were completed by participants at home, without the presence of a researcher. Participants were asked to have video and microphone on during initial session. Participants were advised to sit or lie down during use, not to use the headset outdoors, close to water, whilst driving, during any activity that could lead to a significant risk of injury, while intoxicated or incapacitated, or in environments with strong magnetic fields. MADRS-s was completed by participants in their own time on the study app. All other clinical assessments and data were collected during videoconference with researchers. Recruitment was from May 12, 2022 to March 10, 2023. Final open-label follow up was conducted on August 23, 2023.

Outcomes

Primary effectiveness outcome was estimated mean group difference in HDRS scores in participants randomized to active and sham treatments using a mixed model for repeated measures (MMRM). The model included the HDRS baseline value, antidepressant medication status, psychotherapy treatment, age, and sex. Missing data were categorized by the reason for missingness (missing at random or not) and differentially imputed based on that classification. If p-value were less than one-sided $p = 0.025$, then endpoint would be declared positive (SAP sections 3.1 to 3.1.4. sections 4 and 5).

MMRM allows for inclusion of data from all time points in the model and not only baseline and week 10 end of treatment values, and MMRM allows for inclusion of participants with missing week 10 values. The MMRM approach is a direct likelihood approach. MMRM parameters were estimated using SAS PROC MIXED (SAS Institute, Cary NC Version 9.4 or higher). In a matrix equation, the MMRM can be expressed as: $Yi = Xi\beta + Ziu + ei$; where β is the vector of fixed-effect regression parameters (for the overall mean change, the treatment effect θ, a vector of post-baseline time effects τ, a vector of treatment-by-time interaction effects η, and a vector of covariate effects ϕ that includes baseline HDRS-17 and optionally, other a priori selected covariates). X is a design matrix for the fixed effects, Z is a design matrix used to account for other random effects u, if any were included. Key assumptions are about e, the random error vector. It is assumed that the expected values are zero, i.e., $E(e) = 0$. An unstructured covariance is assumed requiring estimation of variances at each visit and all pairwise covariances, i.e., Var(e)=σe2Vunstructured.

If the primary endpoint is met, then secondary endpoints can be tested based on a hierarchical approach. As specified in the protocol, Hochberg65,66 approach was used for controlling multiplicity (Supplementary Table 11). The Hochberg correction rank orders the endpoints based on the p-value size, ranking them from largest to smallest, and compares those values to a sequentially decreasing alpha-level to determine whether the null hypothesis should be rejected. Secondary outcomes were: HDRS clinical response and remission, EQ-5D-3L change, and change in ratings, response and remission in MADRS and MADRS-s (SAP sections 3.1.5 to 3.1.9).

Plants

Seed stocks

N/A

Novel plant genotypes

N/A

Authentication

N/A
